# Supplementary material for: Systematic review on the compliance of WHO guidelines in the management of patients with advanced HIV disease in Africa: The case of cryptococcal antigen screening
Source: PLoS One. 2025 Jan 24;20(1):e0313453. doi: 10.1371/journal.pone.0313453 (PMC11761098; doi:10.1371/journal.pone.0313453)
Supplement: S2 Table — (DOCX) [file pone.0313453.s002.docx]

| **S2 Table: Systematic Review Search Strategies – cryptococcal antigen screening in people with advanced HIV in low- and middle-income countries** |
| --- |
| Final search run on 20 Mar 2024 by Emilie Ludeman, MSLIS  Total references on 20 Mar 2024: 2,688 references  Total following de-duplication in Covidence: 1,845 references  Filters / Limits: publication date 2005 – present  **PubMed – 1,027** references retrieved on 20 Mar 2024  (cryptococcal meningitis[tiab] OR cryptococcus meningitis[tiab] OR cryptococcal antigen[tiab] OR CrAG[tiab] OR cryptococcal antigenemia[tiab] OR cryptococcal antigenaemia[tiab] OR cryptococcosis[tiab] OR cryptococcal disease[tiab] OR "Meningitis, Cryptococcal"[mesh] OR "Cryptococcosis"[Mesh]) AND  (HIV[tiab] OR HIV-positive[tiab] OR HIV/AIDS[tiab] OR PLWH[tiab] OR PLHIV[tiab] OR PLWHIV[tiab] OR human immunodeficiency virus[tiab] OR "HIV Infections"[mesh] OR "HIV"[mesh]) AND  (developing country[tiab] OR developing countries[tiab] OR "Developing Countries"[Mesh] OR low income country[tiab] OR low-middle income countries[tiab] OR lower-middle income countries[tiab] OR middle income country[tiab] OR low income countries[tiab] OR middle income countries[tiab] OR low-and-middle-income countries[tiab] OR LMIC[tiab] OR low income econom*[tiab] OR low-middle income econom*[tiab] OR lower-middle income econom*[tiab] OR low-and-middle-income econom*[tiab] OR middle income econom*[tiab] OR Afghanistan[tw] OR Gambia[tw] OR Nepal[tw] OR Bangladesh[tw] OR Guinea[tw] OR Niger[tw] OR Benin[tw] OR Guinea-Bissau[tw] OR Rwanda[tw] OR Burkina Faso[tw] OR Haiti[tw] OR Sierra Leone[tw] OR Burundi[tw] OR Kenya[tw] OR Somalia[tw] OR Cambodia[tw] OR Korea[tw] OR Tajikistan[tw] OR Central African Republic[tw] OR Liberia[tw] OR Tanzania[tw] OR Chad[tw] OR Madagascar[tw] OR Togo[tw] OR Comoros[tw] OR Malawi[tw] OR Uganda[tw] OR Congo[tw] OR Mali[tw] OR Zimbabwe[tw] OR Eritrea[tw] OR Mozambique[tw] OR Ethiopia[tw] OR Myanmar[tw] OR Armenia[tw] OR Kiribati[tw] OR (São Tomé and Principe[tw]) OR Bhutan[tw] OR Kosovo[tw] OR Senegal[tw] OR Bolivia[tw] OR Kyrgyz Republic[tw] OR Solomon Islands[tw] OR Cameroon[tw] OR Lao PDR[tw] OR Laos[tw] OR South Sudan[tw] OR Cabo Verde[tw] OR Lesotho[tw] OR Sri Lanka[tw] OR Mauritania[tw] OR Sudan[tw] OR Côte d'Ivoire[tw] OR Micronesia[tw] OR Swaziland[tw] OR Djibouti[tw] OR Moldova[tw] OR Syrian Arab Republic[tw] OR Syria[tw] OR Egypt[tw] OR Mongolia[tw] OR Timor-Leste[tw] OR El Salvador[tw] OR Morocco[tw] OR Ukraine[tw] OR Georgia[tw] OR Nicaragua[tw] OR Uzbekistan[tw] OR Ghana[tw] OR Nigeria[tw] OR Vanuatu[tw] OR Guatemala[tw] OR Pakistan[tw] OR Vietnam[tw] OR Guyana[tw] OR Papua New Guinea[tw] OR West Bank and Gaza[tw] OR Gaza[tw] OR Honduras[tw] OR Paraguay[tw] OR Yemen[tw] OR Indonesia[tw] OR Philippines[tw] OR Zambia[tw] OR India[tw] OR Samoa[tw] OR Angola[tw] OR Fiji[tw] OR Palau[tw] OR Albania[tw] OR Gabon[tw] OR Panama[tw] OR Algeria[tw] OR Grenada[tw] OR Peru[tw] OR American Samoa[tw] OR Hungary[tw] OR Romania[tw] OR Argentina[tw] OR Iran[tw] OR Serbia[tw] OR Azerbaijan[tw] OR Iraq[tw] OR Seychelles[tw] OR Belarus[tw] OR Jamaica[tw] OR South Africa[tw] OR Belize[tw] OR Jordan[tw] OR St. Lucia[tw] OR (Bosnia and Herzegovina[tw]) OR Bosnia[tw] OR Kazakhstan[tw] OR (St. Vincent and the Grenadines[tw]) OR Botswana[tw] OR Lebanon[tw] OR Suriname[tw] OR Brazil[tw] OR Libya[tw] OR Thailand[tw] OR Bulgaria[tw] OR Macedonia[tw] OR Tonga[tw] OR China[tw] OR Malaysia[tw] OR Tunisia[tw] OR Colombia[tw] OR Maldives[tw] OR Turkey[tw] OR Costa Rica[tw] OR Marshall Islands[tw] OR Turkmenistan[tw] OR Cuba[tw] OR Mauritius[tw] OR Tuvalu[tw] OR Dominica[tw] OR Mexico[tw] OR Venezuela[tw] OR Dominican Republic[tw] OR Montenegro[tw] OR Ecuador[tw] OR Namibia[tw])  **Embase** – 1,563 references retrieved on 20 Mar 2024  ('cryptococcal meningitis':ab,ti OR 'cryptococcus meningitis':ab,ti OR 'cryptococcal antigen':ab,ti OR CrAG:ab,ti OR 'cryptococcal antigenemia':ab,ti OR 'cryptococcal antigenaemia':ab,ti OR cryptococcosis:ab,ti OR 'cryptococcal disease':ab,ti OR 'cryptococcal meningitis'/exp OR 'cryptococcosis'/de OR 'cryptococcus test kit'/de) AND  (HIV:ab,ti OR HIV-positive:ab,ti OR HIV*AIDS:ab,ti OR PLWH:ab,ti OR PLHIV:ab,ti OR PLWHIV:ab,ti OR 'human immunodeficiency virus':ab,ti OR 'Human immunodeficiency virus'/exp OR 'acute HIV infection'/de) AND  (‘developing countr*’:ab,ti OR ‘developing country’/syn OR ‘low income country’/syn OR ‘middle income country’/syn OR ‘low income countr*’:ab,ti OR ‘low-middle income countries’:ab,ti OR ‘lower-middle income countries’:ab,ti OR ‘middle income countr*’:ab,ti OR ‘low-and-middle-income countries’:ab,ti OR LMIC:ab,ti OR ‘low income econom*’:ab,ti OR ‘low-middle income econom*’:ab,ti OR ‘lower-middle income econom*’:ab,ti OR ‘low-and-middle-income econom*’:ab,ti OR ‘middle income econom*’:ab,ti OR Afghanistan:de,ab,ti OR Gambia:de,ab,ti OR Nepal:de,ab,ti OR Bangladesh:de,ab,ti OR Guinea:de,ab,ti OR Niger:de,ab,ti OR Benin:de,ab,ti OR Guinea-Bissau:de,ab,ti OR Rwanda:de,ab,ti OR ‘Burkina Faso’:de,ab,ti OR Haiti:de,ab,ti OR ‘Sierra Leone’:de,ab,ti OR Burundi:de,ab,ti OR Kenya:de,ab,ti OR Somalia:de,ab,ti OR Cambodia:de,ab,ti OR Korea:de,ab,ti OR Tajikistan:de,ab,ti OR ‘Central African Republic’:de,ab,ti OR Liberia:de,ab,ti OR Tanzania:de,ab,ti OR Chad:de,ab,ti OR Madagascar:de,ab,ti OR Togo:de,ab,ti OR Comoros:de,ab,ti OR Malawi:de,ab,ti OR Uganda:de,ab,ti OR Congo:de,ab,ti OR Mali:de,ab,ti OR Zimbabwe:de,ab,ti OR Eritrea:de,ab,ti OR Mozambique:de,ab,ti OR Ethiopia:de,ab,ti OR Myanmar:de,ab,ti OR Armenia:de,ab,ti OR Kiribati:de,ab,ti OR ‘São Tomé and Principe’:de,ab,ti OR Bhutan:de,ab,ti OR Kosovo:de,ab,ti OR Senegal:de,ab,ti OR Bolivia:de,ab,ti OR ‘Kyrgyz Republic’:de,ab,ti OR ‘Solomon Islands’:de,ab,ti OR Cameroon:de,ab,ti OR ‘Lao PDR’:de,ab,ti OR Laos:de,ab,ti OR ‘South Sudan’:de,ab,ti OR ‘Cabo Verde’:de,ab,ti OR Lesotho:de,ab,ti OR ‘Sri Lanka’:de,ab,ti OR Mauritania:de,ab,ti OR Sudan:de,ab,ti OR ‘Côte d?Ivoire’:de,ab,ti OR Micronesia:de,ab,ti OR Swaziland:de,ab,ti OR Djibouti:de,ab,ti OR Moldova:de,ab,ti OR ‘Syrian Arab Republic’:de,ab,ti OR Syria:de,ab,ti OR Egypt:de,ab,ti OR Mongolia:de,ab,ti OR Timor-Leste:de,ab,ti OR ‘El Salvador’:de,ab,ti OR Morocco:de,ab,ti OR Ukraine:de,ab,ti OR Georgia:de,ab,ti OR Nicaragua:de,ab,ti OR Uzbekistan:de,ab,ti OR Ghana:de,ab,ti OR Nigeria:de,ab,ti OR Vanuatu:de,ab,ti OR Guatemala:de,ab,ti OR Pakistan:de,ab,ti OR Vietnam:de,ab,ti OR Guyana:de,ab,ti OR ‘Papua New Guinea’:de,ab,ti OR ‘West Bank and Gaza’:de,ab,ti OR Gaza:de,ab,ti OR Honduras:de,ab,ti OR Paraguay:de,ab,ti OR Yemen:de,ab,ti OR Indonesia:de,ab,ti OR Philippines:de,ab,ti OR Zambia:de,ab,ti OR India:de,ab,ti OR Samoa:de,ab,ti OR Angola:de,ab,ti OR Fiji:de,ab,ti OR Palau:de,ab,ti OR Albania:de,ab,ti OR Gabon:de,ab,ti OR Panama:de,ab,ti OR Algeria:de,ab,ti OR Grenada:de,ab,ti OR Peru:de,ab,ti OR ‘American Samoa’:de,ab,ti OR Hungary:de,ab,ti OR Romania:de,ab,ti OR Argentina:de,ab,ti OR Iran:de,ab,ti OR Serbia:de,ab,ti OR Azerbaijan:de,ab,ti OR Iraq:de,ab,ti OR Seychelles:de,ab,ti OR Belarus:de,ab,ti OR Jamaica:de,ab,ti OR ‘South Africa’:de,ab,ti OR Belize:de,ab,ti OR Jordan:de,ab,ti OR ‘St. Lucia’:de,ab,ti OR ‘Bosnia and Herzegovina’:de,ab,ti OR Bosnia:de,ab,ti OR Kazakhstan:de,ab,ti OR ‘St. Vincent and the Grenadines’:de,ab,ti OR Botswana:de,ab,ti OR Lebanon:de,ab,ti OR Suriname:de,ab,ti OR Brazil:de,ab,ti OR Libya:de,ab,ti OR Thailand:de,ab,ti OR Bulgaria:de,ab,ti OR Macedonia:de,ab,ti OR Tonga:de,ab,ti OR China:de,ab,ti OR Malaysia:de,ab,ti OR Tunisia:de,ab,ti OR Colombia:de,ab,ti OR Maldives:de,ab,ti OR Turkey:de,ab,ti OR ‘Costa Rica’:de,ab,ti OR ‘Marshall Islands’:de,ab,ti OR Turkmenistan:de,ab,ti OR Cuba:de,ab,ti OR Mauritius:de,ab,ti OR Tuvalu:de,ab,ti OR Dominica:de,ab,ti OR Mexico:de,ab,ti OR Venezuela:de,ab,ti OR ‘Dominican Republic’:de,ab,ti OR Montenegro:de,ab,ti OR Ecuador:de,ab,ti OR Namibia:de,ab,ti)  **CENTRAL** – 98 references retrieved on 20 Mar 2024  *Advanced Search using Search manager*  ("cryptococcal meningitis" OR "cryptococcus meningitis" OR "cryptococcal antigen" OR CrAG OR "cryptococcal antigenemia" OR "cryptococcal antigenaemia" OR cryptococcosis OR "cryptococcal disease"):ab,ti,kw OR [mh "Meningitis, Cryptococcal"] OR [mh "Cryptococcosis"] AND  (HIV OR HIV-positive OR HIV*AIDS OR PLWH OR PLHIV OR PLWHIV OR "human immunodeficiency virus"):ab,ti,kw OR [mh "HIV Infections"] OR [mh "HIV"] AND  ([mh "Developing Countries"] OR (“developing country” OR “developing countries” OR “low income country” OR “low middle income countries” OR “lower middle income countries” OR “middle income country” OR “low income countries” OR “middle income countries” OR “low and middle income countries” OR LMIC OR (“low income” NEXT econom*) OR (“low middle income” NEXT econom*) OR (“lower middle income” NEXT econom*) OR (“low and middle income” NEXT econom*) OR (“middle income” NEXT econom*)):ab,ti,kw OR (Afghanistan OR Gambia OR Nepal OR Bangladesh OR Guinea OR Niger OR Benin OR Guinea?Bissau OR Rwanda OR “Burkina Faso” OR Haiti OR “Sierra Leone” OR Burundi OR Kenya OR Somalia OR Cambodia OR Korea OR Tajikistan OR “Central African Republic” OR Liberia OR Tanzania OR Chad OR Madagascar OR Togo OR Comoros OR Malawi OR Uganda OR Congo OR Mali OR Zimbabwe OR Eritrea OR Mozambique OR Ethiopia OR Myanmar OR Armenia OR Kiribati OR “São Tomé and Principe” OR Bhutan OR Kosovo OR Senegal OR Bolivia OR “Kyrgyz Republic” OR “Solomon Islands” OR Cameroon OR “Lao PDR” OR Laos OR “South Sudan” OR “Cabo Verde” OR Lesotho OR “Sri Lanka” OR Mauritania OR Sudan OR (Côte NEXT d?Ivoire) OR Micronesia OR Swaziland OR Djibouti OR Moldova OR “Syrian Arab Republic” OR Syria OR Egypt OR Mongolia OR Timor?Leste OR “El Salvador” OR Morocco OR Ukraine OR Georgia OR Nicaragua OR Uzbekistan OR Ghana OR Nigeria OR Vanuatu OR Guatemala OR Pakistan OR Vietnam OR Guyana OR “Papua New Guinea” OR “West Bank and Gaza” OR Gaza OR Honduras OR Paraguay OR Yemen OR Indonesia OR Philippines OR Zambia OR India OR Samoa OR Angola OR Fiji OR Palau OR Albania OR Gabon OR Panama OR Algeria OR Grenada OR Peru OR “American Samoa” OR Hungary OR Romania OR Argentina OR Iran OR Serbia OR Azerbaijan OR Iraq OR Seychelles OR Belarus OR Jamaica OR “South Africa” OR Belize OR Jordan OR “St. Lucia” OR “Bosnia and Herzegovina” OR Bosnia OR Kazakhstan OR “St. Vincent and the Grenadines” OR Botswana OR Lebanon OR Suriname OR Brazil OR Libya OR Thailand OR Bulgaria OR Macedonia OR Tonga OR China OR Malaysia OR Tunisia OR Colombia OR Maldives OR Turkey OR “Costa Rica” OR “Marshall Islands” OR Turkmenistan OR Cuba OR Mauritius OR Tuvalu OR Dominica OR Mexico OR Venezuela OR “Dominican Republic” OR Montenegro OR Ecuador OR Namibia)):ti,ab,kw  #1 AND #2 AND #3 |
