# Supplementary material for: Systematic review on the compliance of WHO guidelines in the management of patients with advanced HIV disease in Africa: The case of cryptococcal antigen screening
Source: PLoS One. 2025 Jan 24;20(1):e0313453. doi: 10.1371/journal.pone.0313453 (PMC11761098; doi:10.1371/journal.pone.0313453)
Supplement: S3 Table — (DOCX) [file pone.0313453.s003.docx]

| **S3 Table. Data collection form** | | | | | | | | | | | | | | | | | |
| --- | --- | --- | --- | --- | --- | --- | --- | --- | --- | --- | --- | --- | --- | --- | --- | --- | --- |
| First author’s name | Publication year | Country | Study year | ART status at the time of screening | CrAg criteria CD4 threshold | # of people eligible for CrAg screening | # of people screened for CrAg | # of people not screened for CrAg | # of people positive for CrAg | # of people negative for CrAg | # of people positive for CrAg and initiated treatment | # of people positive for CrAg | # of people positive for CrAg who had lumbar puncture | # of people with cryptococcal meningitis | Study site | CrAg assessments | Confirmation of eligibility |
| Baluku | 2021 | Uganda | 2018-2019 | Mixed | <100 | 8147 | 1721 | 6426 | 210 | 1511 |  |  |  |  | Multiple | Provider initiated | Eligible |
| Enock | 2022 | Uganda | 2016-2017 | ART naïve | ≤100 | 359 | 255 | 104 | 56 | 199 | 47 | 56 | 39 |  | Multiple | Provider initiated | Eligible |
| Tiam | 2023 | Lesotho | 2018-2019 | ART naïve | <200 | 109 | 39 | 70 | 5 | 34 |  | 5 | 5 | 4 | Multiple | Provider initiated | Eligible |
| Blankley | 2019 | Zimbabwe | 2015-2016 | ART naïve | <100 | 377 | 313 | 64 | 25 | 288 |  | 25 | 18 | 8 | Single | Provider initiated | Eligible |
| Vallabhaneni | 2016 | South Africa | 2012-2013 | ART naïve | <100 | 4395 | 1170 | 3225 | 24 | 1146 | 13 |  |  |  | Multiple | Provider initiated | Eligible |
| Drain | 2021 | South Africa | 2013-2019 | ART naïve | ≤200 | 908 | 301 | 607 | 28 | 273 | 28 |  |  |  | Single | Lab/provider | Eligible |
| Heller | 2022 | Malawi | 2017-2020 | Mixed | <200 | 475 | 423 | 52 | 59 | 364 |  | 37 | 24 | 15 | Single | Provider initiated | Eligible |
| Sing’oei | 2017 | Multiple countries | 2013-2017 | Mixed | <200 | 494 | 397 | 97 | 15 | 382 |  |  |  |  | Multiple | Provider initiated | Eligible |
| Kanyama | 2022 | Malawi | 2016-1017 | Mixed | ≤200 | 221 | 179 | 42 | 19 | 160 |  | 23 | 23 | 13 | Single | Provider initiated | Eligible |
| Hurt | 2021 | Botswana | 2014-2016 | Mixed | ≤100 | 3335 | 1645 | 1690 | 104 | 1541 |  | 72 | 20 |  | Multiple | Lab reflex | Eligible |
| Multiple countries included Kenya, Tanzania, Uganda and Nigeria | | | | | | | | | | | | | | | | | |

| **Data abstractors** | **Dates data was abstracted** |
| --- | --- |
| Zuhura Mbwana Ally | 21st Jan - 17th Feb, 2024 |
| Habib Ramadhani Omari | 21st Jan - 17th Feb, 2024 |
| Zarin Nudar Rodoshi | 15th Jan - 13th Feb, 2024 |
| Biruk D. Ayalew | 15th Jan - 13th Feb, 2024 |
