# Supplementary material for: Systematic review on the compliance of WHO guidelines in the management of patients with advanced HIV disease in Africa: The case of cryptococcal antigen screening
Source: PLoS One. 2025 Jan 24;20(1):e0313453. doi: 10.1371/journal.pone.0313453 (PMC11761098; doi:10.1371/journal.pone.0313453)
Supplement: S4 Table — (DOCX) [file pone.0313453.s004.docx]

| **S4 Table: Summary of all studies identified through literature search.** | | | | | |
| --- | --- | --- | --- | --- | --- |
| Study number | Title | Authors | Published Year | Status | Comments |
| 1 | Laboratory Reflex and Clinic-Based Point-of-Care Cryptococcal Antigen Screening for Preventing Meningitis and Mortality Among People Living With HIV. | Drain PK; Galagan SR; Govere S; Krows M; Thulare H; Wallis CL; Gosnell BI; Moosa MY; Celum C; Bassett IV | 2021 | Included | Eligible |
| 2 | Prevalence and Sequelae of Cryptococcal Antigenemia in Antiretroviral Therapy-Experienced Populations: An Evaluation of Reflex Cryptococcal Antigen Screening in Botswana. | Hurt WJ; Tenforde MW; Molefi M; Mitchell HK; Milton T; Azama MS; Goercke I; Mulenga F; Tlhako N; Tsholo K; Srivastava T; Leeme TB; Simoonga G; Muthoga C; Lechiile K; Mine M; Jarvis JN | 2021 | Included | Eligible |
| 3 | Evaluation of the initial 12â€‰months of a routine cryptococcal antigen screening program in reduction of HIV-associated cryptococcal meningitis in Uganda. | Enock K; Julius K; Griffith BC; Abila DB; Rutakingirwa MK; Kasibante J; Kandole KT; Kwizera R; Semeere A; Meya DB | 2022 | Included | Eligible |
| 4 | Implementation of tuberculosis and cryptococcal meningitis rapid diagnostic tests amongst patients with advanced HIV at Kamuzu Central Hospital, Malawi, 2016-2017. | Kanyama C; Chagomerana MB; Chawinga C; Ngoma J; Shumba I; Kumwenda W; Armando B; Kumwenda T; Kumwenda E; Hosseinipour MC | 2022 | Included | Eligible |
| 5 | The impact of routine cryptococcal antigen screening on survival among HIV-infected individuals with advanced immunosuppression in Kenya. | Meyer AC; Kendi CK; Penner JA; Odhiambo N; Otieno B; Omondi E; Opiyo E; Bukusi EA; Cohen CR | 2013 | Excluded | Evaluation was done before WHO guidelines were implemented |
| 6 | Implementation and Operational Research: Evaluation of a Public-Sector, Provider-Initiated Cryptococcal Antigen Screening and Treatment Program, Western Cape, South Africa. | Vallabhaneni S; Longley N; Smith M; Smith R; Osler M; Kelly N; Cross A; Boulle A; Meintjes G; Govender NP | 2016 | Included | Eligible |
| 7 | Rapid antiretroviral therapy initiation in patients with advanced HIV disease: 6-month outcomes of an observational cohort evaluation in Lesotho. | Tiam A; Paulin H; Machekano R; Oboho I; Agyemang E; Mugyenyi FA; Maama-Maime L; Mengistu Y; Chatora T; Mungati M; Mokone M; Mots'oane T; Masheane A; Tukei V | 2023 | Included | Eligible |
| 8 | Advanced HIV disease management practices within inpatient medicine units at a referral hospital in Zambia: a retrospective chart review. | Mbewe N; Vinikoor MJ; Fwoloshi S; Mwitumwa M; Lakhi S; Sivile S; Yavatkar M; Lindsay B; Stafford K; Hachaambwa L; Mulenga L; Claassen CW | 2022 | Excluded | Country did not adopt CrAg screening policy at the time the study was conducted |
| 9 | Screening for Cryptococcal Antigenemia and Burden of Cryptococcosis at the Time of HIV Diagnosis: A Retrospective Multicenter Study | Huang | 2021 | Excluded | Evaluation was done before WHO guidelines were implemented |
| 10 | Cryptococcal Antigen Screening Among Antiretroviral Therapy-Experienced People With HIV With Viral Load Nonsuppression in Rural Uganda. | Baluku JB; Mugabe P; Mwebaza S; Nakaweesi J; Senyimba C; Opio JP; Mukasa B | 2021 | Included | Eligible |
| 11 | Point-of-care cryptococcal antigen screening for preventing meningitis and mortality | National registry | 2017 | Excluded | Only percent provided but not numerator/denominator |
| 12 | Cryptococcal antigenemia in HIV-infected adults in the African cohort study | Sing'oei, V.; Owuoth, J.; Ganesan, K.; Andagalu, B.; Amusu, S.; Bahemana, E.; Kiweewa, F.; Maswai, J.; Ake, J.; Esber, A.; Crowell, T.A.; Polyak, C. | 2017 | Included | Eligible |
| 13 | Determinants of cryptococcal antigen (CrAg) screening uptake in Kampala, Uganda: An assessment of health center characteristics | Huang SH,Lee CY , Tsai CS, Tsai MS , Liu CE, Hsu WT, et al. | 2022 | Excluded | Only percent provided but not numerator/denominator |
| 14 | Lessons learned: Retrospective assessment of outcomes and management of patients with advanced HIV disease in a semi-urban polyclinic in Epworth, Zimbabwe | Blankley S, Gashu T, Ahmad B, Belaye AK, Ringtho L, Mesic A, et al. | 2019 | Included | Eligible |
| 15 | Implementing Advanced HIV Disease Care for Inpatients in a Referral Hospital in Malawi - Demand, Results and Cost Implications | Heller T, Damba D, Kumwenda T, Huwa J, Kamamia C, Nhlema A, et al. | 2022 | Included | Eligible |
| 16 | Global burden of disease of HIV-associated cryptococcal meningitis: an updated analysis. | Rajasingham R; Smith RM; Park BJ; Jarvis JN; Govender NP; Chiller TM; Denning DW; Loyse A; Boulware DR | 2017 | Excluded | Irrelevant, did not discuss the subject matter |
| 17 | Treatment for HIV-associated cryptococcal meningitis. | Tenforde MW; Shapiro AE; Rouse B; Jarvis JN; Li T; Eshun-Wilson I; Ford N | 2018 | Excluded | Irrelevant, did not discuss the subject matter |
| 18 | Epidemiology of Cryptococcus and cryptococcosis in China. | Fang W; Fa Z; Liao W | 2015 | Excluded | Irrelevant, did not discuss the subject matter |
| 19 | Epidemiology of Cryptococcus and cryptococcosis in Western Africa. | Akaihe CL; Nweze EI | 2021 | Excluded | Irrelevant, did not discuss the subject matter |
| 20 | Genetic diversity and microevolution in clinical Cryptococcus isolates from Cameroon. | Sephton-Clark P; Temfack E; Tenor JL; Toffaletti DL; Loyse A; Molloy SF; Perfect JR; Bicanic T; Harrison TS; Lortholary O; Kouanfack C; Cuomo CA | 2023 | Excluded | Irrelevant, did not discuss the subject matter |
| 21 | Tuberculosis/cryptococcosis co-infection in China between 1965 and 2016. | Fang W; Zhang L; Liu J; Denning DW; Hagen F; Jiang W; Hong N; Deng S; Lei X; Deng D; Liao W; Xu J; Boekhout T; Chen M; Pan W | 2017 | Excluded | Irrelevant, did not discuss the subject matter |
| 22 | Cryptococcosis in the Democratic Republic of Congo from 1953 to 2021: A systematic review and meta-analysis. | Zono BB; Kasumba DM; Situakibanza Nani-Tuma H; Bepouka Izizag B; Yambayamba Kapenga M; Nsuka Yanga R; Tshimanga Yona T; Kamangu Ntambwe E; Hayette MP; Mvumbi Lelo G | 2022 | Excluded | Irrelevant, did not discuss the subject matter |
| 23 | Treatment of cryptococcal meningitis in resource limited settings. | Sloan DJ; Dedicoat MJ; Lalloo DG | 2009 | Excluded | Irrelevant, did not discuss the subject matter |
| 24 | Asymptomatic cryptococcal antigenemia in HIV-infected patients: a review of recent studies. | Xu XL; Zhao T; Harypursat V; Lu YQ; Li Y; Chen YK | 2020 | Excluded | Irrelevant, did not discuss the subject matter |
| 25 | Strategies for the diagnosis and management of meningitis in HIV-infected adults in resource limited settings. | Bremer M; Kadernani YE; Wasserman S; Wilkinson RJ; Davis AG | 2021 | Excluded | Irrelevant, did not discuss the subject matter |
| 26 | Integrating cryptococcal antigen screening and pre-emptive treatment into routine HIV care. | Rajasingham R; Meya DB; Boulware DR | 2012 | Excluded | Irrelevant, did not discuss the subject matter |
| 27 | Cryptococcal meningitis: improving access to essential antifungal medicines in resource-poor countries. | Loyse A; Thangaraj H; Easterbrook P; Ford N; Roy M; Chiller T; Govender N; Harrison TS; Bicanic T | 2013 | Excluded | Irrelevant, did not discuss the subject matter |
| 28 | Cryptococcal meningitis in people living with human immunodeficiency virus in Nepal: Perspectives from resource limited setting. | Sharma S; Acharya J; Rijal N; Chalise BS; Bhandari P; Banjara MR; Ghimire P; Singh A | 2023 | Excluded | Irrelevant, did not discuss the subject matter |
| 29 | Early versus delayed antiretroviral treatment in HIV-positive people with cryptococcal meningitis. | Eshun-Wilson I; Okwen MP; Richardson M; Bicanic T | 2018 | Excluded | Irrelevant, did not discuss the subject matter |
| 30 | Cryptococcal meningitis in non-HIV patients in the State of Amazonas, Northern Brazil. | Pinheiro SB; Sousa ES; Cortez ACA; da Silva Rocha DF; Menescal LSF; Chagas VS; GÃ³mez ASP; Cruz KS; Santos LO; Alves MJ; Matsuura ABJ; Wanke B; Trilles L; Frickmann H; de Souza JVB | 2021 | Excluded | Irrelevant, did not discuss the subject matter |
| 31 | Cryptococcal Antigenemia in Human Immunodeficiency Virus Antiretroviral Therapy-Experienced Ugandans With Virologic Failure. | Mpoza E; Rajasingham R; Tugume L; Rhein J; Nabaggala MS; Ssewanyana I; Nyegenye W; Kushemererwa GE; Mulema V; Kalamya J; Kiyaga C; Kabanda J; Ssali M; Boulware DR; Meya DB | 2020 | Excluded | Irrelevant, did not discuss the subject matter |
| 32 | Cryptococcal antigenemia is associated with meningitis or death in HIV-infected adults with CD4 100-200 cells/mm(3). | Wykowski J; Galagan SR; Govere S; Wallis CL; Moosa MY; Celum C; Drain PK | 2020 | Excluded | Irrelevant, did not discuss the subject matter |
| 33 | A case of HIV negative cryptococcal meningitis with antiphospholipid syndrome. | Zhao J; Wu X; Huang Z; Zhang J | 2021 | Excluded | Irrelevant, did not discuss the subject matter |
| 34 | Outcomes of HIV-positive patients with cryptococcal meningitis in the Americas. | Crabtree RamÃ­rez B; Caro Vega Y; Shepherd BE; Le C; Turner M; Frola C; Grinsztejn B; Cortes C; Padgett D; Sterling TR; McGowan CC; Person A | 2017 | Excluded | Irrelevant, did not discuss the subject matter |
| 35 | Cost-effectiveness of single, high-dose, liposomal amphotericin regimen for HIV-associated cryptococcal meningitis in five countries in sub-Saharan Africa: an economic analysis of the AMBITION-cm trial. | Lawrence DS; Muthoga C; Meya DB; Tugume L; Williams D; Rajasingham R; Boulware DR; Mwandumba HC; Moyo M; Dziwani EN; Maheswaran H; Kanyama C; Hosseinipour MC; Chawinga C; Meintjes G; Schutz C; Comins K; Bango F; Muzoora C; Jjunju S; Nuwagira E; Mosepele M; Leeme T; Ndhlovu CE; Hlupeni A; Shamu S; Boyer-Chammard T; Molloy SF; Youssouf N; Chen T; Shiri T; Jaffar S; Harrison TS; Jarvis JN; Niessen LW | 2022 | Excluded | Irrelevant, did not discuss the subject matter |
| 36 | Cost-effectiveness of CRAG-LFA screening for cryptococcal meningitis among people living with HIV in Uganda. | Ramachandran A; Manabe Y; Rajasingham R; Shah M | 2017 | Excluded | Irrelevant, did not discuss the subject matter |
| 37 | Cryptococcal disease and the burden of other fungal diseases in Uganda; Where are the knowledge gaps and how can we fill them? | Parkes-Ratanshi R; Achan B; Kwizera R; Kambugu A; Meya D; Denning DW | 2015 | Excluded | Irrelevant, did not discuss the subject matter |
| 38 | Multicenter Cryptococcal Antigen Screening of HIV-Infected Patients in Iran. | Bandalizadeh Z; Shokohi T; Moosazadeh M; Keikha N; Seyedpor H; Rabie Rudsari M; Babamahmoudi F; Ghasemian R; Mardani M; Javanian M; Soleimanpour S; Sefidgar AA; Shokri M; Gouya MM; Seyedmousavi S | 2020 | Excluded | Irrelevant, did not discuss the subject matter |
| 39 | Management of cryptococcal meningitis in sub-saharan Africa. | Jackson A; Hosseinipour MC | 2010 | Excluded | Irrelevant, did not discuss the subject matter |
| 40 | Screening for cryptococcal antigen in asymptomatic people with HIV: urgent need in Eastern India. | Chakravarty J; Reddy S; Gupta MK; Tilak R; Diwaker C; Sundar S | 2023 | Excluded | Irrelevant, did not discuss the subject matter |
| 41 | Prevalence of Cryptococcus gattii in Ugandan HIV-infected patients presenting with cryptococcal meningitis. | Wembabazi A; Nassozi DR; Akot E; Ochola TI; Kweka PT; Katamu NT; Meya D; Achan B | 2022 | Excluded | Irrelevant, did not discuss the subject matter |
| 42 | Clinical presentation and outcomes of syphilitic meningitis among hospitalized adults in lusaka, Zambia. | Chomba M; Siddiqi OK; Fwoloshi S; Mubanga E; Koralnik IJ; Marra C; Saylor D | 2022 | Excluded | Irrelevant, did not discuss the subject matter |
| 43 | High prevalence of cryptococcal antigenaemia amongst asymptomatic advanced HIV patients in Pune, India. | Kadam D; Chandanwale A; Bharadwaj R; Nevrekar N; Joshi S; Patil S; Gupte N; Sangle S; Chopade K; Kulkarni V; Balasubramanian U; Suryavanshi N; Jain D; Kanade S; Dharmashale S; Kagal A; Gupta A; Mave V | 2017 | Excluded | Irrelevant, did not discuss the subject matter |
| 44 | Seasonality and meteorological factors of HIV-negative cryptococcal meningitis in Guangdong Province, China. | Dai K; Feng Z; Hu T; Su Z; Yuan D; Qin BE; Gu M; Peng F; Jiang Y | 2023 | Excluded | Irrelevant, did not discuss the subject matter |
| 45 | Cryptococcal antigen screening and early antifungal treatment to prevent cryptococcal meningitis: a review of the literature. | Kaplan JE; Vallabhaneni S; Smith RM; Chideya-Chihota S; Chehab J; Park B | 2015 | Excluded | Irrelevant, did not discuss the subject matter |
| 46 | Cryptococcosis in HIV-AIDS patients from Southern Brazil: Still a major problem. | da Silva LB; Bock D; Klafke GB; Sanchotene KO; Basso RP; Benelli JL; Poester VR; da Silva FA; Trilles L; Severo CB; Stevens DA; Xavier MO | 2020 | Excluded | Irrelevant, did not discuss the subject matter |
| 47 | Outcomes of Reflex Cryptococcal Antigen (CrAg) Screening in Human Immunodeficiency Virus (HIV)-Positive Patients With CD4 Counts of 100-200 Cells/ÂµL in Botswana. | Tenforde MW; Milton T; Rulaganyang I; Muthoga C; Tawe L; Chiller T; Greene G; Jordan A; Williams CG; Owen L; Leeme TB; Boose A; Ngidi J; Mine M; Jarvis JN | 2021 | Excluded | Irrelevant, did not discuss the subject matter |
| 48 | Impact of prior cryptococcal antigen screening on in-hospital mortality in cryptococcal meningitis or fungaemia among HIV-seropositive individuals in South Africa: a cross-sectional observational study. | Paccoud O; Shuping L; Mashau R; Greene G; Quan V; Meiring S; Govender NP | 2023 | Excluded | Irrelevant, did not discuss the subject matter |
| 49 | Anti-GM-CSF Neutralizing Autoantibodies in Colombian Patients with Disseminated Cryptococcosis. | Arango-Franco CA; Migaud M; RamÃ­rez-SÃ¡nchez IC; Arango-Bustamante K; Moncada-VÃ©lez M; Rojas J; Gervais A; PatiÃ±o-Giraldo S; Perez-Zapata LJ; Ãlvarez Ãlvarez JA; Orrego JC; Roncancio-Villamil G; Boisson-Dupuis S; Jouanguy E; Abel L; Casanova JL; Bustamante J; Arias AA; Franco JL; Puel A | 2023 | Excluded | Irrelevant, did not discuss the subject matter |
| 50 | Comparative analysis of diagnostic methods for the detection of Cryptococcus neoformans meningitis. | Dantas KC; de Freitas-Xavier RS; Spina Lombardi SCF; JÃºnior AM; da Silva MV; Criado PR; de Freitas VLT; de Almeida TMB | 2023 | Excluded | Irrelevant, did not discuss the subject matter |
| 51 | Laboratory-Reflex Cryptococcal Antigen Screening Is Associated With a Survival Benefit in Tanzania. | Faini D; Kalinjuma AV; Katende A; Mbwaji G; Mnzava D; Nyuri A; Glass TR; Furrer H; Hatz C; Boulware DR; Letang E | 2019 | Excluded | Irrelevant, did not discuss the subject matter |
| 52 | HIV-Associated Cryptococcal Meningitis Occurring at Relatively Higher CD4 Counts. | Tugume L; Rhein J; Hullsiek KH; Mpoza E; Kiggundu R; Ssebambulidde K; Schutz C; Taseera K; Williams DA; Abassi M; Muzoora C; Musubire AK; Meintjes G; Meya DB; Boulware DR | 2019 | Excluded | Irrelevant, did not discuss the subject matter |
| 53 | Cryptococcal meningitis: clinical, diagnostic and therapeutic overviews. | Satishchandra P; Mathew T; Gadre G; Nagarathna S; Chandramukhi A; Mahadevan A; Shankar SK | 2007 | Excluded | Irrelevant, did not discuss the subject matter |
| 54 | Tackling cryptococcal meningitis in Nigeria, one-step at a time; the impact of training. | Oladele RO; Jordan A; Akande P; Akanmu SA; Akase IE; Aliyu S; Denning DW; Chiller T | 2020 | Excluded | Irrelevant, did not discuss the subject matter |
| 55 | Delays in Cryptococcal Meningitis Diagnosis and Care: A Mixed Methods Study in Rural Uganda. | Link A; Okwir M; Nabongo B; Meya D; Iribarren S; Bohjanen P; Kasprzyk D | 2022 | Excluded | Irrelevant, did not discuss the subject matter |
| 56 | Cryptococcal Meningitis in a Mexican Neurological Center. | CÃ¡rdenas G; Vargas-GarcÃ­a LF; Adames-Espinal H; Soto-HernÃ¡ndez JL; CastaÃ±Ã³n-Olivares LR | 2023 | Excluded | Irrelevant, did not discuss the subject matter |
| 57 | Cost-effectiveness analysis and budgetary impact of the Cryptococcal Antigen Lateral Flow Assay (CRAG-LFA) implementation for the screening and diagnosis of cryptococcosis in asymptomatic people living with HIV in Brazil. | Vianna CMM; Mosegui GBG | 2021 | Excluded | Irrelevant, did not discuss the subject matter |
| 58 | Evaluation of the BioFireÂ® FilmArrayÂ® Meningitis/Encephalitis panel in an adult and pediatric Ugandan population. | Bridge S; Hullsiek KH; Nerima C; Evans EE; Nuwagira E; Stadelman AM; Tran T; Kim G; Tadeo KK; Kwizera R; Mwesigye J; Ellis J; Cresswell FV; Meya DB; Muzoora C; Boulware DR; Rhein J | 2021 | Excluded | Irrelevant, did not discuss the subject matter |
| 59 | Cryptococcosis in Colombian children and literature review. | Lizarazo J; EscandÃ³n P; Agudelo CI; CastaÃ±eda E | 2014 | Excluded | Irrelevant, did not discuss the subject matter |
| 60 | Cryptococcal-related Mortality Despite Fluconazole Preemptive Treatment in a Cryptococcal Antigen Screen-and-Treat Program. | Wake RM; Govender NP; Omar T; Nel C; Mazanderani AH; Karat AS; Ismail NA; Tiemessen CT; Jarvis JN; Harrison TS | 2020 | Excluded | Irrelevant, did not discuss the subject matter |
| 61 | Clinical and Epidemiological Characteristics of Neurocryptococcosis Associated with HIV in Northeastern Brazil. | Oliveira EP; de Sousa BR; de Freitas JF; Neves RP; JucÃ¡ MB; de AraÃºjo PSR; da Costa Lima JL; Maciel MAV; de Lima-Neto RG | 2023 | Excluded | Irrelevant, did not discuss the subject matter |
| 62 | Cryptococcus Neoformans Meningitis Cases Among China's HIV-Infected Population may have been Severely Under-Reported. | Chen M; Xu N; Xu J | 2020 | Excluded | Irrelevant, did not discuss the subject matter |
| 63 | Cryptococcus neoformans seropositivity and some haematological parameters in HIV seropositive subjects. | Chukwuanukwu RC; Uchenna N; Mbagwu SI; Chukwuanukwu TO; Charles O | 2020 | Excluded | Irrelevant, did not discuss the subject matter |
| 64 | The clinical profiles and outcomes of HIV-negative cryptococcal meningitis patients in type II diabetes mellitus. | Li H; Li X; Zhang L; Fang W; Zhang K; Arastehfar A; Ilkit M; Hu D; Chen X; Wang H; Ling L; Lin J; Xu B; Liao W; Pan W; Zhang Q | 2021 | Excluded | Irrelevant, did not discuss the subject matter |
| 65 | Adjunctive Dexamethasone in HIV-Associated Cryptococcal Meningitis. | Beardsley J; Wolbers M; Kibengo FM; Ggayi AB; Kamali A; Cuc NT; Binh TQ; Chau NV; Farrar J; Merson L; Phuong L; Thwaites G; Van Kinh N; Thuy PT; Chierakul W; Siriboon S; Thiansukhon E; Onsanit S; Supphamongkholchaikul W; Chan AK; Heyderman R; Mwinjiwa E; van Oosterhout JJ; Imran D; Basri H; Mayxay M; Dance D; Phimmasone P; Rattanavong S; Lalloo DG; Day JN | 2016 | Excluded | Irrelevant, did not discuss the subject matter |
| 66 | Symptomatic Cryptococcal Antigenemia Presenting as Early Cryptococcal Meningitis With Negative Cerebral Spinal Fluid Analysis. | Ssebambulidde K; Bangdiwala AS; Kwizera R; Kandole TK; Tugume L; Kiggundu R; Mpoza E; Nuwagira E; Williams DA; Lofgren SM; Abassi M; Musubire AK; Cresswell FV; Rhein J; Muzoora C; Hullsiek KH; Boulware DR; Meya DB | 2019 | Excluded | Irrelevant, did not discuss the subject matter |
| 67 | Performance of Metagenomic Next-Generation Sequencing for the Diagnosis of Cryptococcal Meningitis in HIV-Negative Patients. | Gan Z; Liu J; Wang Y; Yang L; Lou Z; Xia H; Li M; Chen Z; Jiang Y; Peng F | 2022 | Excluded | Irrelevant, did not discuss the subject matter |
| 68 | Burden of serious fungal infections in the Dominican Republic. | Gugnani HC; Denning DW | 2016 | Excluded | Irrelevant, did not discuss the subject matter |
| 69 | Estimation of the current global burden of cryptococcal meningitis among persons living with HIV/AIDS. | Park BJ; Wannemuehler KA; Marston BJ; Govender N; Pappas PG; Chiller TM | 2009 | Excluded | Irrelevant, did not discuss the subject matter |
| 70 | Sporadic occurrence of cryptococcal meningitis in HIV-seronegative patients: Uncommon etiology? | Das S; Datt S; Roy P; Saha R; Xess I | 2017 | Excluded | Irrelevant, did not discuss the subject matter |
| 71 | Impact of biological sex on cryptococcal meningitis mortality in Uganda and South Africa. | Stadelman AM; Ssebambulidde K; Tugume L; Pastick KA; Hullsiek KH; Lofgren S; Nuwagira E; Evans EE; Williams DA; Muzoora C; Meya DB; Rajasingham R; Rhein J; Boulware DR | 2021 | Excluded | Irrelevant, did not discuss the subject matter |
| 72 | Strategies to reduce early morbidity and mortality in adults receiving antiretroviral therapy in resource-limited settings. | Lawn SD; Harries AD; Wood R | 2010 | Excluded | Irrelevant, did not discuss the subject matter |
| 73 | Reduction in mortality from HIV-related CNS infections in routine care in Africa (DREAMM): a before-and-after, implementation study. | Mfinanga S; Kanyama C; Kouanfack C; Nyirenda S; Kivuyo SL; Boyer-Chammard T; Phiri S; Ngoma J; Shimwela M; Nkungu D; Fomete LN; Simbauranga R; Chawinga C; Ngakam N; Heller T; Lontsi SS; Aghakishiyeva E; Jalava K; Fuller S; Reid AM; Rajasingham R; Lawrence DS; Hosseinipour MC; Beaumont E; Bradley J; Jaffar S; Lortholary O; Harrison T; Molloy SF; Sturny-LeclÃ¨re A; Loyse A | 2023 | Excluded | Irrelevant, did not discuss the subject matter |
| 74 | High burden of cryptococcal antigenemia and meningitis among patients presenting at an emergency department in Maputo, Mozambique. | Deiss R; Loreti CV; Gutierrez AG; Filipe E; Tatia M; Issufo S; Ciglenecki I; Loarec A; Vivaldo H; Barra C; Siufi C; Molfino L; Tamayo Antabak N | 2021 | Excluded | Irrelevant, did not discuss the subject matter |
| 75 | Magnitude of Cryptococcal Antigenemia among HIV Infected Patients at a Referral Hospital, Northwest Ethiopia. | Derbie A; Ayalew W; Mekonnen D; Alemu M; Mulugeta Y | 2018 | Excluded | Irrelevant, did not discuss the subject matter |
| 76 | Cryptococcal meningitis epidemiology: 17 years of experience in a State of the Brazilian Pantanal. | Nunes JO; Tsujisaki RAS; Nunes MO; Lima GME; Paniago AMM; Pontes ERJC; Chang MR | 2018 | Excluded | Irrelevant, did not discuss the subject matter |
| 77 | Asymptomatic Cryptococcal Antigenemia in People Living with HIV (PLHIV) with Severe Immunosuppression: Is Routine CrAg Screening Indicated in India? | Anuradha S; H AN; Dewan R; Kaur R; Rajeshwari K | 2017 | Excluded | Irrelevant, did not discuss the subject matter |
| 78 | Implementation of rapid diagnostics assays for detection of histoplasmosis and cryptococcosis in central american people living with HIV. | Caceres DH; Arauz AB; Flores C; Santiago E; Montoya S; Saenz C; Torres-Meneses FA; Peralta H; Zuniga-Moya JC; Lainez Arteaga IZ; Garcia A; Abdo J; Verweij PE; Chiller TM; Forno D | 2021 | Excluded | Irrelevant, did not discuss the subject matter |
| 79 | Inadequacy of High-Dose Fluconazole Monotherapy Among Cerebrospinal Fluid Cryptococcal Antigen (CrAg)-Positive Human Immunodeficiency Virus-Infected Persons in an Ethiopian CrAg Screening Program. | Beyene T; Zewde AG; Balcha A; Hirpo B; Yitbarik T; Gebissa T; Rajasingham R; Boulware DR | 2017 | Excluded | Irrelevant, did not discuss the subject matter |
| 80 | Cryptococcosis in HIV/AIDS patients in northern Brazil: Clinical aspects, molecular types and isolation of agents from environmental samples associated with patients. | Alves MJ; do Nascimento IS; Cruz KS; Menescal VVF; Menescal LSF; Silva LSC; Pinheiro SB; GÃ³mez ASP; de Souza JVB; Lazera MDS; Jackisch-Matsuura AB | 2022 | Excluded | Irrelevant, did not discuss the subject matter |
| 81 | Cryptococcal Antigen Screening in Patients Initiating ART in South Africa: A Prospective Cohort Study. | Longley N; Jarvis JN; Meintjes G; Boulle A; Cross A; Kelly N; Govender NP; Bekker LG; Wood R; Harrison TS | 2016 | Excluded | Irrelevant, did not discuss the subject matter |
| 82 | Surgical treatment and operation time in human immunodeficiency virus-negative cryptococcal meningitis. | Zhao J; Zhao X; Yang S; Miao S; Liu Y | 2020 | Excluded | Irrelevant, did not discuss the subject matter |
| 83 | Cryptococcosis in pregnancy and the postpartum period: Case series and systematic review with recommendations for management. | Pastick KA; Nalintya E; Tugume L; Ssebambulidde K; Stephens N; Evans EE; Ndyetukira JF; Nuwagira E; Skipper C; Muzoora C; Meya DB; Rhein J; Boulware DR; Rajasingham R | 2020 | Excluded | Irrelevant, did not discuss the subject matter |
| 84 | Estimating the burden of fungal disease in Vietnam. | Beardsley J; Denning DW; Chau NV; Yen NT; Crump JA; Day JN | 2015 | Excluded | Irrelevant, did not discuss the subject matter |
| 85 | Leave no one behind: response to new evidence and guidelines for the management of cryptococcal meningitis in low-income and middle-income countries. | Loyse A; Burry J; Cohn J; Ford N; Chiller T; Ribeiro I; Koulla-Shiro S; Mghamba J; Ramadhani A; Nyirenda R; Aliyu SH; Wilson D; Le T; Oladele R; Lesikari S; Muzoora C; Kalata N; Temfack E; Mapoure Y; Sini V; Chanda D; Shimwela M; Lakhi S; Ngoma J; Gondwe-Chunda L; Perfect C; Shroufi A; Andrieux-Meyer I; Chan A; Schutz C; Hosseinipour M; Van der Horst C; Klausner JD; Boulware DR; Heyderman R; Lalloo D; Day J; Jarvis JN; Rodrigues M; Jaffar S; Denning D; Migone C; Doherty M; Lortholary O; Dromer F; Stack M; Molloy SF; Bicanic T; van Oosterhout J; Mwaba P; Kanyama C; Kouanfack C; Mfinanga S; Govender N; Harrison TS | 2019 | Excluded | Irrelevant, did not discuss the subject matter |
| 86 | Population pharmacokinetics and CSF penetration of flucytosine in adults with HIV-associated cryptococcal meningoencephalitis. | Stott KE; Ahmadu A; Kajanga C; Moyo M; Gondwe E; Chimang'anga W; Chasweka M; Unsworth J; Jimenez-Valverde A; Jagota B; Shah RV; Lawrence DS; Lalloo DG; Harrison T; Jarvis JN; Hope W; Mwandumba HC | 2023 | Excluded | Irrelevant, did not discuss the subject matter |
| 87 | Mortality due to Cryptococcus neoformans and Cryptococcus gattii in low-income settings: an autopsy study. | Hurtado JC; Castillo P; Fernandes F; Navarro M; Lovane L; Casas I; QuintÃ³ L; Marco F; Jordao D; Ismail MR; Lorenzoni C; Martinez-Palhares AE; Ferreira L; Lacerda M; Monteiro W; Sanz A; Letang E; Marimon L; Jesri S; Cossa A; Mandomando I; Vila J; Bassat Q; Ordi J; MenÃ©ndez C; Carrilho C; MartÃ­nez MJ | 2019 | Excluded | Irrelevant, did not discuss the subject matter |
| 88 | Treatment of acute cryptococcal meningitis in HIV infected adults, with an emphasis on resource-limited settings. | Sloan D; Dlamini S; Paul N; Dedicoat M | 2008 | Excluded | Irrelevant, did not discuss the subject matter |
| 89 | Prevalence of cryptococcal infection among advanced HIV patients in Argentina using lateral flow immunoassay. | Frola C; Guelfand L; Blugerman G; Szyld E; Kaufman S; Cahn P; Sued O; PÃ©rez H | 2017 | Excluded | Irrelevant, did not discuss the subject matter |
| 90 | Cryptococcal meningitis in HIV-negative patients with systemic connective tissue diseases. | Gonzalez-Duarte A; Saniger-Alba Mdel M; Higuera-Calleja J | 2015 | Excluded | Irrelevant, did not discuss the subject matter |
| 91 | Prospective cohort of AIDS patients screened for cryptococcal antigenaemia, pre-emptively treated and followed in Brazil. | Borges MASB; de AraÃºjo Filho JA; Oliveira BJS; Moreira IS; de Paula VV; de Bastos AL; Soares RBA; Turchi MD | 2019 | Excluded | Irrelevant, did not discuss the subject matter |
| 92 | Ambulatory induction phase treatment of cryptococcal meningitis in HIV integrated primary care clinics, Yangon, Myanmar. | Warrell CE; Macrae C; McLean ARD; Wilkins E; Ashley EA; Smithuis F; Tun NN | 2021 | Excluded | Irrelevant, did not discuss the subject matter |
| 93 | Management of cryptococcal meningitis in AIDS: the need for specific studies in developing countries. | Lortholary O | 2007 | Excluded | Irrelevant, did not discuss the subject matter |
| 94 | Adjunctive sertraline for HIV-associated cryptococcal meningitis: a randomised, placebo-controlled, double-blind phase 3 trial. | Rhein J; Huppler Hullsiek K; Tugume L; Nuwagira E; Mpoza E; Evans EE; Kiggundu R; Pastick KA; Ssebambulidde K; Akampurira A; Williams DA; Bangdiwala AS; Abassi M; Musubire AK; Nicol MR; Muzoora C; Meya DB; Boulware DR | 2019 | Excluded | Irrelevant, did not discuss the subject matter |
| 95 | [Expert consensus on the diagnosis and treatment of cryptococcal meningitis]. | Liu ZY; Wang GQ; Zhu LP; Lyu XJ; Zhang QQ; Yu YS; Zhou ZH; Liu YB; Cai WP; Li RY; Zhang WH; Zhang FJ; Wu H; Xu YC; Lu HZ; Li TS | 2018 | Excluded | Irrelevant, did not discuss the subject matter |
| 96 | Integrating clinical services for HIV, tuberculosis, and cryptococcal disease in the developing world: a step forward with 2 novel diagnostic tests. | Vijayan T; Klausner JD | 2013 | Excluded | Irrelevant, did not discuss the subject matter |
| 97 | Dynamic ploidy changes drive fluconazole resistance in human cryptococcal meningitis. | Stone NR; Rhodes J; Fisher MC; Mfinanga S; Kivuyo S; Rugemalila J; Segal ES; Needleman L; Molloy SF; Kwon-Chung J; Harrison TS; Hope W; Berman J; Bicanic T | 2019 | Excluded | Irrelevant, did not discuss the subject matter |
| 98 | A qualitative evaluation of an implementation study for cryptococcal antigen screening and treatment in Uganda. | Lofgren SM; Nalintya E; Meya DB; Boulware DR; Rajasingham R | 2018 | Excluded | Irrelevant, did not discuss the subject matter |
| 99 | Causes of Pediatric Meningitis in Botswana: Results From a 16-Year National Meningitis Audit. | Mitchell HK; Mokomane M; Leeme T; Tlhako N; Tsholo K; Ramodimoosi C; Dube B; Mokobela KO; Tawanana E; Chebani T; Setlhake P; Pilatwe T; Hurt WJ; Molefi M; Mullan PC; Steenhoff AP; Mine M; Jarvis JN; Tenforde MW | 2019 | Excluded | Irrelevant, did not discuss the subject matter |
| 100 | A clinical, aetiological, and public health perspective on central nervous system infections in Bolivia, 2017-2018. | Saba Villarroel PM; Castro Soto MDR; Melendres Flores O; Peralta LandÃ­var A; CalderÃ³n ME; Loayza R; Boucraut J; Thirion L; Dubot-PÃ©rÃ¨s A; Ninove L; de Lamballerie X | 2021 | Excluded | Irrelevant, did not discuss the subject matter |
| 101 | HIV Infection Does Not Increase 10-Week Mortality of Chinese Cryptococcal Meningitis Patients. | Xu L; Yu J; Zheng B; Su F; Yang Z; Guo Y; Tao R; Dai X; Huang Y; Shi J; Zhang Z; Chen Y; Zhu B | 2020 | Excluded | Irrelevant, did not discuss the subject matter |
| 102 | Blood neutrophil counts in HIV-infected patients with cryptococcal meningitis: Association with mortality. | Musubire AK; Meya DB; Rhein J; Meintjes G; Bohjanen PR; Nuwagira E; Muzoora C; Boulware DR; Hullsiek KH | 2018 | Excluded | Irrelevant, did not discuss the subject matter |
| 103 | Cryptococcal antigen carriage among HIV infected children aged 6 months to 15 years at Laquintinie Hospital in Douala. | Kalla GCM; Mboumnyemb JF; Assob JCN; Ehouzou Mandeng MN; Kamgaing Noubi N; Okomo Assoumou MC; Mbopi-Keou FX; Monebenimp F | 2021 | Excluded | Irrelevant, did not discuss the subject matter |
| 104 | Etiology of meningitis among adults in three quaternary hospitals in Mozambique, 2016-2017: The role of HIV. | Nhantumbo AA; ComÃ© CE; Maholela PI; Munguambe AM; Costa PD; Mott M; Cunha GR; Chambal L; Dias C; Cantarelli VV; Gudo ES | 2022 | Excluded | Irrelevant, did not discuss the subject matter |
| 105 | Adherence of health workers to guidelines for screening and management of cryptococcal meningitis in Uganda. | Namuju OC; Namuwenge PM; Kwizera R; Obuya E; Kirumira P; Naluyima R; Ahimbisibwe C; Ndyetukira J; Nakato H; Kirungi R; Gakuru J; Junju S; Nuwagira E; Rutakagirwa M; Nsibirwa S; Nabitaka V; Nalintya E; Mpoza E; Muzoora CK; Musubire AK; Boulware DR; Meya DB | 2023 | Excluded | Irrelevant, did not discuss the subject matter |
| 106 | Cryptococcosis among HIV negative liver disease patients: Epidemiology, underlying conditions, antifungal susceptibility profile from tertiary care hepatobiliary center. | Patel D; Khillan V; Patel N; Kale P | 2023 | Excluded | Irrelevant, did not discuss the subject matter |
| 107 | Survival following screening and preemptive antifungal therapy for subclinical cryptococcal disease in advanced HIV infection. | Makadzange TA; Hlupeni A; Machekano R; Boyd K; Mtisi T; Nyamayaro P; Ross C; Vallabhaneni S; Balachandra S; Chonzi P; Ndhlovu CE | 2021 | Excluded | Irrelevant, did not discuss the subject matter |
| 108 | Cryptococcal meningitis in HIV-infected patients: a longitudinal study in Cambodia. | EspiÃ© E; Pinoges L; Balkan S; Chanchhaya N; Molfino L; Narom P; Pujades-RodrÃ­guez M | 2010 | Excluded | Irrelevant, did not discuss the subject matter |
| 109 | Induction-phase treatment costs for cryptococcal meningitis in high HIV-burden African countries: New opportunities with lower costs. | Larson B; Shroufi A; Muthoga C; Oladele R; Rajasingham R; Jordan A; Jarvis JN; Chiller TM; Govender NP | 2021 | Excluded | Irrelevant, did not discuss the subject matter |
| 110 | Asymptomatic cryptococcal antigenemia is associated with mortality among HIV-positive patients in Indonesia. | Ganiem AR; Indrati AR; Wisaksana R; Meijerink H; van der Ven A; Alisjahbana B; van Crevel R | 2014 | Excluded | Irrelevant, did not discuss the subject matter |
| 111 | Prevalence of cryptococcal antigenemia and cost-effectiveness of a cryptococcal antigen screening program--Vietnam. | Smith RM; Nguyen TA; Ha HT; Thang PH; Thuy C; Lien TX; Bui HT; Le TH; Struminger B; McConnell MS; Fanfair RN; Park BJ; Harris JR | 2013 | Excluded | Irrelevant, did not discuss the subject matter |
| 112 | Clinical and microbiological features of cryptococcal meningitis. | Hasimoto e Souza LK; Costa CR; Fernandes Ode F; AbrÃ£o FY; Silva TC; TremÃ©a CM; Silva Mdo R | 2013 | Excluded | Irrelevant, did not discuss the subject matter |
| 113 | Cost-effectiveness of single-dose AmBisome pre-emptive treatment for the prevention of cryptococcal meningitis in African low and middle-income countries. | Rajasingham R; Nalintya E; Israelski DM; Meya DB; Larson BA; Boulware DR | 2022 | Excluded | Irrelevant, did not discuss the subject matter |
| 114 | Diagnosis of cryptococcal and tuberculous meningitis in a resource-limited African setting. | Cohen DB; Zijlstra EE; Mukaka M; Reiss M; Kamphambale S; Scholing M; Waitt PI; Neuhann F | 2010 | Excluded | Irrelevant, did not discuss the subject matter |
| 115 | Prevalence of Advanced HIV Disease, Cryptococcal Antigenemia, and Suboptimal Clinical Outcomes Among Those Enrolled in Care in Vietnam. | Dat VQ; Lyss S; Dung NTH; Hung LM; Pals SL; Anh HTV; Kinh NV; Bateganya M | 2021 | Excluded | Irrelevant, did not discuss the subject matter |
| 116 | New insights in the prevention, diagnosis, and treatment of cryptococcal meningitis. | Jackson A; van der Horst C | 2012 | Excluded | Irrelevant, did not discuss the subject matter |
| 117 | Cryptococcal Meningitis: A Rare Complication in HIV-Negative Patients with Nephrotic Syndrome in A Chinese Teaching Hospital. | Hu D; Zhang Q; Jiang W; Arastehfar A; Ilkit M; Fang W; Li H; Chen X; Zhang L; Li X; Pan W; Liao W | 2020 | Excluded | Irrelevant, did not discuss the subject matter |
| 118 | Diagnosis of fungal opportunistic infections in people living with HIV from Guatemala and El Salvador. | Forno D; Samayoa B; Medina N; Arathoon E; Mejia CR; Gordillo R; Cedillos R; Rodas J; Ahlquist Cleveland A; Chiller T; Caceres DH | 2021 | Excluded | Irrelevant, did not discuss the subject matter |
| 119 | Characteristics and outcomes of cryptococcal meningitis in HIV seronegative children in Beijing, China, 2002-2013. | Guo LY; Liu LL; Liu Y; Chen TM; Li SY; Yang YH; Liu G | 2016 | Excluded | Irrelevant, did not discuss the subject matter |
| 120 | Factors associated with early mycological clearance in HIV-associated cryptococcal meningitis. | Concha-Velasco F; GonzÃ¡lez-Lagos E; Seas C; Bustamante B | 2017 | Excluded | Irrelevant, did not discuss the subject matter |
| 121 | MORTALITY AFTER CLINICAL MANAGEMENT OF AIDS-ASSOCIATED CRYPTOCOCCAL MENINGITIS IN KENYA. | Baldassarre R; Mdodo R; Omonge E; Jaoko W; Baddley J; Pappas P; Aban I; Odera S; Suleh A; Jolly PE | 2014 | Excluded | Irrelevant, did not discuss the subject matter |
| 122 | Neurological manifestations of HIV-AIDS at a tertiary care institute in North Eastern India. | Sharma SR; Hussain M; Habung H | 2017 | Excluded | Irrelevant, did not discuss the subject matter |
| 123 | CLINICAL OUTCOMES OF CRYPTOCOCCAL MENINGITIS AMONG HIV-INFECTED PATIENTS IN THE ERA OF ANTIRETROVIRAL THERAPY. | Kobayashi T; Pitisuttithum P; Kaewkingwal J; Phuphuakrat A; Sungkanuparph S | 2017 | Excluded | Irrelevant, did not discuss the subject matter |
| 124 | Cryptococcal meningitis in patients with or without human immunodeficiency virus: experience in a tertiary hospital. | Lee SJ; Choi HK; Son J; Kim KH; Lee SH | 2011 | Excluded | Irrelevant, did not discuss the subject matter |
| 125 | Cryptococcal genotype influences immunologic response and human clinical outcome after meningitis. | Wiesner DL; Moskalenko O; Corcoran JM; McDonald T; Rolfes MA; Meya DB; Kajumbula H; Kambugu A; Bohjanen PR; Knight JF; Boulware DR; Nielsen K | 2012 | Excluded | Irrelevant, did not discuss the subject matter |
| 126 | Cryptococcal meningitis in non-HIV-infected patients in a Chinese tertiary care hospital, 1997-2007. | Zhu LP; Wu JQ; Xu B; Ou XT; Zhang QQ; Weng XH | 2010 | Excluded | Irrelevant, did not discuss the subject matter |
| 127 | Three-year mortality in cryptococcal meningitis: Hyperglycemia predict unfavorable outcome. | Tsai ST; Lin FY; Chen PS; Chiang HY; Kuo CC | 2021 | Excluded | Irrelevant, did not discuss the subject matter |
| 128 | LATERAL FLOW ASSAY FOR CRYPTOCOCCAL ANTIGEN: AN IMPORTANT ADVANCE TO IMPROVE THE CONTINUUM OF HIV CARE AND REDUCE CRYPTOCOCCAL MENINGITIS-RELATED MORTALITY. | Vidal JE; Boulware DR | 2015 | Excluded | Irrelevant, did not discuss the subject matter |
| 129 | The acceptability of the AMBITION-cm treatment regimen for HIV-associated cryptococcal meningitis: Findings from a qualitative methods study of participants and researchers in Botswana and Uganda. | Lawrence DS; Ssali A; Moshashane N; Nabaggala G; Maphane L; Harrison TS; Meya DB; Jarvis JN; Seeley J | 2022 | Excluded | Irrelevant, did not discuss the subject matter |
| 130 | Meningitis caused by Filobasidium uniguttulatum: case report and overview of the literature. | Pan W; Liao W; Hagen F; Theelen B; Shi W; Meis JF; Boekhout T | 2012 | Excluded | Irrelevant, did not discuss the subject matter |
| 131 | Screening for cryptococcal antigenemia in anti-retroviral naÃ¯ve AIDS patients in benin city, Nigeria. | Osazuwa F; Dirisu JO; Okuonghae PE; Ugbebor O | 2012 | Excluded | Irrelevant, did not discuss the subject matter |
| 132 | Identification of Cryptococcus antigen in human immunodeficiency virus-positive Turkish patients by using the Dynamiker(Â®) lateral flow assay. | Karaman E; Ilkit M; KuÅŸÃ§u F | 2019 | Excluded | Irrelevant, did not discuss the subject matter |
| 133 | Cost-Effectiveness Analysis of the Implementation of Cryptococcal Antigen Lateral Flow Assay for the Diagnosis of Cryptococcal Meningitis in Symptomatic People Living With Human Immunodeficiency Virus in Brazil. | Vianna CMM; Mosegui GBG | 2022 | Excluded | Irrelevant, did not discuss the subject matter |
| 134 | Prevalence of cryptococcosis in AtlÃ¡ntico, department of Colombia assessed with an active epidemiological search. | Noguera MC; EscandÃ³n P; ArÃ©valo M; GarcÃ­a Y; SuÃ¡rez LE; CastaÃ±eda E | 2019 | Excluded | Irrelevant, did not discuss the subject matter |
| 135 | Clinical features and treatment outcomes of human immunodeficiency virus-associated cryptococcal meningitis: a 2-year retrospective analysis. | Song W; Shen YZ; Wang ZY; Qi TK; Liu L; Zhang RF; Wang JR; Tang Y; Chen J; Sun JJ; Lu HZ | 2020 | Excluded | Irrelevant, did not discuss the subject matter |
| 136 | Magnitude of Cryptococcosis among HIV patients in sub-Saharan Africa countries: a systematic review and meta-analysis. | Alemayehu T; Ayalew S; Buzayehu T; Daka D | 2020 | Excluded | Irrelevant, did not discuss the subject matter |
| 137 | Cryptococcus neoformans- and Cryptococcus gattii-specific IgG, IgA and IgM differ among children and adults with and without cryptococcosis from Colombia. | Becerra-Ãlvarez P; EscandÃ³n P; Lizarazo J; QuirÃ³s-GÃ³mez Ã“; Firacative C | 2022 | Excluded | Irrelevant, did not discuss the subject matter |
| 138 | A nomogram for predicting paradoxical immune reconstitution inflammatory syndrome associated with cryptococcal meningitis among HIV-infected individuals in China. | Han X; Liu H; Wang Y; Wang P; Wang X; Yi Y; Li X | 2022 | Excluded | Irrelevant, did not discuss the subject matter |
| 139 | The clinical characteristics and outcome of cryptococcal meningitis with AIDS in a tertiary hospital in China: an observational cohort study. | Wu L; Xiao J; Song Y; Gao G; Zhao H | 2020 | Excluded | Irrelevant, did not discuss the subject matter |
| 140 | Cryptoccocal meningitis and HIV in the era of HAART in CÃ´te d'Ivoire. | Aoussi EF; Ehui E; DembÃ©lÃ© JP; Kolia-Diafouka P; Elloh NF; Ouattara SI; Tanon KA; Doumbia A; Adou-Bryn KD; EholiÃ© SP; BissagnÃ©nÃ© E | 2012 | Excluded | Irrelevant, did not discuss the subject matter |
| 141 | Cryptococcal meningitis due to Cryptococcus neoformans genotype AFLP1/VNI in Iran: a review of the literature. | Badali H; Alian S; Fakhim H; Falahatinejad M; Moradi A; Mohammad Davoudi M; Hagen F; Meis JF | 2015 | Excluded | Irrelevant, did not discuss the subject matter |
| 142 | HIV-Associated Cryptococcal Meningitis Patients Treated with Amphotericin B Deoxycholate Plus Flucytosine under Routine Care Conditions in a Referral Center in SÃ£o Paulo, Brazil. | Vidal JE; de Albuquerque Moraes C; de Siqueira REB; Miranda NFB; Marcusso R; Boulware DR; de Oliveira ACP | 2021 | Excluded | Irrelevant, did not discuss the subject matter |
| 143 | Comparison of features and outcomes between HIV-negative patients with Cryptococcus gattii meningitis and Cryptococcus neoformans meningitis in South China. | Su XH; Li WP; Liu JY; Wang YJ; Liu J; Xu XF; Yang L; Xia H; Jiang Y; Peng FH | 2022 | Excluded | Irrelevant, did not discuss the subject matter |
| 144 | Development and validation of quantitative PCR assays for HIV-associated cryptococcal meningitis in sub-Saharan Africa: aÂ diagnostic accuracy study. | Mbangiwa T; Sturny-LeclÃ¨re A; Lechiile K; Kajanga C; Boyer-Chammard T; Hoving JC; Leeme T; Moyo M; Youssouf N; Lawrence DS; Mwandumba H; Mosepele M; Harrison TS; Jarvis JN; Lortholary O; Alanio A | 2024 | Excluded | Irrelevant, did not discuss the subject matter |
| 145 | Retrospective Assessment of a National Reflex Cryptococcal Antigen Screening Program in South Africa Through Interlaboratory Comparison of Lateral Flow Assay Results. | Blasich NP; Coetzee LM; Sriruttan C; DeSanto D; Greene GS; Glencross DK; Govender NP | 2022 | Excluded | Irrelevant, did not discuss the subject matter |
| 146 | Therapeutic lumbar puncture and lumbar drainage: which is more effective for the management of intracranial hypertension in HIV patients with cryptococcal meningitis? Results of a prospective non-randomized interventional study in China. | Xu XL; Zhao T; Huang YQ; Lu YQ; He XJ; Wu YS; Zhang W; Yu JH; Yang TT; Xu LJ; Lan K; Zhang DF; Harypursat V; Chen YK | 2022 | Excluded | Irrelevant, did not discuss the subject matter |
| 147 | Evaluation of a national cryptococcal antigen screening program for HIV-infected patients in Uganda: A cost-effectiveness modeling analysis. | Rajasingham R; Meya DB; Greene GS; Jordan A; Nakawuka M; Chiller TM; Boulware DR; Larson BA | 2019 | Excluded | Irrelevant, did not discuss the subject matter |
| 148 | Increased mortality among HIV infected patients with cryptococcal antigenemia in Guinea-Bissau. | Thomsen D; Hviid CJ; HÃ¸nge BL; Medina C; TÃ© DDS; Correira FG; Ã˜stergaard L; Erikstrup C; Wejse C; Laursen AL; Jespersen S | 2018 | Excluded | Irrelevant, did not discuss the subject matter |
| 149 | Evaluation of screening and treatment of cryptococcal antigenaemia among HIV-infected persons in Soweto, South Africa. | Govender NP; Roy M; Mendes JF; Zulu TG; Chiller TM; Karstaedt AS | 2015 | Excluded | Irrelevant, did not discuss the subject matter |
| 150 | Cryptococcal antigenemia in anti-retroviral naÃ¯ve AIDS patients: prevalence and its association with CD4 cell count. | Osazuwa OF; Dirisu O; Okuonghae E | 2012 | Excluded | Irrelevant, did not discuss the subject matter |
| 151 | Evaluation of a Cryptococcus capsular polysaccharide detection FungiXpert LFA (lateral flow assay) for the rapid diagnosis of Cryptococcosis. | Liu Y; Kang M; Wu SY; Wu LJ; He L; Xiao YL; Zhang WL; Liao QF; Deng J; Chen ZX; Ma Y | 2022 | Excluded | Irrelevant, did not discuss the subject matter |
| 152 | Assessing the virulence of Cryptococcus neoformans causing meningitis in HIV infected and uninfected patients in Vietnam. | Thanh LT; Toffaletti DL; Tenor JL; Giamberardino C; Sempowski GD; Asfaw Y; Phan HT; Van Duong A; Trinh NM; Thwaites GE; Ashton PM; Chau NVV; Baker SG; Perfect JR; Day JN | 2020 | Excluded | Irrelevant, did not discuss the subject matter |
| 153 | High prevalence of cryptococcal infection among HIV-infected patients hospitalized with pneumonia in Thailand. | Harris JR; Lindsley MD; Henchaichon S; Poonwan N; Naorat S; Prapasiri P; Chantra S; Ruamcharoen F; Chang LS; Chittaganpitch M; Mehta N; Peruski L; Maloney SA; Park BJ; Baggett HC | 2012 | Excluded | Irrelevant, did not discuss the subject matter |
| 154 | Clinical features and risk factors of surgical site infections in HIV-negative patients with cryptococcal meningitis underwent ventriculoperitoneal shunt operations: a retrospective study. | Xu L; Zhu J; Wang X; Zeng G; Gao Z; Liu J | 2022 | Excluded | Irrelevant, did not discuss the subject matter |
| 155 | High concordance in plasma and CSF HIV-1 drug resistance mutations despite high cases of CSF viral escape in individuals with HIV-associated cryptococcal meningitis in Botswana. | Kelentse N; Moyo S; Choga WT; Lechiile K; Leeme TB; Lawrence DS; Kasvosve I; Musonda R; Mosepele M; Harrison TS; Jarvis JN; Gaseitsiwe S | 2022 | Excluded | Irrelevant, did not discuss the subject matter |
| 156 | A decade of antiretroviral therapy in Uganda: what are the emerging causes of death? | Kiragga AN; Mubiru F; Kambugu AD; Kamya MR; Castelnuovo B | 2019 | Excluded | Irrelevant, did not discuss the subject matter |
| 157 | Multisite validation of cryptococcal antigen lateral flow assay and quantification by laser thermal contrast. | Boulware DR; Rolfes MA; Rajasingham R; von Hohenberg M; Qin Z; Taseera K; Schutz C; Kwizera R; Butler EK; Meintjes G; Muzoora C; Bischof JC; Meya DB | 2014 | Excluded | Irrelevant, did not discuss the subject matter |
| 158 | Establishment of a novel scoring model for mortality risk prediction in HIV-infected patients with cryptococcal meningitis. | Zhao T; Xu XL; Nie JM; Chen XH; Jiang ZS; Liu SQ; Yang TT; Yang X; Sun F; Lu YQ; Harypursat V; Chen YK | 2021 | Excluded | Irrelevant, did not discuss the subject matter |
| 159 | Cryptococcal meningitis is a cause for cross-reactivity in cerebrospinal fluid assays for anti-Histoplasma, anti-Coccidioides and anti-Blastomyces antibodies. | Bahr NC; Panackal AA; Durkin MM; Smedema ML; Keown W; Davis TE; Raymond-Guillen L; Park YD; Marr KA; Fries BC; Williamson PR; Boulware DR; Wheat LJ | 2019 | Excluded | Irrelevant, did not discuss the subject matter |
| 160 | The effect of therapeutic lumbar punctures on acute mortality from cryptococcal meningitis. | Rolfes MA; Hullsiek KH; Rhein J; Nabeta HW; Taseera K; Schutz C; Musubire A; Rajasingham R; Williams DA; Thienemann F; Muzoora C; Meintjes G; Meya DB; Boulware DR | 2014 | Excluded | Irrelevant, did not discuss the subject matter |
| 161 | Clinical epidemiology and high genetic diversity amongst Cryptococcus spp. isolates infecting people living with HIV in Kinshasa, Democratic Republic of Congo. | Bive BZ; Sacheli R; Situakibanza Nani-Tuma H; Kabututu Zakayi P; Ka A; Mbula Mambimbi M; Muendele G; Boreux R; Landu N; Nzanzu Mudogo C; M'Buze PR; Moutschen M; Meyer W; Mvumbi Lelo G; Hayette MP | 2022 | Excluded | Irrelevant, did not discuss the subject matter |
| 162 | National coverage of reflex cryptococcal antigen screening: A milestone achievement in the care of persons with advanced HIV disease. | Govender NP; Glencross DK | 2018 | Excluded | Irrelevant, did not discuss the subject matter |
| 163 | Should we perform the serum cryptococcal antigen test in people living with HIV hospitalized due to a community-acquired pneumonia episode? | Silva AP; Zenatti CT; Figueiredo-Mello C; Negra MD; Levin AS; Boulware DR; Vidal JE | 2020 | Excluded | Irrelevant, did not discuss the subject matter |
| 164 | Risk Factors for Cryptococcal Meningitis Recurrence in Human Immunodeficiency Virus (HIV)-Infected Patients in a Large Chinese Acquired Immune Deficiency Syndrome (AIDS) Treatment Center. | Zhou Y; Li F; Li R; Peng Y; He M; Sun F; Yang M | 2021 | Excluded | Irrelevant, did not discuss the subject matter |
| 165 | Diagnostic Accuracy of Point of Care Cryptococcal Antigen Lateral Flow Assay in Fingerprick Whole Blood and Urine Samples for the Detection of Asymptomatic Cryptococcal Disease in Patients with Advanced HIV Disease. | Boyd K; Kouamou V; Hlupeni A; Tangwena Z; Ndhlovu CE; Makadzange AT | 2022 | Excluded | Irrelevant, did not discuss the subject matter |
| 166 | Three phylogenetic groups have driven the recent population expansion of Cryptococcus neoformans. | Ashton PM; Thanh LT; Trieu PH; Van Anh D; Trinh NM; Beardsley J; Kibengo F; Chierakul W; Dance DAB; Rattanavong S; Davong V; Hung LQ; Chau NVV; Tung NLN; Chan AK; Thwaites GE; Lalloo DG; Anscombe C; Nhat LTH; Perfect J; Dougan G; Baker S; Harris S; Day JN | 2019 | Excluded | Irrelevant, did not discuss the subject matter |
| 167 | Genetic influence of Toll-like receptors on non-HIV cryptococcal meningitis: An observational cohort study. | Jiang YK; Wu JQ; Zhao HZ; Wang X; Wang RY; Zhou LH; Yip CW; Huang LP; Cheng JH; Chen YH; Li H; Zhu LP; Weng XH | 2018 | Excluded | Irrelevant, did not discuss the subject matter |
| 168 | Outcomes of cryptococcal meningitis in Uganda before and after the availability of highly active antiretroviral therapy. | Kambugu A; Meya DB; Rhein J; O'Brien M; Janoff EN; Ronald AR; Kamya MR; Mayanja-Kizza H; Sande MA; Bohjanen PR; Boulware DR | 2008 | Excluded | Irrelevant, did not discuss the subject matter |
| 169 | Infectious causes of acute meningitis among Thai adults in a university hospital. | Aimbudlop K; Bruminhent J; Kiertiburanakul S | 2021 | Excluded | Irrelevant, did not discuss the subject matter |
| 170 | Pathways to care and outcomes among hospitalised HIV-seropositive persons with cryptococcal meningitis in South Africa. | Quan V; Toro-Silva S; Sriruttan C; Chetty V; Chihota V; Candfield S; Vassall A; Grant AD; Govender NP | 2019 | Excluded | Irrelevant, did not discuss the subject matter |
| 171 | Low CD4 count plus coma predicts cryptococcal meningitis in Tanzania. | Kisenge PR; Hawkins AT; Maro VP; McHele JP; Swai NS; Mueller A; Houpt ER | 2007 | Excluded | Irrelevant, did not discuss the subject matter |
| 172 | [Microbiological aspects of the cryptococcosis in the post-HAART era]. | Mazuelos EM; GarcÃ­a AI | 2010 | Excluded | Irrelevant, did not discuss the subject matter |
| 173 | Validation of clinic-based cryptococcal antigen lateral flow assay screening in HIV-infected adults in South Africa. | Drain PK; Hong T; Krows M; Govere S; Thulare H; Wallis CL; Gosnell BI; Moosa MY; Bassett IV; Celum C | 2019 | Excluded | Irrelevant, did not discuss the subject matter |
| 174 | Cryptococcal meningitis in Rio de Janeiro State, Brazil, 1994-2004. | Leimann BC; Koifman RJ | 2008 | Excluded | Irrelevant, did not discuss the subject matter |
| 175 | Susceptibility profile and epidemiological cut-off values of Cryptococcus neoformans species complex from Argentina. | CÃ³rdoba S; Isla MG; Szusz W; Vivot W; Altamirano R; Davel G | 2016 | Excluded | Irrelevant, did not discuss the subject matter |
| 176 | Comparison of clinical and biological characteristics of HIV-infected patients presenting Cryptococcus neoformans versus C. curvatus/C. laurentii meningitis. | Zono B; Moutschen M; Situakibanza H; Sacheli R; Muendele G; Kabututu P; Biakabuswa A; Landu N; Mvumbi G; Hayette MP | 2021 | Excluded | Irrelevant, did not discuss the subject matter |
| 177 | Cryptococcal meningitis in a tertiary hospital in Pretoria, mortality and risk factors - A retrospective cohort study. | Hiesgen J; Schutte C; Olorunju S; Retief J | 2017 | Excluded | Irrelevant, did not discuss the subject matter |
| 178 | Modelling the impact of CD4 testing on mortality from TB and cryptococcal meningitis among patients with advanced HIV disease in nine countries. | Oboho IK; Paulin H; Corcoran C; Hamilton M; Jordan A; Kirking HL; Agyemang E; Podewils LJ; Pretorius C; Greene G; Chiller T; Desai M; Bhatkoti R; Shiraishi RW; Shah NS | 2023 | Excluded | Irrelevant, did not discuss the subject matter |
| 179 | Cryptococcal Meningitis Presenting as a Complication in HIV-infected Children: A Case Series From Sub-Saharan Africa. | Nyazika TK; Masanganise F; Hagen F; Bwakura-Dangarembizi MF; Ticklay IM; Robertson VJ | 2016 | Excluded | Irrelevant, did not discuss the subject matter |
| 180 | Genotyping and antifungal susceptibility testing of Cryptococcus neoformans isolates from Cameroonian HIV-positive adult patients. | Bertout S; Drakulovski P; Kouanfack C; Krasteva D; Ngouana T; Dunyach-RÃ©my C; Dongtsa J; Aghokeng A; Delaporte E; Koulla-Shiro S; Reynes J; MalliÃ© M | 2013 | Excluded | Irrelevant, did not discuss the subject matter |
| 181 | Epidemiology of meningitis in an HIV-infected Ugandan cohort. | Rajasingham R; Rhein J; Klammer K; Musubire A; Nabeta H; Akampurira A; Mossel EC; Williams DA; Boxrud DJ; Crabtree MB; Miller BR; Rolfes MA; Tengsupakul S; Andama AO; Meya DB; Boulware DR | 2015 | Excluded | Irrelevant, did not discuss the subject matter |
| 182 | Trends of CNS Cryptococcosis during Pre- and Post-HIV era: A 38 years' retrospective cohort analysis from south India. | Lahiri S; Maji S; Manjunath N; Bahubali VH; Chandrashekar N | 2023 | Excluded | Irrelevant, did not discuss the subject matter |
| 183 | Predictive Model for Permanent Shunting in Cryptococcal meningitis. | Phusoongnern W; Anunnatsiri S; Sawanyawisuth K; Kitkhuandee A | 2017 | Excluded | Irrelevant, did not discuss the subject matter |
| 184 | A Prospective Evaluation of a Multisite Cryptococcal Screening and Treatment Program in HIV Clinics in Uganda. | Nalintya E; Meya DB; Lofgren S; Huppler Hullsiek K; Boulware DR; Rajasingham R | 2018 | Excluded | Irrelevant, did not discuss the subject matter |
| 185 | Epidemiological profile of cryptococcal meningitis patients in Rio Grande do Sul, Brazil. | Leal AL; Faganello J; Fuentefria AM; Boldo JT; Bassanesi MC; Vainstein MH | 2008 | Excluded | Irrelevant, did not discuss the subject matter |
| 186 | High Cryptococcal Antigen Titers in Blood Are Predictive of Subclinical Cryptococcal Meningitis Among Human Immunodeficiency Virus-Infected Patients. | Wake RM; Britz E; Sriruttan C; Rukasha I; Omar T; Spencer DC; Nel JS; Mashamaite S; Adelekan A; Chiller TM; Jarvis JN; Harrison TS; Govender NP | 2018 | Excluded | Irrelevant, did not discuss the subject matter |
| 187 | Relationship between intracranial pressure and antifungal agents levels in the CSF of patients with cryptococcal meningitis. | Wirth F; de Azevedo MI; Pilla C; Aquino VR; Neto GW; Goldani LZ | 2018 | Excluded | Irrelevant, did not discuss the subject matter |
| 188 | Cost-effectiveness analysis of flucytosine as induction therapy in the treatment of cryptococcal meningitis in HIV-infected adults in South Africa. | Miot J; Leong T; Takuva S; Parrish A; Dawood H | 2021 | Excluded | Irrelevant, did not discuss the subject matter |
| 189 | Cryptococcus gattii Complex Infections in HIV-Infected Patients, Southeastern United States. | Bruner KT; Franco-Paredes C; Henao-MartÃ­nez AF; Steele GM; Chastain DB | 2018 | Excluded | Irrelevant, did not discuss the subject matter |
| 190 | Six months survival and risk factors for attrition for patients detected with cryptococcal antigenemia through screening in Malawi. | Chisale MRO; Jordan A; Kamudumuli PS; Mvula B; Odo M; Maida A; Kandulu J; Chilima B; Sinyiza FW; Katundu P; Lee HY; Mtegha R; Wu TJ; Bitirinyo J; Nyirenda R; Kalua T; Greene G; Chiller T | 2023 | Excluded | Irrelevant, did not discuss the subject matter |
| 191 | Evaluation of fingerstick cryptococcal antigen lateral flow assay in HIV-infected persons: a diagnostic accuracy study. | Williams DA; Kiiza T; Kwizera R; Kiggundu R; Velamakanni S; Meya DB; Rhein J; Boulware DR | 2015 | Excluded | Irrelevant, did not discuss the subject matter |
| 192 | A case-control study of human immunodeficiency virus-negative patients with cryptococcemia and cryptococcal meningitis in a Chinese tertiary care hospital during 10 years. | Liang L; Liang Z; She D; Chen L | 2021 | Excluded | Irrelevant, did not discuss the subject matter |
| 193 | Point-of-Care Testing for Cryptococcal Disease Among Hospitalized Human Immunodeficiency Virus-Infected Adults in Ethiopia. | Mamuye AT; Bornstein E; Temesgen O; Blumberg HM; Kempker RR | 2016 | Excluded | Irrelevant, did not discuss the subject matter |
| 194 | Cryptococcal meningitis in Senegal: epidemiology, laboratory findings, therapeutic and outcome of cases diagnosed from 2004 to 2011. | Sow D; Tine RC; Sylla K; Djiba M; Ndour CT; Dieng T; Ndiaye JL; Faye B; Ndiaye D; Gaye O; Dieng Y | 2013 | Excluded | Irrelevant, did not discuss the subject matter |
| 195 | Clinical Diversity of CNS Cryptococcosis. | Koshy JM; Mohan S; Deodhar D; John M; Oberoi A; Pannu A | 2016 | Excluded | Irrelevant, did not discuss the subject matter |
| 196 | Is HIV-associated tuberculosis a risk factor for the development of cryptococcal disease? | Jarvis JN; Harrison TS; Corbett EL; Wood R; Lawn SD | 2010 | Excluded | Irrelevant, did not discuss the subject matter |
| 197 | Tuberculosis and neurocryptococcosis by Cryptococcus neoformans molecular type VNI in A non-HIV patient: A comorbidities case report. | Oliveira EP; InÃ¡cio CP; de Freitas JF; Valeriano CAT; Neves RP; Sobrinho PM; Lima-Neto RG | 2022 | Excluded | Irrelevant, did not discuss the subject matter |
| 198 | Phenotypic Variability Correlates with Clinical Outcome in Cryptococcus Isolates Obtained from Botswanan HIV/AIDS Patients. | Fernandes KE; Brockway A; Haverkamp M; Cuomo CA; van Ogtrop F; Perfect JR; Carter DA | 2018 | Excluded | Irrelevant, did not discuss the subject matter |
| 199 | Cryptococcal meningoencephalitis: Risk factors associated to death in a hospital in Northeastern Brazil. | Damasceno LS; CÃ©zar RC; Canuto MCDS; Barbosa Neto JP; LeitÃ£o TDMJS | 2023 | Excluded | Irrelevant, did not discuss the subject matter |
| 200 | An open label randomized controlled trial of tamoxifen combined with amphotericin B and fluconazole for cryptococcal meningitis. | Ngan NTT; Thanh Hoang Le N; Vi Vi NN; Van NTT; Mai NTH; Van Anh D; Trieu PH; Lan NPH; Phu NH; Chau NVV; Lalloo DG; Hope W; Beardsley J; White NJ; Geskus R; Thwaites GE; Krysan D; Tai LTH; Kestelyn E; Binh TQ; Hung LQ; Tung NLN; Day JN | 2021 | Excluded | Irrelevant, did not discuss the subject matter |
| 201 | Do Intracerebral Cytokine Responses Explain the Harmful Effects of Dexamethasone in Human Immunodeficiency Virus-associated Cryptococcal Meningitis? | Beardsley J; Hoang NLT; Kibengo FM; Tung NLN; Binh TQ; Hung LQ; Chierakul W; Thwaites GE; Chau NVV; Nguyen TTT; Geskus RB; Day JN | 2019 | Excluded | Irrelevant, did not discuss the subject matter |
| 202 | Lack of education, knowledge, and supplies are barriers to cryptococcal meningitis care among nurses and other healthcare providers in rural Uganda: A mixed methods study. | Link A; Okwir M; Iribarren S; Meya D; Bohjanen PR; Kasprzyk D | 2023 | Excluded | Irrelevant, did not discuss the subject matter |
| 203 | Prevalence of Asymptomatic Cryptococcal Antigenemia and Association with Follow-up Risk of Cryptococcal Meningitis and Mortality among HIV Infected Patients in North West India: A Prospective Cohort Study. | Bhati R; Pramendra S; Sejoo B; Kumar D; Bohra GK; Meena DS; Verma D; Midha NK | 2021 | Excluded | Irrelevant, did not discuss the subject matter |
| 204 | Incidence and predictors of reoccurrence of opportunistic infection among adult HIV/AIDS patients attending ART clinic at public health facilities in Arba Minch town, southern Ethiopia: A retrospective cohort study. | Dembelu M; Kote M; Gilano G; Mohammed T | 2021 | Excluded | Irrelevant, did not discuss the subject matter |
| 205 | Symptomatic relapse of HIV-associated cryptococcal meningitis in South Africa: the role of inadequate secondary prophylaxis. | Jarvis JN; Meintjes G; Williams Z; Rebe K; Harrison TS | 2010 | Excluded | Irrelevant, did not discuss the subject matter |
| 206 | The Lived Experience Of Participants in an African RandomiseD trial (LEOPARD): protocol for an in-depth qualitative study within a multisite randomised controlled trial for HIV-associated cryptococcal meningitis. | Lawrence DS; Tsholo K; Ssali A; Mupambireyi Z; Hoddinott G; Nyirenda D; Meya DB; Ndhlovu C; Harrison TS; Jarvis JN; Seeley J | 2021 | Excluded | Irrelevant, did not discuss the subject matter |
| 207 | Primary prophylaxis of cryptococcal disease with fluconazole in HIV-positive Ugandan adults: a double-blind, randomised, placebo-controlled trial. | Parkes-Ratanshi R; Wakeham K; Levin J; Namusoke D; Whitworth J; Coutinho A; Mugisha NK; Grosskurth H; Kamali A; Lalloo DG | 2011 | Excluded | Irrelevant, did not discuss the subject matter |
| 208 | Multilocus sequence typing of Cryptococcus neoformans var. grubii from Laos in a regional and global context. | Thanh LT; Phan TH; Rattanavong S; Nguyen TM; Duong AV; Dacon C; Hoang TN; Nguyen LPH; Tran CTH; Davong V; Nguyen CVV; Thwaites GE; Boni MF; Dance D; Ashton PM; Day JN | 2019 | Excluded | Irrelevant, did not discuss the subject matter |
| 209 | Cryptococcal antigenemia prevalence and clinical data in HIV-infected patients from the reference centre at INI-FIOCRUZ, Rio de Janeiro, Southeast of Brazil. | Ferreira MF; Brito-Santos F; Trilles L; Almeida MA; Wanke B; Veloso VG; Nunes EP; Lazera MDS | 2020 | Excluded | Irrelevant, did not discuss the subject matter |
| 210 | Cryptococcal meningitis in HIV-infected patients at Chiang Mai University Hospital: a retrospective study. | Chaiwarith R; Vongsanim S; Supparatpinyo K | 2014 | Excluded | Irrelevant, did not discuss the subject matter |
| 211 | Association of Antibody Immunity With Cryptococcal Antigenemia and Mortality in a South African Cohort With Advanced Human Immunodeficiency Virus Disease. | Yoon H; Wake RM; Nakouzi AS; Wang T; Agalliu I; Tiemessen CT; Govender NP; Jarvis JN; Harrison TS; Pirofski LA | 2023 | Excluded | Irrelevant, did not discuss the subject matter |
| 212 | Outpatient Cryptococcal Antigen Screening Is Associated With Favorable Baseline Characteristics and Improved Survival in Persons With Cryptococcal Meningitis in Uganda. | Levin AE; Bangdiwala AS; Nalintya E; Kagimu E; Kasibante J; Rutakingirwa MK; Mpoza E; Jjunju S; Nuwagira E; Naluyima R; Kirumira P; Hou C; Ssebambulidde K; Musubire AK; Williams DA; Abassi M; Muzoora C; Hullsiek KH; Rajasingham R; Meya DB; Boulware DR; Skipper CP | 2023 | Excluded | Irrelevant, did not discuss the subject matter |
| 213 | Apparent performance of metagenomic next-generation sequencing in the diagnosis of cryptococcal meningitis: a descriptive study. | Xing XW; Zhang JT; Ma YB; Zheng N; Yang F; Yu SY | 2019 | Excluded | Irrelevant, did not discuss the subject matter |
| 214 | High rates of cerebral toxoplasmosis in HIV patients presenting with meningitis in Accra, Ghana. | Opintan JA; Awadzi BK; Biney IJK; Ganu V; Doe R; Kenu E; Adu RF; Osei MM; Akumwena A; Grigg ME; Fahle GA; Newman MJ; Williamson PR; Lartey M | 2017 | Excluded | Irrelevant, did not discuss the subject matter |
| 215 | High prevalence of Cryptococcal antigenemia using a finger-prick lateral flow assay in individuals with advanced HIV disease in SantarÃ©m Municipality, Brazilian Amazon Basin. | Assy JGPL; Said RDC; Pinheiro OC; BrandÃ£o ADS; Boulware DR; FranÃ§a FOS; Vidal JE | 2021 | Excluded | Irrelevant, did not discuss the subject matter |
| 216 | High prevalence of central nervous system cryptococcosis using a fingerprick whole-blood lateral flow assay in individuals with neurological symptoms and advanced HIV disease in a Brazilian emergency department. | Oliveira FG; Nakagawa JAS; de Oliveira JMM; JÃºnior RML; Marcusso R; Vidal JE | 2023 | Excluded | Irrelevant, did not discuss the subject matter |
| 217 | Cost-effectiveness of primary prophylaxis of AIDS associated cryptococcosis in Cambodia. | Micol R; Tajahmady A; Lortholary O; Balkan S; Quillet C; Dousset JP; Chanroeun H; Madec Y; Fontanet A; Yazdanpanah Y | 2010 | Excluded | Irrelevant, did not discuss the subject matter |
| 218 | Molecular types of Cryptococcus species isolated from patients with cryptococcal meningitis in a Brazilian tertiary care hospital. | Wirth F; Azevedo MI; Goldani LZ | 2018 | Excluded | Irrelevant, did not discuss the subject matter |
| 219 | Prevalence of Cryptococcal Antigenemia and associated factors among HIV/AIDS patients on second-line antiretroviral therapy at two hospitals in Western Oromia, Ethiopia. | Geda N; Beyene T; Dabsu R; Mengist HM | 2019 | Excluded | Irrelevant, did not discuss the subject matter |
| 220 | Cryptococcal antigenemia and associated risk factors among ART-naÃ¯ve and ART-experienced HIV-infected peoples at selected health institutions of Mekelle, Northern Ethiopia. | Hailu K; Niguse S; Hagos K; Abdulkader M | 2019 | Excluded | Irrelevant, did not discuss the subject matter |
| 221 | Cryptoccocal meningitis in YaoundÃ© (Cameroon) HIV infected patients: Diagnosis, frequency and Cryptococcus neoformans isolates susceptibility study to fluconazole. | Kammalac Ngouana T; Dongtsa J; Kouanfack C; Tonfack C; Fomena S; MalliÃ© M; Delaporte E; Boyom FF; Bertout S | 2015 | Excluded | Irrelevant, did not discuss the subject matter |
| 222 | Access to Medicines for Treating People With Cryptococcal Meningitis. | Burry J; Casas CP; Ford N | 2023 | Excluded | Irrelevant, did not discuss the subject matter |
| 223 | Frequency of cryptococcal meningitis in HIV-1 infected patients in north central Nigeria. | Gomerep SS; Idoko JA; Ladep NG; Ugoya SO; Obaseki D; Agbaji OA; Agaba P; Akanbi MO; Badung BP; Otitoloju O | 2010 | Excluded | Irrelevant, did not discuss the subject matter |
| 224 | Diagnostic performance of a multiplex PCR assay for meningitis in an HIV-infected population in Uganda. | Rhein J; Bahr NC; Hemmert AC; Cloud JL; Bellamkonda S; Oswald C; Lo E; Nabeta H; Kiggundu R; Akampurira A; Musubire A; Williams DA; Meya DB; Boulware DR | 2016 | Excluded | Irrelevant, did not discuss the subject matter |
| 225 | Cryptococcal antigen test revisited: significance for cryptococcal meningitis therapy monitoring in a tertiary chinese hospital. | Lu H; Zhou Y; Yin Y; Pan X; Weng X | 2005 | Excluded | Irrelevant, did not discuss the subject matter |
| 226 | Efficacy of adjunctive sertraline for the treatment of HIV-associated cryptococcal meningitis: an open-label dose-ranging study. | Rhein J; Morawski BM; Hullsiek KH; Nabeta HW; Kiggundu R; Tugume L; Musubire A; Akampurira A; Smith KD; Alhadab A; Williams DA; Abassi M; Bahr NC; Velamakanni SS; Fisher J; Nielsen K; Meya DB; Boulware DR | 2016 | Excluded | Irrelevant, did not discuss the subject matter |
| 227 | Increased activity of the complement system in cerebrospinal fluid of the patients with Non-HIV Cryptococcal meningitis. | Shen L; Zheng J; Wang Y; Zhu M; Zhu H; Cheng Q; Li Q | 2017 | Excluded | Irrelevant, did not discuss the subject matter |
| 228 | [Fluconazole 1200mg or 800mg for cryptococcal meningitis treatment in Ivory Coast]. | Kouakou GA; Ello NF; Kassi NA; Keita M; Doumbia A; Mossou C; Kassi FK; Tanon A; Ehui E; EholiÃ© SP | 2017 | Excluded | Irrelevant, did not discuss the subject matter |
| 229 | Cryptococcus neoformans population diversity and clinical outcomes of HIV-associated cryptococcal meningitis patients in Zimbabwe. | Nyazika TK; Hagen F; Machiridza T; Kutepa M; Masanganise F; Hendrickx M; Boekhout T; Magombei-Majinjiwa T; Siziba N; Chin'ombe N; Mateveke K; Meis JF; Robertson VJ | 2016 | Excluded | Irrelevant, did not discuss the subject matter |
| 230 | Prevalence of cryptococcosis among HIV-infected patients in Yaounde, Cameroon. | Dzoyem JP; Kechia FA; Ngaba GP; Lunga PK; Lohoue PJ | 2012 | Excluded | Irrelevant, did not discuss the subject matter |
| 231 | Cryptococcal antigen positivity combined with the percentage of HIV-seropositive samples with CD4 counts <100 cells/Î¼l identifies districts in South Africa with advanced burden of disease. | Coetzee LM; Cassim N; Sriruttan C; Mhlanga M; Govender NP; Glencross DK | 2018 | Excluded | Irrelevant, did not discuss the subject matter |
| 232 | Comparison of two commercial tests (Immy vs. Dynamiker) for cryptococcal capsular antigen. | Noguera MC; EscandÃ³n P; RodrÃ­guez J; Parody A; Camargo L | 2021 | Excluded | Irrelevant, did not discuss the subject matter |
| 233 | Burden of cryptococcal meningitis in Malawi. | Makombe SD; Nkhata A; Schouten EJ; Kamoto K; Harries AD | 2009 | Excluded | Irrelevant, did not discuss the subject matter |
| 234 | Cryptococcal antigen prevalence in HIV-infected Tanzanians: a cross-sectional study and evaluation of a point-of-care lateral flow assay. | Rugemalila J; Maro VP; Kapanda G; Ndaro AJ; Jarvis JN | 2013 | Excluded | Irrelevant, did not discuss the subject matter |
| 235 | Post-infectious inflammatory response syndrome in an HIV-negative patient after Cryptococcus gattii meningoencephalitis: a case report and review of the literature. | Lan J; Lv L; Ye L; Wang T; Wu Z; Wu S; Peng C; Lu W; Lu T | 2023 | Excluded | Irrelevant, did not discuss the subject matter |
| 236 | Evaluation of five conventional and molecular approaches for diagnosis of cryptococcal meningitis in non-HIV-infected patients. | Chen M; Zhou J; Li J; Li M; Sun J; Fang WJ; Al-Hatmi AM; Xu J; Boekhout T; Liao WQ; Pan WH | 2016 | Excluded | Irrelevant, did not discuss the subject matter |
| 237 | Different characteristics of cryptococcal meningitis between HIV-infected and HIV-uninfected patients in the Southwest of China. | Liu Y; Kang M; Wu SY; Ma Y; Chen ZX; Xie Y; Tang JT | 2017 | Excluded | Irrelevant, did not discuss the subject matter |
| 238 | Short Communication: A Descriptive Analysis of Dried Blood Spot Adherence Testing Among Ugandans with HIV Presenting with Cryptococcal Meningitis. | Lofgren SM; Nicol MR; Kandole TK; Castillo-Mancilla J; Anderson PL; Mpoza E; Tugume L; Bangdiwala AS; Ssebambulidde K; Hullsiek KH; Rhein J; Meya DB; Boulware DR | 2021 | Excluded | Irrelevant, did not discuss the subject matter |
| 239 | Short-course amphotericin B in addition to sertraline and fluconazole for treatment of HIV-associated cryptococcal meningitis in rural Tanzania. | Katende A; Mbwanji G; Faini D; Nyuri A; Kalinjuma AV; Mnzava D; Hullsiek KH; Rhein J; Weisser M; Meya DB; Boulware DR; Letang E | 2019 | Excluded | Irrelevant, did not discuss the subject matter |
| 240 | An audit of the screen-and-treat intervention to reduce cryptococcal meningitis in HIV-positive patients with low CD4 count. | Ndayishimiye E; Ross AJ | 2018 | Excluded | Irrelevant, did not discuss the subject matter |
| 241 | CRYPTOCOCCAL meningitis in a HIV negative newly diagnosed diabetic patient: a CASE report. | Owuor OH; Chege P | 2019 | Excluded | Irrelevant, did not discuss the subject matter |
| 242 | Cryptococcal antigen screening by lay cadres using a rapid test at the point of care: A feasibility study in rural Lesotho. | Rick F; Niyibizi AA; Shroufi A; Onami K; Steele SJ; Kuleile M; Muleya I; Chiller T; Walker T; Van Cutsem G | 2017 | Excluded | Irrelevant, did not discuss the subject matter |
| 243 | Short Communication: Low seroprevalence of cryptococcal antigenaemia in patients with advanced HIV infection enrolling in an antiretroviral programme in Ghana. | Mamoojee Y; Shakoor S; Gorton RL; Sarfo S; Appiah LT; Norman B; Balakrishnan I; Phillips R; Chadwick D | 2011 | Excluded | Irrelevant, did not discuss the subject matter |
| 244 | Acridine orange fluorescent microscopy is more sensitive than India ink light microscopy in the rapid detection of cryptococcosis among CrAg positive HIV patients. | Kwizera R; Akampurira A; Williams D; Boulware DR; Meya DB | 2017 | Excluded | Irrelevant, did not discuss the subject matter |
| 245 | Short course amphotericin B with high dose fluconazole for HIV-associated cryptococcal meningitis. | Muzoora CK; Kabanda T; Ortu G; Ssentamu J; Hearn P; Mwesigye J; Longley N; Jarvis JN; Jaffar S; Harrison TS | 2012 | Excluded | Irrelevant, did not discuss the subject matter |
| 246 | Screening for cryptococcal antigenemia in patients accessing an antiretroviral treatment program in South Africa. | Jarvis JN; Lawn SD; Vogt M; Bangani N; Wood R; Harrison TS | 2009 | Excluded | Irrelevant, did not discuss the subject matter |
| 247 | [Molecular characterization of Cryptococcus neoformans isolates from HIV patients, Guayaquil, Ecuador]. | SÃ¡nchez S; Zambrano D; GarcÃ­a M; Bedoya C; FernÃ¡ndez C; Illnait-ZaragozÃ­ MT | 2017 | Excluded | Irrelevant, did not discuss the subject matter |
| 248 | Management of amphotericin-induced phlebitis among HIV patients with cryptococcal meningitis in a resource-limited setting: a prospective cohort study. | Ahimbisibwe C; Kwizera R; Ndyetukira JF; Kugonza F; Sadiq A; Hullsiek KH; Williams DA; Rhein J; Boulware DR; Meya DB | 2019 | Excluded | Irrelevant, did not discuss the subject matter |
| 249 | HIV-1 Central Nervous System Compartmentalization and Cytokine Interplay in Non-Subtype B HIV-1 Infections in Nigeria and Malawi. | Adewumi OM; Dukhovlinova E; Shehu NY; Zhou S; Council OD; Akanbi MO; Taiwo B; Ogunniyi A; Robertson K; Kanyama C; Hosseinipour MC; Swanstrom R | 2020 | Excluded | Irrelevant, did not discuss the subject matter |
| 250 | Epidemiology of adult meningitis during antiretroviral therapy scale-up in southern Africa: Results from the Botswana national meningitis survey. | Tenforde MW; Mokomane M; Leeme T; Tlhako N; Tsholo K; Ramodimoosi C; Dube B; Mokobela KO; Tawanana E; Chebani T; Pilatwe T; Hurt WJ; Mitchell HK; Molefi M; Mullan PC; Guthrie BL; Farquhar C; Steenhoff AP; Mine M; Jarvis JN | 2019 | Excluded | Irrelevant, did not discuss the subject matter |
| 251 | Treatment of cryptococcal meningitis in KwaZulu-Natal, South Africa. | Lightowler JV; Cooke GS; Mutevedzi P; Lessells RJ; Newell ML; Dedicoat M | 2010 | Excluded | Irrelevant, did not discuss the subject matter |
| 252 | Causes and outcome of hospitalization among HIV-infected adults receiving antiretroviral therapy in Mulago hospital, Uganda. | Namutebi AM; Kamya MR; Byakika-Kibwika P | 2013 | Excluded | Irrelevant, did not discuss the subject matter |
| 253 | International collaboration between US and Thailand on a clinical trial of treatment for HIV-associated cryptococcal meningitis. | Zimmer LO; Nolen TL; Pramanpol S; Wallace D; Walker ME; Pappas P; Chetchotisakd P | 2010 | Excluded | Irrelevant, did not discuss the subject matter |
| 254 | HIV-1C env and gag Variation in the Cerebrospinal Fluid and Plasma of Patients with HIV-Associated Cryptococcal Meningitis in Botswana. | Kelentse N; Moyo S; Mogwele ML; Ditshwanelo D; Mokaleng B; Moraka NO; Lechiile K; Leeme TB; Lawrence DS; Musonda R; Kasvosve I; Harrison TS; Jarvis JN; Gaseitsiwe S | 2020 | Excluded | Irrelevant, did not discuss the subject matter |
| 255 | Short-course High-dose Liposomal Amphotericin B for Human Immunodeficiency Virus-associated Cryptococcal Meningitis: A Phase 2 Randomized Controlled Trial. | Jarvis JN; Leeme TB; Molefi M; Chofle AA; Bidwell G; Tsholo K; Tlhako N; Mawoko N; Patel RKK; Tenforde MW; Muthoga C; Bisson GP; Kidola J; Changalucha J; Lawrence D; Jaffar S; Hope W; Molloy SLF; Harrison TS | 2019 | Excluded | Irrelevant, did not discuss the subject matter |
| 256 | Diagnostic Accuracy of the Biosynex CryptoPS Cryptococcal Antigen Semiquantitative Lateral Flow Assay in Patients with Advanced HIV Disease. | Tenforde MW; Boyer-Chammard T; Muthoga C; Tawe L; Milton T; Rulaganyang I; Lechiile K; Rukasha I; Leeme TB; Govender NP; Ngidi J; Mine M; Molloy SF; Harrison TS; Lortholary O; Jarvis JN | 2020 | Excluded | Irrelevant, did not discuss the subject matter |
| 257 | A phase II randomized controlled trial adding oral flucytosine to high-dose fluconazole, with short-course amphotericin B, for cryptococcal meningitis. | Jackson AT; Nussbaum JC; Phulusa J; Namarika D; Chikasema M; Kanyemba C; Jarvis JN; Jaffar S; Hosseinipour MC; van der Horst C; Harrison TS | 2012 | Excluded | Irrelevant, did not discuss the subject matter |
| 258 | High prevalence of Cryptococcal antigenemia among HIV-infected patients receiving antiretroviral therapy in Ethiopia. | Alemu AS; Kempker RR; Tenna A; Smitson C; Berhe N; Fekade D; Blumberg HM; Aseffa A | 2013 | Excluded | Irrelevant, did not discuss the subject matter |
| 259 | Poor long-term outcomes for cryptococcal meningitis in rural South Africa. | Lessells RJ; Mutevedzi PC; Heller T; Newell ML | 2011 | Excluded | Irrelevant, did not discuss the subject matter |
| 260 | Clinical presentation and opportunistic infections in HIV-1, HIV-2 and HIV-1/2 dual seropositive patients in Guinea-Bissau. | SÃ¸rensen A; Jespersen S; Katzenstein TL; Medina C; TÃ© Dda S; Correira FG; Hviid CJ; Laursen AL; Wejse C | 2016 | Excluded | Irrelevant, did not discuss the subject matter |
| 261 | The utility of cerebrospinal fluid white cell count during the prognostic assessment for cryptococcal meningitis patients: a retrospective study. | Qu J; Jiang J; Lv X | 2020 | Excluded | Irrelevant, did not discuss the subject matter |
| 262 | Genotypic diversity in clinical and environmental isolates of Cryptococcus neoformans from India using multilocus microsatellite and multilocus sequence typing. | Prakash A; Sundar G; Sharma B; Hagen F; Meis JF; Chowdhary A | 2020 | Excluded | Irrelevant, did not discuss the subject matter |
| 263 | Asymptomatic cryptococcal antigen prevalence detected by lateral flow assay in hospitalised HIV-infected patients in SÃ£o Paulo, Brazil. | Vidal JE; Toniolo C; Paulino A; Colombo A; Dos Anjos Martins M; da Silva Meira C; Pereira-Chioccola VL; Figueiredo-Mello C; Barros T; Duarte J; Fonseca F; Alves Cunha M; Mendes C; Ribero T; Dos Santos Lazera M; Rajasingham R; Boulware DR | 2016 | Excluded | Irrelevant, did not discuss the subject matter |
| 264 | Changing paradigm of cryptococcal meningitis: an eight-year experience from a tertiary hospital in South India. | Abhilash KP; Mitra S; Arul JJ; Raj PM; Balaji V; Kannangai R; Thomas SA; Abraham OC | 2015 | Excluded | Irrelevant, did not discuss the subject matter |
| 265 | Cryptococcal antigen screening and preemptive therapy in patients initiating antiretroviral therapy in resource-limited settings: a proposed algorithm for clinical implementation. | Jarvis JN; Govender N; Chiller T; Park BJ; Longley N; Meintjes G; Bekker LG; Wood R; Lawn SD; Harrison TS | 2012 | Excluded | Irrelevant, did not discuss the subject matter |
| 266 | Advanced Human Immunodeficiency Virus Disease in Botswana Following Successful Antiretroviral Therapy Rollout: Incidence of and Temporal Trends in Cryptococcal Meningitis. | Tenforde MW; Mokomane M; Leeme T; Patel RKK; Lekwape N; Ramodimoosi C; Dube B; Williams EA; Mokobela KO; Tawanana E; Pilatwe T; Hurt WJ; Mitchell H; Banda DL; Stone H; Molefi M; Mokgacha K; Phillips H; Mullan PC; Steenhoff AP; Mashalla Y; Mine M; Jarvis JN | 2017 | Excluded | Irrelevant, did not discuss the subject matter |
| 267 | Clinical evaluation of the antifungal effect of sertraline in the treatment of cryptococcal meningitis in HIV patients: a single Mexican center experience. | Villanueva-Lozano H; TreviÃ±o-Rangel RJ; GonzÃ¡lez GM; HernÃ¡ndez-RodrÃ­guez PA; Camacho-Ortiz A; Castillo-Reyna L; Galindo-Alvarado SG; MartÃ­nez-ResÃ©ndez MF | 2018 | Excluded | Irrelevant, did not discuss the subject matter |
| 268 | Voriconazole: a novel treatment option for cryptococcal meningitis. | Yao Y; Zhang JT; Yan B; Gao T; Xing XW; Tian CL; Huang XS; Yu SY | 2015 | Excluded | Irrelevant, did not discuss the subject matter |
| 269 | Cryptococcus neoformans var. grubii Infection in HIV-Seronegative Patients from Northeast India: Report of Two Cases with Review of Literature. | Nath R; Laskar B; Ahmed J; Das S; Timung L; Saikia L | 2016 | Excluded | Irrelevant, did not discuss the subject matter |
| 270 | Epidemiological characteristics of cryptococcal meningoencephalitis associated with Cryptococcus neoformans var. grubii from HIV-infected patients in Madagascar: A cross-sectional study. | Rakotoarivelo RA; Raberahona M; Rasamoelina T; Rabezanahary A; Rakotomalala FA; Razafinambinintsoa T; BÃ©net T; Vanhems P; Randria MJD; RomanÃ² L; Cogliati M; Cornet M; Rakoto Andrianarivelo M | 2020 | Excluded | Irrelevant, did not discuss the subject matter |
| 271 | Genomic epidemiology of Cryptococcus yeasts identifies adaptation to environmental niches underpinning infection across an African HIV/AIDS cohort. | Vanhove M; Beale MA; Rhodes J; Chanda D; Lakhi S; Kwenda G; Molloy S; Karunaharan N; Stone N; Harrison TS; Bicanic T; Fisher MC | 2017 | Excluded | Irrelevant, did not discuss the subject matter |
| 272 | One-year Mortality Outcomes From the Advancing Cryptococcal Meningitis Treatment for Africa Trial of Cryptococcal Meningitis Treatment in Malawi. | Kanyama C; Molloy SF; Chan AK; Lupiya D; Chawinga C; Adams J; Bright P; Lalloo DG; Heyderman RS; Lortholary O; Jaffar S; Loyse A; van Oosterhout JJ; Hosseinipour MC; Harrison TS | 2020 | Excluded | Irrelevant, did not discuss the subject matter |
| 273 | Prevalence and mortality of cryptococcal disease in adults with advanced HIV in an urban tertiary hospital in Sierra Leone: a prospective study. | Lakoh S; Rickman H; Sesay M; Kenneh S; Burke R; Baldeh M; Jiba DF; Tejan YS; Boyle S; Koroma C; Deen GF; Beynon F | 2020 | Excluded | Irrelevant, did not discuss the subject matter |
| 274 | Factors affecting mortality among HIV positive patients two years after completing recommended therapy for Cryptococcal meningitis in Uganda. | Kitonsa J; Mayanja Y; Aling E; Kiwanuka J; Namutundu J; Anywaine Z; Ggayi AB; Kibengo F; Kiwanuka N; Kaleebu P | 2019 | Excluded | Irrelevant, did not discuss the subject matter |
| 275 | Prevalence of cryptococcal antigenuria at initial HIV diagnosis in KwaZulu-Natal. | Drain PK; Kleene JM; Coleman SM; Losina E; Katz JN; Giddy J; Ross D; Freedberg KA; Bassett IV | 2015 | Excluded | Irrelevant, did not discuss the subject matter |
| 276 | Prognostic markers in AIDS-related cryptococcal meningitis. | Majumder S; Mandal SK; Bandyopadhyay D | 2011 | Excluded | Irrelevant, did not discuss the subject matter |
| 277 | Implementation and operational research: Integrated pre-antiretroviral therapy screening and treatment for tuberculosis and cryptococcal antigenemia. | Pac L; Horwitz MM; Namutebi AM; Auerbach BJ; Semeere A; Namulema T; Schwarz M; Bbosa R; Muruta A; Meya DB; Manabe YC | 2015 | Excluded | Irrelevant, did not discuss the subject matter |
| 278 | Prevalence and nature of potential drug-drug interactions among hospitalized HIV patients presenting with suspected meningitis in Uganda. | Eneh PC; Hullsiek KH; Kiiza D; Rhein J; Meya DB; Boulware DR; Nicol MR | 2020 | Excluded | Irrelevant, did not discuss the subject matter |
| 279 | Estimating the cost-per-result of a national reflexed Cryptococcal antigenaemia screening program: Forecasting the impact of potential HIV guideline changes and treatment goals. | Cassim N; Coetzee LM; Schnippel K; Glencross DK | 2017 | Excluded | Irrelevant, did not discuss the subject matter |
| 280 | Concurrent cryptococcal meningitis and disseminated tuberculosis occurring in an immunocompetent male. | Musabende M; Mukabatsinda C; Riviello ED; Ogbuagu O | 2016 | Excluded | Irrelevant, did not discuss the subject matter |
| 281 | Determinants of two-year mortality among HIV positive patients with Cryptococcal meningitis initiating standard antifungal treatment with or without adjunctive dexamethasone in Uganda. | Kitonsa J; Nsubuga R; Mayanja Y; Kiwanuka J; Nikweri Y; Onyango M; Anywaine Z; Ggayi AB; Kibengo FM; Kaleebu P; Day J | 2020 | Excluded | Irrelevant, did not discuss the subject matter |
| 282 | Nephrotoxicity of amphotericin B in the treatment of cryptococcal meningitis in acquired immunodeficiency syndrome patients. | Ochieng PO; McLigeyo SO; Amayo EO; Kayima JK; Omonge EO | 2009 | Excluded | Irrelevant, did not discuss the subject matter |
| 283 | Cryptococcal Meningitis Screening and Community-based Early Adherence Support in People With Advanced Human Immunodeficiency Virus Infection Starting Antiretroviral Therapy in Tanzania and Zambia: A Cost-effectiveness Analysis. | Kimaro GD; Guinness L; Shiri T; Kivuyo S; Chanda D; Bottomley C; Chen T; Kahwa A; Hawkins N; Mwaba P; Mfinanga SG; Harrison TS; Jaffar S; Niessen LW | 2020 | Excluded | Irrelevant, did not discuss the subject matter |
| 284 | Oral versus intravenous flucytosine in patients with human immunodeficiency virus-associated cryptococcal meningitis. | Brouwer AE; van Kan HJ; Johnson E; Rajanuwong A; Teparrukkul P; Wuthiekanun V; Chierakul W; Day N; Harrison TS | 2007 | Excluded | Irrelevant, did not discuss the subject matter |
| 285 | A comparative evaluation of three methods for the rapid diagnosis of cryptococcal meningitis (CM) among HIV-infected patients in Northern Malawi. | Chisale MR; Salema D; Sinyiza F; Mkwaila J; Kamudumuli P; Lee HY | 2020 | Excluded | Irrelevant, did not discuss the subject matter |
| 286 | Differences in human immunodeficiency virus-1C viral load and drug resistance mutation between plasma and cerebrospinal fluid in patients with human immunodeficiency virus-associated cryptococcal meningitis in Botswana. | Kelentse N; Moyo S; Mogwele M; Lechiile K; Moraka NO; Maruapula D; Seatla KK; Esele L; Molebatsi K; Leeme TB; Lawrence DS; Musonda R; Kasvosve I; Harrison TS; Jarvis JN; Gaseitsiwe S | 2020 | Excluded | Irrelevant, did not discuss the subject matter |
| 287 | Performance of cryptococcal antigen lateral flow assay in serum, cerebrospinal fluid, whole blood, and urine in HIV-infected patients with culture-proven cryptococcal meningitis admitted at a Brazilian referral center. | Vidal JE; Toniolo C; Paulino A; Colombo AL; Martins MDA; Meira CDS; Azevedo RGS; Pereira-Chioccola VL; Gomes HR; Lazera MDS; Oliveira ACP; Boulware DR | 2018 | Excluded | Irrelevant, did not discuss the subject matter |
| 288 | Multilocus sequence typing of serially collected isolates of Cryptococcus from HIV-infected patients in South Africa. | Van Wyk M; Govender NP; Mitchell TG; Litvintseva AP | 2014 | Excluded | Irrelevant, did not discuss the subject matter |
| 289 | Impaired consciousness and decreased glucose concentration of CSF as prognostic factors in immunocompetent patients with cryptococcal meningitis. | Zhang C; Tan Z; Tian F | 2020 | Excluded | Irrelevant, did not discuss the subject matter |
| 290 | Meningitis due to Cryptococcus neoformans: treatment with posaconazole. | Flores VG; Tovar RM; Zaldivar PG; Martinez EA | 2012 | Excluded | Irrelevant, did not discuss the subject matter |
| 291 | A prospective study of the immune reconstitution inflammatory syndrome (IRIS) in HIV-infected children from high prevalence countries. | Cotton MF; Rabie H; Nemes E; Mujuru H; Bobat R; Njau B; Violari A; Mave V; Mitchell C; Oleske J; Zimmer B; Varghese G; Pahwa S | 2019 | Excluded | Irrelevant, did not discuss the subject matter |
| 292 | Comparative genomics of Cryptococcus neoformans var. grubii associated with meningitis in HIV infected and uninfected patients in Vietnam. | Day JN; Qihui S; Thanh LT; Trieu PH; Van AD; Thu NH; Chau TTH; Lan NPH; Chau NVV; Ashton PM; Thwaites GE; Boni MF; Wolbers M; Nagarajan N; Tan PBO; Baker S | 2017 | Excluded | Irrelevant, did not discuss the subject matter |
| 293 | Cryptococcosis in an Infectious Diseases Hospital of Buenos Aires, Argentina. Revision of 2041 cases: Diagnosis, clinical features and therapeutics. | Arechavala A; Negroni R; Messina F; Romero M; MarÃ­n E; Depardo R; Walker L; Santiso G | 2018 | Excluded | Irrelevant, did not discuss the subject matter |
| 294 | Case report: a special case of cryptococcal infection-related inflammatory syndrome in a non-HIV infected and non-transplant patient. | Su XH; Li WP; Lin Q; Zheng XJ; Fang T; Jiang Y; Peng FH | 2022 | Excluded | Irrelevant, did not discuss the subject matter |
| 295 | [Opportunistic infections of the HIV/AIDS in adults in hospital settings in Togo]. | Apetse K; Assogba K; Kevi K; Balogou AA; Pitche P; Grunitzky E | 2011 | Excluded | Irrelevant, did not discuss the subject matter |
| 296 | Prevalence of central nervous system cryptococcosis in human immunodeficiency virus reactive hospitalized patients. | Lakshmi V; Sudha T; Teja VD; Umabala P | 2007 | Excluded | Irrelevant, did not discuss the subject matter |
| 297 | Outcomes of flucytosine-containing combination treatment for cryptococcal meningitis in a South African national access programme: a cross-sectional observational study. | Mashau RC; Meiring ST; Quan VC; Nel J; Greene GS; Garcia A; Menezes C; Reddy DL; Venter M; Stacey S; Madua M; Boretti L; Harrison TS; Meintjes G; Shroufi A; Trivino-Duran L; Black J; Govender NP | 2022 | Excluded | Irrelevant, did not discuss the subject matter |
| 298 | Long term 5-year survival of persons with cryptococcal meningitis or asymptomatic subclinical antigenemia in Uganda. | Butler EK; Boulware DR; Bohjanen PR; Meya DB | 2012 | Excluded | Irrelevant, did not discuss the subject matter |
| 299 | Geographic distribution of patients affected by Cryptococcus neoformans/Cryptococcus gattii species complexes meningitis, pigeon and tree populations in Southern Brazil. | Spina-Tensini T; Muro MD; Queiroz-Telles F; Strozzi I; Moraes ST; Petterle RR; Vettorello M; Staudacher C; Miguez LA; de Almeida SM | 2017 | Excluded | Irrelevant, did not discuss the subject matter |
| 300 | Prevalence and factors associated with cryptococcal antigenemia among severely immunosuppressed HIV-infected adults in Uganda: a cross-sectional study. | Oyella J; Meya D; Bajunirwe F; Kamya MR | 2012 | Excluded | Irrelevant, did not discuss the subject matter |
| 301 | Low diversity Cryptococcus neoformans variety grubii multilocus sequence types from Thailand are consistent with an ancestral African origin. | Simwami SP; Khayhan K; Henk DA; Aanensen DM; Boekhout T; Hagen F; Brouwer AE; Harrison TS; Donnelly CA; Fisher MC | 2011 | Excluded | Irrelevant, did not discuss the subject matter |
| 302 | Point of care testing evaluation of lateral flow immunoassay for diagnosis of cryptococcus meningitis in HIV-positive patients at an urban hospital in Nairobi, Kenya, 2017. | Gitonga LK; Boru WG; Kwena A; Maritim M; Wamicwe J; Ransom J | 2019 | Excluded | Irrelevant, did not discuss the subject matter |
| 303 | Screening for cryptococcal antigenemia using the lateral flow assay in antiretroviral therapy-naÃ¯ve HIV-positive adults at an Ethiopian hospital clinic. | Reepalu A; Balcha TT; Yitbarek T; Jarso G; SturegÃ¥rd E; BjÃ¶rkman P | 2015 | Excluded | Irrelevant, did not discuss the subject matter |
| 304 | Genotypes coding for mannose-binding lectin deficiency correlated with cryptococcal meningitis in HIV-uninfected Chinese patients. | Ou XT; Wu JQ; Zhu LP; Guan M; Xu B; Hu XP; Wang X; Weng XH | 2011 | Excluded | Irrelevant, did not discuss the subject matter |
| 305 | Cryptococcal meningitis in HIV infected: experience from a North Indian tertiary center. | Kumar S; Wanchu A; Chakrabarti A; Sharma A; Bambery P; Singh S | 2008 | Excluded | Irrelevant, did not discuss the subject matter |
| 306 | Dose response effect of high-dose fluconazole for HIV-associated cryptococcal meningitis in southwestern Uganda. | Longley N; Muzoora C; Taseera K; Mwesigye J; Rwebembera J; Chakera A; Wall E; Andia I; Jaffar S; Harrison TS | 2008 | Excluded | Irrelevant, did not discuss the subject matter |
| 307 | Genotypes of Cryptococcus neoformans and Cryptococcus gattii as agents of endemic cryptococcosis in Teresina, PiauÃ­ (northeastern Brazil). | Martins LM; Wanke B; LazÃ©ra Mdos S; Trilles L; Barbosa GG; Macedo RC; Cavalcanti Mdo A; EulÃ¡lio KD; Castro JA; Silva AS; Nascimento FF; Gouveia VA; Monte SJ | 2011 | Excluded | Irrelevant, did not discuss the subject matter |
| 308 | Profile of central nervous system disease in HIV/AIDS patients with special reference to cryptococcal infections. | Wadhwa A; Kaur R; Bhalla P | 2008 | Excluded | Irrelevant, did not discuss the subject matter |
| 309 | Comparison of clinical features in patients with persistent and nonpersistent cryptococcal meningitis: twelve years of clinical experience in four centers in China. | Xu XG; Pan WH; Bi XL; Fang W; Chen M; Zhu Y; Zhou J; Zhou N; Pan B; Li M; Liao WQ; Qi ZT | 2013 | Excluded | Irrelevant, did not discuss the subject matter |
| 310 | Comparison of clinical features and prognostic factors in HIV-negative adults with cryptococcal meningitis and tuberculous meningitis: a retrospective study. | Qu J; Zhou T; Zhong C; Deng R; LÃ¼ X | 2017 | Excluded | Irrelevant, did not discuss the subject matter |
| 311 | Regional Differences in the Prevalence of Major Opportunistic Infections among Antiretroviral-NaÃ¯ve Human Immunodeficiency Virus Patients in Japan, Northern Thailand, Northern Vietnam, and the Philippines. | Gangcuangco LMA; Sawada I; Tsuchiya N; Do CD; Pham TTT; Rojanawiwat A; Alejandria M; Leyritana K; Yokomaku Y; Pathipvanich P; Ariyoshi K | 2017 | Excluded | Irrelevant, did not discuss the subject matter |
| 312 | Predictors of poor clinical outcome of cryptococcal meningitis in HIV-infected patients. | Anekthananon T; Manosuthi W; Chetchotisakd P; Kiertiburanakul S; Supparatpinyo K; Ratanasuwan W; Pappas PG; Filler SG; Kopetskie HA; Nolen TL; Kendrick AS; Larsen RA | 2011 | Excluded | Irrelevant, did not discuss the subject matter |
| 313 | Evaluation of trypan blue stain in the TC20 automated cell counter as a point-of-care for the enumeration of viable cryptococcal cells in cerebrospinal fluid. | Kwizera R; Akampurira A; Kandole TK; Nabaggala MS; Williams DA; Kambugu A; Meya DB; Rhein J; Boulware DR | 2018 | Excluded | Irrelevant, did not discuss the subject matter |
| 314 | Prognostic implications of baseline anaemia and changes in haemoglobin concentrations with amphotericin B therapy for cryptococcal meningitis. | Tugume L; Morawski BM; Abassi M; Bahr NC; Kiggundu R; Nabeta HW; Hullsiek KH; Taseera K; Musubire AK; Schutz C; Muzoora C; Williams DA; Rolfes MA; Meintjes G; Rhein J; Meya DB; Boulware DR | 2017 | Excluded | Irrelevant, did not discuss the subject matter |
| 315 | Prevalence and outcomes of cryptococcal antigenemia in HIV-seropositive patients hospitalized for suspected tuberculosis in Uganda. | Andama AO; den Boon S; Meya D; Cattamanchi A; Worodria W; Davis JL; Walter ND; Yoo SD; Kalema N; Haller B; Huang L | 2013 | Excluded | Irrelevant, did not discuss the subject matter |
| 316 | Cryptococcus tetragattii as a major cause of cryptococcal meningitis among HIV-infected individuals in Harare, Zimbabwe. | Nyazika TK; Hagen F; Meis JF; Robertson VJ | 2016 | Excluded | Irrelevant, did not discuss the subject matter |
| 317 | Comparison of cryptococcal antigenemia between antiretroviral naÃ¯ve and antiretroviral experienced HIV positive patients at two hospitals in Ethiopia. | Beyene T; Woldeamanuel Y; Asrat D; Ayana G; Boulware DR | 2013 | Excluded | Irrelevant, did not discuss the subject matter |
| 318 | Epidemiological and TNFÎ± polymorphism evaluation in patients with cryptococcal meningitis treated at a referral hospital in North Brazil. | Diniz AMM; Feio DCA; Silva ASN; Burbano RR; Lima PDL | 2019 | Excluded | Irrelevant, did not discuss the subject matter |
| 319 | The costs of providing antiretroviral therapy services to HIV-infected individuals presenting with advanced HIV disease at public health centres in Dar es Salaam, Tanzania: Findings from a randomised trial evaluating different health care strategies. | Kimaro GD; Mfinanga S; Simms V; Kivuyo S; Bottomley C; Hawkins N; Harrison TS; Jaffar S; Guinness L | 2017 | Excluded | Irrelevant, did not discuss the subject matter |
| 320 | Case report: false negative serum cryptococcal latex agglutination test in a patient with disseminated cryptococcal disease. | Navabi N; Montebatsi M; Scott M; Gluckman SJ; Reid MJ | 2015 | Excluded | Irrelevant, did not discuss the subject matter |
| 321 | [Neuromeningeal cryptococcosis in an HIV-negative patient with pulmonary tuberculosis in the Infectious and tropical diseases department of the University teaching hospital Point G of Bamako, Mali]. | Loua OO; Alle Akakpo AE; Ouedraogo D; Cissoko Y; SoumarÃ© M; KonatÃ© I; Dao S | 2022 | Excluded | Irrelevant, did not discuss the subject matter |
| 322 | Cryptococcus meningitis and skin lesions in an HIV negative child. | Swe Han KS; Bekker A; Greeff S; Perkins DR | 2008 | Excluded | Irrelevant, did not discuss the subject matter |
| 323 | Utility of Cryptococcal Antigen Screening and Evolution of Asymptomatic Cryptococcal Antigenemia among HIV-Infected Women Starting Antiretroviral Therapy in Thailand. | Kwan CK; Leelawiwat W; Intalapaporn P; Anekthananon T; Raengsakulrach B; Peters PJ; McNicholl JM; Park BJ; McConnell MS; Weidle PJ | 2014 | Excluded | Irrelevant, did not discuss the subject matter |
| 324 | Epidemiology of cryptococcosis in Malaysia. | Tay ST; Rohani MY; Hoo TS; Hamimah H | 2010 | Excluded | Irrelevant, did not discuss the subject matter |
| 325 | Cryptococcal meningitis and immune reconstitution inflammatory syndrome in a pediatric patient with HIV after switching to second line antiretroviral therapy: a case report. | Otto SBJ; George PE; Mercedes R; Nabukeera-Barungi N | 2020 | Excluded | Irrelevant, did not discuss the subject matter |
| 326 | Ischemic stroke as a complication of cryptococcal meningitis and immune reconstitution inflammatory syndrome: a case report. | Ellis JP; Kalata N; Joekes EC; Kampondeni S; Benjamin LA; Harrison TS; Lalloo DG; Heyderman RS | 2018 | Excluded | Irrelevant, did not discuss the subject matter |
| 327 | Cryptococcal antigenemia screening among human immunodeficiency virus-infected cases with tuberculosis: Chance and cost effectiveness. | Yasri S; Wiwanitkit V | 2018 | Excluded | Irrelevant, did not discuss the subject matter |
| 328 | AMBIsome Therapy Induction OptimisatioN (AMBITION): High dose AmBisome for cryptococcal meningitis induction therapy in sub-Saharan Africa: economic evaluation protocol for a randomised controlled trial-based equivalence study. | Ponatshego PL; Lawrence DS; Youssouf N; Molloy SF; Alufandika M; Bango F; Boulware DR; Chawinga C; Dziwani E; Gondwe E; Hlupeni A; Hosseinipour MC; Kanyama C; Meya DB; Mosepele M; Muthoga C; Muzoora CK; Mwandumba H; Ndhlovu CE; Rajasingham R; Sayed S; Shamu S; Tsholo K; Tugume L; Williams D; Maheswaran H; Shiri T; Boyer-Chammard T; Loyse A; Chen T; Wang D; Lortholary O; Lalloo DG; Meintjes G; Jaffar S; Harrison TS; Jarvis JN; Niessen LW | 2019 | Excluded | Irrelevant, did not discuss the subject matter |
| 329 | Evaluation of trypan blue stain in a haemocytometer for rapid detection of cerebrospinal fluid sterility in HIV patients with cryptococcal meningitis. | Kwizera R; Akampurira A; Kandole TK; Nielsen K; Kambugu A; Meya DB; Boulware DR; Rhein J | 2017 | Excluded | Irrelevant, did not discuss the subject matter |
| 330 | AMBIsome Therapy Induction OptimisatioN (AMBITION): High Dose AmBisome for Cryptococcal Meningitis Induction Therapy in sub-Saharan Africa: Study Protocol for a Phase 3 Randomised Controlled Non-Inferiority Trial. | Lawrence DS; Youssouf N; Molloy SF; Alanio A; Alufandika M; Boulware DR; Boyer-Chammard T; Chen T; Dromer F; Hlupeni A; Hope W; Hosseinipour MC; Kanyama C; Lortholary O; Loyse A; Meya DB; Mosepele M; Muzoora C; Mwandumba HC; Ndhlovu CE; Niessen L; Schutz C; Stott KE; Wang D; Lalloo DG; Meintjes G; Jaffar S; Harrison TS; Jarvis JN | 2018 | Excluded | Irrelevant, did not discuss the subject matter |
| 331 | Symptomatic relapse of HIV-associated cryptococcal meningitis after initial fluconazole monotherapy: the role of fluconazole resistance and immune reconstitution. | Bicanic T; Harrison T; Niepieklo A; Dyakopu N; Meintjes G | 2006 | Excluded | Irrelevant, did not discuss the subject matter |
| 332 | No association of cryptococcal antigenemia with poor outcomes among antiretroviral therapy-experienced HIV-infected patients in Addis Ababa, Ethiopia. | Smitson CC; Tenna A; Tsegaye M; Alemu AS; Fekade D; Aseffa A; Blumberg HM; Kempker RR | 2014 | Excluded | Irrelevant, did not discuss the subject matter |
| 333 | Asymptomatic serum cryptococcal antigenemia and early mortality during antiretroviral therapy in rural Uganda. | Liechty CA; Solberg P; Were W; Ekwaru JP; Ransom RL; Weidle PJ; Downing R; Coutinho A; Mermin J | 2007 | Excluded | Irrelevant, did not discuss the subject matter |
| 334 | [Cryptococcus meningitis, five years of experience and literature review]. | Dorneanu O; Filip O; Miftode E; Radu I; Nicolau C; Damian I; Luca V | 2008 | Excluded | Irrelevant, did not discuss the subject matter |
| 335 | Prevalence and Hospital Management of Amphotericin B Deoxycholate-Related Toxicities during Treatment of HIV-Associated Cryptococcal Meningitis in South Africa. | Meiring S; Fortuin-de Smidt M; Kularatne R; Dawood H; Govender NP | 2016 | Excluded | Irrelevant, did not discuss the subject matter |
| 336 | Ocular complications of HIV infection in sub-Sahara Africa. | Nkomazana O; Tshitswana D | 2008 | Excluded | Irrelevant, did not discuss the subject matter |
| 337 | A case-control study of risk factors for HIV-negative children with cryptococcal meningitis in Shi Jiazhuang, China. | Guo J; Zhou J; Zhang S; Zhang X; Li J; Sun Y; Qi S | 2012 | Excluded | Irrelevant, did not discuss the subject matter |
| 338 | Cost effectiveness of cryptococcal antigen screening as a strategy to prevent HIV-associated cryptococcal meningitis in South Africa. | Jarvis JN; Harrison TS; Lawn SD; Meintjes G; Wood R; Cleary S | 2013 | Excluded | Irrelevant, did not discuss the subject matter |
| 339 | Early versus delayed antiretroviral therapy and cerebrospinal fluid fungal clearance in adults with HIV and cryptococcal meningitis. | Bisson GP; Molefi M; Bellamy S; Thakur R; Steenhoff A; Tamuhla N; Rantleru T; Tsimako I; Gluckman S; Ravimohan S; Weissman D; Tebas P | 2013 | Excluded | Irrelevant, did not discuss the subject matter |
| 340 | Point-of-care lateral flow assays for tuberculosis and cryptococcal antigenuria predict death in HIV infected adults in Uganda. | Manabe YC; Nonyane BA; Nakiyingi L; Mbabazi O; Lubega G; Shah M; Moulton LH; Joloba M; Ellner J; Dorman SE | 2014 | Excluded | Irrelevant, did not discuss the subject matter |
| 341 | An analysis of three opportunistic infections in an outpatient HIV clinic in Jamaica. | Barrow G; Clarke TR; Carrington D; Harvey K; Barton EN | 2010 | Excluded | Irrelevant, did not discuss the subject matter |
| 342 | [Forty cases of neuromeningeal cryptococcosis diagnosed at the Mycology-Parasitology Department of the Ibn Sina hospital in Rabat, over a 21-year period]. | Bandadi FZ; Raiss C; Moustachi A; Lyagoubi M; Aoufi S | 2019 | Excluded | Irrelevant, did not discuss the subject matter |
| 343 | MLST-Based Population Genetic Analysis in a Global Context Reveals Clonality amongst Cryptococcus neoformans var. grubii VNI Isolates from HIV Patients in Southeastern Brazil. | Ferreira-Paim K; Andrade-Silva L; Fonseca FM; Ferreira TB; Mora DJ; Andrade-Silva J; Khan A; Dao A; Reis EC; Almeida MT; Maltos A; Junior VR; Trilles L; Rickerts V; Chindamporn A; Sykes JE; Cogliati M; Nielsen K; Boekhout T; Fisher M; Kwon-Chung J; Engelthaler DM; LazÃ©ra M; Meyer W; Silva-Vergara ML | 2017 | Excluded | Irrelevant, did not discuss the subject matter |
| 344 | 'Time-to-amphotericin B' in cryptococcal meningitis in a European low-prevalence setting: analysis of diagnostic delays. | Katchanov J; von Kleist M; ArastÃ©h K; Stocker H | 2014 | Excluded | Irrelevant, did not discuss the subject matter |
| 345 | In-hospital mortality of HIV-infected cryptococcal meningitis patients with C. gattii and C. neoformans infection in Gaborone, Botswana. | Steele KT; Thakur R; Nthobatsang R; Steenhoff AP; Bisson GP | 2010 | Excluded | Irrelevant, did not discuss the subject matter |
| 346 | Brief Report: Point of Care Cryptococcal Antigen Screening: Pipetting Finger-Prick Blood Improves Performance of Immunomycologics Lateral Flow Assay. | Wake RM; Jarvis JN; Harrison TS; Govender NP | 2018 | Excluded | Irrelevant, did not discuss the subject matter |
| 347 | Molecular epidemiology reveals genetic diversity among 363 isolates of the Cryptococcus neoformans and Cryptococcus gattii species complex in 61 Ivorian HIV-positive patients. | Kassi FK; Drakulovski P; Bellet V; Krasteva D; Gatchitch F; Doumbia A; Kouakou GA; Delaporte E; Reynes J; MalliÃ© M; Menan HIE; Bertout S | 2016 | Excluded | Irrelevant, did not discuss the subject matter |
| 348 | Prevalence of HIV-associated cryptococcal meningitis and utility of microbiological determinants for its diagnosis in a tertiary care center. | Thakur R; Sarma S; Kushwaha S | 2008 | Excluded | Irrelevant, did not discuss the subject matter |
| 349 | Clinical study of 23 pediatric patients with cryptococcosis. | Luo FL; Tao YH; Wang YM; Li H | 2015 | Excluded | Irrelevant, did not discuss the subject matter |
| 350 | Cryptococcosis in Acquired Immunodeficiency Syndrome Patients Clinically Confirmed and/or Diagnosed at Necropsy in a Teaching Hospital in Brazil. | Torres RG; Etchebehere RM; Adad SJ; Micheletti AR; Ribeiro BM; Silva LE; Mora DJ; Paim KF; Silva-Vergara ML | 2016 | Excluded | Irrelevant, did not discuss the subject matter |
| 351 | Sensitivity and specificity of an index for the diagnosis of TB meningitis in patients in an urban teaching hospital in Malawi. | Checkley AM; Njalale Y; Scarborough M; Zjilstra EE | 2008 | Excluded | Irrelevant, did not discuss the subject matter |
| 352 | Early versus delayed initiation of antiretroviral therapy for concurrent HIV infection and cryptococcal meningitis in sub-saharan Africa. | Makadzange AT; Ndhlovu CE; Takarinda K; Reid M; Kurangwa M; Gona P; Hakim JG | 2010 | Excluded | Irrelevant, did not discuss the subject matter |
| 353 | Who is seeking antiretroviral treatment for HIV now? Characteristics of patients presenting in Kenya and South Africa in 2017-2018. | Brennan AT; Maskew M; Larson BA; Tsikhutsu I; Bii M; Vezi L; Fox MP; Venter WD; Ehrenkranz P; Rosen S | 2019 | Excluded | Irrelevant, did not discuss the subject matter |
| 354 | Feasibility and Acceptability of Cryptococcal Antigen Screening and Prevalence of Cryptocococcemia in Patients Attending a Resource-Limited HIV/AIDS Clinic in Malawi. | Chipungu C; Veltman JA; Jansen P; Chiliko P; Lossa C; Namarika D; Benner B; Hoffman RM; Bristow CC; Klausner JD | 2015 | Excluded | Irrelevant, did not discuss the subject matter |
| 355 | Screening HIV-Infected Patients with Low CD4 Counts for Cryptococcal Antigenemia prior to Initiation of Antiretroviral Therapy: Cost Effectiveness of Alternative Screening Strategies in South Africa. | Larson BA; Rockers PC; Bonawitz R; Sriruttan C; Glencross DK; Cassim N; Coetzee LM; Greene GS; Chiller TM; Vallabhaneni S; Long L; van Rensburg C; Govender NP | 2016 | Excluded | Irrelevant, did not discuss the subject matter |
| 356 | Cryptococcus neoformans ex vivo capsule size is associated with intracranial pressure and host immune response in HIV-associated cryptococcal meningitis. | Robertson EJ; Najjuka G; Rolfes MA; Akampurira A; Jain N; Anantharanjit J; von Hohenberg M; Tassieri M; Carlsson A; Meya DB; Harrison TS; Fries BC; Boulware DR; Bicanic T | 2014 | Excluded | Irrelevant, did not discuss the subject matter |
| 357 | The phenotype of the Cryptococcus-specific CD4+ memory T-cell response is associated with disease severity and outcome in HIV-associated cryptococcal meningitis. | Jarvis JN; Casazza JP; Stone HH; Meintjes G; Lawn SD; Levitz SM; Harrison TS; Koup RA | 2013 | Excluded | Irrelevant, did not discuss the subject matter |
| 358 | Role of quantitative CSF microscopy to predict culture status and outcome in HIV-associated cryptococcal meningitis in a Brazilian cohort. | Vidal JE; Gerhardt J; Peixoto de Miranda EJ; Dauar RF; Oliveira Filho GS; Penalva de Oliveira AC; Boulware DR | 2012 | Excluded | Irrelevant, did not discuss the subject matter |
| 359 | Timing of antiretroviral therapy after diagnosis of cryptococcal meningitis. | Boulware DR; Meya DB; Muzoora C; Rolfes MA; Huppler Hullsiek K; Musubire A; Taseera K; Nabeta HW; Schutz C; Williams DA; Rajasingham R; Rhein J; Thienemann F; Lo MW; Nielsen K; Bergemann TL; Kambugu A; Manabe YC; Janoff EN; Bohjanen PR; Meintjes G | 2014 | Excluded | Irrelevant, did not discuss the subject matter |
| 360 | The prevalence, clinical features, risk factors and outcome associated with cryptococcal meningitis in HIV positive patients in Kenya. | Mdodo R; Brown K; Omonge E; Jaoko W; Baddley J; Pappas P; Kempf MC; Aban I; Odera S; Suleh A; Jolly PE | 2010 | Excluded | Irrelevant, did not discuss the subject matter |
| 361 | A phase II randomized trial of amphotericin B alone or combined with fluconazole in the treatment of HIV-associated cryptococcal meningitis. | Pappas PG; Chetchotisakd P; Larsen RA; Manosuthi W; Morris MI; Anekthananon T; Sungkanuparph S; Supparatpinyo K; Nolen TL; Zimmer LO; Kendrick AS; Johnson P; Sobel JD; Filler SG | 2009 | Excluded | Irrelevant, did not discuss the subject matter |
| 362 | Multiple opportunistic fungal infections in an individual with severe HIV disease: A case report. | Almeida-Silva F; Damasceno LS; Serna MJ; Valero C; Quintella LP; Almeida-Paes R; Muniz Mde M; Zancope-Oliveira RM | 2016 | Excluded | Irrelevant, did not discuss the subject matter |
| 363 | Very low levels of 25-hydroxyvitamin D are not associated with immunologic changes or clinical outcome in South African patients with HIV-associated cryptococcal meningitis. | Jarvis JN; Bicanic T; Loyse A; Meintjes G; Hogan L; Roberts CH; Shoham S; Perfect JR; Govender NP; Harrison TS | 2014 | Excluded | Irrelevant, did not discuss the subject matter |
| 364 | [Prevalence and factors associated with cryptococcal antigenemia in HIV-infected patients in Cotonou/Benin]. | OgouyÃ¨mi-Hounto A; Zannou DM; Ayihounton G; Ahouada C; Azon-Kouanou A; Acakpo J; Sissinto Savi de Tove Y; Kinde Gazard D | 2016 | Excluded | Irrelevant, did not discuss the subject matter |
| 365 | Determinants of mortality in a combined cohort of 501 patients with HIV-associated Cryptococcal meningitis: implications for improving outcomes. | Jarvis JN; Bicanic T; Loyse A; Namarika D; Jackson A; Nussbaum JC; Longley N; Muzoora C; Phulusa J; Taseera K; Kanyembe C; Wilson D; Hosseinipour MC; Brouwer AE; Limmathurotsakul D; White N; van der Horst C; Wood R; Meintjes G; Bradley J; Jaffar S; Harrison T | 2014 | Excluded | Irrelevant, did not discuss the subject matter |
| 366 | Predictors of outcome in routine care for Cryptococcal meningitis in Western Kenya: lessons for HIV outpatient care in resource-limited settings. | Kendi C; Penner J; Koech J; Nyonda M; Cohen CR; Bukusi EA; Ngugi E; Meyer AC | 2013 | Excluded | Irrelevant, did not discuss the subject matter |
| 367 | In vitro antifungal activities of amphotericin B, 5-fluorocytosine, fluconazole and itraconazole against Cryptococcus neoformans isolated from cerebrospinal fluid and blood from patients in Serbia. | TrpkoviÄ‡ A; PekmezoviÄ‡ M; BaraÄ‡ A; CrnÄeviÄ‡ RadoviÄ‡ L; ArsiÄ‡ ArsenijeviÄ‡ V | 2012 | Excluded | Irrelevant, did not discuss the subject matter |
| 368 | Cryptococcal meningitis management in Tanzania with strict schedule of serial lumber punctures using intravenous tubing sets: an operational research study. | Meda J; Kalluvya S; Downs JA; Chofle AA; Seni J; Kidenya B; Fitzgerald DW; Peck RN | 2014 | Excluded | Irrelevant, did not discuss the subject matter |
| 369 | ISOLATION AND CHARACTERISATION OF CRYPTOCOCCUS NEOFORMANS AND CRYPTOCOCCUS GATTII FROM ENVIRONMENTAL SOURCES IN NAIROBI, KENYA. | Kangogo M; Boga H; Wanyoike W; Bii C | 2014 | Excluded | Irrelevant, did not discuss the subject matter |
| 370 | Lessons learned: Retrospective assessment of outcomes and management of patients with advanced HIV disease in a semi-urban polyclinic in Epworth, Zimbabwe. | Blankley S; Gashu T; Ahmad B; Belaye AK; Ringtho L; Mesic A; Zizhou S; Casas EC | 2019 | Excluded | Irrelevant, did not discuss the subject matter |
| 371 | Universal screening of Tanzanian HIV-infected adult inpatients with the serum cryptococcal antigen to improve diagnosis and reduce mortality: an operational study. | Wajanga BM; Kalluvya S; Downs JA; Johnson WD; Fitzgerald DW; Peck RN | 2011 | Excluded | Irrelevant, did not discuss the subject matter |
| 372 | A Glucuronoxylomannan-Associated Immune Signature, Characterized by Monocyte Deactivation and an Increased Interleukin 10 Level, Is a Predictor of Death in Cryptococcal Meningitis. | Scriven JE; Graham LM; Schutz C; Scriba TJ; Wilkinson KA; Wilkinson RJ; Boulware DR; Urban BC; Lalloo DG; Meintjes G | 2016 | Excluded | Irrelevant, did not discuss the subject matter |
| 373 | A prospective longitudinal study of the clinical outcomes from cryptococcal meningitis following treatment induction with 800 mg oral fluconazole in Blantyre, Malawi. | Rothe C; Sloan DJ; Goodson P; Chikafa J; Mukaka M; Denis B; Harrison T; van Oosterhout JJ; Heyderman RS; Lalloo DG; Allain T; Feasey NA | 2013 | Excluded | Irrelevant, did not discuss the subject matter |
| 374 | Liposomal amphotericin B (Fungisome) for the treatment of cryptococcal meningitis in HIV/AIDS patients in India: a multicentric, randomized controlled trial. | Jadhav MP; Bamba A; Shinde VM; Gogtay N; Kshirsagar NA; Bichile LS; Mathai D; Sharma A; Varma S; Digumarathi R | 2010 | Excluded | Irrelevant, did not discuss the subject matter |
| 375 | Incidence of common opportunistic infections in HIV-infected individuals in Pune, India: analysis by stages of immunosuppression represented by CD4 counts. | Ghate M; Deshpande S; Tripathy S; Nene M; Gedam P; Godbole S; Thakar M; Risbud A; Bollinger R; Mehendale S | 2009 | Excluded | Irrelevant, did not discuss the subject matter |
| 376 | Cryptococcal meningitis screening and community-based early adherence support in people with advanced HIV infection starting antiretroviral therapy in Tanzania and Zambia: an open-label, randomised controlled trial. | Mfinanga S; Chanda D; Kivuyo SL; Guinness L; Bottomley C; Simms V; Chijoka C; Masasi A; Kimaro G; Ngowi B; Kahwa A; Mwaba P; Harrison TS; Egwaga S; Jaffar S | 2015 | Excluded | Irrelevant, did not discuss the subject matter |
| 377 | Utility of urine and serum lateral flow assays to determine the prevalence and predictors of cryptococcal antigenemia in HIV-positive outpatients beginning antiretroviral therapy in Mwanza, Tanzania. | Magambo KA; Kalluvya SE; Kapoor SW; Seni J; Chofle AA; Fitzgerald DW; Downs JA | 2014 | Excluded | Irrelevant, did not discuss the subject matter |
| 378 | Symptomatic relapse of HIV-associated cryptococcal meningitis: recurrent cryptococcal meningitis or Cryptococcus-related immune reconstitution inflammatory syndrome? | Jhamb R; Kashyap B; Das S; Berry N; Garg A | 2014 | Excluded | Irrelevant, did not discuss the subject matter |
| 379 | A retrospective study of AIDS-associated cryptomeningitis. | Sachdeva RK; Randev S; Sharma A; Wanchu A; Chakrabarti A; Singh S; Varma S | 2012 | Excluded | Irrelevant, did not discuss the subject matter |
| 380 | Six-month outcomes of HIV-infected patients given short-course fluconazole therapy for asymptomatic cryptococcal antigenemia. | Kapoor SW; Magambo KA; Kalluvya SE; Fitzgerald DW; Peck RN; Downs JA | 2015 | Excluded | Irrelevant, did not discuss the subject matter |
| 381 | Cost-effectiveness of serum cryptococcal antigen screening to prevent deaths among HIV-infected persons with a CD4+ cell count < or = 100 cells/microL who start HIV therapy in resource-limited settings. | Meya DB; Manabe YC; Castelnuovo B; Cook BA; Elbireer AM; Kambugu A; Kamya MR; Bohjanen PR; Boulware DR | 2010 | Excluded | Irrelevant, did not discuss the subject matter |
| 382 | A Retrospective Cohort Study of Lesion Distribution of HIV-1 Infection Patients With Cryptococcal Meningoencephalitis on MRI: Correlation With Immunity and Immune Reconstitution. | Xia S; Li X; Shi Y; Liu J; Zhang M; Gu T; Pan S; Song L; Xu J; Sun Y; Zhao Q; Lu Z; Lu P; Li H | 2016 | Excluded | Irrelevant, did not discuss the subject matter |
| 383 | Tuberculous and cryptococcal meningitis in a setting with high TB and low HIV prevalence. | Khan A; Jamil B; Ali R; Sultan S | 2009 | Excluded | Irrelevant, did not discuss the subject matter |
| 384 | Prevalence, treatment, and factors associated with cryptococcal meningitis post introduction of integrase inhibitors antiretroviral based regimens among People Living with HIV in Tanzania. | Minja M; Mbilinyi T; Mkinga B; Philipo EG; Owenya J; Kilonzi M | 2024 | Excluded | Irrelevant, did not discuss the subject matter |
| 385 | Molecular epidemiology of cryptococcal genotype VNIc/ST5 in Siriraj Hospital, Thailand. | Hatthakaroon C; Pharkjaksu S; Chongtrakool P; Suwannakarn K; Kiratisin P; Ngamskulrungroj P | 2017 | Excluded | Irrelevant, did not discuss the subject matter |
| 386 | Fatal Cryptococcus gattii genotype VGI infection in an HIV-positive patient in Barranquilla, Colombia. | Noguera MC; EscandÃ³n P; CastaÃ±eda E | 2017 | Excluded | Irrelevant, did not discuss the subject matter |
| 387 | Chronic meningitis in Thailand. Clinical characteristics, laboratory data and outcome in patients with specific reference to tuberculosis and cryptococcosis. | Helbok R; Pongpakdee S; Yenjun S; Dent W; Beer R; Lackner P; Bunyaratvej P; Prasert B; Vejjajiva A; Schmutzhard E | 2006 | Excluded | Irrelevant, did not discuss the subject matter |
| 388 | Independent association between rate of clearance of infection and clinical outcome of HIV-associated cryptococcal meningitis: analysis of a combined cohort of 262 patients. | Bicanic T; Muzoora C; Brouwer AE; Meintjes G; Longley N; Taseera K; Rebe K; Loyse A; Jarvis J; Bekker LG; Wood R; Limmathurotsakul D; Chierakul W; Stepniewska K; White NJ; Jaffar S; Harrison TS | 2009 | Excluded | Irrelevant, did not discuss the subject matter |
| 389 | Prevalence, determinants of positivity, and clinical utility of cryptococcal antigenemia in Cambodian HIV-infected patients. | Micol R; Lortholary O; Sar B; Laureillard D; Ngeth C; Dousset JP; Chanroeun H; Ferradini L; Guerin PJ; Dromer F; Fontanet A | 2007 | Excluded | Irrelevant, did not discuss the subject matter |
| 390 | Epidemiological, clinical and biological profile of neuromeningeal cryptococcosis among people living with HIV in Kinshasa, Democratic Republic of Congo. | Zono B; Kamangu E; Situakibanza H; Amaela E; Bepouka B; Mbula M; Kayembe JM; Mvumbi G; Hayette MP | 2020 | Excluded | Irrelevant, did not discuss the subject matter |
| 391 | Decreasing incidence of cryptococcal meningitis in West Africa in the era of highly active antiretroviral therapy. | Bamba S; Lortholary O; Sawadogo A; Millogo A; GuiguemdÃ© RT; Bretagne S | 2012 | Excluded | Irrelevant, did not discuss the subject matter |
| 392 | Cause-specific mortality and the contribution of immune reconstitution inflammatory syndrome in the first 3 years after antiretroviral therapy initiation in an urban African cohort. | Castelnuovo B; Manabe YC; Kiragga A; Kamya M; Easterbrook P; Kambugu A | 2009 | Excluded | Irrelevant, did not discuss the subject matter |
| 393 | Case histories of infectious disease management in developing countries: Phnom Penh and Kabul. | Pilsczek FH | 2009 | Excluded | Irrelevant, did not discuss the subject matter |
| 394 | Combination flucytosine and high-dose fluconazole compared with fluconazole monotherapy for the treatment of cryptococcal meningitis: a randomized trial in Malawi. | Nussbaum JC; Jackson A; Namarika D; Phulusa J; Kenala J; Kanyemba C; Jarvis JN; Jaffar S; Hosseinipour MC; Kamwendo D; van der Horst CM; Harrison TS | 2010 | Excluded | Irrelevant, did not discuss the subject matter |
| 395 | Co-infection of molluscum contagiosum virus and cryptococcosis in the same skin lesion in a HIV-infected patient. | Annam V; Inamadar AC; Palit A; Yelikar BR | 2008 | Excluded | Irrelevant, did not discuss the subject matter |
| 396 | A prospective study of mortality from cryptococcal meningitis following treatment induction with 1200 mg oral fluconazole in Blantyre, Malawi. | Gaskell KM; Rothe C; Gnanadurai R; Goodson P; Jassi C; Heyderman RS; Allain TJ; Harrison TS; Lalloo DG; Sloan DJ; Feasey NA | 2014 | Excluded | Irrelevant, did not discuss the subject matter |
| 397 | Dual infections with pigmented and albino strains of Cryptococcus neoformans in patients with or without human immunodeficiency virus infection in India. | Mandal P; Banerjee U; Casadevall A; Nosanchuk JD | 2005 | Excluded | Irrelevant, did not discuss the subject matter |
| 398 | Clinical characteristics and prognostic factors of 60 patients with acquired immune deficiency syndrome combined with Cryptococcus neoformans. | Liu S; Chen W; Cheng F; Ye X; Pan N; Lu H | 2023 | Excluded | Irrelevant, did not discuss the subject matter |
| 399 | A retrospective study of contributing factors for prognosis and survival length of cryptococcal meningoencephalitis in Southern part of China (1998-2013). | Zheng H; Li M; Luo Y; Wang D; Yang J; Chen Q; Lao J; Chen N; Yang M; Wang Q | 2015 | Excluded | Irrelevant, did not discuss the subject matter |
| 400 | Relationship of cerebrospinal fluid pressure, fungal burden and outcome in patients with cryptococcal meningitis undergoing serial lumbar punctures. | Bicanic T; Brouwer AE; Meintjes G; Rebe K; Limmathurotsakul D; Chierakul W; Teparrakkul P; Loyse A; White NJ; Wood R; Jaffar S; Harrison T | 2009 | Excluded | Irrelevant, did not discuss the subject matter |
| 401 | In vitro antifungal susceptibility profiles of Cryptococcus species isolated from HIV-associated cryptococcal meningitis patients in Zimbabwe. | Nyazika TK; Herkert PF; Hagen F; Mateveke K; Robertson VJ; Meis JF | 2016 | Excluded | Irrelevant, did not discuss the subject matter |
| 402 | Immunological profiles of immune restoration disease presenting as mycobacterial lymphadenitis and cryptococcal meningitis. | Tan DB; Yong YK; Tan HY; Kamarulzaman A; Tan LH; Lim A; James I; French M; Price P | 2008 | Excluded | Irrelevant, did not discuss the subject matter |
| 403 | Increased Antifungal Drug Resistance in Clinical Isolates of Cryptococcus neoformans in Uganda. | Smith KD; Achan B; Hullsiek KH; McDonald TR; Okagaki LH; Alhadab AA; Akampurira A; Rhein JR; Meya DB; Boulware DR; Nielsen K | 2015 | Excluded | Irrelevant, did not discuss the subject matter |
| 404 | Cryptococcus deuterogattii VGIIa Infection Associated with Travel to the Pacific Northwest Outbreak Region in an Anti-Granulocyte-Macrophage Colony-Stimulating Factor Autoantibody-Positive Patient in the United States. | Applen Clancey S; Ciccone EJ; Coelho MA; Davis J; Ding L; Betancourt R; Glaubiger S; Lee Y; Holland SM; Gilligan P; Sung J; Heitman J | 2019 | Excluded | Irrelevant, did not discuss the subject matter |
| 405 | Management of cryptococcal meningitis in a district hospital in KwaZulu-Natal: a clinical audit. | Adeyemi BO; Ross A | 2014 | Excluded | Irrelevant, did not discuss the subject matter |
| 406 | Lessons learned about opportunistic infections in southeast Asia. | Nissapatorn V | 2008 | Excluded | Irrelevant, did not discuss the subject matter |
| 407 | Alternate-day versus once-daily administration of amphotericin B in the treatment of cryptococcal meningitis: a randomized controlled trial. | Techapornroong M; Suankratay C | 2007 | Excluded | Irrelevant, did not discuss the subject matter |
| 408 | [Cryptococcal neuromeningitidis in HIV-infected patients in Bangui, in the era of antiretroviral treatment]. | Gbangba-Ngai E; Fikouma V; Mossoro-Kpinde CD; Tekpa G; Ouavene JO; Yangba Mongba DS; Mbelesso P | 2014 | Excluded | Irrelevant, did not discuss the subject matter |
| 409 | CryptoDex: a randomised, double-blind, placebo-controlled phase III trial of adjunctive dexamethasone in HIV-infected adults with cryptococcal meningitis: study protocol for a randomised control trial. | Day J; Imran D; Ganiem AR; Tjahjani N; Wahyuningsih R; Adawiyah R; Dance D; Mayxay M; Newton P; Phetsouvanh R; Rattanavong S; Chan AK; Heyderman R; van Oosterhout JJ; Chierakul W; Day N; Kamali A; Kibengo F; Ruzagira E; Gray A; Lalloo DG; Beardsley J; Binh TQ; Chau TT; Chau NV; Cuc NT; Farrar J; Hien TT; Van Kinh N; Merson L; Phuong L; Tho LT; Thuy PT; Thwaites G; Wertheim H; Wolbers M | 2014 | Excluded | Irrelevant, did not discuss the subject matter |
| 410 | Cryptococcal meningitis: a blind spot in curbing AIDS. | Adams P | 2016 | Excluded | Irrelevant, did not discuss the subject matter |
| 411 | Human immunodeficiency virus (HIV) related cryptococcal meningitis in rural central Thailand--treatment difficulties and prevention strategies. | Wright P; Inverarity D | 2007 | Excluded | Irrelevant, did not discuss the subject matter |
| 412 | Clinical characteristics and outcomes of patients with cryptococcal meningoencephalitis in a resource-limited setting. | Chayakulkeeree M; Wangchinda P | 2014 | Excluded | Irrelevant, did not discuss the subject matter |
| 413 | Pulmonary coinfection by Pneumocystis jirovecii and Cryptococcus species in a patient with undiagnosed advanced HIV. | Valente-Acosta B; Padua-Garcia J; Tame-Elorduy A | 2020 | Excluded | Irrelevant, did not discuss the subject matter |
| 414 | Molecular typing and antifungal susceptibility of clinical sequential isolates of Cryptococcus neoformans from Sao Paulo State, Brazil. | Almeida AM; Matsumoto MT; Baeza LC; de Oliveira E Silva RB; Kleiner AA; Melhem Mde S; Mendes Giannini MJ | 2007 | Excluded | Irrelevant, did not discuss the subject matter |
| 415 | Cryptococcal meningitis in a newly diagnosed AIDS patient: a case report. | Salami AK; Ogunmodele JA; Fowotade A; Nwabuisi C; Wahab KW; Desalu OO; Fadeyi A | 2009 | Excluded | Irrelevant, did not discuss the subject matter |
| 416 | Cryptococcus neoformans isolates from YaoundÃ© human immunodeficiency virus-infected patients exhibited intra-individual genetic diversity and variation in antifungal susceptibility profiles between isolates from the same patient. | Kammalac Ngouana T; Drakulovski P; Krasteva D; Kouanfack C; Reynes J; Delaporte E; Boyom FF; MalliÃ© M; Bertout S | 2016 | Excluded | Irrelevant, did not discuss the subject matter |
| 417 | [Cryptococcus neoformans meningitis in children and adolescents infected with HIV]. | Quian J; GutiÃ©rrez S; GonzÃ¡lez V; SÃ¡nchez M; Abayian M; Baccino F | 2012 | Excluded | Irrelevant, did not discuss the subject matter |
| 418 | First report of two cases of cryptococcosis in Tripoli, Libya, infected with Cryptococcus neoformans isolates present in the urban area. | Ellabib MS; Krema ZA; Allafi AA; Cogliati M | 2017 | Excluded | Irrelevant, did not discuss the subject matter |
| 419 | Reproducibility of CSF quantitative culture methods for estimating rate of clearance in cryptococcal meningitis. | Dyal J; Akampurira A; Rhein J; Morawski BM; Kiggundu R; Nabeta HW; Musubire AK; Bahr NC; Williams DA; Bicanic T; Larsen RA; Meya DB; Boulware DR | 2016 | Excluded | Irrelevant, did not discuss the subject matter |
| 420 | Comparison of clinical features and survival between cryptococcosis in human immunodeficiency virus (HIV)-positive and HIV-negative patients. | Jongwutiwes U; Sungkanuparph S; Kiertiburanakul S | 2008 | Excluded | Irrelevant, did not discuss the subject matter |
| 421 | Outcomes of cryptococcal meningitis in antiretroviral naÃ¯ve and experienced patients in South Africa. | Jarvis JN; Meintjes G; Harrison TS | 2010 | Excluded | Irrelevant, did not discuss the subject matter |
| 422 | Population-based surveillance for cryptococcosis in an antiretroviral-naive South African province with a high HIV seroprevalence. | McCarthy KM; Morgan J; Wannemuehler KA; Mirza SA; Gould SM; Mhlongo N; Moeng P; Maloba BR; Crewe-Brown HH; Brandt ME; Hajjeh RA | 2006 | Excluded | Irrelevant, did not discuss the subject matter |
| 423 | AMBITION-cm: intermittent high dose AmBisome on a high dose fluconazole backbone for cryptococcal meningitis induction therapy in sub-Saharan Africa: study protocol for a randomized controlled trial. | Molefi M; Chofle AA; Molloy SF; Kalluvya S; Changalucha JM; Cainelli F; Leeme T; Lekwape N; Goldberg DW; Haverkamp M; Bisson GP; Perfect JR; Letang E; Fenner L; Meintjes G; Burton R; Makadzange T; Ndhlovu CE; Hope W; Harrison TS; Jarvis JN | 2015 | Excluded | Irrelevant, did not discuss the subject matter |
| 424 | Free fluconazole for cryptococcal meningitis: too little of a good thing? | Hamill RJ | 2006 | Excluded | Irrelevant, did not discuss the subject matter |
| 425 | Histopathological aspects of neurocryptococcosis in HIV-infected patients: autopsy report of 45 patients. | Klock C; Cerski M; Goldani LZ | 2009 | Excluded | Irrelevant, did not discuss the subject matter |
| 426 | A comparison of cases of paediatric-onset and adult-onset cryptococcosis detected through population-based surveillance, 2005-2007. | Meiring ST; Quan VC; Cohen C; Dawood H; Karstaedt AS; McCarthy KM; Whitelaw AC; Govender NP | 2012 | Excluded | Irrelevant, did not discuss the subject matter |
| 427 | Feasibility study of serial lumbar puncture and acetazolamide combination in the management of elevated cerebrospinal fluid pressure in AIDS patients with cryptococcal meningitis in Uganda. | Orem J; Tindyebwa L; Twinoweitu O; Mukasa B; Tomberland M; Mbidde EK | 2005 | Excluded | Irrelevant, did not discuss the subject matter |
| 428 | The use of HAART is associated with decreased risk of death during initial treatment of cryptococcal meningitis in adults in Botswana. | Bisson GP; Nthobatsong R; Thakur R; Lesetedi G; Vinekar K; Tebas P; Bennett JE; Gluckman S; Gaolathe T; MacGregor RR | 2008 | Excluded | Irrelevant, did not discuss the subject matter |
| 429 | [Neuromeningeal cryptococcosis in Mali]. | Minta DK; Dolo A; Dembele M; Kaya AS; Sidibe AT; Coulibaly I; Maiga II; Diallo M; Traore AM; Maiga MY; Doumbo OK; Traore HA; Pichard E; Chabasse D | 2011 | Excluded | Irrelevant, did not discuss the subject matter |
| 430 | Antifungal drug susceptibility of Cryptococcus neoformans from clinical sources in Nairobi, Kenya. | Bii CC; Makimura K; Abe S; Taguchi H; Mugasia OM; Revathi G; Wamae NC; Kamiya S | 2007 | Excluded | Irrelevant, did not discuss the subject matter |
| 431 | Long-term follow-up and survival of antiretroviral-naive patients with cryptococcal meningitis in the pre-antiretroviral therapy era, Gauteng Province, South Africa. | Park BJ; Shetty S; Ahlquist A; Greenbaum A; Miller JL; Motsi A; McCarthy K; Govender N | 2011 | Excluded | Irrelevant, did not discuss the subject matter |
| 432 | Evaluation of a newly developed lateral flow immunoassay for the diagnosis of cryptococcosis. | Lindsley MD; Mekha N; Baggett HC; Surinthong Y; Autthateinchai R; Sawatwong P; Harris JR; Park BJ; Chiller T; Balajee SA; Poonwan N | 2011 | Excluded | Irrelevant, did not discuss the subject matter |
| 433 | [Retrospective study of neuromeningeal cryptococcosis in patients infected with HIV in the infectious diseases unit of university hospital of Casablanca, Morocco]. | Dollo I; Marih L; El Fane M; Es-Sebbani M; Sodqi M; Oulad Lahsen A; Chakib A; El Kadioui F; Hamdani A; El Mabrouki MJ; Soussi Abdallaoui M; Karima Z; Hassoune S; Maaroufi A; Marhoum El Filali K | 2016 | Excluded | Irrelevant, did not discuss the subject matter |
| 434 | Extracellular enzymatic activities in Cryptococcus neoformans strains isolated from AIDS patients in different countries. | Vidotto V; Ito-Kuwa S; Nakamura K; Aoki S; Melhem M; Fukushima K; Bollo E | 2006 | Excluded | Irrelevant, did not discuss the subject matter |
| 435 | Most cases of cryptococcal meningitis in HIV-uninfected patients in Vietnam are due to a distinct amplified fragment length polymorphism-defined cluster of Cryptococcus neoformans var. grubii VN1. | Day JN; Hoang TN; Duong AV; Hong CT; Diep PT; Campbell JI; Sieu TP; Hien TT; Bui T; Boni MF; Lalloo DG; Carter D; Baker S; Farrar JJ | 2011 | Excluded | Irrelevant, did not discuss the subject matter |
| 436 | Performance of cryptococcal antigen lateral flow assay using saliva in Ugandans with CD4 <100. | Kwizera R; Nguna J; Kiragga A; Nakavuma J; Rajasingham R; Boulware DR; Meya DB | 2014 | Excluded | Irrelevant, did not discuss the subject matter |
| 437 | Non-adherence to antiretroviral therapy in patients infected with HIV and cryptococcal meningitis: two cases at the LambarÃ©nÃ© Hospital Center in Gabon. | Kombila UD; Iba Ba J; Tsoumbou-Bakana G; Moussavou Kombila JB | 2016 | Excluded | Irrelevant, did not discuss the subject matter |
| 438 | [Epidemiological, clinical and prognostic aspects of cryptococcal meningitis in hospital area of Bamako, Mali]. | Oumar AA; Dao S; Ba M; Poudiougou B; Diallo A | 2008 | Excluded | Irrelevant, did not discuss the subject matter |
| 439 | [Neuromeningeal cryptococcosis and AIDS: an 11-case series from Libreville, Gabon]. | Ondounda M; Mounguengui D; Mandji LJ; Magne C; Nziengui MM; Kombila U; Nzenze JR | 2010 | Excluded | Irrelevant, did not discuss the subject matter |
| 440 | A prospective descriptive study of cryptococcal meningitis in HIV uninfected patients in Vietnam - high prevalence of Cryptococcus neoformans var grubii in the absence of underlying disease. | Chau TT; Mai NH; Phu NH; Nghia HD; Chuong LV; Sinh DX; Duong VA; Diep PT; Campbell JI; Baker S; Hien TT; Lalloo DG; Farrar JJ; Day JN | 2010 | Excluded | Irrelevant, did not discuss the subject matter |
| 441 | Genotype and mating type distribution within clinical Cryptococcus neoformans and Cryptococcus gattii isolates from patients with cryptococcal meningitis in Uberaba, Minas Gerais, Brazil. | Mora DJ; Pedrosa AL; Rodrigues V; Leite Maffei CM; Trilles L; Dos Santos LazÃ©ra M; Silva-Vergara ML | 2010 | Excluded | Irrelevant, did not discuss the subject matter |
| 442 | [Non-neuromeningeal cryptococcosis in patients with AIDS in Bamako, Mali: 2Â case reports]. | Minta DK; TraorÃ© AM; Coulibaly I; Diallo K; Soukho-Kaya A; Dolo A; KamatÃ© B; Ouologuem DS; DembÃ©lÃ© M; TraorÃ© HA; Chabasse D; Pichard E | 2014 | Excluded | Irrelevant, did not discuss the subject matter |
| 443 | Chronic meningitis in immunocompromised adult Ethiopians visiting Tikur Anbessa Teaching Hospital and Ye'huleshet Clinic from 2003-2004. | Mihret W; Zenebe G; Bekele A; Abebe M; Wassie L; Yamuah LK; Woldemeskel D; Kassahun Y; Medhin G; Engers H; Aseffa A | 2014 | Excluded | Irrelevant, did not discuss the subject matter |
| 444 | Cryptococcal Antigen Detection in Serum: Significance as a Screening Modality for Indian PLHIV. | Bajpai S | 2017 | Excluded | Irrelevant, did not discuss the subject matter |
| 445 | False-negative cryptococcal antigen test due to the postzone phenomenon. | Kojima N; Chimombo M; Kahn DG | 2018 | Excluded | Irrelevant, did not discuss the subject matter |
| 446 | HIV-2 in the United States: rare but not forgotten. | Saraiya N; Kanagala V; Corpuz M | 2018 | Excluded | Irrelevant, did not discuss the subject matter |
| 447 | Primary and opportunistic pathogens associated with meningitis in adults in Bangui, Central African Republic, in relation to human immunodeficiency virus serostatus. | BÃ©kondi C; Bernede C; Passone N; Minssart P; Kamalo C; Mbolidi D; Germani Y | 2006 | Excluded | Irrelevant, did not discuss the subject matter |
| 448 | [Bacterial meningitis in HIV patient: think about co-infections!]. | Nadaud J; Villevieille T; Boulland P; Allanic L; Plancade D; Steiner T; Favier JC; RÃ¼ttimann M; Puyhardy JM | 2007 | Excluded | Irrelevant, did not discuss the subject matter |
| 449 | Clinical and mycological profile of cryptococcosis in a tertiary care hospital. | Capoor MR; Nair D; Deb M; Gupta B; Aggarwal P | 2007 | Excluded | Irrelevant, did not discuss the subject matter |
| 450 | Cryptococcal pneumonia in African miners at autopsy. | Wong ML; Back P; Candy G; Nelson G; Murray J | 2007 | Excluded | Irrelevant, did not discuss the subject matter |
| 451 | Fatal Case of Polymicrobial Meningitis Caused by Cryptococcus liquefaciens and Mycobacterium tuberculosis Complex in a Human Immunodeficiency Virus-Infected Patient. | Conde-Pereira C; Rodas-RodrÃ­guez L; DÃ­az-Paz M; Palacios-Rivera H; Firacative C; Meyer W; AlcÃ¡zar-Castillo M | 2015 | Excluded | Irrelevant, did not discuss the subject matter |
| 452 | [Factors associated with mortality in cryptococcal meningitis]. | Wu JQ; Xu B; Ou XT; Zhu LP; Weng XH | 2010 | Excluded | Irrelevant, did not discuss the subject matter |
| 453 | Mortality & clinical characteristics of hospitalized adult patients with HIV in Pune, India. | Sobhani R; Basavaraj A; Gupta A; Bhave AS; Kadam DB; Sangle SA; Prasad HB; Choi J; Josephs J; Gebo KA; Morde SN; Bollinger RC Jr; Kakrani AL | 2007 | Excluded | Irrelevant, did not discuss the subject matter |
| 454 | Cryptococcal meningitis in patients with acquired immunudeficiency syndrome in prehaart era at Gondar College of Medical Sciences Hospital north-west Ethiopia. | Seboxa T; Alemu S; Assefa A; Asefa A; Diro E | 2010 | Excluded | Irrelevant, did not discuss the subject matter |
| 455 | AIDS-related opportunistic mycoses seen in a tertiary care hospital in North India. | Wadhwa A; Kaur R; Agarwal SK; Jain S; Bhalla P | 2007 | Excluded | Irrelevant, did not discuss the subject matter |
| 456 | Multiple intracranial cryptococcomas in an immunocompetent patient with pulmonary involvement. | Raman S; Mukherjee N; Dash K; Sen KK | 2020 | Excluded | Irrelevant, did not discuss the subject matter |
| 457 | Cryptococcal meningitis with an antecedent cutaneous Cryptococcal lesion. | Tilak R; Prakash P; Nigam C; Tilak V; Gambhir IS; Gulati AK | 2009 | Excluded | Irrelevant, did not discuss the subject matter |
| 458 | Cryptococcus gattii infection: characteristics and epidemiology of cases identified in a South African province with high HIV seroprevalence, 2002-2004. | Morgan J; McCarthy KM; Gould S; Fan K; Arthington-Skaggs B; Iqbal N; Stamey K; Hajjeh RA; Brandt ME | 2006 | Excluded | Irrelevant, did not discuss the subject matter |
| 459 | Atypical forms of Cryptococcus neoformans in CSF of an AIDS patient. | Bava J; Solari R; Isla G; Troncoso A | 2008 | Excluded | Irrelevant, did not discuss the subject matter |
| 460 | Prevalence of clinical isolates of Cryptococcus gattii serotype C among patients with AIDS in Sub-Saharan Africa. | Litvintseva AP; Thakur R; Reller LB; Mitchell TG | 2005 | Excluded | Irrelevant, did not discuss the subject matter |
| 461 | [Retrospective study of cases of neuromeningeal cryptococcosis at the University Hospital of Bobo Dioulasso since accessibility to antiretroviral in Burkina Faso]. | Bamba S; Barro-TraorÃ© F; Sawadogo E; Millogo A; GuiguemdÃ© RT | 2012 | Excluded | Irrelevant, did not discuss the subject matter |
| 462 | Challenges in diagnosis and management of Cryptococcal immune reconstitution inflammatory syndrome (IRIS) in resource limited settings. | Musubire AK; Meya BD; Mayanja-Kizza H; Lukande R; Wiesner LD; Bohjanen P; R Boulware RD | 2012 | Excluded | Irrelevant, did not discuss the subject matter |
| 463 | Cryptococcal peritonitis as the first AIDS-defining illness in Zimbabwe. | Ferrand RA; Maunganidze AJ; Mgodi NM | 2006 | Excluded | Irrelevant, did not discuss the subject matter |
| 464 | Testing but not treating: missed opportunities and lost lives in the South African antiretroviral therapy programme. | Jarvis JN; Meintjes G; Wood R; Harrison TS | 2010 | Excluded | Irrelevant, did not discuss the subject matter |
| 465 | Comparison of biotyping methods as alternative identification tools to molecular typing of pathogenic Cryptococcus species in sub-Saharan Africa. | Nyazika TK; Robertson VJ; Nherera B; Mapondera PT; Meis JF; Hagen F | 2016 | Excluded | Irrelevant, did not discuss the subject matter |
| 466 | [Results of nine years of the clinical and epidemiological survey on cryptococcosis in Colombia, 1997-2005]. | Lizarazo J; Linares M; de Bedout C; Restrepo A; Agudelo CI; CastaÃ±eda E | 2007 | Excluded | Irrelevant, did not discuss the subject matter |
| 467 | Lymphocyte transformation assay for C neoformans antigen is not reliable for detecting cellular impairment in patients with neurocryptococcosis. | Rocha KC; Pinhal C; Cavalcanti S; Vidal MS; Toscano M; Moraes-Vasconcelos D; Duarte AJ; Fonseca FL; de Abreu LC; Valenti VE; Grumach AS | 2012 | Excluded | Irrelevant, did not discuss the subject matter |
| 468 | Concomitant disseminated histoplasmosis and cryptococcosis in a person with AIDS. | Swaminathan S; Imrit K; Green J; Das K | 2006 | Excluded | Irrelevant, did not discuss the subject matter |
| 469 | [Causes of death in patients with HIV infection in two Tunisian medical centers]. | Chelli J; Bellazreg F; Aouem A; Hattab Z; Mesmia H; Lasfar NB; Hachfi W; Masmoudi T; Chakroun M; Letaief A | 2016 | Excluded | Irrelevant, did not discuss the subject matter |
| 470 | Cryptococcocal immune reconstitution disease: a major cause of early mortality in a South African antiretroviral programme. | Lawn SD; Bekker LG; Myer L; Orrell C; Wood R | 2005 | Excluded | Irrelevant, did not discuss the subject matter |
| 471 | Cryptococcal meningitis in a tertiary care hospital. | Juhi T; BibhaBati M; Aradhana B; Poonam L; Vinita D; Archana T | 2009 | Excluded | Irrelevant, did not discuss the subject matter |
| 472 | Concerns regarding a randomized study of the timing of antiretroviral therapy in zimbabweans with AIDS and acute cryptococcal meningitis. | Grant PM; Aberg JA; Zolopa AR | 2010 | Excluded | Irrelevant, did not discuss the subject matter |
| 473 | Pulmonary cryptococcosis misdiagnosed as smear-negative pulmonary tuberculosis with fatal consequences. | Jarvis JN; Wainwright H; Harrison TS; Rebe K; Meintjes G | 2010 | Excluded | Irrelevant, did not discuss the subject matter |
| 474 | Prevalence of opportunistic infections in AIDS patients in Mangalore, Karnataka. | Saldanha D; Gupta N; Shenoy S; Saralaya V | 2008 | Excluded | Irrelevant, did not discuss the subject matter |
| 475 | Acute demyelinating encephalomyelitis (ADEM), cryptococcal reactivation and disseminated Herpes simplex in an HIV infected child following HAART. | van Toorn R; Kritzinger F; Rabie H | 2005 | Excluded | Irrelevant, did not discuss the subject matter |
| 476 | [Cryptococcal neuromeningitis in immunosuppressed subjects at Rabat University Hospital (Morocco)]. | Aoufi S; Agoumi A; Seqat M | 2008 | Excluded | Irrelevant, did not discuss the subject matter |
| 477 | [Immune reconstitution inflammatory syndrome in cryptococcal meningitis: a rare phenomenon?]. | Schulze K; Schmiedel S; van Lunzen J | 2012 | Excluded | Irrelevant, did not discuss the subject matter |
| 478 | [Cryptococcosis in AIDS patients: case study from 1996 to 2006 in Paroissien Hospital]. | MÃ³naco LS; Tamayo Antabak N | 2008 | Excluded | Irrelevant, did not discuss the subject matter |
| 479 | [Multiple facial nodules revealing disseminated cryptococcosis in an immunocompetent patient]. | Rachadi H; Senouci K; Lyagoubi M; Benzekri A; Mansouri S; Ramli I; Ismaili N; Hassam B; Benzekri L | 2016 | Excluded | Irrelevant, did not discuss the subject matter |
| 480 | [Twenty-two cases of neuromeningeal cryptococcosis in Tunisia]. | Kaouech E; Kallel K; Belhadj S; Anane S; Ben ChÃ¢abane T; Ben Fadhl K; Khedher A; Meddeb B; Ben Lakhal S; Chaker E | 2009 | Excluded | Irrelevant, did not discuss the subject matter |
| 481 | Case 8-2017. A 39-Year-Old Zimbabwean Man with a Severe Headache. | Boulware DR; Makadzange AT | 2017 | Excluded | Irrelevant, did not discuss the subject matter |
| 482 | CRYPTOCOCCAL MENINGITIS IN IMMUNOCOMPETENT PATIENT. | Arif S; Ghazanfar K; Muhammad WW; Malik H | 2015 | Excluded | Irrelevant, did not discuss the subject matter |
| 483 | [Lymphocytic meningitis in Bamako, Mali]. | Minta DK; Dembele M; Kaya AS; Sidibe AT; Coulibaly I; Mieret S; Diallo B; Traore A; Ba B; Sidibe AF; Diallo DA; Traore AK; Traore HA | 2010 | Excluded | Irrelevant, did not discuss the subject matter |
| 484 | Mortality rate of early versus deferred initiation of antiretroviral therapy in HIV-1-infected patients with cryptococcal meningitis. | Manosuthi W; Chottanapund S; Sungkanuparph S | 2008 | Excluded | Irrelevant, did not discuss the subject matter |
| 485 | Treatment with adalimumab for severe immune reconstitution inflammatory syndrome in an HIV-infected patient presenting with cryptococcal meningitis. | Gaube G; De Castro N; Gueguen A; Lascoux C; Zagdanski AM; Alanio A; Molina JM | 2016 | Excluded | Irrelevant, did not discuss the subject matter |
| 486 | [Intestinal cryptococcosis: an unusual presentation of disseminated cryptococcosis]. | Hajoui FZ; Ghfir B; Moustachi A; Lyagoubi M; Aoufi S | 2014 | Excluded | Irrelevant, did not discuss the subject matter |
| 487 | Comment on "cryptococcal antigenemia among severely immunosuppressed HIV-infected adults in Uganda (Oyella et al. 2012)". | Joob B; Wiwanitkit V | 2012 | Excluded | Irrelevant, did not discuss the subject matter |
| 488 | Cryptococcus neoformans var. gattii meningitis in Egypt: a case report. | Mansour A; Nakhla I; El Sherif M; Sultan YA; Frenck RW | 2006 | Excluded | Irrelevant, did not discuss the subject matter |
| 489 | [Clear-fluid meningitis in HIV-infected patients in Dakar]. | Soumare M; Seydi M; Ndour CT; Dieng Y; Ngom-Faye NF; Fall N; Diop BM | 2005 | Excluded | Irrelevant, did not discuss the subject matter |
| 490 | Access to flucytosine for HIV-infected patients with cryptococcal meningitis â€“ an urgent need. | Govender NP; Meintjes G; Banoo S | 2014 | Excluded | Irrelevant, did not discuss the subject matter |
| 491 | Management of cryptoccocal meningitis in resource-limited settings: a systematic review. | Sloan D; Dlamini S; Dedicoat M | 2009 | Excluded | Irrelevant, did not discuss the subject matter |
| 492 | Idiopathic CD4+ T-lymphocytopenia with cryptococcal meningitis: first case report from Cambodia. | Augusto E; Raguenaud ME; Kim C; Mony M; Isaakidis P | 2009 | Excluded | Irrelevant, did not discuss the subject matter |
| 493 | [Epidemiological, clinical, etiological features of neuromeningeal diseases at the Fann Hospital Infectious Diseases Clinic, Dakar (Senegal)]. | SoumarÃ© M; Seydi M; Ndour CT; Fall N; Dieng Y; Sow AI; Diop BM | 2005 | Excluded | Irrelevant, did not discuss the subject matter |
| 494 | Cryptococcus gattii: A dilemma in diagnosis and treatment in sub-Saharan Africa an area with high HIV prevalence. | Robertson VJ; Nyazika TK; Katsidzira L; Gutu TI | 2013 | Excluded | Irrelevant, did not discuss the subject matter |
| 495 | [Neuromeningeal cryptococcosis in non-HIV patients to CHU ward of Point G in Bamako (Mali): 3 case report]. | Minta DK; DembÃ©lÃ© M; Diarra AS; SidibÃ© AT; KonatÃ© A; Diarra M; Coulibaly I; MaÃ¯ga II; TraorÃ© AK; MaÃ¯ga MY; Doumbo OK; TraorÃ© HA; Pichard E; Chabasse D | 2008 | Excluded | Irrelevant, did not discuss the subject matter |
| 496 | [Cryptococcal meningitis in children: description of 3 cases]. | Ndiaye M; Diagne NR; Seck LB; Sow AD; SÃ¨ne MS; Diop AG; Sow HD; Ndiaye MM | 2011 | Excluded | Irrelevant, did not discuss the subject matter |
| 497 | Cryptococcal antigen screening for patients initiating antiretroviral therapy: time for action. | Jarvis JN; Lawn SD; Wood R; Harrison TS | 2010 | Excluded | Irrelevant, did not discuss the subject matter |
| 498 | Meningitis in Jamaican adults. | Moonah SN; Bodonaik NC; Nicholson AM | 2006 | Excluded | Irrelevant, did not discuss the subject matter |
| 499 | Induced sputum for diagnosis of cryptococcal pneumonia in Botswana. | Morse M; Kessler J; Albrecht S; Kim R; Thakur R; Nthobatsang R; Radisowa K; Maunatlala C; Macgregor RR; Friedman H | 2009 | Excluded | Irrelevant, did not discuss the subject matter |
| 500 | [Cryptococcus neoformans meningoencephalitis in immunocompetent schoolchildren]. | Guevara-Campos J; GonzÃ¡lez-Guevara L; UrbÃ©z-Cano J; FermÃ­n S | 2009 | Excluded | Irrelevant, did not discuss the subject matter |
| 501 | [Update on neuromeningeal cryptococcosis in Dakar]. | SoumarÃ© M; Seydi M; Ndour CT; Dieng Y; Diouf AM; Diop BM | 2005 | Excluded | Irrelevant, did not discuss the subject matter |
| 502 | Routine cryptococcal antigen screening for HIV-infected patients with low CD4+ T-lymphocyte counts--time to implement in South Africa? | Jarvis JN; Harrison TS; Govender N; Lawn SD; Longley N; Bicanic T; Maartens G; Venter F; Bekker LG; Wood R; Meintjes G | 2011 | Excluded | Irrelevant, did not discuss the subject matter |
| 503 | [Neuromeningeal cryptococcosis in patients apparently non-immunodeficient: report of 3 cases in Dakar, Senegal]. | Ndiaye M; SoumarÃ© M; Mapoure YN; Seydi M; SÃ¨ne-Diouf F; Ngom NF; SÃ¨ne MS; Sow AD; Diop AG; Sow PS; Ndiaye MM; Ndiaye IP | 2008 | Excluded | Irrelevant, did not discuss the subject matter |
| 504 | Response to comment on "prevalence and factors associated with cryptococcal antigenemia among severely immunosuppressed HIV-infected adults in Uganda (Oyella et al. 2012)". | Oyella J; Meya D; Bajunirwe F; Kamya MR | 2012 | Excluded | Irrelevant, did not discuss the subject matter |
| 505 | Distinct paradoxical inflammatory reaction followed by postantiretroviral therapy immune reconstitution syndrome in cryptococcal meningitis. | Gaillet A; Calin R; Imbert S; Ollivier M; Guillot H; Fekkar A; Pourcher V | 2018 | Excluded | Irrelevant, did not discuss the subject matter |
| 506 | [Two cases of cryptococcal meningitis revealed by an ischemic stroke]. | Kouame-Assouan AE; Cowppli-Bony P; Aka-Anghui Diarra E; Assi B; Doumbia M; Diallo L; Adjien KC; Akani E; Sonan T; Diagana M; Boa YE; Kouassi B | 2007 | Excluded | Irrelevant, did not discuss the subject matter |
| 507 | Should antiretroviral therapy be delayed for 10 weeks for patients treated with fluconazole for cryptococcal meningitis? | Bicanic T; Jarvis JN; Muzoora C; Harrison TS | 2010 | Excluded | Irrelevant, did not discuss the subject matter |
| 508 | Recent advances in AIDS-related cryptococcal meningitis treatment with an emphasis on resource limited settings. | Lofgren S; Abassi M; Rhein J; Boulware DR | 2017 | Excluded | Irrelevant, did not discuss the subject matter |
| 509 | Clinical and laboratory characteristics of hospitalised patients with neurological manifestations of HIV/AIDS at the Nairobi hospital. | Jowi JO; Mativo PM; Musoke SS | 2007 | Excluded | Irrelevant, did not discuss the subject matter |
| 510 | The prevalence and clinical course of HIV-associated pulmonary cryptococcosis in Uganda. | Deok-jong Yoo S; Worodria W; Davis JL; Cattamanchi A; den Boon S; Kyeyune R; Kisembo H; Huang L | 2010 | Excluded | Irrelevant, did not discuss the subject matter |
| 511 | Paradoxical immune reconstitution inflammatory syndrome associated with cryptococcal meningitis in China: a 5-year retrospective cohort study. | Yan S; Chen L; Wu W; Li Z; Fu Z; Zhang H; Xue J; Hu Y; Mou J; Fu C | 2015 | Excluded | Irrelevant, did not discuss the subject matter |
| 512 | It is not always Tuberculosis! A case of pulmonary cryptococcosis in an immunocompetent child in Uganda. | Nakatudde I; Kasirye P; Kiguli S; Musoke P | 2021 | Excluded | Irrelevant, did not discuss the subject matter |
| 513 | Evaluation of a rapid lateral flow immunoassay for the detection of cryptococcal antigen for the early diagnosis of cryptococcosis in HIV patients in Colombia. | EscandÃ³n P; Lizarazo J; Agudelo CI; Chiller T; CastaÃ±eda E | 2013 | Excluded | Irrelevant, did not discuss the subject matter |
| 514 | The use of ventriculoperitoneal shunts for uncontrollable intracranial hypertension in patients with HIV-associated cryptococcal meningitis with or without hydrocephalus. | Liu L; Zhang R; Tang Y; Lu H | 2014 | Excluded | Irrelevant, did not discuss the subject matter |
| 515 | Comparison of cryptococcal meningitis in HIV-negative patients with and without lung infections. | Yang M; Cheng L; Sun F; Liu F; Feng W; Yao P; Weng B; Xia P | 2020 | Excluded | Irrelevant, did not discuss the subject matter |
| 516 | Predictors of mortality in patients initiating antiretroviral therapy in Durban, South Africa. | Ojikutu BO; Zheng H; Walensky RP; Lu Z; Losina E; Giddy J; Freedberg KA | 2008 | Excluded | Irrelevant, did not discuss the subject matter |
| 517 | Cryptococcosis in Gauteng: implications for monitoring of HIV treatment programmes. | McCarthy KM; Cohen C; Schneider H; Gould SM; Brandt ME; Hajjeh RA | 2008 | Excluded | Irrelevant, did not discuss the subject matter |
| 518 | Comparison of one week with two week regimens of amphotericin B both followed by fluconazole in the treatment of cryptococcal meningitis among AIDS patients. | Tansuphaswadikul S; Maek-a-Nantawat W; Phonrat B; Boonpokbn L; Mctm AG; Pitisuttithum P | 2006 | Excluded | Irrelevant, did not discuss the subject matter |
| 519 | Estimated burden of fungal infections in Panama. | RodrÃ­guez-Vargas C; Alastruey-Izquierdo A; Denning DW; BelÃ©n AraÃºz A | 2024 | Excluded | Irrelevant, did not discuss the subject matter |
| 520 | Estimated Prevalence of Cryptococcus Antigenemia (CrAg) among HIV-Infected Adults with Advanced Immunosuppression in Namibia Justifies Routine Screening and Preemptive Treatment. | Sawadogo S; Makumbi B; Purfield A; Ndjavera C; Mutandi G; Maher A; Kaindjee-Tjituka F; Kaplan JE; Park BJ; Lowrance DW | 2016 | Excluded | Irrelevant, did not discuss the subject matter |
| 521 | Burden of serious fungal infections in Ukraine. | Osmanov A; Denning DW | 2015 | Excluded | Irrelevant, did not discuss the subject matter |
| 522 | Establishing a cost-per-result of laboratory-based, reflex Cryptococcal antigenaemia screening (CrAg) in HIV+ patients with CD4 counts less than 100 cells/Î¼l using a Lateral Flow Assay (LFA) at a typical busy CD4 laboratory in South Africa. | Cassim N; Schnippel K; Coetzee LM; Glencross DK | 2017 | Excluded | Irrelevant, did not discuss the subject matter |
| 523 | Clinical and epidemiological features of 123 cases of cryptococcosis in Mato Grosso do Sul, Brazil. | Lindenberg Ade S; Chang MR; Paniago AM; LazÃ©ra Mdos S; Moncada PM; Bonfim GF; Nogueira SA; Wanke B | 2008 | Excluded | Irrelevant, did not discuss the subject matter |
| 524 | Cryptococcus spp. and Cryptococcosis: focusing on the infection in Brazil. | do Carmo FN; de Camargo Fenley J; Garcia MT; Rossoni RD; Junqueira JC; de Barros PP; Scorzoni L | 2022 | Excluded | Irrelevant, did not discuss the subject matter |
| 525 | Cryptococcosis in AtlÃ¡ntico, Colombia: an approximation of the prevalence of this mycosis and the distribution of the etiological agent in the environment. | Noguera MC; EscandÃ³n P; CastaÃ±eda E | 2015 | Excluded | Irrelevant, did not discuss the subject matter |
| 526 | Characterization of human immunodeficiency virus (HIV)-infected cells in infiltrates associated with CNS opportunistic infections in patients with HIV clade C infection. | Mahadevan A; Shankar SK; Satishchandra P; Ranga U; Chickabasaviah YT; Santosh V; Vasanthapuram R; Pardo CA; Nath A; Zink MC | 2007 | Excluded | Irrelevant, did not discuss the subject matter |
| 527 | High-dose amphotericin B with flucytosine for the treatment of cryptococcal meningitis in HIV-infected patients: a randomized trial. | Bicanic T; Wood R; Meintjes G; Rebe K; Brouwer A; Loyse A; Bekker LG; Jaffar S; Harrison T | 2008 | Excluded | Irrelevant, did not discuss the subject matter |
| 528 | Detection of Cryptococcus neoformans capsular antigen in HIV-infected patients in the state of Para in the north of Brazil. | Costa MM; Madeira Ld; Feitosa RN; Ishak Mde O; Ishak R; Silva SH; Vallinoto AC | 2013 | Excluded | Irrelevant, did not discuss the subject matter |
| 529 | Cryptococcosis-IRIS is associated with lower cryptococcus-specific IFN-Î³ responses before antiretroviral therapy but not higher T-cell responses during therapy. | Chang CC; Lim A; Omarjee S; Levitz SM; Gosnell BI; Spelman T; Elliott JH; Carr WH; Moosa MY; Ndung'u T; Lewin SR; French MA | 2013 | Excluded | Irrelevant, did not discuss the subject matter |
| 530 | Association of FcÎ³ receptor IIB polymorphism with cryptococcal meningitis in HIV-uninfected Chinese patients. | Hu XP; Wu JQ; Zhu LP; Wang X; Xu B; Wang RY; Ou XT; Weng XH | 2012 | Excluded | Irrelevant, did not discuss the subject matter |
| 531 | Neurological complications of human immunodeficiency virus infection in Zimbabwe-2005. | Mielke J | 2005 | Excluded | Irrelevant, did not discuss the subject matter |
| 532 | Single-Dose Liposomal Amphotericin B Treatment for Cryptococcal Meningitis. | Jarvis JN; Lawrence DS; Meya DB; Kagimu E; Kasibante J; Mpoza E; Rutakingirwa MK; Ssebambulidde K; Tugume L; Rhein J; Boulware DR; Mwandumba HC; Moyo M; Mzinganjira H; Kanyama C; Hosseinipour MC; Chawinga C; Meintjes G; Schutz C; Comins K; Singh A; Muzoora C; Jjunju S; Nuwagira E; Mosepele M; Leeme T; Siamisang K; Ndhlovu CE; Hlupeni A; Mutata C; van Widenfelt E; Chen T; Wang D; Hope W; Boyer-Chammard T; Loyse A; Molloy SF; Youssouf N; Lortholary O; Lalloo DG; Jaffar S; Harrison TS | 2022 | Excluded | Irrelevant, did not discuss the subject matter |
| 533 | Clinical insights and epidemiology of central nervous system infection due to Cryptococcus neoformans/gattii species complexes: A prospective study from South India. | Lahiri S; Manjunath N; Bhat M; Hagen F; Bahubali VH; Palaniappan M; Maji S; Chandrashekar N | 2020 | Excluded | Irrelevant, did not discuss the subject matter |
| 534 | [Efficiency of ethanol of Thonningia sanguinea against Cryptococcus neoformans]. | Ouattara B; Kra AM; Coulibaly A; Guede-Guina F | 2007 | Excluded | Irrelevant, did not discuss the subject matter |
| 535 | Pharmacokinetics-pharmacodynamics of sertraline as an antifungal in HIV-infected Ugandans with cryptococcal meningitis. | Alhadab AA; Rhein J; Tugume L; Musubire A; Williams DA; Abassi M; Nicol MR; Meya DB; Boulware DR; Brundage RC | 2019 | Excluded | Irrelevant, did not discuss the subject matter |
| 536 | Characteristics, mortality, associated variables with death, and therapeutic response among HIV-positive, solid organ transplant (SOT), and non-HIV-positive/non-transplant (NHNT) patients with cryptococcosis: First multicenter cohort study in Brazil. | Bridi Cavassin F; Vidal JE; BaÃº-Carneiro JL; Silva de Miranda Godoy C; de Bastos AscenÃ§o Soares R; Magri MMC; Falci DR; Sakuma De Oliveira C; Verena Almeida Mendes A; Breda GL; Rego CM; Araujo FÃ©lix M; Pacheco Katopodis P; da Silva do Ã“ JR; Pereira Lima AbrÃ£o M; Taborda M; Teles Teixeira Pereira T; Queiroz-Telles F | 2023 | Excluded | Irrelevant, did not discuss the subject matter |
| 537 | [Meningeal cryptococcosis in patients non infected with HIV: presentation of two cases and review of the literature]. | RamÃ­rez-Ramos C; Galindo J; Correa S; Giraldo-Bahamon G; Rivera J; Solano J; Peralta M; GonzÃ¡lez-Manrique G | 2018 | Excluded | Irrelevant, did not discuss the subject matter |
| 538 | [Proctocolitis by cryptococcus in an immunocompetent patient: first report in Peru]. | Medina Alvarez J; Tanta Chipana C; ArÃ©valo Suarez F | 2019 | Excluded | Irrelevant, did not discuss the subject matter |
| 539 | Characteristics and prognostic risk factors of patients with sequence type 5 lineage-associated cryptococcosis in China. | Tian Y; Wang J; Shen Y; Zhao J; Hu J; Zhu X; Zhu M; Guan M | 2023 | Excluded | Irrelevant, did not discuss the subject matter |
| 540 | Disseminated infection with Cryptococcus neoformans var neoformans in an 8 years immunocompetent girl. | Chaudhary MW; Sardana K; Kumar P; Dewan V; Anand VK | 2005 | Excluded | Irrelevant, did not discuss the subject matter |
| 541 | Cryptococcosis in Colombia: results of the national surveillance program for the years 2006-2010. | EscandÃ³n P; de Bedout C; Lizarazo J; Agudelo CI; TobÃ³n A; Bello S; Restrepo A; CastaÃ±eda E | 2012 | Excluded | Irrelevant, did not discuss the subject matter |
| 542 | Fluconazole and amphotericin B susceptibility testing of Cryptococcus neoformans: results of minimal inhibitory concentrations against 265 isolates from HIV-positive patients before and after two or more months of antifungal therapy. | Arechavala AI; Ochiuzzi ME; Borgnia MD; Santiso GM | 2009 | Excluded | Irrelevant, did not discuss the subject matter |
| 543 | Prevalence, predictors, and management of advanced HIV disease among individuals initiating ART in Senegal, West Africa. | Benzekri NA; Sambou JF; Ndong S; Tamba IT; Faye D; Diallo MB; Diatta JP; Faye K; Sall I; Sall F; Manga NM; Malomar JJ; Ndour CT; Hawes SE; Seydi M; Gottlieb GS | 2019 | Excluded | Irrelevant, did not discuss the subject matter |
| 544 | Antifungal susceptibilities of Cryptococcus neoformans cerebrospinal fluid isolates from AIDS patients in Kenya. | Mdodo R; Moser SA; Jaoko W; Baddley J; Pappas P; Kempf MC; Aban I; Odera S; Jolly P | 2011 | Excluded | Irrelevant, did not discuss the subject matter |
| 545 | High dose fluconazole in salvage therapy for HIV-uninfected cryptococcal meningitis. | Zhao HZ; Wang RY; Wang X; Jiang YK; Zhou LH; Cheng JH; Huang LP; Harrison TS; Zhu LP | 2018 | Excluded | Irrelevant, did not discuss the subject matter |
| 546 | Equity in clinical trials for HIV-associated cryptococcal meningitis: A systematic review of global representation and inclusion of patients and researchers. | Lawrence DS; Leeme T; Mosepele M; Harrison TS; Seeley J; Jarvis JN | 2021 | Excluded | Irrelevant, did not discuss the subject matter |
| 547 | Comparative performance of the laboratory assays used by a Diagnostic Laboratory Hub for opportunistic infections in people living with HIV. | Medina N; Alastruey-Izquierdo A; Mercado D; Bonilla O; PÃ©rez JC; Aguirre L; Samayoa B; Arathoon E; Denning DW; Rodriguez-Tudela JL | 2020 | Excluded | Irrelevant, did not discuss the subject matter |
| 548 | Underlying Cryptococcal Diseases and the Correlation With Serum Cryptococcal Antigen Titers in Hospitalized HIV-Infected Patients Screened Positive for Cryptococcal Antigenemia. | Xu M; Peng Z; Xu C; Chen Y; Cheng J; Chi Y; Wei H; Chen W; Hu Z | 2020 | Excluded | Irrelevant, did not discuss the subject matter |
| 549 | Immune reconstitution inflammatory syndrome (IRIS) associated with Cryptococcus neoformans infection in AIDS patients. | da Cunha Colombo ER; Mora DJ; Silva-Vergara ML | 2011 | Excluded | Irrelevant, did not discuss the subject matter |
| 550 | Cryptococcosis in human immunodeficiency virus-negative patients. | Kiertiburanakul S; Wirojtananugoon S; Pracharktam R; Sungkanuparph S | 2006 | Excluded | Irrelevant, did not discuss the subject matter |
| 551 | First report of cryptococcosis due to Cryptococcus gattii sensu stricto VGI in an Ivorian HIV negative patient. | Bellet V; Kassi FK; Krasteva D; Roger F; Drakulovski P; Mossou C; Kouakou GA; Doumbia A; Delaporte E; Menan H; Bertout S | 2021 | Excluded | Irrelevant, did not discuss the subject matter |
| 552 | Clinical and mycological predictors of cryptococcosis-associated immune reconstitution inflammatory syndrome. | Chang CC; Dorasamy AA; Gosnell BI; Elliott JH; Spelman T; Omarjee S; Naranbhai V; Coovadia Y; Ndung'u T; Moosa MY; Lewin SR; French MA | 2013 | Excluded | Irrelevant, did not discuss the subject matter |
| 553 | Screening for invasive fungal disease using non-culture-based assays among inpatients with advanced HIV disease at a large academic hospital in South Africa. | van Schalkwyk E; Mhlanga M; Maphanga TG; Mpembe RS; Shillubane A; Iyaloo S; Tsotetsi E; Pieton K; Karstaedt AS; Sahid F; Menezes CN; Tsitsi M; Motau A; Wadula J; Seetharam S; van den Berg E; Sriruttan C; Govender NP | 2020 | Excluded | Irrelevant, did not discuss the subject matter |
| 554 | Cryptococcal lymphadenitis-First presentation in an HIV-positive patient. | Ahuja S; Singh M | 2023 | Excluded | Irrelevant, did not discuss the subject matter |
| 555 | Pregnancy in HIV clinical trials in Sub Saharan Africa: failure of consent or contraception? | Ssali A; Namukwaya S; Bufumbo L; Seeley J; G Lalloo D; Kamali A; Parkes-Ratanshi R | 2013 | Excluded | Irrelevant, did not discuss the subject matter |
| 556 | AIDS-associated central nervous system cryptococcosis: a Brazilian case study. | Pappalardo MC; Paschoal RC; Melhem MS | 2007 | Excluded | Irrelevant, did not discuss the subject matter |
| 557 | Genotypic diversity and antifungal susceptibility of Cryptococcus neoformans species complex from China, including the diploid VNIII isolates from HIV-infected patients in Chongqing region. | Zhang L; Wang S; Hong N; Li M; Liu Y; Zhou T; Peng Y; Hu C; Li X; Zhang Z; Guo M; Cogliati M; Hitchcock M; Xu J; Chen M; Liao G | 2023 | Excluded | Irrelevant, did not discuss the subject matter |
| 558 | Molecular type distribution and fluconazole susceptibility of clinical Cryptococcus gattii isolates from South African laboratory-based surveillance, 2005-2013. | Naicker SD; Firacative C; van Schalkwyk E; Maphanga TG; Monroy-Nieto J; Bowers JR; Engelthaler DM; Meyer W; Govender NP | 2022 | Excluded | Irrelevant, did not discuss the subject matter |
| 559 | Multiple pathogens contribute to human immunodeficiency virus-related sepsis in addition to Mycobacterium tuberculosis: A prospective cohort in Tanzania. | Tsere DB; Shirima GM; Grundy BS; Heysell SK; Mpagama SG; Mziray SR; Mbelele PM | 2022 | Excluded | Irrelevant, did not discuss the subject matter |
| 560 | Molecular epidemiology reveals genetic diversity amongst isolates of the Cryptococcus neoformans/C. gattii species complex in Thailand. | Kaocharoen S; Ngamskulrungroj P; Firacative C; Trilles L; Piyabongkarn D; Banlunara W; Poonwan N; Chaiprasert A; Meyer W; Chindamporn A | 2013 | Excluded | Irrelevant, did not discuss the subject matter |
| 561 | High Prevalence of HIV-Related Cryptococcosis and Increased Resistance to Fluconazole of the Cryptococcus neoformans Complex in Jiangxi Province, South Central China. | Yang C; Bian Z; Blechert O; Deng F; Chen H; Li Y; Yang Y; Chen M; Zhan P | 2021 | Excluded | Irrelevant, did not discuss the subject matter |
| 562 | Implementation of the advanced HIV disease care package with point-of-care CD4 testing during tuberculosis case finding: A mixed-methods evaluation. | Gils T; Kamele M; Madonsela T; Bosman S; Ngubane T; Joseph P; Reither K; Bresser M; Vlieghe E; Decroo T; Ayakaka I; Lynen L; Van Heerden A | 2023 | Excluded | Irrelevant, did not discuss the subject matter |
| 563 | [Cryptococosis: clinical epidemiological laboratorial study and fungi varieties in 96 patients]. | Moreira Tde A; Ferreira MS; Ribas RM; Borges AS | 2006 | Excluded | Irrelevant, did not discuss the subject matter |
| 564 | Autopsy Prevalence of Tuberculosis and Other Potentially Treatable Infections among Adults with Advanced HIV Enrolled in Out-Patient Care in South Africa. | Karat AS; Omar T; von Gottberg A; Tlali M; Chihota VN; Churchyard GJ; Fielding KL; Johnson S; Martinson NA; McCarthy K; Wolter N; Wong EB; Charalambous S; Grant AD | 2016 | Excluded | Irrelevant, did not discuss the subject matter |
| 565 | Diversity and Antifungal Drug Susceptibility of Cryptococcus Isolates in Thailand. | Worasilchai N; Tangwattanachuleeporn M; Meesilpavikkai K; Folba C; Kangogo M; GroÃŸ U; Weig M; Bader O; Chindamporn A | 2017 | Excluded | Irrelevant, did not discuss the subject matter |
| 566 | Epidemiology and Mortality of Cryptococcal Disease in Guatemala: Two-Year Results of a Cryptococcal Antigen Screening Program. | Medina N; Rodriguez-Tudela JL; PÃ©rez JC; Mercado D; Bonilla O; Arathoon E; Alastruey-Izquierdo A | 2022 | Excluded | Irrelevant, did not discuss the subject matter |
| 567 | A mistaken diagnosis of Molluscum contagiosum in a HIV-positive patient in rural South Africa. | Sornum A | 2012 | Excluded | Irrelevant, did not discuss the subject matter |
| 568 | Feasibility of implementing the advanced HIV disease care package as part of community-based HIV/TB activities: a mixed-methods study protocol. | Gils T; Lynen L; Muhairwe J; Mashaete K; Lejone TI; Joseph P; Ngubane T; Keter AK; Reither K; van Heerden A | 2022 | Excluded | Irrelevant, did not discuss the subject matter |
| 569 | Clinical diversity of invasive cryptococcosis in AIDS patients from central China: report of two cases with review of literature. | Zhang Y; Cooper B; Gui X; Sherer R; Cao Q | 2019 | Excluded | Irrelevant, did not discuss the subject matter |
| 570 | Dectin-2 polymorphism associated with pulmonary cryptococcosis in HIV-uninfected Chinese patients. | Hu XP; Wang RY; Wang X; Cao YH; Chen YQ; Zhao HZ; Wu JQ; Weng XH; Gao XH; Sun RH; Zhu LP | 2015 | Excluded | Irrelevant, did not discuss the subject matter |
| 571 | Glial alterations in tuberculous and cryptococcal meningitis and their relation to HIV co-infection--a study on human brains. | Tripathi S; Patro I; Mahadevan A; Patro N; Phillip M; Shankar SK | 2014 | Excluded | Irrelevant, did not discuss the subject matter |
| 572 | Examination of a Chinese-made cryptococcal glucuronoxylomannan antigen test in serum and bronchoalveolar lavage fluid for diagnosing pulmonary cryptococcosis in HIV-negative patients. | Li Z; Wang M; Zeng P; Chen Z; Zhan Y; Li S; Lin Y; Cheng J; Ye F | 2022 | Excluded | Irrelevant, did not discuss the subject matter |
| 573 | Improved detection and management of advanced HIV disease through a community adult TB-contact tracing intervention with same-day provision of the WHO-recommended package of care including ART initiation in a rural district of Mozambique. | Izco S; Murias-Closas A; Jordan AM; Greene G; Catorze N; Chiconela H; Garcia JI; Blanco-Arevalo A; Febrer A; Casellas A; Saavedra B; Chiller T; Nhampossa T; Garcia-Basteiro A; Letang E | 2021 | Excluded | Irrelevant, did not discuss the subject matter |
| 574 | Cryptococcal meningitis treatment strategies in resource-limited settings: a cost-effectiveness analysis. | Rajasingham R; Rolfes MA; Birkenkamp KE; Meya DB; Boulware DR | 2012 | Excluded | Irrelevant, did not discuss the subject matter |
| 575 | Cryptococcus neoformans strains and infection in apparently immunocompetent patients, China. | Chen J; Varma A; Diaz MR; Litvintseva AP; Wollenberg KK; Kwon-Chung KJ | 2008 | Excluded | Irrelevant, did not discuss the subject matter |
| 576 | [Central Neurological Diagnosis in Patients Infected with HIV in the Infectious Diseases Unit of University Hospital of Casablanca, Morocco]. | El Fane M; Sodqi M; Lamdini H; Marih L; Lahsen AO; Chakib A; El Filali KM | 2018 | Excluded | Irrelevant, did not discuss the subject matter |
| 577 | Minimally Invasive Tissue Sampling: A Tool to Guide Efforts to Reduce AIDS-Related Mortality in Resource-Limited Settings. | Letang E; Rakislova N; Martinez MJ; Carlos Hurtado J; Carrilho C; Bene R; Mandomando I; QuintÃ³ L; Nhampossa T; Chicamba V; Luis E; Ismail MR; Fernandes F; Lorenzoni C; Ferreira L; Freire M; Teresa Rodrigo-Calvo M; Guerrero J; Munguambe K; Maixenchs M; Navarro M; Casas I; Marimon L; Ferrando M; Macete E; Lacerda M; Bassat Q; MenÃ©ndez C; Ordi J | 2021 | Excluded | Irrelevant, did not discuss the subject matter |
| 578 | The estimated burden of fungal diseases in Mali. | Doumbo SN; Cissoko Y; Dama S; Niangaly A; Garango A; KonatÃ© A; KonÃ© A; TraorÃ© B; Thera M; Djimde A; Denning DW | 2023 | Excluded | Irrelevant, did not discuss the subject matter |
| 579 | [A case of cryptococcal meningitis with nephrotic syndrome and renal insufficiency under immunosuppressive therapy]. | Tsuchida H; Ichikawa D; Shima Y; Yasuda T; Sato T; Kimura K | 2007 | Excluded | Irrelevant, did not discuss the subject matter |
| 580 | Isolation of cryptococcus, Candida, Aspergillus, Rhodotorula and nocardia from meningitis patients in egypt. | Elias ML; Soliman AK; Mahoney FJ; Karam El-Din AZ; El-Kebbi RA; Ismail TF; Wasfy MM; Mansour AM; Sultan YA; Pimentel G; Earhart KC | 2009 | Excluded | Irrelevant, did not discuss the subject matter |
| 581 | [Systemic mycosis: factors associated with death among patients infected with the human immunodeficiency virus, CuiabÃ¡, State of Mato Grosso, Brazil, 2005-2008]. | Ribeiro LC; Hahn RC; Favalessa OC; Tadano T; Fontes CJ | 2009 | Excluded | Irrelevant, did not discuss the subject matter |
| 582 | Treatment of cryptococcal meningitis in Peruvian AIDS patients using amphotericin B and fluconazole. | Dammert P; Bustamante B; Ticona E; Llanos-Cuentas A; Huaroto L; ChÃ¡vez VM; Campos PE | 2008 | Excluded | Irrelevant, did not discuss the subject matter |
| 583 | [Mycoses at Hospital Universitario "Ruiz y PÃ¡ez", Ciudad BolÃ­var, Venezuela, 2002]. | CermeÃ±o JR; HernÃ¡ndez I; Godoy G; Cabello I; CermeÃ±o JJ; OrellÃ¡n Y; Blanco Y | 2005 | Excluded | Irrelevant, did not discuss the subject matter |
| 584 | Disseminated Histoplasma capsulatum and Cryptococcus neoformans co-infection in patients with AIDS. | Aronis ML; dos Santos RP; Goldani LZ | 2011 | Excluded | Irrelevant, did not discuss the subject matter |
| 585 | Pathways to care with HIV-associated cryptococcal meningitis in Botswana and Uganda: Findings from a qualitative methods study. | Lawrence DS; Ssali A; Moshashane N; Nabaggala G; Maphane L; Harrison TS; Meya D; Jarvis JN; Seeley J | 2023 | Excluded | Irrelevant, did not discuss the subject matter |
| 586 | Tuberculosis and the risk of opportunistic infections and cancers in HIV-infected patients starting ART in Southern Africa. | Fenner L; Reid SE; Fox MP; Garone D; Wellington M; Prozesky H; Zwahlen M; Schomaker M; Wandeler G; Kancheya N; Boulle A; Wood R; Henostroza G; Egger M | 2013 | Excluded | Irrelevant, did not discuss the subject matter |
| 587 | The aetiology, clinical presentations and outcome of febrile encephalopathy in children in Papua New Guinea. | Anga G; Barnabas R; Kaminiel O; Tefuarani N; Vince J; Ripa P; Riddell M; Duke T | 2010 | Excluded | Irrelevant, did not discuss the subject matter |
| 588 | Tuberculosis is the leading cause of lymphadenopathy in HIV-infected persons in India: results of a fine-needle aspiration analysis. | Kamana NK; Wanchu A; Sachdeva RK; Kalra N; Rajawanshi A | 2010 | Excluded | Irrelevant, did not discuss the subject matter |
| 589 | Cryptococcosis by Cryptococcus neoformans/Cryptococcus gattii Species Complexes in non-HIV-Infected Patients in Southeastern Brazil. | Nascimento E; BariÃ£o PHG; Kress MRVZ; Vilar FC; Santana RC; Gaspar GG; Martinez R | 2021 | Excluded | Irrelevant, did not discuss the subject matter |
| 590 | Immune reconstitution inflammatory syndrome in a patient with cryptococcal lymphadenitis as the first presentation of acquired immunodeficiency syndrome. | Tahir M; Sharma SK; Sinha S; Das CJ | 2007 | Excluded | Irrelevant, did not discuss the subject matter |
| 591 | Clinical, epidemiological and outcome features of patients with cryptococcosis in Uberaba, Minas Gerais, Brazil. | Mora DJ; da Cunha Colombo ER; Ferreira-Paim K; Andrade-Silva LE; Nascentes GA; Silva-Vergara ML | 2012 | Excluded | Irrelevant, did not discuss the subject matter |
| 592 | Opportunistic infection among HIV seropositive cases in Manipal Teaching Hospital, Pokhara, Nepal. | Dhungel BA; Dhungel KU; Easow JM; Singh YI | 2008 | Excluded | Irrelevant, did not discuss the subject matter |
| 593 | Primary bilateral multifocal choroiditis as an initial manifestation of disseminated cryptococcosis in a HIV-positive patient. | Babu K; Murthy KR; Rajagopalan N | 2008 | Excluded | Irrelevant, did not discuss the subject matter |
| 594 | Cryptococcus and cryptococcosis in Iran during 1969-2019: A systematic review and meta-analysis. | Bandalizadeh Z; Javidnia J; Hosseini SA; Moosazadeh M; Amouei A; Kermani F; Seyedmousavi S; Shokohi T | 2020 | Excluded | Irrelevant, did not discuss the subject matter |
| 595 | Multiple ring enhancing brain lesions on computed tomography: an Indian perspective. | Garg RK; Desai P; Kar M; Kar AM | 2008 | Excluded | Irrelevant, did not discuss the subject matter |
| 596 | Burden of acute kidney injury in HIV patients under deoxycholate amphotericin B therapy for cryptococcal meningitis and cost-minimization analysis of amphotericin B lipid complex. | Tuon FF; Florencio KL; Rocha JL | 2019 | Excluded | Irrelevant, did not discuss the subject matter |
| 597 | Responding to the evidence for improved treatment for cryptococcal meningitis in resource-limited settings. | Milani B; Ford N | 2012 | Excluded | Irrelevant, did not discuss the subject matter |
| 598 | Establishing targets for advanced HIV disease: A call to action. | Meya DB; Tugume L; Nabitaka V; Namuwenge P; Phiri S; Oladele R; Jibrin B; Mobolaji-Bello M; Kanyama C; Maokola W; Mfinanga S; Katureebe C; Amamilo I; Ngwatu B; Jarvis JN; Harrison TS; Shroufi A; Rajasingham R; Boulware D; Govender NP; Loyse A | 2021 | Excluded | Irrelevant, did not discuss the subject matter |
| 599 | Current scenario of cryptococcosis and antifungal susceptibility pattern in India: a cause for reappraisal. | Capoor MR; Mandal P; Deb M; Aggarwal P; Banerjee U | 2008 | Excluded | Irrelevant, did not discuss the subject matter |
| 600 | Epidemiological, Clinical and Outcome Aspects of Patients with Cryptococcosis Caused by Cryptococcus gattii from a Non-endemic Area of Brazil. | Damasceno-Escoura AH; de Souza ML; de Oliveira Nunes F; Pardi TC; Gazotto FC; Florentino DH; Mora DJ; Silva-Vergara ML | 2019 | Excluded | Irrelevant, did not discuss the subject matter |
| 601 | Cryptococcosis in the Amazon: A current overview and future perspectives. | CorrÃªa Pinheiro M; Dos Reis DST; de Brito MTFM; SimÃµes Quaresma JA | 2019 | Excluded | Irrelevant, did not discuss the subject matter |
| 602 | Poor specificity of urinary cryptococcal antigen testing: Reply to Drain etÂ al. Prevalence of cryptococcal antigenuria at initial HIV diagnosis in KwaZulu-Natal. | Tenforde MW; Longley N; Meya DB; Boulware DR; Meintjes G; Goercke I; Harrison TS; Jarvis JN | 2018 | Excluded | Irrelevant, did not discuss the subject matter |
| 603 | Molecular types of Cryptococcus gattii/Cryptococcus neoformans species complex from clinical and environmental sources in Nairobi, Kenya. | Kangogo M; Bader O; Boga H; Wanyoike W; Folba C; Worasilchai N; Weig M; GroÃŸ U; Bii CC | 2015 | Excluded | Irrelevant, did not discuss the subject matter |
| 604 | Detection of Cryptococcus by conventional, serological and molecular methods. | Saha DC; Xess I; Biswas A; Bhowmik DM; Padma MV | 2009 | Excluded | Irrelevant, did not discuss the subject matter |
| 605 | Correlation of antifungal susceptibility and sequence types within Cryptococcus neoformans VNI from HIV patients, and ERG11 gene polymorphism. | Bive BZ; Sacheli R; Mudogo CN; Zakayi PK; Bontems S; Lelo GM; Hayette MP | 2023 | Excluded | Irrelevant, did not discuss the subject matter |
| 606 | Evaluation of a point-of-care immunoassay test kit 'StrongStep' for cryptococcal antigen detection. | Mpoza E; Mukaremera L; Kundura DA; Akampurira A; Luggya T; Tadeo KK; Pastick KA; Bridge SC; Tugume L; Kiggundu R; Musubire AK; Williams DA; Muzoora C; Nalintya E; Rajasingham R; Rhein J; Boulware DR; Meya DB; Abassi M | 2018 | Excluded | Irrelevant, did not discuss the subject matter |
| 607 | Cryptococcosis in China (1985-2010): review of cases from Chinese database. | Yuchong C; Fubin C; Jianghan C; Fenglian W; Nan X; Minghui Y; Yalin S; Zhizhong Z | 2012 | Excluded | Irrelevant, did not discuss the subject matter |
| 608 | Implementing Advanced HIV Disease Care for Inpatients in a Referral Hospital in Malawi - Demand, Results and Cost Implications. | Heller T; Damba D; Kumwenda T; Huwa J; Kamamia C; Nhlema A; Wallrauch C; Chawinga C; Kanyama C; Gondwe-Chunda L; Ngoma J; Matanje B; Tweya H | 2022 | Excluded | Irrelevant, did not discuss the subject matter |
| 609 | Clinical characteristics, Cryptococcus neoformans genotypes, antifungal susceptibility, and outcomes in human immunodeficiency virus-positive patients in Beijing, China. | Xu X; Du P; Wang H; Yang X; Liu T; Zhang Y; Wang Y | 2021 | Excluded | Irrelevant, did not discuss the subject matter |
| 610 | Disseminated cryptococcosis in a human immunodeficiency virus-negative patient: a case report. | Kokturk N; Ekim N; Kervan F; Arman D; Memis L; Caglar K; Kalkanci A; Demircan S; Kurul C; Akyurek N | 2005 | Excluded | Irrelevant, did not discuss the subject matter |
| 611 | Retrospective study of the epidemiology and clinical manifestations of Cryptococcus gattii infections in Colombia from 1997-2011. | Lizarazo J; EscandÃ³n P; Agudelo CI; Firacative C; Meyer W; CastaÃ±eda E | 2014 | Excluded | Irrelevant, did not discuss the subject matter |
| 612 | Association of neurotropic viruses in HIV-infected individuals who died of secondary complications of tuberculosis, cryptococcosis, or toxoplasmosis in South India. | Kannangai R; Sachithanandham J; Mahadevan A; Abraham AM; Sridharan G; Desai A; Ravi V; Shankar SK | 2013 | Excluded | Irrelevant, did not discuss the subject matter |
| 613 | Brief Report: Geographical Variation in Prevalence of Cryptococcal Antigenemia Among HIV-Infected, Treatment-Naive Patients in Nigeria: A Multicenter Cross-Sectional Study. | Ezeanolue EE; Nwizu C; Greene GS; Amusu O; Chukwuka C; Ndembi N; Smith RM; Chiller T; Pharr J; Kozel TR | 2016 | Excluded | Irrelevant, did not discuss the subject matter |
| 614 | Comparison of amphotericin B deoxycholate in combination with either flucytosine or fluconazole, and voriconazole plus flucytosine for the treatment of HIV-associated cryptococcal meningitis: a prospective multicenter study in China. | Zhao T; Xu X; Wu Y; Zhang W; Zeng Q; Lu Y; Yang T; Zhou G; Yu J; Lan K; Harypursat V; Chen Y | 2022 | Excluded | Irrelevant, did not discuss the subject matter |
| 615 | Molecular diagnosis of central nervous system opportunistic infections in HIV-infected Zambian adults. | Siddiqi OK; Ghebremichael M; Dang X; Atadzhanov M; Kaonga P; Khoury MN; Koralnik IJ | 2014 | Excluded | Irrelevant, did not discuss the subject matter |
| 616 | Cryptococcal gastroduodenitis: a rare location of the disease. | Girardin M; Greloz V; Hadengue A | 2010 | Excluded | Irrelevant, did not discuss the subject matter |
| 617 | FATAL DISSEMINATED CRYPTOCOCCOSIS WITH RENAL INVOLVEMENT IN AN HIV-INFECTED PATIENT. | Daher Ede F; Nasserala JC; Silva Junior GB; Oliveira AR; Medeiros Neto JU; Sousa AQ | 2015 | Excluded | Irrelevant, did not discuss the subject matter |
| 618 | Tuberculosis-associated hemophagocytic lymphohistiocytosis with subsequent unmasking cryptococcal immune reconstitution inflammatory syndrome (IRIS) in an HIV-negative man. | Geerdes-Fenge HF; LÃ¶bermann M; Hemmer CJ; Benedek O; Reisinger EC | 2019 | Excluded | Irrelevant, did not discuss the subject matter |
| 619 | Usual Presentation Of An Unusual Pathogen - Cryptococcus Laurentii Meningitis: A Case Report. | Zulfiqar A; Irfan R; Hanif F; Usman J | 2023 | Excluded | Irrelevant, did not discuss the subject matter |
| 620 | Compartmentalization of innate immune responses in the central nervous system during cryptococcal meningitis/HIV coinfection. | Naranbhai V; Chang CC; Durgiah R; Omarjee S; Lim A; Moosa MY; Elliot JH; Ndung'u T; Lewin SR; French MA; Carr WH | 2014 | Excluded | Irrelevant, did not discuss the subject matter |
| 621 | Prevalence of Cryptococcal Antigen and Outcomes in People With Human Immunodeficiency Virus in Honduras: A Cohort Study. | Zuniga-Moya JC; Romero-Reyes LE; Saavedra EB; Montoya S; Varela D; Borjas M; Cerna A; Bejarano S; Martinez P; Lujan K; Erazo K; Lainez I; Pineda L; Yanes D; O'Halloran JA; Spec A | 2021 | Excluded | Irrelevant, did not discuss the subject matter |
| 622 | Lymphnodal Co-infection of Cryptococcus and Histoplasma in a HIV-Infected Patient and Review of Published Reports. | Ghosh A; Tilak R; Bhushan R; Dhameja N; Chakravarty J | 2015 | Excluded | Irrelevant, did not discuss the subject matter |
| 623 | Postmortem findings in HIV/AIDS patients in a tertiary care hospital in rural South Africa. | Garcia-Jardon M; Bhat VG; Blanco-Blanco E; Stepian A | 2010 | Excluded | Irrelevant, did not discuss the subject matter |
| 624 | Primary endemic Cryptococcosis gattii by molecular type VGII in the state of ParÃ¡, Brazil. | Santos WR; Meyer W; Wanke B; Costa SP; Trilles L; Nascimento JL; Medeiros R; Morales BP; Bezerra Cde C; MacÃªdo RC; Ferreira SO; Barbosa GG; Perez MA; Nishikawa MM; LazÃ©ra Mdos S | 2008 | Excluded | Irrelevant, did not discuss the subject matter |
| 625 | Cytokine profiles at admission can be related to outcome in AIDS patients with cryptococcal meningitis. | Mora DJ; Fortunato LR; Andrade-Silva LE; Ferreira-Paim K; Rocha IH; Vasconcelos RR; Silva-Teixeira DN; Nascentes GA; Silva-Vergara ML | 2015 | Excluded | Irrelevant, did not discuss the subject matter |
| 626 | Seroprevalence of histoplasmosis in Kampala, Uganda. | Bahr NC; Sarosi GA; Meya DB; Bohjanen PR; Richer SM; Swartzentruber S; Halupnick R; Jarrett D; Wheat LJ; Boulware DR | 2016 | Excluded | Irrelevant, did not discuss the subject matter |
| 627 | Molecular epidemiology and antifungal susceptibilities of Cryptococcus species isolates from HIV and non-HIV patients in Southwest China. | Wu SY; Kang M; Liu Y; Chen ZX; Xiao YL; He C; Ma Y | 2021 | Excluded | Irrelevant, did not discuss the subject matter |
| 628 | Choroiditis in a HIV-infected patient with disseminated cryptococcal infection: A case report and literature review. | Heitor DF; Mora DJ; Damasceno-Escoura AH; Micheletti AMR; Garcia Torres R; Castro Gazotto F; Pardi TC; Rosa A; Silva-Vergara ML | 2019 | Excluded | Irrelevant, did not discuss the subject matter |
| 629 | Retrospective analysis of pulmonary cryptococcosis and extrapulmonary cryptococcosis in a chinese tertiary hospital. | Shi J; Chen J; Hu L; Ma AHY; Hu H; Wang C; Huang J; Song Q; Qian G | 2023 | Excluded | Irrelevant, did not discuss the subject matter |
| 630 | Comparison of genotypes between environmental and clinical isolates of Cryptococcus neoformans var. grubii based on microsatellite patterns. | Zhu J; Kang Y; Uno J; Taguchi H; Liu Y; Ohata M; Tanaka R; Moretti ML; Mikami Y | 2010 | Excluded | Irrelevant, did not discuss the subject matter |
| 631 | Postmortem findings and opportunistic infections in HIV-positive patients from a public hospital in Peru. | Eza D; Cerrillo G; Moore DA; Castro C; Ticona E; Morales D; Cabanillas J; Barrantes F; Alfaro A; Benavides A; Rafael A; Valladares G; Arevalo F; Evans CA; Gilman RH | 2006 | Excluded | Irrelevant, did not discuss the subject matter |
| 632 | Cryptococcosis in non-HIV/non-transplant patients: A Brazilian case series. | Lomes NR; Melhem MS; Szeszs MW; Martins Mdos A; Buccheri R | 2016 | Excluded | Irrelevant, did not discuss the subject matter |
| 633 | Clinical analysis of 76 patients pathologically diagnosed with pulmonary cryptococcosis. | Zhang Y; Li N; Li H; Chen X; Wang S; Zhang X; Zhang R; Xu J; Shi J; Yung RC | 2012 | Excluded | Irrelevant, did not discuss the subject matter |
| 634 | The epidemiology of cryptococcosis and the characterization of Cryptococcus neoformans isolated in a Brazilian University Hospital. | Aguiar PADF; Pedroso RDS; Borges AS; Moreira TA; AraÃºjo LB; RÃ¶der DVDB | 2017 | Excluded | Irrelevant, did not discuss the subject matter |
| 635 | Cryptococcus neoformans-reactive and total immunoglobulin profiles of human immunodeficiency virus-infected and uninfected Ugandans. | Subramaniam K; French N; Pirofski LA | 2005 | Excluded | Irrelevant, did not discuss the subject matter |
| 636 | Disseminated Cryptococcosis in an HIV-negative patient in South Africa: the elusive differential diagnosis. | Mitha M; Naicker P; Mahida P | 2010 | Excluded | Irrelevant, did not discuss the subject matter |
| 637 | Current situation of fungal diseases in Eritrea. | Werkneh S; Orefuwa E; Denning DW | 2022 | Excluded | Irrelevant, did not discuss the subject matter |
| 638 | Extracellular enzymatic activity and serotype of Cryptococcus neoformans strains isolated from AIDS patients in Brazil. | Vidotto V; Melhem M; Pukinskas S; Aoki S; Carrara C; Pugliese A | 2005 | Excluded | Irrelevant, did not discuss the subject matter |
| 639 | Disseminated cryptococcosis in a patient with newly diagnosed HTLV-1 infection. | Motoa G; Powers HR; Brumble LM | 2021 | Excluded | Irrelevant, did not discuss the subject matter |
| 640 | A diverse population of Cryptococcus gattii molecular type VGIII in southern Californian HIV/AIDS patients. | Byrnes EJ 3rd; Li W; Ren P; Lewit Y; Voelz K; Fraser JA; Dietrich FS; May RC; Chaturvedi S; Chaturvedi V; Heitman J | 2011 | Excluded | Irrelevant, did not discuss the subject matter |
| 641 | Molecular characterisation of the causative agents of Cryptococcosis in patients of a tertiary healthcare facility in the state of Amazonas-Brazil. | Freire AK; dos Santos Bentes A; de Lima Sampaio I; Matsuura AB; Ogusku MM; Salem JI; Wanke B; de Souza JV | 2012 | Excluded | Irrelevant, did not discuss the subject matter |
| 642 | Susceptibility of clinical isolates of Cryptococcus neoformans to amphotericin B using time-kill methodology. | Pappalardo MC; Szeszs MW; Martins MA; Baceti LB; Bonfietti LX; Purisco SU; Baez AA; Melhem MS | 2009 | Excluded | Irrelevant, did not discuss the subject matter |
| 643 | Donor-Derived Transmission of Cryptococcus gattii sensu lato in Kidney Transplant Recipients. | Santos DWCL; Hagen F; Meis JF; Cristelli MP; Viana LA; Bernardi FDC; Tedesco-Silva H; Medina-Pestana JO; Colombo AL | 2020 | Excluded | Irrelevant, did not discuss the subject matter |
| 644 | PTX3 gene polymorphism associated with cryptococcosis in HIV-uninfected Chinese patients. | Zhang W; Liao Q; Liu Y; Wu S; Deng J; Xiao Y; Ma Y; Xie Y; Kang M | 2021 | Excluded | Irrelevant, did not discuss the subject matter |
| 645 | Multi-locus sequence typing reveals genotypic similarity in Nigerian Cryptococcus neoformans AFLP1/VNI of environmental and clinical origin. | Chidebelu PE; Nweze EI; Meis JF; Cogliati M; Hagen F | 2021 | Excluded | Irrelevant, did not discuss the subject matter |
| 646 | Paciente inmunocompetente con criptococosis cerebral: reporte de un caso. | Becerra-Pedraza LC; MartÃ­nez-PiÃ±a DA; Calles-Carmona GR; San-Juan D | 2017 | Excluded | Irrelevant, did not discuss the subject matter |
| 647 | Burden of serious fungal infections in Honduras. | Agudelo Higuita NI; Varela Bustillo D; Denning DW | 2022 | Excluded | Irrelevant, did not discuss the subject matter |
| 648 | Anemia in human immunodeficiency virus-infected and uninfected women in Rwanda. | Masaisa F; Gahutu JB; Mukiibi J; Delanghe J; PhilippÃ© J | 2011 | Excluded | Irrelevant, did not discuss the subject matter |
| 649 | Estimated burden of serious fungal infections in Togo. | Dorkenoo AM; Adjetey-Toglozombio AK; Ocansey BK; Sossou E; Lack F; Denning DW | 2021 | Excluded | Irrelevant, did not discuss the subject matter |
| 650 | Burden of serious fungal infections in Tanzania. | Faini D; Maokola W; Furrer H; Hatz C; Battegay M; Tanner M; Denning DW; Letang E | 2015 | Excluded | Irrelevant, did not discuss the subject matter |
| 651 | Cryptococcal Antigen Screening in Asymptomatic HIV-Infected Antiretroviral NaÃ¯ve Patients in Cameroon and Evaluation of the New Semi-Quantitative Biosynex CryptoPS Test. | Temfack E; Kouanfack C; Mossiang L; Loyse A; Fonkoua MC; Molloy SF; Koulla-Shiro S; Delaporte E; Dromer F; Harrison T; Lortholary O | 2018 | Excluded | Irrelevant, did not discuss the subject matter |
| 652 | Estimated Burden of Serious Fungal Diseases in Serbia. | ArsenijeviÄ‡ VA; Denning DW | 2018 | Excluded | Irrelevant, did not discuss the subject matter |
| 653 | Molecular typing and in vitro antifungal susceptibility of Cryptococcus spp from patients in Midwest Brazil. | Favalessa OC; de Paula DA; Dutra V; Nakazato L; Tadano T; Lazera Mdos S; Wanke B; Trilles L; Walderez Szeszs M; Silva D; Hahn RC | 2014 | Excluded | Irrelevant, did not discuss the subject matter |
| 654 | Estimates of serious fungal infection burden in CÃ´te d'Ivoire and country health profile. | Koffi D; Bonouman IV; Toure AO; Kouadjo F; N'Gou MRE; Sylla K; Dosso M; Denning DW | 2021 | Excluded | Irrelevant, did not discuss the subject matter |
| 655 | Aetiology of hospitalized fever and risk of death at Arua and Mubende tertiary care hospitals in Uganda from August 2019 to August 2020. | Blair PW; Kobba K; Kakooza F; Robinson ML; Candia E; Mayito J; Ndawula EC; Kandathil AJ; Matovu A; Aniku G; Manabe YC; Lamorde M | 2022 | Excluded | Irrelevant, did not discuss the subject matter |
| 656 | An AIDS patient with urine retention. | Xu L; Tao R; Zhao Q; Cheng J; Zhu B | 2019 | Excluded | Irrelevant, did not discuss the subject matter |
| 657 | Invasive fungal infections among inpatients with acquired immune deficiency syndrome at a Chinese university hospital. | Shen YZ; Qi TK; Ma JX; Jiang XY; Wang JR; Xu QN; Huang Q; Liu XN; Sun HQ; Lu HZ | 2007 | Excluded | Irrelevant, did not discuss the subject matter |
| 658 | Cryptococcus gattii meningoencephalitis in an HIV-negative patient from the Peruvian Andes. | Gutierrez EL; Valqui W; Vilchez L; Evangelista L; Crispin S; Tello M; Navincopa M; BÃ©jar V; GonzÃ¡les J; Ortega-Loayza AG | 2010 | Excluded | Irrelevant, did not discuss the subject matter |
| 659 | Molecular epidemiology of Italian clinical Cryptococcus neoformans var. grubii isolates. | Cogliati M; Zamfirova RR; Tortorano AM; Viviani MA | 2013 | Excluded | Irrelevant, did not discuss the subject matter |
| 660 | A multicenter survey of asymptomatic cryptococcal antigenemia among patients with advanced HIV disease in Nigeria. | Oladele RO; Jordan AM; Okaa JU; Osaigbovo II; Shettima SA; Shehu NY; Davies AA; Mohammed Y; Alex-Wele MA; Iliyasu G; Nwaokenye JC; Fayemiwo SA; Udoh UA; Gbajabiamila T; Denning DW; Chiller TM | 2023 | Excluded | Irrelevant, did not discuss the subject matter |
| 661 | High prevalence of tuberculosis and serious bloodstream infections in ambulatory individuals presenting for antiretroviral therapy in Malawi. | Bedell RA; Anderson ST; van Lettow M; Akesson A; Corbett EL; Kumwenda M; Chan AK; Heyderman RS; Zachariah R; Harries AD; Ramsay AR | 2012 | Excluded | Irrelevant, did not discuss the subject matter |
| 662 | [Opportunistic diseases in HIV-infected patients at the Jeanne Ebori Foundation in Libreville, Gabon]. | Okome-Nkoumou M; Boguikouma JB; Kombila M | 2006 | Excluded | Irrelevant, did not discuss the subject matter |
| 663 | Opportunistic diseases in HIV-infected patients in Gabon following the administration of highly active antiretroviral therapy: a retrospective study. | Okome-Nkoumou M; Guiyedi V; Ondounda M; Efire N; Clevenbergh P; Dibo M; Dzeing-Ella A | 2014 | Excluded | Irrelevant, did not discuss the subject matter |
| 664 | Co-administration of fluconazole increases nevirapine concentrations in HIV-infected Ugandans. | Wakeham K; Parkes-Ratanshi R; Watson V; Ggayi AB; Khoo S; Lalloo DG | 2010 | Excluded | Irrelevant, did not discuss the subject matter |
| 665 | Clinical and microbiological characteristics of cryptococcosis at an university hospital in China from 2013 to 2017. | Fang LF; Zhang PP; Wang J; Yang Q; Qu TT | 2020 | Excluded | Irrelevant, did not discuss the subject matter |
| 666 | Cryptococcus neoformans septicemia in an immunocompetent neonate: first case report in Thailand. | Nakwan N; Ngerncham S; Srisuparp P; Lapphra K; Chokephaibulkit K | 2008 | Excluded | Irrelevant, did not discuss the subject matter |
| 667 | [A prospective multi-center clinical investigation of HIV-negative pulmonary cryptococcosis in China]. | Chen LA; She DY; Liang ZX; Liang LL; Chen RC; Ye F; Li YP; Zhou Y; Chen XH; Fang SF; Lai GX; Hu Q; Xie BS; Yao XJ; Shi Y; Su X; He LX; Zhou JY; Zhong SC; Zhang QL; Xiong SD; Qu JM; Tong ZH; Jiang SJ; Liu J; Xu F; He B; Li ER; Yuan YD; Zhang XY; Sun TY; Liu YN | 2021 | Excluded | Irrelevant, did not discuss the subject matter |
| 668 | Comparison of Etests and Vitek 2 Â® to broth microdilution for the susceptibility testing of Cryptococcus neoformans. | Mahabeer Y; Chang CC; Naidu D; Dorasamy A; Lewin S; Ndung'u T; Moosa MY; French M; Mlisana K; Coovadia Y | 2014 | Excluded | Irrelevant, did not discuss the subject matter |
| 669 | Serious fungal disease incidence and prevalence in Indonesia. | Wahyuningsih R; Adawiyah R; Sjam R; Prihartono J; Ayu Tri Wulandari E; Rozaliyani A; Ronny R; Imran D; Tugiran M; Siagian FE; Denning DW | 2021 | Excluded | Irrelevant, did not discuss the subject matter |
| 670 | Molecular epidemiology and antifungal susceptibility of Serbian Cryptococcus neoformans isolates. | Arsic Arsenijevic V; Pekmezovic MG; Meis JF; Hagen F | 2014 | Excluded | Irrelevant, did not discuss the subject matter |
| 671 | Central nervous system immune reconstitution inflammatory syndrome in AIDS: experience of a Mexican neurological centre. | Guevara-Silva EA; RamÃ­rez-Crescencio MA; Soto-HernÃ¡ndez JL; CÃ¡rdenas G | 2012 | Excluded | Irrelevant, did not discuss the subject matter |
| 672 | Fatal Cryptococcus gattii genotype AFLP6/VGII infection in a HIV-negative patient: case report and a literature review. | Favalessa OC; LÃ¡zera Mdos S; Wanke B; Trilles L; Takahara DT; Tadano T; Dias LB; Vieira AC; Novack GV; Hahn RC | 2014 | Excluded | Irrelevant, did not discuss the subject matter |
| 673 | Incidence and risk factors of major opportunistic infections after initiation of antiretroviral therapy among advanced HIV-infected patients in a resource-limited setting. | Manosuthi W; Chaovavanich A; Tansuphaswadikul S; Prasithsirikul W; Inthong Y; Chottanapund S; Sittibusaya C; Moolasart V; Termvises P; Sungkanuparph S | 2007 | Excluded | Irrelevant, did not discuss the subject matter |
| 674 | Neuro OIs: developed and developing countries. | Wright EJ | 2014 | Excluded | Irrelevant, did not discuss the subject matter |
| 675 | Immune reconstitution inflammatory syndrome among HIV/AIDS patients during highly active antiretroviral therapy in Addis Ababa, Ethiopia. | Huruy K; Mulu A; Mengistu G; Shewa-Amare A; Akalu A; Kassu A; Andargie G; Elias D; Torben W | 2008 | Excluded | Irrelevant, did not discuss the subject matter |
| 676 | Multilocus sequence typing of Cryptococcus neoformans in non-HIV associated cryptococcosis in Nagasaki, Japan. | Mihara T; Izumikawa K; Kakeya H; Ngamskulrungroj P; Umeyama T; Takazono T; Tashiro M; Nakamura S; Imamura Y; Miyazaki T; Ohno H; Yamamoto Y; Yanagihara K; Miyzaki Y; Kohno S | 2013 | Excluded | Irrelevant, did not discuss the subject matter |
| 677 | Proceedings of The 8th Romanian National HIV/AIDS Congress and The 3rd Central European HIV Forum : Sibiu, Romania. 5-7 May 2016. | Alexiev I; Dimitrova R; Gancheva A; Kostadinova A; Stoycheva M; Nikolova D; Elenkov I; TiliÈ™can C; Predescu M; PÄƒunescu B; Streinu-Cercel A; SÄƒndulescu O; È˜chiopu CM; Hristache M; BrÃ®nduÈ™e LA; Todorovic M; Siljic M; Salemovic D; Nikolic V; Pesic-Pavlovic I; Ranin J; Jevtovic D; Stanojevic M; Tudor AM; Vlad D; MÄƒrdÄƒrescu M; Petrea S; Petre C; Neagu-DrÄƒghicenoiu R; Ungurianu R; Cibea A; ChirilÄƒ O; Anghelina C; Coserea I; Krikelli PA; Pavlitina E; Psichogiou M; Lamnisos D; Williams L; Korobchuk A; Skaathun B; Smyrnov P; Schneider J; Sypsa V; Paraskevis D; Hatzakis A; Friedman SR; Nikolopoulos GK; DragoviÄ‡ G; SrdiÄ‡ D; Khawla AM; SoldatoviÄ‡ I; NikoliÄ‡ J; JevtoviÄ‡ D; Nair D; Temereanca A; Rosca A; Ene L; Soontornniyomkij B; Diaconu C; Dita C; Achim C; Ruta S; Benea È˜; Moroti R; Jipa R; Manea E; Stan A; Benea E; OÈ›elea D; Hristea A; LÄƒpÄƒdat I; AntonicÄƒ D; Panait I; Petre R; Kowalska JD; Pietraszkiewicz E; Grycner E; Firlag-Burkacka E; Horban A; Vlaicu O; BÄƒnicÄƒ L; Paraschiv S; DimitrijeviÄ‡ B; JevtoviÄ‡ Ä; KusiÄ‡ J; SalemoviÄ‡ D; Florea D; BÄƒdicuÈ› I; Rafila A; Camburu C; Histrea A; FrÄƒÈ›ilÄƒ M; Gmizic I; Djonin-Nenezic M; Milosevic I; Brmbolic B; NeguÈ› AC; MÄƒrdÄƒrescu A; SÄƒndulescu M; PÃ©rez AB; Chueca N; Ãlvarez M; Alados JC; Rivero A; Vera F; Delgado M; Salmeron J; JimÃ©nez M; Blanco MJ; Diago M; Garcia-deltoro M; Alvarez M; TÃ©llez F; GarcÃ­a F; TÄƒnase D; Bacruban R; Grgic I; Planinic A; Santak M; Gorenec L; Lepej SZ; Vince A; Niculae CM; Merisor S; Kostaki E; Paraskeva D; Skoutelis A; Malliori M; Hackiewicz M; Zabek P; Lunar MM; Mlakar J; Poljak M; Martin E; GheorghiÈ›Äƒ V; Petrescu A; Popescu CI; Neaga E; Ovidiu V; Juncu A; Luca A; LazÄƒr F; Luca AE; GingÄƒraÅŸ C; Anton È˜A; RÄƒdoi R; Tetradov S; ÈšÃ¢rdei G; Nica M; CapÅŸa RA; Achim CL; Oprea C; SzymaÅ„ska B; Gawron N; Pluta A; Åojek E; FirlÄ…g-Burkacka E; Bornstein R; BurcoÈ™ O; Erscoiu SM; Cojanu FB; Toderan A; Popa IC; CeauÈ™u E; Calistru PI; Arbune M; Alexandrache M; Arbune AA; Voinescu DC; Diaconu IA; Stratan L; AramÄƒ V; Nichita L; Diaconu A; Negru A; Orfanu A; LeuÈ™tean A; Ion DA; Ianache I; Popescu C; Catana R; Murariu C; RÄƒdulescu M; Marincu I; Poptelecan P; BicÄƒ V; Tirnea L; Calistru P; Osoianu I; Halacu A; Stoian AC; Dumitrescu F; Diaconescu I; CupÈ™a A; Giubelan L; Ionescu L; Niculescu I; Chiriac C; È˜incu N; Kezdi IZ; Georgescu A; Èšilea B; Girbovan C; Incze A; Fodor A; DrÄƒghicenoiu R; Matei C; Dumea E; Petcu LC; Cambrea SC; Cupsa A; Hurezeanu D; Dragonu L; Cotulbea M; Stroie D; Ionescu P; DuÈ›Äƒ N; Dobrea C; Voican I; Obretin O; StÄƒnescu M; Jianu M; Bes JI; Begovac J; Horga LE; Itu C; David-Aldea LA; Ciorogar A; Jianu C; LupÈ™e M; Caramangiu I; RoÈ™ca O; Cialma M; Ardeleanu A; Irimescu N; Niculae C; Kusic J; Dimitrijevic B; Dragovic G; Aldea-David LA; Manciuc C; Nicolau C; PrisÄƒcariu L; Largu A; Iancu M; VintilÄƒ S; Vitelaru D; Ionel I; MÄƒrdÄƒrescu AH; Micsanschi P; Holban T; BÃ®striÈ›chi I; PÃ¢rÈ›Ã¢nÄƒ L; NagÃ®È› A; Popovici S; Talmaci M; Cucerova I; Mitrescu SG; Mihalcea D; Maricu I; Munteanu D; MihÄƒilescu R; Diaconu I; NÄƒstase R; Molagic V; Duport I; Dragomirescu C; AramÄƒ È˜S; NegruÈ› NM; NiÈ›Äƒ VE; Munteanu DI; AramÄƒ SÈ˜; Pavlovia IP; Desaga D; Mitrescu S; È˜incu NI; GÃ¢rbovan C; Chiriac CL; Talnariu A; Suciu S; Iacob DG; Iacob S; DrÄƒgÄƒnescu M; Iancu A; Benea S; Hrisca R; Tanase D | 2016 | Excluded | Irrelevant, did not discuss the subject matter |
| 678 | Pulmonary Cryptococcosis in HIV- sero-negative patients: case series from India. | Chopra S; Capoor MR; Mallik R; Gupta S; Ray A; Khanna G; Suri JC; Bhattacharya D; Raghavan S | 2015 | Excluded | Irrelevant, did not discuss the subject matter |
| 679 | Prevalence of the VNIc genotype of Cryptococcus neoformans in non-HIV-associated cryptococcosis in the Republic of Korea. | Choi YH; Ngamskulrungroj P; Varma A; Sionov E; Hwang SM; Carriconde F; Meyer W; Litvintseva AP; Lee WG; Shin JH; Kim EC; Lee KW; Choi TY; Lee YS; Kwon-Chung KJ | 2010 | Excluded | Irrelevant, did not discuss the subject matter |
| 680 | Antibody responses to Cryptococcus neoformans in Indian patients with cryptococcosis. | Saha DC; Xess I; Zeng WY; Goldman DL | 2008 | Excluded | Irrelevant, did not discuss the subject matter |
| 681 | Determining the burden of fungal infections in Zimbabwe. | Pfavayi LT; Denning DW; Baker S; Sibanda EN; Mutapi F | 2021 | Excluded | Irrelevant, did not discuss the subject matter |
| 682 | Microevolution of Serial Clinical Isolates of Cryptococcus neoformans var. grubii and C.Â gattii. | Chen Y; Farrer RA; Giamberardino C; Sakthikumar S; Jones A; Yang T; Tenor JL; Wagih O; Van Wyk M; Govender NP; Mitchell TG; Litvintseva AP; Cuomo CA; Perfect JR | 2017 | Excluded | Irrelevant, did not discuss the subject matter |
| 683 | International NeuroAIDS: prospects of HIV-1 associated neurological complications. | Trujillo JR; Jaramillo-Rangel G; Ortega-Martinez M; Penalva de Oliveira AC; Vidal JE; Bryant J; Gallo RC | 2005 | Excluded | Irrelevant, did not discuss the subject matter |
| 684 | Educational Content and Acceptability of Training Using Mobile Instant Messaging in Large HIV Clinics in Malawi. | Heller T; BÃ©lard S; Sande O; Kumwenda T; Gumulira J; Ganesh P; Gugsa S; Tweya H; Phiri S | 2021 | Excluded | Irrelevant, did not discuss the subject matter |
| 685 | Burden of fungal infections in Senegal. | Badiane AS; Ndiaye D; Denning DW | 2015 | Excluded | Irrelevant, did not discuss the subject matter |
| 686 | Opportunistic infections and HIV clinical disease stage among patients presenting for care in Phnom Penh, Cambodia. | Kong BN; Harwell JI; Suos P; Lynen L; Mohiuddin S; Reinert S; Pugatch D | 2007 | Excluded | Irrelevant, did not discuss the subject matter |
| 687 | Bictegravir, emtricitabine, and tenofovir alafenamide versus dolutegravir, emtricitabine, and tenofovir disoproxil fumarate for initial treatment of HIV-1 and hepatitis B coinfection (ALLIANCE): a double-blind, multicentre, randomised controlled, phase 3 | Avihingsanon A; Lu H; Leong CL; Hung CC; Koenig E; Kiertiburanakul S; Lee MP; Supparatpinyo K; Zhang F; Rahman S; D'Antoni ML; Wang H; Hindman JT; Martin H; Baeten JM; Li T | 2023 | Excluded | Irrelevant, did not discuss the subject matter |
| 688 | [Overview of infectious and non-infectious diseases in French Guiana in 2022]. | Epelboin L; Abboud P; Abdelmoumen K; About F; Adenis A; Blaise T; Blaizot R; Bonifay T; Bourne-Watrin M; Boutrou M; Carles G; Carlier PY; Carod JF; Carvalho L; CouppiÃ© P; De Toffol B; Delon F; Demar M; Destoop J; Douine M; Droz JP; Elenga N; Enfissi A; Franck YK; Fremery A; Gaillet M; Kallel H; Kpangon AA; Lavergne A; Le Turnier P; Maisonobe L; Michaud C; Mutricy R; Nacher M; Naldjinan-Kodbaye R; Oberlis M; Odonne G; Osei L; Pujo J; Rabier S; Roman-Laverdure B; Rousseau C; Rousset D; Sabbah N; Sainte-Rose V; Schaub R; Sylla K; Tareau MA; Tertre V; Thorey C; Vialette V; Walter G; Zappa M; Djossou F; Vignier N | 2023 | Excluded | Irrelevant, did not discuss the subject matter |
| 689 | Current burden of serious fungal infections in Republic of Congo. | Amona FM; Denning DW; Moukassa D; Hennequin C | 2020 | Excluded | Irrelevant, did not discuss the subject matter |
| 690 | Cost-effectiveness of reflex laboratory-based cryptococcal antigen screening for the prevention and treatment of cryptococcal meningitis in Botswana. | Tenforde MW; Muthoga C; Callaghan A; Ponatshego P; Ngidi J; Mine M; Jordan A; Chiller T; Larson BA; Jarvis JN | 2019 | Excluded | Irrelevant, did not discuss the subject matter |
| 691 | Presentation and outcome of suspected sepsis in a high-HIV burden, high antiretroviral coverage setting. | Chaka W; Berger C; Huo S; Robertson V; Tachiona C; Magwenzi M; Magombei T; Mpamhanga C; Katzenstein D; Metcalfe J | 2020 | Excluded | Irrelevant, did not discuss the subject matter |
| 692 | Trends in prevalence of selected opportunistic infections associated with HIV/AIDS in Uganda. | Rubaihayo J; Tumwesigye NM; Konde-Lule J | 2015 | Excluded | Irrelevant, did not discuss the subject matter |
| 693 | Superposition of leprosy and other neglected tropical diseases in the state of Rio de Janeiro: a case series report. | Di Luca DG; De Andrade PJ; Sales AM; De Menezes VM; Galhardo MC; Pimentel MI; Lyra MR; Nery JA | 2013 | Excluded | Irrelevant, did not discuss the subject matter |
| 694 | Chronic cough in primary health care attendees, Harare, Zimbabwe: diagnosis and impact of HIV infection. | Munyati SS; Dhoba T; Makanza ED; Mungofa S; Wellington M; Mutsvangwa J; Gwanzura L; Hakim J; Nyakabau M; Mason PR; Robertson V; Rusakaniko S; Butterworth AE; Corbett EL | 2005 | Excluded | Irrelevant, did not discuss the subject matter |
| 695 | The Burden of Serious Fungal Infections in Tajikistan. | Bobokhojaev OI; Osmanov A; Aliev SP; Radjabzoda AS; Avgonov ZT; Manonov ST; Denning DW | 2019 | Excluded | Irrelevant, did not discuss the subject matter |
| 696 | Invasive bacterial and fungal infections among hospitalized HIV-infected and HIV-uninfected children and infants in northern Tanzania. | Crump JA; Ramadhani HO; Morrissey AB; Msuya LJ; Yang LY; Chow SC; Morpeth SC; Reyburn H; Njau BN; Shaw AV; Diefenthal HC; Bartlett JA; Shao JF; Schimana W; Cunningham CK; Kinabo GD | 2011 | Excluded | Irrelevant, did not discuss the subject matter |
| 697 | Correlation between oral lesions and opportunistic infections among human immunodeficiency virus - infected individuals in Indian population. | Sanadhya YK; Sanadhya S; Nagarajappa R; Jain S; Aapaliya P; Sharma N | 2014 | Excluded | Irrelevant, did not discuss the subject matter |
| 698 | Spatio-temporal Distribution of Meningitis in HIV Patients in Northern Egypt (2000-2018). | Abd El-Wahab EW; Hegazy Y; Farrag T; Metwally M | 2020 | Excluded | Irrelevant, did not discuss the subject matter |
| 699 | A holistic review on Cryptococcus neoformans. | Rathore SS; Sathiyamoorthy J; Lalitha C; Ramakrishnan J | 2022 | Excluded | Irrelevant, did not discuss the subject matter |
| 700 | [Clinical, biological, therapeutic and evolving profile of patients with HIV infection hospitalized at Infectious and tropical diseases unit in Abidjan (Ivory Coast)]. | Kra O; Aba YT; Yao KH; Ouattara B; Abouo F; Tanon KA; EholiÃ© S; BissagnenÃ© E | 2013 | Excluded | Irrelevant, did not discuss the subject matter |
| 701 | HIV and/or AIDS-related deaths and modifiable risk factors: A descriptive study of medical admissions at Oshakati Intermediate Hospital in Northern Namibia. | Mgori NK; Mash R | 2015 | Excluded | Irrelevant, did not discuss the subject matter |
| 702 | A Minority of Patients Newly Diagnosed with AIDS Are Started on Antiretroviral Therapy at the Time of Diagnosis in a Large Public Hospital in the Southeastern United States. | Goswami ND; Colasanti J; Khoubian JJ; Huang Y; Armstrong WS; Del Rio C | 2017 | Excluded | Irrelevant, did not discuss the subject matter |
| 703 | Efficacy of amphotericin B in a fat emulsion for the treatment of cryptococcal meningitis in AIDS patients. | Rubio FG; Zanon JR; de Almeida MT; de GÃ³ngora DV | 2007 | Excluded | Irrelevant, did not discuss the subject matter |
| 704 | Population Genetic Analysis Reveals a High Genetic Diversity in the Brazilian Cryptococcus gattii VGII Population and Shifts the Global Origin from the Amazon Rainforest to the Semi-arid Desert in the Northeast of Brazil. | Souto AC; Bonfietti LX; Ferreira-Paim K; Trilles L; Martins M; Ribeiro-Alves M; Pham CD; Martins L; Dos Santos W; Chang M; Brito-Santos F; Santos DC; Fortes S; Lockhart SR; Wanke B; Melhem MS; LazÃ©ra MS; Meyer W | 2016 | Excluded | Irrelevant, did not discuss the subject matter |
| 705 | Multilocus Sequence Typing Reveals both Shared and Unique Genotypes of Cryptococcus neoformans in Jiangxi Province, China. | Chen YH; Yu F; Bian ZY; Hong JM; Zhang N; Zhong QS; Hang YP; Xu J; Hu LH | 2018 | Excluded | Irrelevant, did not discuss the subject matter |
| 706 | Cryptococcus gattii serotype-C strains isolated in Bangalore, Karnataka, India. | Cogliati M; Chandrashekar N; Esposto MC; Chandramuki A; Petrini B; Viviani MA | 2012 | Excluded | Irrelevant, did not discuss the subject matter |
| 707 | Cryptococcal and Histoplasma Antigen Screening Among People With Human Immunodeficiency Virus in Ghana and Comparative Analysis of OIDx Histoplasma Lateral Flow Assay and IMMY Histoplasma Enzyme Immunoassay. | Ocansey BK; Otoo B; Asamoah I; Ganu V; Berko KP; Oladele O; Amankwa EA; Opoku-Asare B; Agyei M; George L; Kotey FCN; Kosmidis C; Puplampu P; Opintan JA; Denning DW | 2022 | Excluded | Irrelevant, did not discuss the subject matter |
| 708 | Cryptococcosis in nonhuman immunodeficiency virus-infected children. | Huang KY; Huang YC; Hung IJ; Lin TY | 2010 | Excluded | Irrelevant, did not discuss the subject matter |
| 709 | Diagnostic capacity for invasive fungal infections in advanced HIV disease in Africa: a continent-wide survey. | Lakoh S; Kamudumuli PS; Penney ROS; Haumba SM; Jarvis JN; Hassan AJ; Moudoute NLE; Ocansey BK; Izco S; Kipkerich S; Sacarlal J; Awopeju AT; Govender NP; Munyanji CIM; Guyguy K; Orefuwa E; Denning DW | 2023 | Excluded | Irrelevant, did not discuss the subject matter |
| 710 | Neurological complications in patients with HIV/AIDS. | Bolokadze N; Gabunia P; Ezugbaia M; Gatserelia L; Khechiashvili G | 2008 | Excluded | Irrelevant, did not discuss the subject matter |
| 711 | [Cryptococcus neoformans in the gastric contents of an AIDS patient]. | Garro S; Bava AJ | 2006 | Excluded | Irrelevant, did not discuss the subject matter |
| 712 | Molecular epidemiology and antifungal susceptibility profiles of clinical Cryptococcus neoformans/Cryptococcus gattii species complex. | Bandalizadeh Z; Shokohi T; Badali H; Abastabar M; Babamahmoudi F; Davoodi L; Mardani M; Javanian M; Cheraghmakani H; Sepidgar AA; Badiee P; Khodavaisy S; Afshari SAK; Ahmadikia K; Seyedmousavi S | 2020 | Excluded | Irrelevant, did not discuss the subject matter |
| 713 | Genomic Variation across a Clinical Cryptococcus Population Linked to Disease Outcome. | Sephton-Clark P; Tenor JL; Toffaletti DL; Meyers N; Giamberardino C; Molloy SF; Palmucci JR; Chan A; Chikaonda T; Heyderman R; Hosseinipour M; Kalata N; Kanyama C; Kukacha C; Lupiya D; Mwandumba HC; Harrison T; Bicanic T; Perfect JR; Cuomo CA | 2022 | Excluded | Irrelevant, did not discuss the subject matter |
| 714 | Measurement of antifungal drug levels in cerebrospinal fluid for cryptococcal meningoencephalitis. | Shoji H; Takuma T; Ohbayashi H; Yoshida K; Yamamoto T; Niki Y | 2012 | Excluded | Irrelevant, did not discuss the subject matter |
| 715 | Isolated skull cryptococcosis in an immunocompetent patient. | Kong QT; Zhou WQ; Feng J; Sang H; Deng DQ; Wang Z; Li J; Shi QL; Wu B; Liu WD | 2013 | Excluded | Irrelevant, did not discuss the subject matter |
| 716 | Cryptococcal granulomas in an immunocompromised HIV-negative patient. | Taneja J; Bhargava A; Loomba P; Dogra V; Thakur A; Mishra B | 2008 | Excluded | Irrelevant, did not discuss the subject matter |
| 717 | Immune reconstitution inflammatory syndrome in a resource-poor setting. | Klotz SA; Aziz Mohammed A; Girmai Woldemichael M; Worku Mitku M; Handrich M | 2009 | Excluded | Irrelevant, did not discuss the subject matter |
| 718 | Disseminated cryptococcosis in a patient with HIV/AIDS at a teaching hospital in Ghana. | Akakpo KP; Quayson SE; Lartey M | 2015 | Excluded | Irrelevant, did not discuss the subject matter |
| 719 | Epidemiology and trend of neurological diseases associated to HIV/AIDS. Experience of Mexican patients 1995-2009. | RamÃ­rez-Crescencio MA; VelÃ¡squez-PÃ©rez L | 2013 | Excluded | Irrelevant, did not discuss the subject matter |
| 720 | Central nervous system fungal infections: observations from a large tertiary hospital in northern India. | Sethi PK; Khanna L; Batra A; Anand I; Sethi NK; Torgovnick J; Arsura E | 2012 | Excluded | Irrelevant, did not discuss the subject matter |
| 721 | Cost-effectiveness of cryptococcal antigen screening at CD4 counts of 101-200 cells/ÂµL in Botswana. | Tenforde MW; Muthoga C; Ponatshego P; Ngidi J; Mine M; Greene G; Jordan A; Chiller T; Larson BA; Jarvis JN | 2021 | Excluded | Irrelevant, did not discuss the subject matter |
| 722 | MLST reveals a clonal population structure for Cryptococcus neoformans molecular type VNI isolates from clinical sources in Amazonas, Northern-Brazil. | Rocha DFS; Cruz KS; Santos CSDS; Menescal LSF; Neto JRDS; Pinheiro SB; Silva LM; Trilles L; Braga de Souza JV | 2018 | Excluded | Irrelevant, did not discuss the subject matter |
| 723 | Genotype and mating type analysis of Cryptococcus neoformans and Cryptococcus gattii isolates from China that mainly originated from non-HIV-infected patients. | Feng X; Yao Z; Ren D; Liao W; Wu J | 2008 | Excluded | Irrelevant, did not discuss the subject matter |
| 724 | The clinical utility of the urine-based lateral flow lipoarabinomannan assay in HIV-infected adults in Myanmar: an observational study. | Thit SS; Aung NM; Htet ZW; Boyd MA; Saw HA; Anstey NM; Kyi TT; Cooper DA; Kyi MM; Hanson J | 2017 | Excluded | Irrelevant, did not discuss the subject matter |
| 725 | Clinical characteristics and mortality risk factors of cryptococcal infection among HIV-negative patients. | Pongmekin P; Chongtrakool P; Santanirand P; Kiertiburanakul S | 2014 | Excluded | Irrelevant, did not discuss the subject matter |
| 726 | Predictors of AIDS-related death among adult HIV-infected inpatients in Kisangani, the Democratic Republic of Congo. | Tepungipame AT; Tonen-Wolyec S; Kalla GC; Longembe EB; Atike RO; Likwela JL; Mbopi-KÃ©ou FX; BÃ©lec L; Batina-Agasa S | 2020 | Excluded | Irrelevant, did not discuss the subject matter |
| 727 | [Fungemia in hospitals of the city of Buenos Aires, Argentina]. | LÃ³pez Moral L; Tiraboschi IN; Schijman M; Bianchi M; Guelfand L; Cataldi S | 2012 | Excluded | Irrelevant, did not discuss the subject matter |
| 728 | [Causes of death among 91 HIV-infected adults in the era of potent antiretroviral therapy]. | Sodqi M; Marih L; Lahsen AO; Bensghir R; Chakib A; Himmich H; El Filali KM | 2012 | Excluded | Irrelevant, did not discuss the subject matter |
| 729 | A 12-year study of fungal infections in Rio Grande do Sul, Southern Brazil. | Fay VDS; Gregianini TS; Veiga ABGD; GonÃ§alves SMB; Rodrigues DM; Bonamigo RR | 2019 | Excluded | Irrelevant, did not discuss the subject matter |
| 730 | Molecular characterization and antifungal susceptibility testing of Cryptococcus neoformans sensu stricto from southern Brazil. | Herkert PF; Meis JF; Lucca de Oliveira Salvador G; Rodrigues Gomes R; Aparecida Vicente V; Dominguez Muro M; Lameira Pinheiro R; Lopes Colombo A; Vargas Schwarzbold A; Sakuma de Oliveira C; SimÃ£o Ferreira M; Queiroz-Telles F; Hagen F | 2018 | Excluded | Irrelevant, did not discuss the subject matter |
| 731 | Fungal infections in Sudan: An underestimated health problem. | Ahmed SA; Ismail M; Albirair M; Nail AMA; Denning DW | 2023 | Excluded | Irrelevant, did not discuss the subject matter |
| 732 | [Paraparesis and fever in a Tunisian woman: cryptococcal spondylitis with spinal involvement]. | Nfoussi H; Chelly I; Aamari L; Ben Salem T; Azouz H; Tiouiri Benaissia H; Kchir N; Haouet S; Zitouna M | 2010 | Excluded | Irrelevant, did not discuss the subject matter |
| 733 | [EPIDEMIOLOGY OF VISCERAL FUNGAL INFECTION IN FRANCE AND IN THE WORLD]. | Blot M; Lanternier F; Lortholary O | 2015 | Excluded | Irrelevant, did not discuss the subject matter |
| 734 | Clinical profile of HIV in India. | Kumarasamy N; Vallabhaneni S; Flanigan TP; Mayer KH; Solomon S | 2005 | Excluded | Irrelevant, did not discuss the subject matter |
| 735 | Tracking cryptococcal meningitis to monitor HIV program success during the Treat-All era: an analysis of national data in Botswana. | Milburn J; Ntwayagae O; Suresh R; Ngoni K; Northcott C; Penny J; Kinsella M; Mechie I; Ensor S; Thamae G; Leeme T; Lawrence DS; Chebani T; Grint D; Tenforde MW; Avalos A; Ramaabya D; Ogando J; Mokomane M; Mine M; Jarvis JN | 2024 | Excluded | Irrelevant, did not discuss the subject matter |
| 736 | Hospitalization of HIV positive patients in a referral tertiary care hospital in Antananarivo Madagascar, 2010-2016: Trends, causes and outcome. | Raberahona M; Razafinambinintsoa T; Andriananja V; Ravololomanana N; Tongavelona J; Rakotomalala R; Andriamamonjisoa J; Andrianasolo RL; Rakotoarivelo RA; Randria MJD | 2018 | Excluded | Irrelevant, did not discuss the subject matter |
| 737 | HIV/AIDS care and treatment in three provinces in northern Thailand before the national scale-up of highly-active antiretroviral therapy. | Thanprasertsuk S; Lertpiriyasuwat C; Leusaree T; Sirinirund P; Sumanapan S; Chariyalertsak C; Simmons N; Ellerbrock TV; Siraprapasiri T; Yachompoo C; Panputtanakul S; Virapat P; Supakalin P; Srithaniviboonchai K; Mock P; Supawitkul S; Tappero JW; Levine WC | 2006 | Excluded | Irrelevant, did not discuss the subject matter |
| 738 | The treatment of a pregnant HIV positive patient with cryptococcal meningitis in Malawi. Case report and review of treatment options. | Bright PD; Lupiya D; van Oosterhout JJ; Chen A; Harrison TS; Chan AK | 2018 | Excluded | Irrelevant, did not discuss the subject matter |
| 739 | Operationalizing early antiretroviral therapy in HIV-infected in-patients with opportunistic infections including tuberculosis. | Sunpath H; Edwin C; Chelin N; Nadesan S; Maharaj R; Moosa Y; Smeaton L; Court R; Knight S; Gwyther E; Murphy RA | 2012 | Excluded | Irrelevant, did not discuss the subject matter |
| 740 | Neuropathology of HIV/AIDS with an overview of the Indian scene. | Shankar SK; Mahadevan A; Satishchandra P; Kumar RU; Yasha TC; Santosh V; Chandramuki A; Ravi V; Nath A | 2005 | Excluded | Irrelevant, did not discuss the subject matter |
| 741 | Cryptococcal Antigenemia in Immunocompromised Human Immunodeficiency Virus Patients in Rural Tanzania: A Preventable Cause of Early Mortality. | Letang E; MÃ¼ller MC; Ntamatungiro AJ; Kimera N; Faini D; Furrer H; Battegay M; Tanner M; Hatz C; Boulware DR; Glass TR | 2015 | Excluded | Irrelevant, did not discuss the subject matter |
| 742 | Opportunistic Cryptococcal Antigenemia in the HAART Era at HIV Epidemic Settings of Northwest Ethiopia. | Negash M; Wondmagegn T; Tajebe F | 2020 | Excluded | Irrelevant, did not discuss the subject matter |
| 743 | AIDS-defining illnesses: a comparison between before and after commencement of highly active antiretroviral therapy (HAART). | Lian YL; Heng BS; Nissapatorn V; Lee C | 2007 | Excluded | Irrelevant, did not discuss the subject matter |
| 744 | Comparative typing analyses of clinical and environmental strains of the Cryptococcus neoformans/Cryptococcus gattii species complex from Ivory Coast. | Kassi FK; Bellet V; Drakulovski P; Krasteva D; Roger F; ValÃ©rie BA; Aboubakar T; Doumbia A; Kouakou GA; Delaporte E; Reynes J; Yavo W; Menan HIE; Bertout S | 2018 | Excluded | Irrelevant, did not discuss the subject matter |
| 745 | The burden of serious fungal infections in Azerbaijan. | Huseynov RM; Javadov SS; Osmanov A; Khasiyev S; Valiyeva SR; Almammadova E; Denning DW | 2021 | Excluded | Irrelevant, did not discuss the subject matter |
| 746 | Invasive bacterial and fungal infections among hospitalized HIV-infected and HIV-uninfected adults and adolescents in northern Tanzania. | Crump JA; Ramadhani HO; Morrissey AB; Saganda W; Mwako MS; Yang LY; Chow SC; Morpeth SC; Reyburn H; Njau BN; Shaw AV; Diefenthal HC; Shao JF; Bartlett JA; Maro VP | 2011 | Excluded | Irrelevant, did not discuss the subject matter |
| 747 | Highly active antiretroviral therapy access and neurological complications of human immunodeficiency virus infection: impact versus resources in Brazil. | Silva MT; AraÃºjo A | 2005 | Excluded | Irrelevant, did not discuss the subject matter |
| 748 | Immune reconstitution syndrome after highly active antiretroviral therapy in human immunodeficiency virus-infected thai children. | Puthanakit T; Oberdorfer P; Akarathum N; Wannarit P; Sirisanthana T; Sirisanthana V | 2006 | Excluded | Irrelevant, did not discuss the subject matter |
| 749 | Immune reconstitution inflammatory syndrome in adult human immunodeficiency virus-infected patients in Thailand. | Aramaki M; Silachamroon U; Desakorn V; Maek-A-nantawat W; Waiwaruwut J; Jutiwarakun K; Kim JH; Pitisuttithum P | 2010 | Excluded | Irrelevant, did not discuss the subject matter |
| 750 | Human Immune Response Varies by the Degree of Relative Cryptococcal Antigen Shedding. | Boulware DR; von Hohenberg M; Rolfes MA; Bahr NC; Rhein J; Akampurira A; Williams DA; Taseera K; Schutz C; McDonald T; Muzoora C; Meintjes G; Meya DB; Nielsen K; Huppler Hullsiek K | 2016 | Excluded | Irrelevant, did not discuss the subject matter |
| 751 | Incidence and predictors of mortality and the effect of tuberculosis immune reconstitution inflammatory syndrome in a cohort of TB/HIV patients commencing antiretroviral therapy. | Worodria W; Massinga-Loembe M; Mazakpwe D; Luzinda K; Menten J; Van Leth F; Mayanja-Kizza H; Kestens L; Mugerwa RD; Reiss P; Colebunders R | 2011 | Excluded | Irrelevant, did not discuss the subject matter |
| 752 | Serious fungal diseases in Democratic Republic of Congo - Incidence and prevalence estimates. | Kamwiziku GK; Makangara JC; Orefuwa E; Denning DW | 2021 | Excluded | Irrelevant, did not discuss the subject matter |
| 753 | Clinical significance of normal chest radiographs among HIV-seropositive patients with suspected tuberculosis in Uganda. | Yoo SD; Cattamanchi A; Den Boon S; Worodria W; Kisembo H; Huang L; Davis JL | 2011 | Excluded | Irrelevant, did not discuss the subject matter |
| 754 | The burden of serious fungal infections in Sierra Leone: a national estimate. | Lakoh S; Orefuwa E; Kamara MN; Jiba DF; Kamara JB; Kpaka S; Denning DW | 2021 | Excluded | Irrelevant, did not discuss the subject matter |
| 755 | Spectrum and mortality of opportunistic infections among HIV/AIDS patients in southwestern China. | Meng S; Tang Q; Xie Z; Wu N; Qin Y; Chen R; Chen X; Li Y; Shi M; Ye L; Liang H; Jiang J; Zhou B; Lin J | 2023 | Excluded | Irrelevant, did not discuss the subject matter |
| 756 | Identification of Pathogen Genomic Differences That Impact Human Immune Response and Disease during Cryptococcus neoformans Infection. | Gerstein AC; Jackson KM; McDonald TR; Wang Y; Lueck BD; Bohjanen S; Smith KD; Akampurira A; Meya DB; Xue C; Boulware DR; Nielsen K | 2019 | Excluded | Irrelevant, did not discuss the subject matter |
| 757 | [Mycobacterium bovis tuberculosis in a female patient with AIDS]. | Valerga M; Viola C; Thwaites A; Bases O; Ambroggi M; Poggi S; Marino R | 2005 | Excluded | Irrelevant, did not discuss the subject matter |
| 758 | Cutaneous cryptococcosis due to Cryptococcus gattii in a patient on chronic corticotherapy. | Bellissimo-Rodrigues F; Baciotti M; Zanatto MP; Silva JO; Martins Mdos A; Martinez R | 2010 | Excluded | Irrelevant, did not discuss the subject matter |
| 759 | Clinical and epidemiological analysis of patients with HIV/AIDS admitted to a reference hospital in the northeast region of Brazil. | Soares VY; LÃºcio Filho CE; Carvalho LI; Silva AM; EulÃ¡lio KD | 2008 | Excluded | Irrelevant, did not discuss the subject matter |
| 760 | Prevalence of cryptococcal antigen positivity among HIV infected patient with CD4 cell count less than 100 of Imam Khomeini Hospital, Tehran, Iran. | Hajiabdolbaghi M; Kalantari S; Jamshidi-Makiani M; Shojaei E; Abbasian L; Rasoulinezhad M; Tayeri K | 2017 | Excluded | Irrelevant, did not discuss the subject matter |
| 761 | Seizure in HIV-infected patients: clinical presentation, cause and treatment outcome in Ethiopia-a retrospective study. | Amare A | 2021 | Excluded | Irrelevant, did not discuss the subject matter |
| 762 | Genotypic diversity and antifungal susceptibility of Cryptococcus neoformans isolates from paediatric patients in China. | Hong N; Chen M; Xu N; Al-Hatmi AMS; Zhang C; Pan WH; Hagen F; Boekhout T; Xu J; Zou XB; Liao WQ | 2019 | Excluded | Irrelevant, did not discuss the subject matter |
| 763 | Symptomatic Cryptococcal Meningitis with Negative Serum and Cerebrospinal Fluid Cryptococcal Antigen Tests. | Nanfuka V; Mkhoi ML; Gakuru J; Kwizera R; Baluku JB; Bongomin F; Meya DB | 2021 | Excluded | Irrelevant, did not discuss the subject matter |
| 764 | Effects of Gender and Baseline CD4 Count on Post-Treatment CD4 Count Recovery and Outcomes in Patients with Advanced HIV Disease: A Retrospective Cohort Study. | Kouamou V; Gundidza P; Ndhlovu CE; Makadzange AT | 2023 | Excluded | Irrelevant, did not discuss the subject matter |
| 765 | Neuropathology of human immunodeficiency virus infection: a forensic autopsy study in Dar Es Salaam, Tanzania. | Ng'walali PM; Kibayashi K; Mbonde MP; Harada S; Mwakagile D; Kitinya JN; Tsunenari S | 2005 | Excluded | Irrelevant, did not discuss the subject matter |
| 766 | Predictors of prolonged hospital stay in HIV-positive patients presenting to the emergency department. | Laher AE; Paruk F; Richards GA; Venter WDF | 2021 | Excluded | Irrelevant, did not discuss the subject matter |
| 767 | Effects of CD4 cell count and antiretroviral therapy on mucocutaneous manifestations among HIV/AIDS patients in Yunnan, China. | Li YY; Yang SH; Wang RR; Tang JT; Wang HM; Kuang YQ | 2020 | Excluded | Irrelevant, did not discuss the subject matter |
| 768 | Cryptococcal antigen prevalence in HIV patients from a tertiary care centre in South India. | Madhavan A; Sachu A; Samuel A; Vasudevapanicker J | 2022 | Excluded | Irrelevant, did not discuss the subject matter |
| 769 | Opportunistic fungal infections in persons living with advanced HIV disease in Lagos, Nigeria; a 12-year retrospective study. | Oladele R; Ogunsola F; Akanmu A; Stocking K; Denning DW; Govender N | 2020 | Excluded | Irrelevant, did not discuss the subject matter |
| 770 | High Burden of Cryptococcal Meningitis Among Antiretroviral Therapy-Experienced Human Immunodeficiency Virus-Infected Patients in Northern Uganda in the Era of "Test and Treat": Implications for Cryptococcal Screening Programs. | Okwir M; Link A; Rhein J; Obbo JS; Okello J; Nabongo B; Alal J; Meya D; Bohjanen PR | 2022 | Excluded | Irrelevant, did not discuss the subject matter |
| 771 | Clinicopathologic and ultrastructural study of non-HIV-related primary pulmonary cryptococcosis in China: report of 43 cases. | Zeng Y; Wu X; Yi X; Luo B; Zhu M; Rui W; Zhu X; Li X; Li H | 2011 | Excluded | Irrelevant, did not discuss the subject matter |
| 772 | The medical management of central nervous system infections in Uganda and the potential impact of an algorithm-based approach to improve outcomes. | Trachtenberg JD; Kambugu AD; McKellar M; Semitala F; Mayanja-Kizza H; Samore MH; Ronald A; Sande MA | 2007 | Excluded | Irrelevant, did not discuss the subject matter |
| 773 | Ecological surveys of the Cryptococcus species complex in China. | Li AS; Pan WH; Wu SX; Hideaki T; Guo NR; Shen YN; Lu GX; Pan RG; Zhu MC; Chen M; Shi WM; Liao WQ | 2012 | Excluded | Irrelevant, did not discuss the subject matter |
| 774 | Multilocus Sequence Typing of Clinical Isolates of Cryptococcus from India. | Xess I; Pandey M; Dabas Y; Agarwal R; Das S; Srivastava PMV; Thakur R; Sharma S; Mani P; Biswas A; Bhowmik D; Das BK; Singh R; Panda P; Singh G; Phukan AC; Yhome M; Valsan C; Kindo AJ | 2021 | Excluded | Irrelevant, did not discuss the subject matter |
| 775 | Estimated Burden of Serious Fungal Infections in Malawi. | Kalua K; Zimba B; Denning DW | 2018 | Excluded | Irrelevant, did not discuss the subject matter |
| 776 | Use of traditional medicines in the management of HIV/AIDS opportunistic infections in Tanzania: a case in the Bukoba rural district. | Kisangau DP; Lyaruu HV; Hosea KM; Joseph CC | 2007 | Excluded | Irrelevant, did not discuss the subject matter |
| 777 | Decision making in a clinical trial for a life-threatening illness: Therapeutic expectation, not misconception. | Lawrence DS; Ssali A; Moshashane N; Nabaggala G; Maphane L; Harrison TS; Meya DB; Jarvis JN; Seeley J | 2022 | Excluded | Irrelevant, did not discuss the subject matter |
| 778 | The effect of HIV infection on adult meningitis in Indonesia: a prospective cohort study. | Ganiem AR; Parwati I; Wisaksana R; van der Zanden A; van de Beek D; Sturm P; van der Ven A; Alisjahbana B; Brouwer AM; Kurniani N; de Gans J; van Crevel R | 2009 | Excluded | Irrelevant, did not discuss the subject matter |
| 779 | Burden of serious fungal infections in Guatemala. | Medina N; Samayoa B; Lau-Bonilla D; Denning DW; Herrera R; Mercado D; GuzmÃ¡n B; PÃ©rez JC; Arathoon E | 2017 | Excluded | Irrelevant, did not discuss the subject matter |
| 780 | Rapid urine-based screening tests increase the yield of same-day tuberculosis diagnoses among patients living with advanced HIV disease. | Wake RM; Govender NP; Omar SV; Ismail F; Tiemessen CT; Harrison TS; Jarvis JN | 2022 | Excluded | Irrelevant, did not discuss the subject matter |
| 781 | HIV-attributable causes of death in the medical ward at the Chris Hani Baragwanath Hospital, South Africa. | Black A; Sitas F; Chibrawara T; Gill Z; Kubanje M; Williams B | 2019 | Excluded | Irrelevant, did not discuss the subject matter |
| 782 | The impact of antiretroviral therapy on symptom burden among HIV outpatients with low CD4 count in rural Uganda: nested longitudinal cohort study. | Wakeham K; Harding R; Levin J; Parkes-Ratanshi R; Kamali A; Lalloo DG | 2017 | Excluded | Irrelevant, did not discuss the subject matter |
| 783 | Characteristics of HIV-positive patients infected by vertical transmission, Campo Grande, MS, Brazil, 1993-2009. | de Matos VT; de Oliveira AL; de Souza AS; Dal Fabbro MM | 2012 | Excluded | Irrelevant, did not discuss the subject matter |
| 784 | Estimated Burden of Fungal Infections in Namibia. | Dunaiski CM; Denning DW | 2019 | Excluded | Irrelevant, did not discuss the subject matter |
| 785 | Finger-Prick Whole Blood Cryptococcal Antigen Lateral Flow Assay for the Diagnosis of Cryptococcosis in HIV-Negative Patients: A Case Series Study in Two Tertiary Centers in SÃ£o Paulo, Brazil. | Vidal JE; Oliveira FG; Vieira M; Pereira L; Lucas Junior RM; Guedes BF; Magri MC; Boulware DR | 2023 | Excluded | Irrelevant, did not discuss the subject matter |
| 786 | Opportunistic Infections among People Living with HIV (PLHIV) with Diabetes Mellitus (DM) Attending a Tertiary Care Hospital in Coastal City of South India. | Indira P; Kumar PM; Shalini S; Vaman K | 2015 | Excluded | Irrelevant, did not discuss the subject matter |
| 787 | Updated estimation of the burden of fungal disease in Vietnam. | Duong TN; Le MH; Beardsley J; Denning DW; Le NH; Nguyen BT | 2023 | Excluded | Irrelevant, did not discuss the subject matter |
| 788 | Low uptake of antiretroviral therapy after admission with human immunodeficiency virus and tuberculosis in KwaZulu-Natal, South Africa. | Murphy RA; Sunpath H; Taha B; Kappagoda S; Maphasa KT; Kuritzkes DR; Smeaton L | 2010 | Excluded | Irrelevant, did not discuss the subject matter |
| 789 | Addressing advanced HIV disease and mortality in global HIV programming. | Boyd AT; Oboho I; Paulin H; Ali H; Godfrey C; Date A; Sean Cavanaugh J | 2020 | Excluded | Irrelevant, did not discuss the subject matter |
| 790 | Neurological disease in HIV-infected patients in the era of highly active antiretroviral treatment: a Brazilian experience. | Oliveira JF; Greco DB; Oliveira GC; Christo PP; GuimarÃ£es MD; Oliveira RC | 2006 | Excluded | Irrelevant, did not discuss the subject matter |
| 791 | Rapid Fatal Outcome of Cryptococcal Meningoencephalitis in a Non-HIV Immunocompromised Patient with a Low Fluconazole Susceptibility Isolate: A Case Report from Madagascar. | Raberahona M; Rakotoarivelo RA; Randriamampionona N; Rakotomalala AF; Razafinambinintsoa T; BÃ©net T; Vanhems P; Randria MJ; Cornet M; Rakoto-Andrianarivelo M | 2016 | Excluded | Irrelevant, did not discuss the subject matter |
| 792 | Cerebral toxoplasmosis mimicking subacute meningitis in HIV-infected patients; a cohort study from Indonesia. | Ganiem AR; Dian S; Indriati A; Chaidir L; Wisaksana R; Sturm P; Melchers W; van der Ven A; Parwati I; van Crevel R | 2013 | Excluded | Irrelevant, did not discuss the subject matter |
| 793 | Management of cryptococcosis in non-HIV-related patients. | Yao Z; Liao W; Chen R | 2005 | Excluded | Irrelevant, did not discuss the subject matter |
| 794 | Meningitis caused by Rhodotorula rubra in an human immunodeficiency virus infected patient. | Thakur K; Singh G; Agarwal S; Rani L | 2007 | Excluded | Irrelevant, did not discuss the subject matter |
| 795 | HIV infection and AIDS in a small municipality in Southeast Brazil. | Eyer-Silva WA; BasÃ­lio-de-Oliveira CA; Morgado MG | 2005 | Excluded | Irrelevant, did not discuss the subject matter |
| 796 | Analyses of pediatric isolates of Cryptococcus neoformans from South Africa. | Miglia KJ; Govender NP; Rossouw J; Meiring S; Mitchell TG | 2011 | Excluded | Irrelevant, did not discuss the subject matter |
| 797 | Using laboratory data to assess the impact of coronavirus (COVID-19) on reflex cryptococcal antigenaemia (CrAg) testing in South Africa. | Cassim N; Coetzee LM; da Silva MP; Stevens WS; Glencross DK | 2023 | Excluded | Irrelevant, did not discuss the subject matter |
| 798 | Central nervous system infections in HIV-infected patients hospitalized at King Chulalongkorn Memorial Hospital. | Kongsiriwattanakul S; Suankratay C | 2011 | Excluded | Irrelevant, did not discuss the subject matter |
| 799 | Correlation of Etest and Neo-Sensitabs diffusion assays on Mueller-Hinton-methylene blue agar with broth microdilution reference method (CLSI-M27-A2) for testing susceptibilities of Cryptococcus neoformans to amphotericin B and fluconazole. | Ochiuzzi ME; Santiso GM; Arechavala AI | 2010 | Excluded | Irrelevant, did not discuss the subject matter |
| 800 | Estimated Burden of Serious Fungal Infections in Jamaica by Literature Review and Modelling. | Gugnani HC; Denning DW | 2015 | Excluded | Irrelevant, did not discuss the subject matter |
| 801 | The Frozen Brain State of Cryptococcus gattii: A Globe-Trotting, Tropical, Neurotropic Fungus. | Akins PT; Jian B | 2019 | Excluded | Irrelevant, did not discuss the subject matter |
| 802 | Etiology and risk factors of stroke in HIV-infected patients in Siriraj Hospital: a case-control study. | Lee B; Anekthananon T; Poungvarin N; Nilanont Y | 2012 | Excluded | Irrelevant, did not discuss the subject matter |
| 803 | Burden of serious fungal infection in Nigeria. | Oladele RO; Denning DW | 2014 | Excluded | Irrelevant, did not discuss the subject matter |
| 804 | Epidemiology of invasive fungal infections in patients with acquired immunodeficiency syndrome at a reference hospital for infectious diseases in Brazil. | de Oliveira RB; Atobe JH; Souza SA; de Castro Lima Santos DW | 2014 | Excluded | Irrelevant, did not discuss the subject matter |
| 805 | [An analysis of opportunistic infection in 762 inpatients with human immunodeficiency virus infection in Guangdong areas]. | Huang LF; Tang XP; Cai WP; Chen XJ; Lei CL; Li LH; Zhang FC | 2010 | Excluded | Irrelevant, did not discuss the subject matter |
| 806 | A Rapid Screening Program for Histoplasmosis, Tuberculosis, and Cryptococcosis Reduces Mortality in HIV Patients from Guatemala. | Medina N; Alastruey-Izquierdo A; Bonilla O; Gamboa O; Mercado D; PÃ©rez JC; Salazar LR; Arathoon E; Denning DW; Rodriguez-Tudela JL | 2021 | Excluded | Irrelevant, did not discuss the subject matter |
| 807 | Central nervous system disorders after starting antiretroviral therapy in South Africa. | Asselman V; Thienemann F; Pepper DJ; Boulle A; Wilkinson RJ; Meintjes G; Marais S | 2010 | Excluded | Irrelevant, did not discuss the subject matter |
| 808 | Nanopore targeted sequencing-based diagnosis of central nervous system infections in HIV-infected patients. | Yang X; Zhou S; Chang Z; Xi X; Li J; Miao M; Chen Y; Chen W; Zhang H; Ding R; Hu Z | 2024 | Excluded | Irrelevant, did not discuss the subject matter |
| 809 | Antifungal susceptibilities of Cryptococcus species complex isolates from AIDS and non-AIDS patients in Southeast China. | Li M; Liao Y; Chen M; Pan W; Weng L | 2012 | Excluded | Irrelevant, did not discuss the subject matter |
| 810 | Electrophoretic karyotypes of C. neoformans serotype A recovered from Thai patients with AIDS. | Ngamwongsatit P; Sukroongreung S; Nilakul C; Prachayasittikul V; Tantimavanich S | 2005 | Excluded | Irrelevant, did not discuss the subject matter |
| 811 | Spectrum of central nervous system disorders in hospitalized HIV/AIDS patients (2009-2011) at a major HIV/AIDS referral center in Beijing, China. | Dai L; Mahajan SD; Guo C; Zhang T; Wang W; Li T; Jiang T; Wu H; Li N | 2014 | Excluded | Irrelevant, did not discuss the subject matter |
| 812 | Molecular Epidemiology Reveals Low Genetic Diversity among Cryptococcus neoformans Isolates from People Living with HIV in Lima, Peru, during the Pre-HAART Era. | van de Wiele N; Neyra E; Firacative C; Gilgado F; Serena C; Bustamante B; Meyer W | 2020 | Excluded | Irrelevant, did not discuss the subject matter |
| 813 | Clinical features vary by the aetiology of meningitis in HIV seropositive patients: A four-year study from a tertiary hospital in India. | Sridhar A; Krishna Sagar G | 2018 | Excluded | Irrelevant, did not discuss the subject matter |
| 814 | Estimated Burden of Serious Fungal Infections in Mozambique. | Sacarlal J; Denning DW | 2018 | Excluded | Irrelevant, did not discuss the subject matter |
| 815 | Predictors of in-hospital mortality among HIV-positive patients presenting with an acute illness to the emergency department. | Laher AE; Paruk F; Venter W; Ayeni OA; Richards GA | 2021 | Excluded | Irrelevant, did not discuss the subject matter |
| 816 | [Current aspects of neuromeningeal cryptococcosis in adults infected with HIV in the internal medicine service of the University Hospital of Treichville Abidjan (Cote d'Ivoire)]. | Kadjo K; Ouattara B; Adoubryn KD; Kra O; Niamkey EK | 2011 | Excluded | Irrelevant, did not discuss the subject matter |
| 817 | Deficiency of mannose-binding lectin is a risk of Pneumocystis jirovecii pneumonia in a natural history cohort of people living with HIV/AIDS in Northern Thailand. | Yanagisawa K; Wichukchinda N; Tsuchiya N; Yasunami M; Rojanawiwat A; Tanaka H; Saji H; Ogawa Y; Handa H; Pathipvanich P; Ariyoshi K; Sawanpanyalert P | 2020 | Excluded | Irrelevant, did not discuss the subject matter |
| 818 | Development of Severe Anemia and Changes in Hemoglobin in a Cohort of HIV-Infected Ugandan Adults Receiving Zidovudine-, Stavudine-, and Tenofovir-Containing Antiretroviral Regimens. | Parkes-Ratanshi R; Katende D; Levin J; Wakeham K; Heiner G; Kamali A; Lalloo DG | 2015 | Excluded | Irrelevant, did not discuss the subject matter |
| 819 | Cryptococcal Antigenemia in Nigerian Patients With Advanced Human Immunodeficiency Virus: Influence of Antiretroviral Therapy Adherence. | Oladele RO; Akanmu AS; Nwosu AO; Ogunsola FT; Richardson MD; Denning DW | 2016 | Excluded | Irrelevant, did not discuss the subject matter |
| 820 | Burden of Severe Fungal Infections in Burkina Faso. | Bamba S; Zida A; SangarÃ© I; CissÃ© M; Denning DW; Hennequin C | 2018 | Excluded | Irrelevant, did not discuss the subject matter |
| 821 | Incidence, clinical spectrum, risk factors and impact of HIV-associated immune reconstitution inflammatory syndrome in South Africa. | Haddow LJ; Moosa MY; Mosam A; Moodley P; Parboosing R; Easterbrook PJ | 2012 | Excluded | Irrelevant, did not discuss the subject matter |
| 822 | Evaluation of point of care serum cryptococcal antigen by lateral flow immunoassay for diagnosis of cryptococcosis and cryptococcal meningitis in HIV-positive patients. | Kamble U; Dheeresh KH; Bhosale K; Indu MB; Sharma B; Chowdhary A | 2021 | Excluded | Irrelevant, did not discuss the subject matter |
| 823 | Disseminated Cryptococcosis Is a Common Finding among Human Immunodeficiency Virus-Infected Patients with Suspected Sepsis and Is Associated with Higher Mortality Rates. | Tufa TB; Orth HM; Wienemann T; Jensen BO; Mackenzie CR; Boulware DR; Luedde T; Feldt T | 2023 | Excluded | Irrelevant, did not discuss the subject matter |
| 824 | Clinical and Metagenomic Characterization of Neurological Infections of People With Human Immunodeficiency Virus in the Peruvian Amazon. | Steinberg HE; Ramachandran PS; Diestra A; Pinchi L; Ferradas C; Kirwan DE; Diaz MM; Sciaudone M; Wapniarski A; Zorn KC; CalderÃ³n M; Cabrera L; Pinedo-Cancino V; Wilson MR; Asayag CR; Gilman RH; Bowman NM | 2023 | Excluded | Irrelevant, did not discuss the subject matter |
| 825 | Neurological manifestations in people living with HIV/AIDS in the late cART era: a prospective observational study at a tertiary healthcare center in SÃ£o Paulo, Brazil. | Telles JP; Fernandes R; Barros TD; Maestri A; Vitoriano T; Borges L; Teixeira R; Marcusso R; Haziot M; De Oliveira AP; Vidal JE | 2021 | Excluded | Irrelevant, did not discuss the subject matter |
| 826 | Estimated burden of fungal infections in Kenya. | Guto JA; Bii CC; Denning DW | 2016 | Excluded | Irrelevant, did not discuss the subject matter |
| 827 | Molecular typing, in vitro susceptibility and virulence of Cryptococcus neoformans/Cryptococcus gattii species complex clinical isolates from south-eastern Brazil. | Grizante BariÃ£o PH; Tonani L; Cocio TA; Martinez R; Nascimento Ã‰; von Zeska Kress MR | 2020 | Excluded | Irrelevant, did not discuss the subject matter |
| 828 | First molecular typing of cryptococcemia-causing cryptococcus in central-west Brazil. | Tsujisaki RA; Paniago AM; Lima JÃºnior MS; Alencar Dde S; Spositto FL; Nunes Mde O; Trilles L; Chang MR | 2013 | Excluded | Irrelevant, did not discuss the subject matter |
| 829 | Pulmonary cryptococcosis presenting as acute severe respiratory distress in a newly diagnosed HIV patient in Tanzania: a case report. | Gunda DW; Bakshi FA; Rambau P; Kilonzo SB | 2015 | Excluded | Irrelevant, did not discuss the subject matter |
| 830 | The Diagnostic Laboratory Hub: A New Health Care System Reveals the Incidence and Mortality of Tuberculosis, Histoplasmosis, and Cryptococcosis of PWH in Guatemala. | Samayoa B; Aguirre L; Bonilla O; Medina N; Lau-Bonilla D; Mercado D; Moller A; Perez JC; Alastruey-Izquierdo A; Arathoon E; Denning DW; RodrÃ­guez-Tudela JL | 2020 | Excluded | Irrelevant, did not discuss the subject matter |
| 831 | Reversal of CSF HIV-1 Escape during Treatment of HIV-Associated Cryptococcal Meningitis in Botswana. | Kelentse N; Moyo S; Molebatsi K; Morerinyane O; Bitsang S; Bareng OT; Lechiile K; Leeme TB; Lawrence DS; Kasvosve I; Musonda R; Mosepele M; Harrison TS; Jarvis JN; Gaseitsiwe S | 2022 | Excluded | Irrelevant, did not discuss the subject matter |
| 832 | Analysis of the causes of cervical lymphadenopathy using fine-needle aspiration cytology combining cell block in Chinese patients with and without HIV infection. | Sun L; Zhang L; Yang K; Chen XM; Chen JM; Xiao J; Zhao HX; Ma ZY; Qi LM; Wang P | 2020 | Excluded | Irrelevant, did not discuss the subject matter |
| 833 | Simplified dolutegravir dosing for children with HIV weighing 20 kg or more: pharmacokinetic and safety substudies of the multicentre, randomised ODYSSEY trial. | Bollen PDJ; Moore CL; Mujuru HA; Makumbi S; Kekitiinwa AR; Kaudha E; Parker A; Musoro G; Nanduudu A; Lugemwa A; Amuge P; Hakim JG; Rojo P; Giaquinto C; Colbers A; Gibb DM; Ford D; Turkova A; Burger DM | 2020 | Excluded | Irrelevant, did not discuss the subject matter |
| 834 | Serious fungal infections in Ecuador. | Zurita J; Denning DW; Paz-Y-MiÃ±o A; SolÃ­s MB; Arias LM | 2017 | Excluded | Irrelevant, did not discuss the subject matter |
| 835 | Preemptive Therapy for Cryptococcal Meningitis: A Valid Strategy for Latin America? | Vidal JE | 2016 | Excluded | Irrelevant, did not discuss the subject matter |
| 836 | Mortality due to systemic mycoses as a primary cause of death or in association with AIDS in Brazil: a review from 1996 to 2006. | Prado M; Silva MB; Laurenti R; Travassos LR; Taborda CP | 2009 | Excluded | Irrelevant, did not discuss the subject matter |
| 837 | Efficacy of Oral Encochleated Amphotericin B in a Mouse Model of Cryptococcal Meningoencephalitis. | Lu R; Hollingsworth C; Qiu J; Wang A; Hughes E; Xin X; Konrath KM; Elsegeiny W; Park YD; Atakulu L; Craft JC; Tramont EC; Mannino R; Williamson PR | 2019 | Excluded | Irrelevant, did not discuss the subject matter |
| 838 | Fever in the tropics: aetiology and case-fatality - a prospective observational study in a tertiary care hospital in South India. | Abrahamsen SK; Haugen CN; Rupali P; Mathai D; Langeland N; Eide GE; MÃ¸rch K | 2013 | Excluded | Irrelevant, did not discuss the subject matter |
| 839 | Barriers to starting ART and how they can be overcome: individual and operational factors associated with early and late start of treatment. | Parkes-Ratanshi R; Bufumbo L; Nyanzi-Wakholi B; Levin J; Grosskurth H; Lalloo DG; Kamali A | 2010 | Excluded | Irrelevant, did not discuss the subject matter |
| 840 | [Causes of lymphocytic meningitis in people with HIV admitted to the Infectious Disease department of Conakry]. | TraorÃ© FA; Cissoko Y; Tounkara TM; Sako FB; Mouelle AD; Kpami DO; TraorÃ© M; Doumbouya M | 2015 | Excluded | Irrelevant, did not discuss the subject matter |
| 841 | [Clinical characteristics of 143 Chinese HIV/AIDS patients]. | Dai Y; Li TS; Wang AX; Qiu ZF; Xie J; Han Y; Liu ZY; Ma XJ; Wang HL; Fan HW; Zuo LY; Li YL; Deng GH; Sheng RY | 2006 | Excluded | Irrelevant, did not discuss the subject matter |
| 842 | District and sub-district analysis of cryptococcal antigenaemia prevalence and specimen positivity in KwaZulu-Natal, South Africa. | Cassim N; Coetzee LM; Govender NP; Glencross DK | 2018 | Excluded | Irrelevant, did not discuss the subject matter |
| 843 | Biopsy of peripheral lymph nodes: a useful tool to diagnose opportunistic diseases in HIV-infected patients. | Ramos CG; Goldani LZ | 2011 | Excluded | Irrelevant, did not discuss the subject matter |
| 844 | Central Nervous System Cryptococcosis among a Cohort of HIV Infected Patients from a University Hospital of North India. | Nigam C; Gahlot R; Kumar V; Chakravarty J; Tilak R | 2012 | Excluded | Irrelevant, did not discuss the subject matter |
| 845 | The Changing Epidemiology of HIV-Associated Adult Meningitis, Uganda 2015-2017. | Ellis J; Bangdiwala AS; Cresswell FV; Rhein J; Nuwagira E; Ssebambulidde K; Tugume L; Rajasingham R; Bridge SC; Muzoora C; Meya DB; Boulware DR | 2019 | Excluded | Irrelevant, did not discuss the subject matter |
| 846 | [Efficacy of the treatment and secondary antifungal prophylaxis in AIDS-related histoplasmosis. Experience at the Francisco J. MuÃ±iz Infectious Diseases Hospital in Buenos Aires]. | Negroni R; Messina F; Arechavala A; Santiso G; Bianchi M | 2017 | Excluded | Irrelevant, did not discuss the subject matter |
| 847 | Cryptococcal antigen among HIV1-infected individuals in north-central Nigeria. | Ezenabike C; S Ashaka O; A Omoare A; Fadeyi A; K Salami A; O Agbede O | 2020 | Excluded | Irrelevant, did not discuss the subject matter |
| 848 | Healthcare utilization of patients accessing an African national treatment program. | Harling G; Orrell C; Wood R | 2007 | Excluded | Irrelevant, did not discuss the subject matter |
| 849 | Cryptococcosis in Colombia: Compilation and Analysis of Data from Laboratory-Based Surveillance. | EscandÃ³n P; Lizarazo J; Agudelo CI; CastaÃ±eda E | 2018 | Excluded | Irrelevant, did not discuss the subject matter |
| 850 | Epidemiology, seasonality, and predictors of outcome of AIDS-associated Penicillium marneffei infection in Ho Chi Minh City, Viet Nam. | Le T; Wolbers M; Chi NH; Quang VM; Chinh NT; Lan NP; Lam PS; Kozal MJ; Shikuma CM; Day JN; Farrar J | 2011 | Excluded | Irrelevant, did not discuss the subject matter |
| 851 | Serious fungal infections in the Philippines. | Batac MCR; Denning D | 2017 | Excluded | Irrelevant, did not discuss the subject matter |
| 852 | Ten years of antiretroviral therapy: Incidences, patterns and risk factors of opportunistic infections in an urban Ugandan cohort. | Weissberg D; Mubiru F; Kambugu A; Fehr J; Kiragga A; von Braun A; Baumann A; Kaelin M; Sekaggya-Wiltshire C; Kamya M; Castelnuovo B | 2018 | Excluded | Irrelevant, did not discuss the subject matter |
| 853 | Antibody Responses in HIV-Infected Patients With Advanced Immunosuppression and Asymptomatic Cryptococcal Antigenemia. | Hlupeni A; Nakouzi A; Wang T; Boyd KF; Makadzange TA; Ndhlovu CE; Pirofski LA | 2019 | Excluded | Irrelevant, did not discuss the subject matter |
| 854 | Cryptococcus neoformans and Cryptococcus gattii clinical isolates from Thailand display diverse phenotypic interactions with macrophages. | Hansakon A; Mutthakalin P; Ngamskulrungroj P; Chayakulkeeree M; Angkasekwinai P | 2019 | Excluded | Irrelevant, did not discuss the subject matter |
| 855 | Evaluation of a Cryptococcal Antigen Lateral Flow Assay and Cryptococcal Antigen Positivity at a Large Public Hospital in Atlanta, Georgia. | Harrington KRV; Wang YF; Rebolledo PA; Liu Z; Yang Q; Kempker RR | 2021 | Excluded | Irrelevant, did not discuss the subject matter |
| 856 | Incidence and risk factors for the immune reconstitution inflammatory syndrome in HIV patients in South Africa: a prospective study. | Murdoch DM; Venter WD; Feldman C; Van Rie A | 2008 | Excluded | Irrelevant, did not discuss the subject matter |
| 857 | Molecular diagnosis of central nervous system opportunistic infections and mortality in HIV-infected adults in Central China. | Yang R; Zhang H; Xiong Y; Gui X; Zhang Y; Deng L; Gao S; Luo M; Hou W; Guo D | 2017 | Excluded | Irrelevant, did not discuss the subject matter |
| 858 | Diagnosing HIV-associated cerebral diseases - the importance of Neuropathology in understanding HIV. | Diaconu IA; Stratan LM; Nichita L; AramÄƒ V; Moroti Constantinescu VR; Diaconu AI; Ion DA | 2016 | Excluded | Irrelevant, did not discuss the subject matter |
| 859 | The Burden of Serious Fungal Infections in Cameroon. | Mandengue CE; Denning DW | 2018 | Excluded | Irrelevant, did not discuss the subject matter |
| 860 | Estimation of the Burden of Serious Human Fungal Infections in Malaysia. | Velayuthan RD; Samudi C; Lakhbeer Singh HK; Ng KP; Shankar EM; Denning DW | 2018 | Excluded | Irrelevant, did not discuss the subject matter |
| 861 | Management of HIV in resource limited settings. | McCutchan JA | 2009 | Excluded | Irrelevant, did not discuss the subject matter |
| 862 | Prevalence of Cryptococcal Antigenemia and Associated Factors among HIV/AIDS Patients at Felege-Hiwot Referral Hospital, Bahir Dar, Northwest Ethiopia. | Jemal M; Deress T; Belachew T; Adem Y | 2021 | Excluded | Irrelevant, did not discuss the subject matter |
| 863 | Diagnosis of coccidioidomycosis in a non-endemic area: Inference of the probable geographic area of an infection. | FernÃ¡ndez R; Arenas R; Duarte-Escalante E; FrÃ­as-De LeÃ³n MG; Vega Memige ME; Acosta Altamirano G; Reyes-Montes MDR | 2017 | Excluded | Irrelevant, did not discuss the subject matter |
| 864 | Disseminated cryptococcosis in a deceased with HIV-1 diagnosed by minimally invasive tissue sampling technique. | Subedi N; Bhattarai S; Ranabhat S; Sharma BK; Baral MP; Upadhyaya TL | 2021 | Excluded | Irrelevant, did not discuss the subject matter |
| 865 | Seizures in HIV-seropositive individuals: NIMHANS experience and review. | Satishchandra P; Sinha S | 2008 | Excluded | Irrelevant, did not discuss the subject matter |
| 866 | Cryptococcal meningitis after ART: Need for proper baseline evaluation in the era of 'Test & Treat'. | Akase IE; Olowoyo O; Oladele RO; Obiako RO; Warris A; Akanmu SA | 2019 | Excluded | Irrelevant, did not discuss the subject matter |
| 867 | Burden of serious fungal infections in Bangladesh. | Gugnani HC; Denning DW; Rahim R; Sadat A; Belal M; Mahbub MS | 2017 | Excluded | Irrelevant, did not discuss the subject matter |
| 868 | Pulmonary and Extrapulmonary Manifestations of Fungal Infections Misdiagnosed as Tuberculosis: The Need for Prompt Diagnosis and Management. | Ekeng BE; Davies AA; Osaigbovo II; Warris A; Oladele RO; Denning DW | 2022 | Excluded | Irrelevant, did not discuss the subject matter |
| 869 | ISOLATION OF Cryptococcus neoformans FROM ENVIRONMENTAL SAMPLES COLLECTED IN SOUTHEASTERN NIGERIA. | Nweze EI; Kechia FA; Dibua UE; Eze C; Onoja US | 2015 | Excluded | Irrelevant, did not discuss the subject matter |
| 870 | Cryptococcus neoformans Causing Meningoencephalitis in Adults and a Child from Lima, Peru: Genotypic Diversity and Antifungal Susceptibility. | Firacative C; Zuluaga-Puerto N; Guevara J | 2022 | Excluded | Irrelevant, did not discuss the subject matter |
| 871 | Cerebrospinal Fluid Culture Positivity and Clinical Outcomes After Amphotericin-Based Induction Therapy for Cryptococcal Meningitis. | Rolfes MA; Rhein J; Schutz C; Taseera K; Nabeta HW; Huppler Hullsiek K; Akampuira A; Rajasingham R; Musubire A; Williams DA; Thienemann F; Bohjanen PR; Muzoora C; Meintjes G; Meya DB; Boulware DR | 2015 | Excluded | Irrelevant, did not discuss the subject matter |
| 872 | Clinical features and para-clinical findings of cryptococcal meningitis in the North of Iran during 2011-19. | Babamahmoodi F; Gerizade Firozjaii K; Bayani M; Shokohi T; Yazdani J; Beyzaee AM; Ahangarkani F | 2020 | Excluded | Irrelevant, did not discuss the subject matter |
| 873 | Spectrums of opportunistic infections and malignancies in HIV-infected patients in tertiary care hospital, China. | Xiao J; Gao G; Li Y; Zhang W; Tian Y; Huang Y; Su W; Han N; Yang D; Zhao H | 2013 | Excluded | Irrelevant, did not discuss the subject matter |
| 874 | Estimated Incidence and Prevalence of Serious Fungal Infections in Morocco. | Lmimouni BE; Hennequin C; Penney ROS; Denning DW | 2022 | Excluded | Irrelevant, did not discuss the subject matter |
| 875 | Diagnostic value of latex agglutination in cryptococcal meningitis. | Dominic RS; Prashanth H; Shenoy S; Baliga S | 2009 | Excluded | Irrelevant, did not discuss the subject matter |
| 876 | [Neuromeningeal cryptococcosis in patients infected with HIV at Agadir regional hospital, (Souss-Massa, Morocco)]. | Chadli S; Aghrouch M; Taqarort N; Malmoussi M; Ouagari Z; Moustaoui F; Bourouache M; Oulkheir S | 2018 | Excluded | Irrelevant, did not discuss the subject matter |
| 877 | Burden of fungal infections in Kenya. | Ratemo SN; Denning DW | 2023 | Excluded | Irrelevant, did not discuss the subject matter |
| 878 | Mortality Patterns of Toxoplasmosis and Its Comorbidities in Tanzania: A 10-Year Retrospective Hospital-Based Survey. | Mboera LEG; Kishamawe C; Kimario E; Rumisha SF | 2019 | Excluded | Irrelevant, did not discuss the subject matter |
| 879 | [Development of AIDS-related malignancies and infections after starting HAART]. | Warley E; Tamayo Antabak N; Desse J; De Luca A; Warley F; FernÃ¡ndez Galimberti G; D'Agostino G; Quintas L; Szyld E | 2010 | Excluded | Irrelevant, did not discuss the subject matter |
| 880 | Fungal Opportunistic Pneumonias in HIV/AIDS Patients: An Indian Tertiary Care Experience. | Kaur R; Mehra B; Dhakad MS; Goyal R; Bhalla P; Dewan R | 2017 | Excluded | Irrelevant, did not discuss the subject matter |
| 881 | The Burden of Fungal Diseases in Romania. | MareÈ™ M; Moroti-Constantinescu VR; Denning DW | 2018 | Excluded | Irrelevant, did not discuss the subject matter |
| 882 | COVID-19, HIV, and Cryptococcal Meningitis Coinfections with Abnormal Laboratory Findings. | Aghamali M; Kazemi A; Asgharzadeh M; Kafil HS | 2023 | Excluded | Irrelevant, did not discuss the subject matter |
| 883 | Serious fungal infections in Egypt. | Zaki SM; Denning DW | 2017 | Excluded | Irrelevant, did not discuss the subject matter |
| 884 | Histological spectrum of liver in HIV - autopsy study. | Amarapurkar AD; Sangle NA | 2005 | Excluded | Irrelevant, did not discuss the subject matter |
| 885 | Estimated Burden of Serious Fungal Infections in Ghana. | Ocansey BK; Pesewu GA; Codjoe FS; Osei-Djarbeng S; Feglo PK; Denning DW | 2019 | Excluded | Irrelevant, did not discuss the subject matter |
| 886 | [Pulmonary mycosis in patients with diabetes mellitus. Clinical characteristics and risk factors]. | HernÃ¡ndez-SolÃ­s A; Camerino Guerrero A; ColÃ­n MuÃ±oz Y; BazÃ¡n Cuervo S; CÃ­cero Sabido R; Reding-Bernal A | 2020 | Excluded | Irrelevant, did not discuss the subject matter |
| 887 | Estimated economic losses of hospitalized AIDS patients at Siriraj Hospital from January 2003 to December 2003: time for aggressive voluntary counseling and HIV testing. | Ratanasuwan W; Anekthananon T; Techasathit W; Rongrungruang Y; Sonjai A; Suwanagool S | 2005 | Excluded | Irrelevant, did not discuss the subject matter |
| 888 | Clinical Features of Cryptococcal Meningoencephalitis in HIV-Positive and -Negative Patients in a Resource-Limited Setting. | Kajeekul R; Mekawichai P; Chayakulkeeree M | 2023 | Excluded | Irrelevant, did not discuss the subject matter |
| 889 | A Journey of Hope: giving research participants a voice to share theirÂ experiences and improve community engagement aroundÂ advanced HIV disease in Uganda. | Cresswell FV; Kasibante J; Martyn EM; Tugume L; Stead G; Ssembambulidde K; Rutakingirwa MK; Kagimu E; Nsangi L; Namuju C; Ndyetukira JF; Ahimbisibwe C; Kugonza F; Sadiq A; Namudde A; Dobbin J; Srishyla D; Quinn C; Kabahubya M; Muzoora C; Watiti S; Meya DB; Elliott AM | 2020 | Excluded | Irrelevant, did not discuss the subject matter |
| 890 | Spectrum of Opportunistic Infections and Risk Factors for In-Hospital Mortality of Admitted AIDS Patients in Shanghai. | Luo B; Sun J; Cai R; Shen Y; Liu L; Wang J; Zhang R; Shen J; Lu H | 2016 | Excluded | Irrelevant, did not discuss the subject matter |
| 891 | Cryptococcal Meningitis: A Retrospective Cohort of a Brazilian Reference Hospital in the Post-HAART Era of Universal Access. | de Azambuja AZ; Wissmann Neto G; Watte G; Antoniolli L; Goldani LZ | 2018 | Excluded | Irrelevant, did not discuss the subject matter |
| 892 | Environmental isolation, biochemical identification, and antifungal drug susceptibility of Cryptococcus species. | Teodoro VL; Gullo FP; Sardi Jde C; Torres EM; Fusco-Almeida AM; Mendes-Giannini MJ | 2013 | Excluded | Irrelevant, did not discuss the subject matter |
| 893 | Re-estimation of the burden of serious fungal diseases in Uganda. | Bongomin F; Kwizera R; Namusobya M; van Rhijn N; Andia-Biraro I; Kirenga BJ; Meya DB; Denning DW | 2024 | Excluded | Irrelevant, did not discuss the subject matter |
| 894 | Diagnostic Mycology Laboratories Should Have a Central Role for the Management of Fungal Disease. | Medina N; Alastruey-Izquierdo A; Mercado D; Denning DW; Arathoon E; Rodriguez-Tudela JL | 2022 | Excluded | Irrelevant, did not discuss the subject matter |
| 895 | Spectrum of central nervous system infections in a tertiary health care centre in Cameroon. | Gams Massi D; Mintyene Mintyene MRR; Magnerou AM; Eko SM; Kenmegne C; Mbahe S; Sounga Bandzouzi PE; Mbatchou Ngahane HB; Mapoure NY | 2022 | Excluded | Irrelevant, did not discuss the subject matter |
| 896 | Incidence of WHO stage 3 and 4 conditions following initiation of anti-retroviral therapy in resource limited settings. | Curtis AJ; Marshall CS; Spelman T; Greig J; Elliot JH; Shanks L; Du Cros P; Casas EC; Da Fonseca MS; O'Brien DP | 2012 | Excluded | Irrelevant, did not discuss the subject matter |
| 897 | Clinical characteristics and morbidity among hospitalized adults with advanced HIV disease in Uganda during 'test and treat' era. | Tugume L; Semitala FC; Owachi D; Kagimu E; Kamya MR; Meya DB | 2023 | Excluded | Irrelevant, did not discuss the subject matter |
| 898 | Comparison of Antigen Detection and Nested PCR in CSF Samples of HIV Positive and Negative Patients with Suspected Cryptococcal Meningitis in a Tertiary Care Hospital. | Kumari S; Verma RK; Singh DP; Yadav R | 2016 | Excluded | Irrelevant, did not discuss the subject matter |
| 899 | Lack of Association between Fluconazole Susceptibility and ERG11 Nucleotide Polymorphisms in Cryptococcus neoformans Clinical Isolates from Uganda. | Atim PB; Meya DB; Gerlach ES; Muhanguzi D; Male A; Kanamwanji B; Nielsen K | 2022 | Excluded | Irrelevant, did not discuss the subject matter |
| 900 | Cerebrospinal T-cell responses aid in the diagnosis of tuberculous meningitis in a human immunodeficiency virus- and tuberculosis-endemic population. | Patel VB; Singh R; Connolly C; Coovadia Y; Peer AK; Parag P; Kasprowicz V; Zumla A; Ndung'u T; Dheda K | 2010 | Excluded | Irrelevant, did not discuss the subject matter |
| 901 | Estimation of CD4+ and CD8+ T-lymphocytes in human immunodeficiency virus infection and acquired immunodeficiency syndrome patients in Manipur. | Singh HR; Singh NG; Singh TB | 2007 | Excluded | Irrelevant, did not discuss the subject matter |
| 902 | Neurological worsening during treatment of an immunocompetent adult with Cryptococcus neoformans meningitis. | Tanu S; Mihir M; Rajeev S; Annu A | 2020 | Excluded | Irrelevant, did not discuss the subject matter |
| 903 | Diagnostic accuracy of Xpert MTB/RIF Ultra for tuberculous meningitis in HIV-infected adults: a prospective cohort study. | Bahr NC; Nuwagira E; Evans EE; Cresswell FV; Bystrom PV; Byamukama A; Bridge SC; Bangdiwala AS; Meya DB; Denkinger CM; Muzoora C; Boulware DR | 2018 | Excluded | Irrelevant, did not discuss the subject matter |
| 904 | High Mortality in HIV-Associated Cryptococcal Meningitis Patients Treated With Amphotericin B-Based Therapy Under Routine Care Conditions in Africa. | Patel RKK; Leeme T; Azzo C; Tlhako N; Tsholo K; Tawanana EO; Molefi M; Mosepele M; Lawrence DS; Mokomane M; Tenforde MW; Jarvis JN | 2018 | Excluded | Irrelevant, did not discuss the subject matter |
| 905 | Predictors of dropout from care among HIV-infected patients initiating antiretroviral therapy at a public sector HIV treatment clinic in sub-Saharan Africa. | Asiimwe SB; Kanyesigye M; Bwana B; Okello S; Muyindike W | 2016 | Excluded | Irrelevant, did not discuss the subject matter |
| 906 | Disseminated nontuberculous mycobacterial infection in patients who are not infected with HIV in Thailand. | Chetchotisakd P; Kiertiburanakul S; Mootsikapun P; Assanasen S; Chaiwarith R; Anunnatsiri S | 2007 | Excluded | Irrelevant, did not discuss the subject matter |
| 907 | Global research priorities for infections that affect the nervous system. | John CC; Carabin H; Montano SM; Bangirana P; Zunt JR; Peterson PK | 2015 | Excluded | Irrelevant, did not discuss the subject matter |
| 908 | Challenges in diagnosis, treatment and follow-up of patients presenting with central nervous system infections in a resource-limited setting. | Leligdowicz A; Katwere M; Piloya T; Ronald A; Kambugu A; Katabira E | 2006 | Excluded | Irrelevant, did not discuss the subject matter |
| 909 | Prevalence and clinical presentation of Cryptococcal meningitis among HIV seropositive patients. | Baradkar V; Mathur M; De A; Kumar S; Rathi M | 2009 | Excluded | Irrelevant, did not discuss the subject matter |
| 910 | Profile and mortality outcome of patients admitted with cryptococcal meningitis to an urban district hospital in KwaZulu-Natal, South Africa. | Adeyemi B; Ross A | 2014 | Excluded | Irrelevant, did not discuss the subject matter |
| 911 | Characteristics of Invasive Fungal Infections among HIV Individuals from an Indigenous Origin in Mexico. | Aranda-Audelo M; Rivera-MartÃ­nez NE; Corzo-LeÃ³n DE | 2018 | Excluded | Irrelevant, did not discuss the subject matter |
| 912 | Patterns and trends in mortality among HIV-infected and HIV-uninfected patients in a major Internal Medicine Unit in YaoundÃ©, Cameroon: a retrospective cohort study. | Mbuagbaw J; Jingi AM; Noubiap JJ; Kaze AD; Nansseu JR; Bigna JJ; Wawo Yonta E; Ngu Blackett K | 2016 | Excluded | Irrelevant, did not discuss the subject matter |
| 913 | Penicillium marneffei infection: an AIDS-defining illness. | Maniar JK; Chitale AR; Miskeen A; Shah K; Maniar A | 2005 | Excluded | Irrelevant, did not discuss the subject matter |
| 914 | Clinical presentation, management, and outcome of suspected central nervous system infections in Indonesia: a prospective cohort study. | Maharani K; Dian S; Ganiem AR; Imran D; Estiasari R; Ardiansyah E; Andini PW; Kristina F; Pangeran D; Chaidir L; Alisjahbana B; Rukmana A; Kusumaningrum A; Adawiyah R; Subekti D; Yunihastuti E; Yunus RE; Waslia L; van Ingen J; van Laarhoven A; Hamers RL; van Crevel R | 2024 | Excluded | Irrelevant, did not discuss the subject matter |
| 915 | The Burden of Fungal Infections in Ethiopia. | Tufa TB; Denning DW | 2019 | Excluded | Irrelevant, did not discuss the subject matter |
| 916 | Cryptococcus gattii meningitis in a diabetic adult in South India. | Jha MK; Mohanty A; Gupta P | 2019 | Excluded | Irrelevant, did not discuss the subject matter |
| 917 | Reversible Deafness and Blindness in a Patient with Cryptococcal Meningitis in Tanzania. | Douglas-Vail M; Bechamp T; Gohal S; Soegtrop R; Vitali S; Rugemalila J; Stone NR | 2015 | Excluded | Irrelevant, did not discuss the subject matter |
| 918 | The epidemiology and clinical spectrum of infections of the central nervous system in adults in north India. | Kumar D; Pannu AK; Dhibar DP; Singh R; Kumari S | 2021 | Excluded | Irrelevant, did not discuss the subject matter |
| 919 | The pattern and predictors of mortality of HIV/AIDS patients with neurologic manifestation in Ethiopia: a retrospective study. | Berhe T; Melkamu Y; Amare A | 2012 | Excluded | Irrelevant, did not discuss the subject matter |
| 920 | Chronic disseminated cryptococcosis without meningeal involvement in a severely immunosuppressed HIV-infected patient successfully treated with fluconazole. | Yonekura TDS; Macedo SD; Moya FG; Vidal JE | 2022 | Excluded | Irrelevant, did not discuss the subject matter |
| 921 | Retrospective Study of Cryptococcal Meningitis With Elevated Minimum Inhibitory Concentration to Fluconazole in Immunocompromised Patients. | Nasri H; Kabbani S; Bou Alwan M; Wang YF; Rebolledo PA; Kraft CS; Nguyen ML; Anderson AM; Rouphael N | 2016 | Excluded | Irrelevant, did not discuss the subject matter |
| 922 | Independent Risk Factors for Deaths due to AIDS in Chongqing, China: Does Age Matter? | Zhou Y; Yang Z; Liu M; Lu Y; Qin Y; He X; Zeng Y; Harypursat V; Chen Y | 2020 | Excluded | Irrelevant, did not discuss the subject matter |
| 923 | Case of moyamoya disease in a patient with advanced acquired immunodeficiency syndrome. | Sharfstein SR; Ahmed S; Islam MQ; Najjar MI; Ratushny V | 2007 | Excluded | Irrelevant, did not discuss the subject matter |
| 924 | Meningitis Caused by Rhodotorula mucilaginosa in HIV-Infected Patient: A Case Report and Review of the Literature. | Mohd Nor F; Tan LH; Na SL; Ng KP | 2015 | Excluded | Irrelevant, did not discuss the subject matter |
| 925 | Burden of fungal infections in Iran. | Hedayati MT; Taghizadeh Armaki M; Yazdani Charati J; Hedayati N; Seyedmousavi S; Denning DW | 2018 | Excluded | Irrelevant, did not discuss the subject matter |
| 926 | 5-Flucytosine Longitudinal Antifungal Susceptibility Testing of Cryptococcus neoformans: A Substudy of the EnACT Trial Testing Oral Amphotericin. | McHale TC; Akampurira A; Gerlach ES; Mucunguzi A; Nicol MR; Williams DA; Nielsen K; Bicanic T; Fieberg A; Dai B; Meya DB; Boulware DR | 2023 | Excluded | Irrelevant, did not discuss the subject matter |
| 927 | Invasive trichosporonosis in an AIDS patient: case report and review of the literature. | Roman AD; SalvaÃ±a EM; Guzman-PeÃ±amora MA; Roxas EA; Leyritana KT; Saniel MC | 2014 | Excluded | Irrelevant, did not discuss the subject matter |
| 928 | Impact of the National Access to Antiretroviral Program on the incidence of opportunistic infections in Thailand. | Rojanawiwat A; Tsuchiya N; Pathipvanich P; Pumpradit W; Schmidt WP; Honda S; Auwanit W; Sawanpanyalert P; Ariyoshi K | 2011 | Excluded | Irrelevant, did not discuss the subject matter |
| 929 | Molecular diversity of serial Cryptococcus neoformans isolates from AIDS patients in the city of SÃ£o Paulo, Brazil. | Martins MA; Pappalardo MC; Melhem MS; Pereira-Chioccola VL | 2007 | Excluded | Irrelevant, did not discuss the subject matter |
| 930 | The spectrum of clinical and pathological manifestations of AIDS in a consecutive series of 236 autopsied cases in mumbai, India. | Lanjewar DN | 2011 | Excluded | Irrelevant, did not discuss the subject matter |
| 931 | Central nervous system cryptococcoma in a Ugandan patient with Human Immunodeficiency Virus. | Velamakanni SS; Bahr NC; Musubire AK; Boulware DR; Rhein J; Nabeta HW | 2014 | Excluded | Irrelevant, did not discuss the subject matter |
| 932 | Clinical Presentation, Aetiology, and Outcomes of Meningitis in a Setting of High HIV and TB Prevalence. | Thinyane KH; Motsemme KM; Cooper VJ | 2015 | Excluded | Irrelevant, did not discuss the subject matter |
| 933 | Impact of community engagement and social support on the outcomes of HIV-related meningitis clinical trials in a resource-limited setting. | Kwizera R; Sadiq A; Ndyetukira JF; Nalintya E; Williams D; Rhein J; Boulware DR; Meya DB | 2020 | Excluded | Irrelevant, did not discuss the subject matter |
| 934 | Cryptococcus gatti serotype B isolated in Sikkim (North-East India)-A new geographical niche. | Gurung S; Sherpa NT; Yoden Bhutia P; Pradhan J; Peralam Yegneshwaran P | 2012 | Excluded | Irrelevant, did not discuss the subject matter |
| 935 | Cryptococcal Meningitis Masquerading as Normal Pressure Hydrocephalus in an Immune-competent Adult. | Raheja H; Sinha A; Irukulla PK; Kupfer Y | 2017 | Excluded | Irrelevant, did not discuss the subject matter |
| 936 | Liposomal Amphotericin B for the Treatment of Cryptococcal Meningitis in HIV/AIDS Patients in India-A Pilot Pharmacokinetic Study. | Jadhav M; Jadhav P; Shinde V; Kadam P; Kshirsagar N | 2013 | Excluded | Irrelevant, did not discuss the subject matter |
| 937 | Epidemiology and identification of potential fungal pathogens causing invasive fungal infections in a tertiary care hospital in northeast Thailand. | Faksri K; Kaewkes W; Chaicumpar K; Chaimanee P; Wongwajana S | 2014 | Excluded | Irrelevant, did not discuss the subject matter |
| 938 | Prevalence and Associated Factors of Cryptococcal Antigenemia in HIV-Infected Patients with CD4 < 200 Cells/ÂµL in SÃ£o Paulo, Brazil: A Bayesian Analysis. | Mimicos EV; Fossaluza V; Picone CM; de Sena CC; Gomes HR; LÃ¡zari CDS; Silva FFD; Nakanishi ES; Nisida IV; Freitas AC; Gryschek RB; Lagonegro ER; LazÃ©ra M; Shikanai-Yasuda MA | 2022 | Excluded | Irrelevant, did not discuss the subject matter |
| 939 | Etiology of Fever and Associated Outcomes Among Adults Receiving Chemotherapy for the Treatment of Solid Tumors in Uganda. | Gulleen EA; Holte S; Zhang Y; Mbarusha I; Mubiru D; Pedun B; Keng M; Heysell SK; Omoding A; Moore CC; Phipps W | 2023 | Excluded | Irrelevant, did not discuss the subject matter |
| 940 | Variables that influence HIV-1 cerebrospinal fluid viral load in cryptococcal meningitis: a linear regression analysis. | Cecchini DM; CaÃ±izal AM; Rojas H; Arechavala A; Negroni R; Bouzas MB; Benetucci JA | 2009 | Excluded | Irrelevant, did not discuss the subject matter |
| 941 | Pattern, Clinical Characteristics, and Outcome of Meningitis among HIV-Infected Adults Admitted in a Tertiary Hospital in North Western Tanzania: A Cross-Sectional Study. | Boaz MM; Kalluvya S; Downs JA; Mpondo BC; Mshana SE | 2016 | Excluded | Irrelevant, did not discuss the subject matter |
| 942 | Trends in antifungal drug susceptibility of Cryptococcus neoformans isolates obtained through population-based surveillance in South Africa in 2002-2003 and 2007-2008. | Govender NP; Patel J; van Wyk M; Chiller TM; Lockhart SR | 2011 | Excluded | Irrelevant, did not discuss the subject matter |
| 943 | First report of urease activity in the novel systemic fungal pathogen Emergomyces africanus: a comparison with the neurotrope Cryptococcus neoformans. | Lerm B; Kenyon C; Schwartz IS; Kroukamp H; de Witt R; Govender NP; de Hoog GS; Botha A | 2017 | Excluded | Irrelevant, did not discuss the subject matter |
| 944 | Characterization of clinical isolates of the Cryptococcus neoformans-Cryptococcus gattii species complex from the Amazonas State in Brazil. | Da Silva BK; Freire AK; Bentes Ados S; Sampaio Ide L; Santos LO; Dos Santos MS; De Souza JV | 2012 | Excluded | Irrelevant, did not discuss the subject matter |
| 945 | Can improved diagnostics reduce mortality from Tuberculous meningitis? Findings from a 6.5-year cohort in Uganda. | Cresswell FV; Bangdiwala AS; Bahr NC; Trautner E; Nuwagira E; Ellis J; Rajasingham R; Rhein J; Williams DA; Muzoora C; Elliott AM; Meya DB; Boulware DR | 2018 | Excluded | Irrelevant, did not discuss the subject matter |
| 946 | Human immunodefeciency virus associated cryptococcal meningitis at a tertiary care centre: diagnostic tools and antifungal susceptibility testing. | Munivenkataswamy R; Gopi A; Usman SM; Jagadeesh | 2013 | Excluded | Irrelevant, did not discuss the subject matter |
| 947 | [Parasitic and fungal neuroinfections at the Infectious Diseases Clinic in Fann Teaching Hospital in Dakar]. | SoumarÃ© M; Seydi M; Diop SA; Ndour CT; Faye N; Fall N; Dieng Y; Diop BM; Sow PS | 2009 | Excluded | Irrelevant, did not discuss the subject matter |
| 948 | Cryptococcal meningitis: epidemiology and therapeutic options. | Sloan DJ; Parris V | 2014 | Excluded | Irrelevant, did not discuss the subject matter |
| 949 | Adult-onset immunodeficiency in Thailand and Taiwan. | Browne SK; Burbelo PD; Chetchotisakd P; Suputtamongkol Y; Kiertiburanakul S; Shaw PA; Kirk JL; Jutivorakool K; Zaman R; Ding L; Hsu AP; Patel SY; Olivier KN; Lulitanond V; Mootsikapun P; Anunnatsiri S; Angkasekwinai N; Sathapatayavongs B; Hsueh PR; Shieh CC; Brown MR; Thongnoppakhun W; Claypool R; Sampaio EP; Thepthai C; Waywa D; Dacombe C; Reizes Y; Zelazny AM; Saleeb P; Rosen LB; Mo A; Iadarola M; Holland SM | 2012 | Excluded | Irrelevant, did not discuss the subject matter |
| 950 | Microbiological, Epidemiological, and Clinical Characteristics of Patients With Cryptococcal Meningitis at a Tertiary Hospital in China: A 6-Year Retrospective Analysis. | Li Y; Zou M; Yin J; Liu Z; Lu B | 2020 | Excluded | Irrelevant, did not discuss the subject matter |
| 951 | A systematic literature review on the prevalence and etiology of meningitis among critically ill and hospitalized patients in India. | Ghia CJ; Rambhad GS | 2021 | Excluded | Irrelevant, did not discuss the subject matter |
| 952 | Disparities in the Magnitude of Human Immunodeficiency Virus-related Opportunistic Infections Between High and Low/Middle-income Countries: Is Highly Active Antiretroviral Therapy Changing the Trend? | Iroezindu MO | 2016 | Excluded | Irrelevant, did not discuss the subject matter |
| 953 | Prevalence of pulmonary mycoses in smear-negative patients with suspected tuberculosis in the Brazilian Amazon. | Matsuda JDS; Wanke B; Balieiro AADS; Santos CSDS; Cavalcante RCDS; Muniz MM; Torres DR; Pinheiro SB; Frickmann H; Souza JVB; Martinez-Espinosa FE | 2021 | Excluded | Irrelevant, did not discuss the subject matter |
| 954 | The spectrum of pathologic lesions in spleen in patients with acquired immunodeficiency syndrome: Autopsy report of 257 patients. | Lanjewar DN; Kavatkar AN | 2023 | Excluded | Irrelevant, did not discuss the subject matter |
| 955 | Nontuberculous mycobacterial infections in King Chulalongkorn Memorial Hospital. | Saritsiri S; Udomsantisook N; Suankratay C | 2006 | Excluded | Irrelevant, did not discuss the subject matter |
| 956 | Cryptococcal Meningitis: Differences between Patients with and without HIV-Infection. | Teekaput C; Yasri S; Chaiwarith R | 2023 | Excluded | Irrelevant, did not discuss the subject matter |
| 957 | [Not Available]. | Ditondo P; Luemba A; Chuy RI; Mucinya G; Ade S | 2023 | Excluded | Irrelevant, did not discuss the subject matter |
| 958 | Population diversity and virulence characteristics of Cryptococcus neoformans/C. gattii species complexes isolated during the pre-HIV-pandemic era. | Pharkjaksu S; Kwon-Chung KJ; Bennett JE; Ngamskulrungroj P | 2020 | Excluded | Irrelevant, did not discuss the subject matter |
| 959 | Burden of Serious Fungal Infections in India. | Ray A; Aayilliath K A; Banerjee S; Chakrabarti A; Denning DW | 2022 | Excluded | Irrelevant, did not discuss the subject matter |
| 960 | The performance of serum cryptococcal capsular polysaccharide antigen test, histopathology and culture of the lung tissue for diagnosis of pulmonary cryptococcosis in patients without HIV infection. | Zhou Y; Lin PC; Ye JR; Su SS; Dong L; Wu Q; Xu HY; Xie YP; Li YP | 2018 | Excluded | Irrelevant, did not discuss the subject matter |
| 961 | Standardized electrolyte supplementation and fluid management improves survival during amphotericin therapy for cryptococcal meningitis in resource-limited settings. | Bahr NC; Rolfes MA; Musubire A; Nabeta H; Williams DA; Rhein J; Kambugu A; Meya DB; Boulware DR | 2014 | Excluded | Irrelevant, did not discuss the subject matter |
| 962 | Characterization of Cryptococcus neoformans isolated from urban environmental sources in GoiÃ¢nia, GoiÃ¡s State, Brazil. | Kobayashi CC; Souza LK; Fernandes Ode F; Brito SC; Silva AC; Sousa ED; Silva Mdo R | 2005 | Excluded | Irrelevant, did not discuss the subject matter |
| 963 | Aetiology, clinical presentation, and outcome of meningitis in patients coinfected with human immunodeficiency virus and tuberculosis. | Bhagwan S; Naidoo K | 2011 | Excluded | Irrelevant, did not discuss the subject matter |
| 964 | Cerebral Cryptococcosis Associated with CD4+ T-lymphocytopenia in Non-HIV Patients after SARS-CoV-2 Infection: Case Series in a Specialized Institute in Lima, Peru. | Huamani-CÃ³rdova JM; Hueda-Zavaleta M; Vargas-Bellina V; Simbron-Ribbeck L; Chong-Chinchay KDR; GÃ³mez de la Torre JC; BenÃ­tes-Zapata VA | 2023 | Excluded | Irrelevant, did not discuss the subject matter |
| 965 | Intracranial pressure management in patients with human immunodeficiency virus-associated cryptococcal meningitis in a resource-constrained setting. | Mkoko P; Du Preez J; Naidoo S | 2020 | Excluded | Irrelevant, did not discuss the subject matter |
| 966 | Mortality in an antiretroviral therapy programme in Jinja, south-east Uganda: a prospective cohort study. | Amuron B; Levin J; Birunghi J; Namara G; Coutinho A; Grosskurth H; Jaffar S | 2011 | Excluded | Irrelevant, did not discuss the subject matter |
| 967 | Clinical spectrum and outcome of hospitalized patients with invasive fungal infections: a prospective study from a medical ward/intensive care unit of a teaching hospital in North India. | Sindhu D; Jorwal P; Gupta N; Xess I; Singh G; Soneja M; Nischal N; Sethi P; Ray A; Biswas A; Wig N | 2019 | Excluded | Irrelevant, did not discuss the subject matter |
| 968 | [Frequency of invasive fungal infections in a Mexican High-Specialty Hospital. Experience of 21 years]. | MÃ©ndez-Tovar LJ; MejÃ­a-Mercado JA; Manzano-Gayosso P; HernÃ¡ndez-HernÃ¡ndez F; LÃ³pez-MartÃ­nez R; Silva-GonzÃ¡lez I | 2016 | Excluded | Irrelevant, did not discuss the subject matter |
| 969 | Presentation, etiology, and outcome of brain infections in an Indonesian hospital: A cohort study. | Imran D; Estiasari R; Maharani K; Sucipto; Lestari DC; Yunus RE; Yunihastuti E; Karyadi TH; Oei D; Timan IS; Wulandari D; Wahyuningsih R; Adawiyah R; Kurniawan A; Mulyadi R; Karuniawati A; Jaya UA; Safari D; van Laarhoven A; Alisjahbana B; Dian S; Chaidir L; Ganiem AR; Lastri DN; Aye Myint KS; van Crevel R | 2018 | Excluded | Irrelevant, did not discuss the subject matter |
| 970 | Recent trends in the spectrum of opportunistic infections in human immunodeficiency virus infected individuals on antiretroviral therapy in South India. | Shahapur PR; Bidri RC | 2014 | Excluded | Irrelevant, did not discuss the subject matter |
| 971 | Therapeutic Lumbar Punctures in Human Immunodeficiency Virus-Associated Cryptococcal Meningitis: Should Opening Pressure Direct Management? | Kagimu E; Engen N; Ssebambulidde K; Kasibante J; Kiiza TK; Mpoza E; Tugume L; Nuwagira E; Nsangi L; Williams DA; Hullsiek KH; Boulware DR; Meya DB; Rhein J; Abassi M; Musubire AK | 2022 | Excluded | Irrelevant, did not discuss the subject matter |
| 972 | Immune restoration disease and changes in CD4+ T-cell count in HIV- infected patients during highly active antiretroviral therapy at Zewditu memorial hospital, Addis Ababa, Ethiopia. | Huruy K; Kassu A; Mulu A; Wondie Y | 2010 | Excluded | Irrelevant, did not discuss the subject matter |
| 973 | Cryptococcosis with Tuberculosis: Overlooked Coinfections. | Suresh CS; Ninan MM; Zachariah A; Michael JS | 2021 | Excluded | Irrelevant, did not discuss the subject matter |
| 974 | Standardized Urine-Based Tuberculosis (TB) Screening With TB-Lipoarabinomannan and Xpert MTB/RIF Ultra in Ugandan Adults With Advanced Human Immunodeficiency Virus Disease and Suspected Meningitis. | Cresswell FV; Ellis J; Kagimu E; Bangdiwala AS; Okirwoth M; Mugumya G; Rutakingirwa M; Kasibante J; Quinn CM; Ssebambulidde K; Rhein J; Nuwagira E; Tugume L; Martyn E; Skipper CP; Muzoora C; Grint D; Meya DB; Bahr NC; Elliott AM; Boulware DR | 2020 | Excluded | Irrelevant, did not discuss the subject matter |
| 975 | Management of cryptococcal meningitis in HIV-infected patients: Experience from western India. | Patel AK; Patel KK; Ranjan R; Shah S; Patel JK | 2010 | Excluded | Irrelevant, did not discuss the subject matter |
| 976 | Spontaneous cryptococcal peritonitis with fungemia in patients with decompensated cirrhosis: Report of two cases. | Bal CK; Bhatia V; Khillan V; Rathor N; Saini D; Daman R; Sarin SK | 2014 | Excluded | Irrelevant, did not discuss the subject matter |
| 977 | Profile of presentation of human immunodeficiency virus infection in north India, 2003-2007. | Kumar S; Wanchu A; Abeygunasekera N; Sharma A; Singh S; Varma S | 2012 | Excluded | Irrelevant, did not discuss the subject matter |
| 978 | Atypical Presentation of Pediatric Systemic Lupus Erythematosus Complicated by Cryptococcal Meningitis. | Hashem HE; Ibrahim ZH | 2021 | Excluded | Irrelevant, did not discuss the subject matter |
| 979 | HIV-Associated Central Nervous System Disease in Patients Admitted at the Douala General Hospital between 2004 and 2009: A Retrospective Study. | Luma HN; Tchaleu BC; Temfack E; Doualla MS; Ndenga DP; Mapoure YN; Njamnshi AK; Djientcheu VD | 2013 | Excluded | Irrelevant, did not discuss the subject matter |
| 980 | Molecular Epidemiology and Antifungal Resistance of Cryptococcus neoformans From Human Immunodeficiency Virus-Negative and Human Immunodeficiency Virus-Positive Patients in Eastern China. | Zhou Z; Zhu C; Ip M; Liu M; Zhu Z; Liu R; Li X; Zeng L; Wu W | 2022 | Excluded | Irrelevant, did not discuss the subject matter |
| 981 | [Dermatomycoses due to pets and farm animals : neglected infections?]. | Nenoff P; Handrick W; KrÃ¼ger C; Vissiennon T; Wichmann K; GrÃ¤ser Y; Tchernev G | 2012 | Excluded | Irrelevant, did not discuss the subject matter |
| 982 | Clinical presentation and aetiologies of acute or complicated headache among HIV-seropositive patients in a Ugandan clinic. | Katwere M; Kambugu A; Piloya T; Wong M; Hendel-Paterson B; Sande MA; Ronald A; Katabira E; Were EM; Menten J; Colebunders R | 2009 | Excluded | Irrelevant, did not discuss the subject matter |
| 983 | Hospital-based Retrospective Study of Cryptococcal Meningitis in a Large Cohort from India. | Naik KR; Saroja AO; Doshi DK | 2017 | Excluded | Irrelevant, did not discuss the subject matter |
| 984 | Clinical and Radiological Features of Patients With Pulmonary Cryptococcosis in a Hospital of North China. | Liang L; Cong P; Wang Y; Liang Z | 2020 | Excluded | Irrelevant, did not discuss the subject matter |
| 985 | Clinical Profile of 24 AIDS Patients with Cryptococcal Meningitis in the HAART Era: A Report from an Infectious Diseases Tertiary Hospital in Western Romania. | Marincu I; Citu C; Vidican I; Bratosin F; Mares M; Suciu O; Frent S; Bota AV; Timircan M; Bratu ML; Grigoras ML | 2021 | Excluded | Irrelevant, did not discuss the subject matter |
| 986 | Neuropathology of AIDS: An Autopsy Review of 284 Cases from Brazil Comparing the Findings Pre- and Post-HAART (Highly Active Antiretroviral Therapy) and Pre- and Postmortem Correlation. | Silva AC; Rodrigues BS; Micheletti AM; Tostes S Jr; Meneses AC; Silva-Vergara ML; Adad SJ | 2012 | Excluded | Irrelevant, did not discuss the subject matter |
| 987 | Spectrum of fungal infection in a neurology tertiary care center in India. | Agarwal R; Kalita J; Marak RS; Misra UK | 2012 | Excluded | Irrelevant, did not discuss the subject matter |
| 988 | Antifungal susceptibility and molecular characteristics of Cryptococcus spp. based on whole-genome sequencing in Zhejiang Province, China. | Zhang J; Wang Z; Chen Y; Zhou Z; Yang Q; Fu Y; Zhao F; Li X; Chen Q; Fang L; Jiang Y; Yu Y | 2022 | Excluded | Irrelevant, did not discuss the subject matter |
| 989 | A randomized open label trial of tamoxifen combined with amphotericin B and fluconazole for cryptococcal meningitis. | Ngan NTT; Mai NTH; Tung NLN; Lan NPH; Tai LTH; Phu NH; Chau NVV; Binh TQ; Hung LQ; Beardsley J; White N; Lalloo D; Krysan D; Hope W; Geskus R; Wolbers M; Nhat LTH; Thwaites G; Kestelyn E; Day J | 2019 | Excluded | Irrelevant, did not discuss the subject matter |
| 990 | Utility of Cerebrospinal Fluid Protein Levels as a Potential Predictive Biomarker of Disease Severity in HIV-Associated Cryptococcal Meningitis. | Kasibante J; Irfanullah E; Wele A; Okafor E; Ssebambulidde K; Okurut S; Kagimu E; Gakuru J; Rutakingirwa MK; Mugabi T; Nuwagira E; Jjunju S; Mpoza E; Tugume L; Nsangi L; Musibire AK; Muzoora C; Rhein J; Meya DB; Boulware DR; Abassi M | 2023 | Excluded | Irrelevant, did not discuss the subject matter |
| 991 | Neurological manifestations of HIV infection in Nigerians. | Imam I | 2007 | Excluded | Irrelevant, did not discuss the subject matter |
| 992 | Cryptococcal Meningitis in an HIV-Negative Pulmonary Tuberculosis Patient: A Case Report. | Naseem MA; Khan MA; Ali W; Danial Malik M; Aslam W | 2023 | Excluded | Irrelevant, did not discuss the subject matter |
| 993 | The conundrum of clinical trials and standard of care in sub-Saharan Africa - the research nurse perspective. | Frances Ndyetukira J; Kwizera R; Kugonza F; Ahimbisibwe C; Namujju C; Sadiq A; Namudde A; Williams D; Abassi M; Rhein J; Boulware DR; Meya DB | 2019 | Excluded | Irrelevant, did not discuss the subject matter |
| 994 | Safety and tolerability of intrathecal liposomal amphotericin B (AmBisome) for cryptococcal meningitis: a retrospective study in HIV-infected patients. | Alvarez-Uria G; Midde M; Battula J; Pujari HNB | 2018 | Excluded | Irrelevant, did not discuss the subject matter |
| 995 | Cutaneous cryptococcosis in an adult male of sub-saharan origin. | Thakur R; Kalsi AS; Kushwaha P | 2023 | Excluded | Irrelevant, did not discuss the subject matter |
| 996 | Interventions to reduce deaths in people living with HIV admitted to hospital in low- and middle-income countries: A systematic review. | Burke RM; Twabi HH; Johnston C; Nliwasa M; Gupta-Wright A; Fielding K; Ford N; MacPherson P; Corbett EL | 2023 | Excluded | Irrelevant, did not discuss the subject matter |
| 997 | Gelatinous marrow transformation: a series of 11 cases from a tertiary care centre in South India. | Das S; Mishra P; Kar R; Basu D | 2014 | Excluded | Irrelevant, did not discuss the subject matter |
| 998 | Fatal Cryptococcal Meningitis in a Patient With Chronic Lymphocytic Leukemia Treated With Ibrutinib. | Oumayma H; Mahtat EM; Moussa Bouh H; Elmaaroufi H; Doghmi K | 2023 | Excluded | Irrelevant, did not discuss the subject matter |
| 999 | Short-Course Induction Treatment with Intrathecal Amphotericin B Lipid Emulsion for HIV Infected Patients with Cryptococcal Meningitis. | Alvarez-Uria G; Midde M; Pakam R; Yalla PS; Naik PK; Reddy R | 2015 | Excluded | Irrelevant, did not discuss the subject matter |
| 1000 | Clinical analysis of non-AIDS patients pathologically diagnosed with pulmonary cryptococcosis. | Liu K; Ding H; Xu B; You R; Xing Z; Chen J; Lin Q; Qu J | 2016 | Excluded | Irrelevant, did not discuss the subject matter |
| 1001 | Cryptococcal meningoencephalitis: time for action. | Stott KE; Loyse A; Jarvis JN; Alufandika M; Harrison TS; Mwandumba HC; Day JN; Lalloo DG; Bicanic T; Perfect JR; Hope W | 2021 | Excluded | Irrelevant, did not discuss the subject matter |
| 1002 | Mortality among HIV-Infected Patients in Resource Limited Settings: A Case Controlled Analysis of Inpatients at a Community Care Center. | Rajagopalan N; Suchitra JB; Shet A; Khan ZK; Martin-Garcia J; Nonnemacher MR; Jacobson JM; Wigdahl B | 2009 | Excluded | Irrelevant, did not discuss the subject matter |
| 1003 | ANTIFUNGAL SUSCEPTIBILITY TESTING AND GENOTYPING CHARACTERIZATION OF Cryptococcus neoformans AND gattii ISOLATES FROM HIV-INFECTED PATIENTS OF RIBEIRÃƒO PRETO, SÃƒO PAULO, BRAZIL. | Figueiredo TP; Lucas RC; Cazzaniga RA; FranÃ§a CN; Segato F; Taglialegna R; Maffei CM | 2016 | Excluded | Irrelevant, did not discuss the subject matter |
| 1004 | The Effect of Early vs. Deferred Antiretroviral Therapy Initiation in HIV-Infected Patients With Cryptococcal Meningitis: A Multicenter Prospective Randomized Controlled Analysis in China. | Zhao T; Xu XL; Lu YQ; Liu M; Yuan J; Nie JM; Yu JH; Liu SQ; Yang TT; Zhou GQ; Liu J; Qin YM; Chen H; Harypursat V; Chen YK | 2021 | Excluded | Irrelevant, did not discuss the subject matter |
| 1005 | Neuromeningeal cryptococcosis in sub-Saharan Africa: Killer disease with sparse data. | Assogba K; Belo M; Wateba MI; Gnonlonfoun DD; Ossou-Nguiet PM; Tsanga BB; Ndiaye M; Grunitzky EK | 2015 | Excluded | Irrelevant, did not discuss the subject matter |
| 1006 | Clinical Characteristics of HIV-Associated Optic Neuritis. | Jindahra P; Phuphuakrat A; Tangjaisanong T; Siriyotha S; Padungkiatsagul T; Vanikieti K; Preechawat P; Poonyathalang A; Sungkanuparph S; Pulkes T; Tunlayadechanont S | 2020 | Excluded | Irrelevant, did not discuss the subject matter |
| 1007 | Cryptococcal Meningitis: Diagnosis and Management Update. | Abassi M; Boulware DR; Rhein J | 2015 | Excluded | Irrelevant, did not discuss the subject matter |
| 1008 | Tracing Genetic Exchange and Biogeography of Cryptococcus neoformans var. grubii at the Global Population Level. | Rhodes J; Desjardins CA; Sykes SM; Beale MA; Vanhove M; Sakthikumar S; Chen Y; Gujja S; Saif S; Chowdhary A; Lawson DJ; Ponzio V; Colombo AL; Meyer W; Engelthaler DM; Hagen F; Illnait-Zaragozi MT; Alanio A; Vreulink JM; Heitman J; Perfect JR; Litvintseva AP; Bicanic T; Harrison TS; Fisher MC; Cuomo CA | 2017 | Excluded | Irrelevant, did not discuss the subject matter |
| 1009 | [Causes and characteristics of patient death under ARV at the Dermatology-Venereology Department of the Donka National Hospital, GUINEA]. | Camara A; Diaby B; Sako FB; Camara AD; BaldÃ© H; Keita M; Doumbouya A; Kaba H; Tounkara TM; Soummah MM; DianÃ© BF; CissÃ© M | 2012 | Excluded | Irrelevant, did not discuss the subject matter |
| 1010 | Clinical analysis of pulmonary cryptococcosis in non-HIV patients in south China. | Xie X; Xu B; Yu C; Chen M; Yao D; Xu X; Cai X; Ding C; Wang L; Huang X | 2015 | Excluded | Irrelevant, did not discuss the subject matter |
| 1011 | Cryptococcal meningitis: An under-reported disease from the hills of Uttarakhand: A hospital-based cross-sectional study. | Mohanty A; Bhatia M; Kabi A; Chatterjee K; Kaistha N; Omar BJ; Gupta PK; Gupta P | 2019 | Excluded | Irrelevant, did not discuss the subject matter |
| 1012 | Prevalence of cryptococcal meningitis among people living with human immunodeficiency virus/acquired immunodeficiency syndrome in a Tertiary Care Hospital, Southern Odisha, India. | Dash M; Padhi S; Sahu R; Turuk J; Pattanaik S; Misra P | 2014 | Excluded | Irrelevant, did not discuss the subject matter |
| 1013 | In vitro characterization and molecular epidemiology of Cryptococcus spp. isolates from non-HIV patients in Guangdong, China. | Wang P; Li Y; Gao L; Tang X; Zheng D; Wu K; Wang L; Guo P; Ye F | 2023 | Excluded | Irrelevant, did not discuss the subject matter |
| 1014 | Comparing the profile of respiratory fungal pathogens amongst immunocompetent and immunocompromised hosts, their susceptibility pattern and correlation of various opportunistic respiratory fungal infections and their progression in relation to the CD4+T-c | Roohani AH; Fatima N; Shameem M; Khan HM; Khan PA; Akhtar A | 2018 | Excluded | Irrelevant, did not discuss the subject matter |
| 1015 | Cross-reactivity of a Histoplasma capsulatum antigen enzyme immunoassay in urine specimens from persons with emergomycosis in South Africa. | Maphanga TG; Naicker SD; GÃ³mez BL; Mhlanga M; Mpembe RS; Schwartz IS; Bamford C; Nel J; Govender NP | 2021 | Excluded | Irrelevant, did not discuss the subject matter |
| 1016 | Cryptococcal Meningitis in HIV-Negative Patients: A 12-Year Single-Center Experience in China. | Huang Y; Jin X; Wu F; Pan T; Wang X; Chen D; Chen Y | 2023 | Excluded | Irrelevant, did not discuss the subject matter |
| 1017 | Is it possible to differentiate tuberculous and cryptococcal meningitis in HIV-infected patients using only clinical and basic cerebrospinal fluid characteristics? | Vidal JE; Peixoto de Miranda EJ; Gerhardt J; Croda M; Boulware DR | 2017 | Excluded | Irrelevant, did not discuss the subject matter |
| 1018 | Computerised tomography findings in HIV-associated cryptococcal meningoencephalitis at a tertiary hospital in Pretoria. | Khan N; Hiesgen J | 2017 | Excluded | Irrelevant, did not discuss the subject matter |
| 1019 | Case Report: Streptococcus Suis Meningitis Diagnosed in a HIV-Infected Patient With Cryptococcal Meningitis Using Next-Generation Sequencing. | Xie Y; Ruan B; Lang G; Zhu B | 2021 | Excluded | Irrelevant, did not discuss the subject matter |
| 1020 | A Study of the Etiology, Clinical Profile, and Diagnosis of Various Types of Central Nervous System Infections in a Tertiary Care Center. | Patel S; Jhala P; Sharma H | 2024 | Excluded | Irrelevant, did not discuss the subject matter |
| 1021 | Spectrum of AIDS Defining Opportunistic Infections in a Series of 77 Hospitalised HIV-infected Omani Patients. | Balkhair AA; Al-Muharrmi ZK; Ganguly S; Al-Jabri AA | 2012 | Excluded | Irrelevant, did not discuss the subject matter |
| 1022 | HIV-Associated Cryptococcal Meningitis: Bridging the Gap Between Developed and Resource-Limited Settings. | Tenforde MW; Wake R; Leeme T; Jarvis JN | 2016 | Excluded | Irrelevant, did not discuss the subject matter |
| 1023 | Therapy for HIV-associated cryptococcal meningitis: a case report demonstrating a new treatment approach emphasizing updated treatment guidelines. | Hachey DM; Wood BR; Buitrago M; Burde A | 2023 | Excluded | Irrelevant, did not discuss the subject matter |
| 1024 | CSF ADA Determination in Early Diagnosis of Tuberculous Meningitis in HIV-Infected Patients. | Ghosh GC; Sharma B; Gupta BB | 2016 | Excluded | Irrelevant, did not discuss the subject matter |
| 1025 | Antimicrobial therapy for the treatment of opportunistic infections in HIV/AIDS patients: a critical appraisal. | Seddon J; Bhagani S | 2011 | Excluded | Irrelevant, did not discuss the subject matter |
| 1026 | Reimagining the future of African brain health: Perspectives for basic research on the pathogenesis of cryptococcal meningitis. | Dangarembizi R | 2021 | Excluded | Irrelevant, did not discuss the subject matter |
| 1027 | Case 259. | Tian D; Zhu X; Xue R; Zhao P; Yao Y | 2018 | Excluded | Irrelevant, did not discuss the subject matter |
| 1028 | Case 259: Primary Central Nervous System Lymphomatoid Granulomatosis Mimicking Chronic Lymphocytic Inflammation with Pontine Perivascular Enhancement Responsive to Steroids (CLIPPERS). | Tian D; Zhu X; Xue R; Zhao P; Yao Y | 2018 | Excluded | Irrelevant, did not discuss the subject matter |
| 1029 | Evaluation of proline-rich antimicrobial peptides as potential lead structures for novel antimycotics against Cryptococcus neoformans. | Brakel A; Grochow T; Fritsche S; Knappe D; Krizsan A; Fietz SA; Alber G; Hoffmann R; MÃ¼ller U | 2023 | Excluded | Irrelevant, did not discuss the subject matter |
| 1030 | Does introduction of thresholds in decision aids benefit the patient?: Comparison between findings-based and threshold-based diagnostic decision aids. | Bisig B; Moreira J; Combes M; Asiimwe A; Bisoffi Z; Haegeman F; Bottieau E; Van den Ende J | 2012 | Excluded | Irrelevant, did not discuss the subject matter |
| 1031 | Mortality and associated factors among people living with HIV admitted at a tertiary-care hospital in Uganda: a cross-sectional study | Owachi, D.; Akatukunda, P.; Nanyanzi, D.S.; Katwesigye, R.; Wanyina, S.; Muddu, M.; Kawuma, S.; Kalema, N.; Kabugo, C.; Semitala, F.C. | 2024 | Excluded | Irrelevant, did not discuss the subject matter |
| 1032 | Comparable clinical outcomes with same-day versus rapid initiation of antiretroviral therapy in Taiwan | Huang, Y.-C.; Yang, C.-J.; Sun, H.-Y.; Lee, C.-H.; Lu, P.-L.; Tang, H.-J.; Liu, C.-E.; Lee, Y.-T.; Tsai, C.-S.; Lee, N.-Y.; Liou, B.-H.; Hung, T.-C.; Lee, M.-H.; Huang, M.-H.; Wang, N.-C.; Lin, C.-Y.; Lee, Y.-C.; Cheng, S.-H.; Hung, C.-C. | 2024 | Excluded | Irrelevant, did not discuss the subject matter |
| 1033 | Predictive model for diagnostic yield of bone marrow examination in patients with HIV infection having fever of unknown origin | Noiperm, P.; Saelue, P. | 2024 | Excluded | Irrelevant, did not discuss the subject matter |
| 1034 | Clinical practice guideline for the diagnosis and treatment of cryptococcal disease among adults living with HIV | Alberto CorteÌs, J.; Valderrama-Rios, M.C.; Lizarazo, J.; MillÃ¡n-OnÌƒate, J.; Diaz-Brochero, C.; GÃ³mez Quintero, C.H.; Nocua-BÃ¡ez, L.C.; Buitrago, E.M.; JoseÌ LÃ³pez, M.; Remolina-Granados, S.A.; Castaneda, E.; EscandÃ³n, P.; GÃ³mez, J.C.; HernÃ¡ndez-Rojas, E.S.; VarÃ³n-Vega, F.; Ãlvarez-Moreno, C.A. | 2024 | Excluded | Irrelevant, did not discuss the subject matter |
| 1035 | Neurological manifestations of syphilis-HIV coinfection in South Africa | Shorer, E.F.; Zauchenberger, C.Z.; Govender, S.; Shorer, G.E.; Geragotellis, A.A.; Centner, C.M.; Marais, S. | 2023 | Excluded | Irrelevant, did not discuss the subject matter |
| 1036 | Lesion size as a prognostic factor in the antifungal treatment of pulmonary cryptococcosis: a retrospective study with chest CT pictorial review of 2-year follow up | Yan, Y.; Wu, Y.; Wang, Q.; Zhu, X.; Li, H.; Jiang, H. | 2023 | Excluded | Irrelevant, did not discuss the subject matter |
| 1037 | Neurological manifestations of HIV infection and itâ€™s correlation with CD 4 counts and viral load | Thakur, S.; Gupta, A.; Kanjolia, J.; Dubay, A.; Rai, S.; Patidar, M. | 2023 | Excluded | Irrelevant, did not discuss the subject matter |
| 1038 | Brain infections among HIV-infected adults in Yogyakarta, Indonesia | Aifa, B.L.; Satiti, S.; Setyopranoto, I.; Putri, V.P. | 2023 | Excluded | Irrelevant, did not discuss the subject matter |
| 1039 | Neurological manifestations during an HIV infection | El Azhari, M.; Saaf, S.; El Yakoubi, Z.; Mimouni, Y.; Hakimi, M.; Lhassani, S.; Hazim, A.; Aasfara, J.; Ouhabi, H. | 2023 | Excluded | Irrelevant, did not discuss the subject matter |
| 1040 | ADVANCED HIV DISEASE PACKAGE IMPLEMENTATION DURING COMMUNITY-BASED ACTIVE TB-CASE FINDING | Gils, T.; Kamele, M.; Madonsela, T.; Ayakaka, I.; Van Heerden, A.; Vlieghe, E.; Decroo, T.; Reither, K.; Lynen, L.; Bosman, S. | 2023 | Excluded | Irrelevant, did not discuss the subject matter |
| 1041 | High direct costs caused by Cryptococcal meningitis treated with Amphotericin B deoxycholate in a public hospital in Lima, Peru. 2021-2022 | Vilcarromero, S.; Luque, A.; Luarte, N.; Mateo-Pacora, J.; Mendo, F. | 2023 | Excluded | Irrelevant, did not discuss the subject matter |
| 1042 | 1497. Cryptococcal Antigen Screening and Pre-emptive Fluconazole Therapy in HIV-infected Patients: A Multicenter, Retrospective Study in China | Lu, Y.; Chen, Y. | 2023 | Excluded | Irrelevant, did not discuss the subject matter |
| 1043 | The Magnitude, Distribution Patterns, and Risk Factors of AIDS-Related Illnesses in Adults on Combined Antiretroviral Therapy in Malawi | Salema, H.H. | 2023 | Excluded | Irrelevant, did not discuss the subject matter |
| 1044 | Prevalence, treatment, and factors associated with cryptococcal meningitis post introduction of integrase inhibitors antiretroviral based regimens among people living with HIV in Tanzania | Minja, M.; Mbilinyi, T.; Mkinga, B.; Philipo, E.G.; Owenya, J.; Kilonzi, M. | 2023 | Excluded | Irrelevant, did not discuss the subject matter |
| 1045 | HIV and fungal priority pathogens | Sati, H.; Alastruey-Izquierdo, A.; Perfect, J.; Govender, N.P.; Harrison, T.S.; Chiller, T.; Sorrell, T.C.; Bongomin, F.; Oladele, R.; Chakrabarti, A.; Wahyuningsih, R.; Colombo, A.L.; Rodriguez-Tudela, J.L.; Beyrer, C.; Ford, N. | 2023 | Excluded | Irrelevant, did not discuss the subject matter |
| 1046 | Characteristics and risk factors for readmission in HIV-infected patients with Talaromyces marneffei infection | Shi, M.; Qin, Y.; Chen, S.; Wei, W.; Meng, S.; Chen, X.; Li, J.; Li, Y.; Chen, R.; Su, J.; Yuan, Z.; Wang, G.; Ye, L.; Liang, H.; Xie, Z.; Jiangid, J. | 2023 | Excluded | Irrelevant, did not discuss the subject matter |
| 1047 | WORKUP AND DISCOVERY OF DISSEMINATED CRYPTOCOCCOSIS IN AN HIV-NEGATIVE INDIVIDUAL | Mckinney, D.; Kosnik, N.R.; Yassine, N.; Hasnain, S.Y.E.D.; Elsayed, O.M.A.R.; Poluru, K. | 2023 | Excluded | Irrelevant, did not discuss the subject matter |
| 1048 | PULMONARY CRYPTOCOCCOSIS DUE TO CRYPTOCOCCUS NEOFORMANS FOUND INCIDENTALLY IN IMMUNOCOMPETENT PATIENT INITIALLY MISDIAGNOSED WITH MALIGNANCY | Gonzalez, V.; Martins, A. | 2023 | Excluded | Irrelevant, did not discuss the subject matter |
| 1049 | PULMONARY EMBOLISM IN A PATIENT WITH TUBERCULOSIS | Carter, D.; Nguyen, T.; Faruqi, M.A. | 2023 | Excluded | Irrelevant, did not discuss the subject matter |
| 1050 | Chinese expert consensus on the diagnosis of non-human immunodeficiency virus related cryptococcal meningitis | | 2023 | Excluded | Irrelevant, did not discuss the subject matter |
| 1051 | Prevalence of serum cryptococcal antigen among HIV infected patients attending a university of Benin Teaching Hospital, Edo state, Nigeria | Constance, O.C.; Emmanuel, M.; Blessing Ubulu, M. | 2023 | Excluded | Irrelevant, did not discuss the subject matter |
| 1052 | Innovative quantitative PCR assays for the assessment of HIV-associated cryptococcal meningoencephalitis in Sub-Saharan Africa | Mbangiwa, T.; Sturny-LeclÃ¨re, A.; Lechiile, K.; Kajanga, C.; Boyer-Chammard, T.; Hoving, J.C.; Leeme, T.; Moyo, M.; Youssouf, N.; Lawrence, D.S.; Mwandumba, H.; Mosepele, M.; Harrison, T.S.; Jarvis, J.N.; Lortholary, O.; Alanio, A.; Goodall, J.; Mawoko, N.; Milburn, J.; Mmipi, R.; Muthoga, C.; Ponatshego, P.; Rulaganyang, I.; Seatla, K.; Tlhako, N.; Tsholo, K.; April, S.; Bekiswa, A.; Boloko, L.; Bookholane, H.; Crede, T.; Davids, L.; Goliath, R.; Hlungulu, S.; Hoffman, R.; Kyepa, H.; Masina, N.; Maughan, D.; Mnguni, T.; Moosa, S.; Morar, T.; Mpalali, M.; Naude, J.; Oliphant, I.; Sayed, S.; Sebesho, L.; Shey, M.; Swanepoel, L.; Chasweka, M.; Chimangâ€™anga, W.; Chimphambano, T.; Dziwani, E.; Gondwe, E.; Kadzilimbile, A.; Kateta, S.; Kossam, E.; Kukacha, C.; Lipenga, B.; Ndaferankhande, J.; Ndalama, M.; Shah, R.; Singini, A.; Stott, K.; Zambasa, A.; Banda, T.; Chikaonda, T.; Chitulo, G.; Chiwoko, L.; Chome, N.; Gwin, M.; Kachitosi, T.; Kamanga, B.; Kazembe, M.; Kumwenda, E.; Kumwenda, M.; Maya, C.; Mhango, W.; Mphande, C.; Msumba, L.; Munthali, T.; Ngoma, D.; Nicholas, S.; Simwinga, L.; Stambuli, A.; Tegha, G.; Zambezi, J.; Ahimbisibwe, C.; Akampurira, A.; Alice, A.; Cresswell, F.; Gakuru, J.; Kiiza, D.; Kisembo, J.; Kwizera, R.; Kugonza, F.; Laker, E.; Luggya, T.; Lule, A.; Musubire, A.; Muyise, R.; Namujju, O.; Ndyetukira, J.; Nsangi, L.; Okirwoth, M.; Sadiq, A.; Tadeo, K.; Tukundane, A.; Williams, D.; Atwine, L.; Buzaare, P.; Collins, M.; Emily, N.; Inyakuwa, C.; Kariisa, S.; Mwesigye, J.; Niwamanya, S.; Rodgers, A.; Rukundo, J.; Rwomushana, I.; Ssemusu, M.; Stead, G.; Boyd, K.; Gondo, S.; Kufa, P.; Makaha, E.; Moyo, C.; Mtisi, T.; Mudzingwa, S.; Mwarumba, T.; Zinyandu, T.; Dromer, F.; Griffin, P.; Hafeez, S. | 2023 | Excluded | Irrelevant, did not discuss the subject matter |
| 1053 | Impact of prior cryptococcal antigen screening on in-hospital mortality in cryptococcal meningitis or fungaemia among HIV-seropositive individuals in South Africa: a cross-sectional observational study | Paccoud, O.; Shuping, L.; Mashau, R.; Greene, G.; Quan, V.; Meiring, S.; Govender, N.P.; Abrahams, S.; Ahmed, K.; Avenant, T.; Bamford, C.; Bhola, P.; Bishop, K.; Black, J.; Blumberg, L.; Bosman, N.; Botha, M.; Brink, A.; Budavari, S.; Burra, A.; Chibabhai, V.; Chomba, R.; Cohen, C.; Coovadia, Y.; Crowther-Gibson, P.; Dawood, H.; de Gouveia, L.; Dlamini, N.; Dlamini, S.; Dreyer, A.; du Plessis, N.; du Plessis, E.; du Plessis, M.; Erasmus, L.; Feldman, C.; Govender, N.; Govind, C.; Groome, M.; Haffejee, S.; Hamese, K.; Haumann, C.; Hoho, N.; Hoosen, A.; Hoosien, E.; Howell, V.; Hoyland, G.; Ismail, F.; Ismail, H.; Ismail, N.; Ive, P.; Jooste, P.; Karstaedt, A.; Khantsi, I.; Kleinhans, V.; Kleynhans, J.; Kolojane, M.; Kufa-Chakezha, T.; Lebaka, T.; Lebudi, J.; Legare, N.; Lekalakala, R.; Lindeque, K.; Lowman, W.; Madhi, S.; Magobo, R.; Mahabeer, P.; Maharaj, A.; Makgoba, M.; Maloba, M.; Maluleka, C.; Manaka, M.; Mangena, P.; Maningi, N.; Marcus, L.; Marshall, T.; Mathebula, R.; Mathunjwa, A.; Mbelle, N.; Mbuthu, B.; McCarthy, K.; Mekgoe, O.; Menezes, C.; Miller, C.; Mlisana, K.; Moncho, M.; Moore, D.; Moremi, M.; Morris, L.; Moshe, M.; Mothibi, L.; Moultrie, H.; Mpembe, R.; Mutevedzi, P.; Mwansa-Kambafwile, J.; Naby, F.; Naicker, P.; Naidoo, R.; Nana, T.; Nchabeleng, M.; Ndlovu, P.; Nel, J.; Ngomane, M.; Ngubane, W.; Nicol, M.; Njikho, S.; Ntlemo, G.; Ntuli, S.; Page, N.; Paulse, N.; Pearce, V.; Perovic, O.; Pillay, K.; Pombo, D.; Poswa, X.; Prentice, E.; Puren, A.; Ramjathan, P.; Ramkillawan, Y.; Reddy, K.; Reubenson, G.; Richards, L.; Said, M.; Samodien, N.; Samuel, C.; Seetharam, S.; Sekwadi, P.; Selekisho, M.; Senekal, M.; Sibiya, N.; Sirkar, S.; Smit, J.; Smith, A.; Smith, M.; Sookan, L.; Sriruttan, C.; Stacey, S.; Swe Han, K.S.; Thomas, T.; Thomas, J.; Tsisti, M.; van Schalkwyk, E.; Variava, E.; Vazi, P.; Verwey, C.; von Gottberg, A.; Wadula, J.; Walaza, S.; Wende, L.; Whitelaw, A.; Wilson, D.; Zietsman, I. | 2023 | Excluded | Irrelevant, did not discuss the subject matter |
| 1054 | Immune responses to human fungal pathogens andÂ therapeutic prospects | Lionakis, M.S.; Drummond, R.A.; Hohl, T.M. | 2023 | Excluded | Irrelevant, did not discuss the subject matter |
| 1055 | Fungal infections in Algeria | Aissat, F.Z.; Denning, D.W. | 2023 | Excluded | Irrelevant, did not discuss the subject matter |
| 1056 | Characterisation and variables associated with mortality in a population with HIV and central nervous system opportunistic infections in a Colombian public hospital in BogotÃ¡ | Torres-UrazÃ¡n, D.; CajicÃ¡-MartÃ­nez, G.; Valencia-Enciso, N.; Espinosa-Jovel, C. | 2023 | Excluded | Irrelevant, did not discuss the subject matter |
| 1057 | Case Report: Role of Point-of-Care Ultrasound in the Diagnosis of Bacillary Angiomatosis | Stroffolini, G.; Rambiki, K.; Wallrauch, C.; Tomoka, T.; Brunetti, E.; Heller, T. | 2023 | Excluded | Irrelevant, did not discuss the subject matter |
| 1058 | Prevalence and Factors Associated With Cryptoccocal Antigenemia Among Patients With Advanced Human Immunodeficiency Virus in Eastern Uganda: A Facility-Based Cross-sectional Study | Eric, E.; Olupot-Olupot, P.; Bwayo, D.; Meya, D.; Katuramu, R. | 2023 | Excluded | Irrelevant, did not discuss the subject matter |
| 1059 | MILIARY PNEUMONIA: HOW AN AUTOMATED CBC DIFFERENTIAL CAN GIVE CLUES | Pham, J.; Ngo, T. | 2023 | Excluded | Irrelevant, did not discuss the subject matter |
| 1060 | Persistently elevated intracranial pressure in cryptococcal meningitisâ€“ 76 therapeutic lumbar punctures | Jjunju, S.; Nuwagira, E.; Meya, D.B.; Muzoora, C. | 2023 | Excluded | Irrelevant, did not discuss the subject matter |
| 1061 | BURDEN OF SERIOUS HUMAN FUNGAL INFECTIONS IN MALAYSIA | Velayuthan, R.D. | 2023 | Excluded | Irrelevant, did not discuss the subject matter |
| 1062 | Clinical Profile of Cutaneous Adverse Drug Reactions in HIV Patients: A Cross-sectional Study | Gangavellaisamy, S.; Masilamani, M.; Thangamuthu, D.; Gopalan, K.; Kumarasamy, N. | 2023 | Excluded | Irrelevant, did not discuss the subject matter |
| 1063 | Tuberculosis and other causes of morbidity and mortality in hospitalized patients with HIV/AIDS | Acosta, M.B.; MÃ©ndez, D.M.; SolÃ³rzano, P.V. | 2023 | Excluded | Irrelevant, did not discuss the subject matter |
| 1064 | Chryseobacterium Indologenes ARDS in a Young, Previously Immunocompetent Patient of Chinese Descent | Hoerle, R.; Mussarat, S.; Qiao, X.; Johnston, M. | 2023 | Excluded | Irrelevant, did not discuss the subject matter |
| 1065 | PHARMACOKINETICS & SAFETY OF SUSTAINED-RELEASE FLUCYTOSINE PELLET FORMULATION | Krantz, E.; Simon, F.; Gillon, J.-Y.; Saayman, A.; Neven, A.; Robinson, S.; Eriksson, J.; Victor, S.; Ruffell, C.; Celebic, A.; Caplain, H.; Louis, M.; Goyal, V.; Ribeiro, I. | 2023 | Excluded | Irrelevant, did not discuss the subject matter |
| 1066 | COST-EFFECTIVENESS OF CRAG SCREENING FOR PLHIV WITH ADVANCED HIV DISEASE IN MALAWI | Feser, M.; Shroufi, A.; Maphosa, T.; Rangaraj, A.; Matiya, E.; Shrestha, P.; Ford, N.P.; Scott, J.; Talbot, V.; Tiam, A.; Phillips, A.; Freedberg, K.A.; Hyle, E.P. | 2023 | Excluded | Irrelevant, did not discuss the subject matter |
| 1067 | ADVANCED HIV DISEASE CARE PACKAGE IMPLEMENTATION IN LESOTHO AND SOUTH AFRICA | Gils, T.; Lynen, L.; Mashaete, K.; Muhairwe, J.; Ngubane, T.; Joseph, P.; Reither, K.; Bresser, M.; Van Heerden, A. | 2023 | Excluded | Irrelevant, did not discuss the subject matter |
| 1068 | CEREBROSPINAL FLUID PROTEIN CLINICAL IMPACT IN HIV-ASSOCIATED CRYPTOCOCCAL MENINGITIS | Kasibante, J.; Irfanullah, E.; Wele, A.; Ssebambulidde, K.; Mugabi, T.; Musubire, A.; Jjunju, S.; Nsangi, L.J.; Kagimu, E.; Rutakingirwa, M.K.; Abassi, M.; Muzoora, C.; Meya, D.B.; Boulware, D.R. | 2023 | Excluded | Irrelevant, did not discuss the subject matter |
| 1069 | Cryptococcal Meningitis and Spatial Distribution of Pigeons(Columba livia) in Colombia | Monsalve, G.P.; Forero, N. | 2023 | Excluded | Irrelevant, did not discuss the subject matter |
| 1070 | CRYPTOCOCCAL MENINGITIS IN THE IMMUNOCOMPETENT PATIENT: A CASE SERIES | Zimilover, A.; Hsieh, J.-C.; Kashfi, S.; Ghani, M.; Islam, I.; Sattler, S.; Chen, R.; Donaghy, H. | 2023 | Excluded | Irrelevant, did not discuss the subject matter |
| 1071 | TUBERCULOSIS IMMUNE RECONSTITUTION INFLAMMATORY SYNDROME: UNUSUAL TB UNMASKING FOLLOWING ART | Arinze, F.; Syed, S.; Demesilassie, T.; Check, R.; Amin, K. | 2023 | Excluded | Irrelevant, did not discuss the subject matter |
| 1072 | Virological Outcome in Newly Adult HIV Cases on Rapid ART initiation in Thailand, A Retrospective Study | Khusuwan, S.; Sirijanchune, P.; Pongprapass, S. | 2023 | Excluded | Irrelevant, did not discuss the subject matter |
| 1073 | Effect of HIV on mortality among hospitalised patients in South Africa | Lamprecht, D.J.; Martinson, N.; Variava, E. | 2023 | Excluded | Irrelevant, did not discuss the subject matter |
| 1074 | A retrospective clinical study of dolutegravir- versus efavirenz-based regimen in treatment-naÃ¯ve patients with advanced HIV infection in Nanjing, China | Zhong, M.; Li, M.; Qi, M.; Su, Y.; Yu, N.; Lv, R.; Ye, Z.; Zhang, X.; Xu, X.; Cheng, C.; Chen, C.; Wei, H. | 2023 | Excluded | Irrelevant, did not discuss the subject matter |
| 1075 | Prevelance of Central Nervous System Cryptococcosis in Human Immunodeficiency Virus Reactive Hospitalised Patients | Ashatha, P.; Sarada, D. | 2023 | Excluded | Irrelevant, did not discuss the subject matter |
| 1076 | Prevalence and determinants of opportunistic infections among HIV-infected adults receiving antiretroviral therapy in Ethiopia: A systematic review and meta-analysis | Woldegeorgis, B.Z.; Zekarias, Z.; Adem, B.G.; Obsa, M.S.; Kerbo, A.A. | 2023 | Excluded | Irrelevant, did not discuss the subject matter |
| 1077 | Incidence of opportunistic infections and its predictors among HIV/AIDS patients on antiretroviral therapy in Gondar University Comprehensive and Specialized Hospital, Ethiopia | Dagnaw, M.; Fekadu, H.; Gebre Egziabher, A.; Yesfue, T.; Indracanti, M.; Tebeje, A. | 2023 | Excluded | Irrelevant, did not discuss the subject matter |
| 1078 | Predictors of a high incidence of opportunistic infections among HIV-infected children receiving antiretroviral therapy at Amhara regional state comprehensive specialized hospitals, Ethiopia: A multicenter institution-based retrospective follow-up study | Mekonnen, G.B.; Birhane, B.M.; Engdaw, M.T.; Kindie, W.; Ayele, A.D.; Wondim, A. | 2023 | Excluded | Irrelevant, did not discuss the subject matter |
| 1079 | HIV/AIDS infection in critical care: epidemiological profile and risk factors for mortality in a Colombian cohort | Gaviria, S.P.; Zapata, N.; Villa, P.; Agudelo, C.A.; Molina, F.J.; GonzÃ¡lez, M.A.; Durango, L.V.; Zapata, S.; Galeano, C.; Cardona, J.; Rivera, S.; Hidron, A.I. | 2023 | Excluded | Irrelevant, did not discuss the subject matter |
| 1080 | Preventable deaths from respiratory diseases in children in low- and middle-income countries | Simba, J.M.; Irungu, A.; Otido, S.; Tumwa, D.; Mugane, S.; Musigula, R.; Andai, D.; Atieno, F.; Nyambura, M.; Mburugu, P. | 2023 | Excluded | Irrelevant, did not discuss the subject matter |
| 1081 | High concordance in plasma and CSF HIV-1 drug resistance mutations despite high cases of CSF viral escape in individuals with HIV-associated cryptococcal meningitis in Botswana | Kelentse, N.; Moyo, S.; Choga, W.T.; Lechiile, K.; Leeme, T.B.; Lawrence, D.S.; Kasvosve, I.; Musonda, R.; Mosepele, M.; Harrison, T.S.; Jarvis, J.N.; Gaseitsiwe, S. | 2023 | Excluded | Irrelevant, did not discuss the subject matter |
| 1082 | Cutaneous tuberculosis: An infrequent manifestation of a common pathogen in South Africa | van Heerden, J.K.; Broadhurst, A.G.B.; de Jager, R.S.; Plessis, W.D.; Ebrahim, N.; Mnguni, A.T.; Schietekat, D.; Meintjes, G. | 2023 | Excluded | Irrelevant, did not discuss the subject matter |
| 1083 | A Study of â€œEtiology and Prognosis of Medical Emergencies in HIV Patientsâ€ at a Tertiary Care Hospital in Southern India | Injeti, K.P.; Parise, G.; Sigamani, S.; Kukka, S.P.R. | 2023 | Excluded | Irrelevant, did not discuss the subject matter |
| 1084 | Contribution of point-of-care diagnostics in the identification of advanced HIV disease | Ditondo, P.; Luemba, A.; Ingwe Chuy, R.; Mucinya, G.; Ade, S. | 2023 | Excluded | Irrelevant, did not discuss the subject matter |
| 1085 | Prevalence of active Tuberculosis among adults with Human Immunodeficiency Virus who completed Isoniazid prophylaxis at Mitooma Health Center IV: A cross-sectional-descriptive study | Mugenyi, N.; Oduoye, M.O.; Suheb, M.K.; Akampwera, A.; Marsool, M.D.M. | 2023 | Excluded | Irrelevant, did not discuss the subject matter |
| 1086 | Spectrum of Opportunistic Fungal Infections in HIV/AIDS Patients in a Tertiary Care Hospital in India: A Retrospective Study | Kumari, S.; Jahan, N.; Rani, K.S.; Nandan, N. | 2023 | Excluded | Irrelevant, did not discuss the subject matter |
| 1087 | Guidelines for Trypanosoma cruzi-HIV Co-infection and other Immunosuppressive Conditions: Diagnosis, Treatment, Monitoring, and Implementation from the International Network of Care and Studies â€“ 2023 | de Almeida, E.A.; Mendes, F.S.N.S.; JÃºnior, A.N.R.; de Sousa, A.S.; Pavan, T.B.S.; Mediano, M.F.F.; Ostermayer, A.L.; Hasslocher-Moreno, A.M.; de Carvalho Britto, C.F.D.P.; Novaes, C.G.; Correia, D.; Santos, F.L.N.; da Silva, G.M.S.; Fernandez, M.L.; Lima, M.M.; de Carvalho, N.B.; da Cruz Moreira, O.; Albajar-ViÃ±as, P.; Leite, R.M.; Palmeira, S.L.; da Costa, V.M.; Yasuda, M.A.S. | 2023 | Excluded | Irrelevant, did not discuss the subject matter |
| 1088 | Coexistence of Cryptococcal Fungemia and Pneumocystis jirovecii Pneumonia in an HIV-Infected Patient: A Case Report | Tatli KiÅŸ, T.; Yildirim, S.; BiÃ§men, C.; YÃ¼cel, N.; Kirakli, C. | 2023 | Excluded | Irrelevant, did not discuss the subject matter |
| 1089 | Study of Cerebro-Spinal Fluid in HIV Infected Patients at a Tertiary Care Hospital in Maharashtra | Wagh, S.; Ubhale, B. | 2023 | Excluded | Irrelevant, did not discuss the subject matter |
| 1090 | Impacts of improved cryptococcal disease screening in adults with advanced HIV disease in Maswa district hospital | Sita, K.; Mponzi, V.; Mutayoba, R.; Abuya, M.; Kulemba, K.; Mkawe, S.; Mbogo, E.; Kilimba, E.; Kweyamba, S. | 2023 | Excluded | Irrelevant, did not discuss the subject matter |
| 1091 | 9th Tanzania Health Summit | | 2023 | Excluded | Irrelevant, did not discuss the subject matter |
| 1092 | Etiologies of fever of unknown origin in HIV/AIDS patients, Hanoi, Vietnam | Nguyen, T.K.; Nguyen, Y.H.; Nguyen, H.T.; Khong, Q.M.; Tran, N.K. | 2022 | Excluded | Irrelevant, did not discuss the subject matter |
| 1093 | Ventriculoperitoneal shunt is associated with increased cerebrospinal fluid protein level in HIV-infected cryptococcal meningitis patients | Tao, R.; Xu, L.; Guo, Y.; Xu, X.; Zheng, J.; Zhu, B. | 2022 | Excluded | Irrelevant, did not discuss the subject matter |
| 1094 | Patient characteristics and outcome of CD20-positive HIV-associated lymphoma: a single-center KwaZulu-Natal, South African hospital 12-year retrospective review | Rapiti, N.; Abdelatif, N.; Rapiti, A.; Moosa, M.-Y. | 2022 | Excluded | Irrelevant, did not discuss the subject matter |
| 1095 | Persistent High Burden and Mortality Associated With Advanced HIV Disease in Rural Tanzania Despite Uptake of World Health Organization "Test and Treat" Guidelines | StÃ¶ger, L.; Katende, A.; Mapesi, H.; Kalinjuma, A.V.; van Essen, L.; Klimkait, T.; Battegay, M.; Weisser, M.; Letang, E. | 2022 | Excluded | Irrelevant, did not discuss the subject matter |
| 1096 | Oral Encochleated Amphotericin B for Cryptococcal Meningitis: a Phase II Randomized Trial | Atukunda, M.; Kagimu, E.; Rutakingirwa, M.K.; Tugume, L.; Nsangi, L.; Musubire, A.; Gakuru, J.; Mugabi, T.; Akampurira, A.; Ssebambulidde, K.; Kasibante, J.; Ellis, J.; Mpoza, E.; Williams, D.A.; Fieberg, A.M.; Skipper, C.; Abassi, M.; Hullsiek, K.H.; Meya, D.; Boulware, D.R. | 2022 | Excluded | Irrelevant, did not discuss the subject matter |
| 1097 | Timing of antiretroviral therapy prior to diagnosis of cryptococcal meningitis | Chang, B.; Wele, A.; Atoma, B.; Kagimu, E.; Muzoora, C.; Musubire, A.; Boulware, D.R.; Meya, D. | 2022 | Excluded | Irrelevant, did not discuss the subject matter |
| 1098 | Metagenomic next-generation sequencing for identification of central nervous system pathogens in HIV-infected patients | Zhu, Y.; Zhao, W.; Yang, X.; Zhang, Y.; Lin, X.; Weng, X.; Wang, Y.; Cheng, C.; Chi, Y.; Wei, H.; Peng, Z.; Hu, Z. | 2022 | Excluded | Irrelevant, did not discuss the subject matter |
| 1099 | The Roles of CD44 Membrane Ruffling and Signaling in Leukocyte Transmigration across Cryptococcus Neoformans-Coinfected Blood-Brain Barrier with HIV-1 gp41 in Vitro | Huang, P.; Gao, X.; Zou, J.; Chen, J.; He, X.; Long, M.; Cao, H. | 2022 | Excluded | Irrelevant, did not discuss the subject matter |
| 1100 | HIV-infected patients rarely develop invasive fungal diseases under good immune reconstitution after ART regardless high prevalence of pathogenic filamentous fungi carriage in nasopharynx/oropharynx | Chen, X.; Cao, Y.; Chen, M.; Wang, H.; Du, P.; Li, H.; Zhong, H.; Li, Q.; Zhao, S.; Yao, Z.; Chen, W.; Cai, W.; Tang, X.; Li, L. | 2022 | Excluded | Irrelevant, did not discuss the subject matter |
| 1101 | A post-mortem analysis of tenofovir, lamivudine, efavirenz and fluconazole penetration in female genital tissues | Wang, F.; Namuju, O.C.; Pastick, K.A.; Abdusalaamu, K.; Mishra, U.; Collins, L.; Boulware, D.R.; Lukande, R.; Meya, D.B.; Nicol, M.R. | 2022 | Excluded | Irrelevant, did not discuss the subject matter |
| 1102 | Preparing for Transplant â€‘ Screening and Prophylaxis of Donor and Recipients before Solid Organ Transplantation: Expert Group Opinion from South Asia | Bansal, S.B.; Kher, V.; Ramsubramanian, V.; Choudhary, N.S.; Kotton, C.N. | 2022 | Excluded | Irrelevant, did not discuss the subject matter |
| 1103 | Disseminated cryptococcosis in Namibia | Mugabe, M.C.; Kidaaga, F.; Hategekimana, J.; Laga, A. | 2022 | Excluded | Irrelevant, did not discuss the subject matter |
| 1104 | Case of Concomitant CNS Coccidioidomycosis and Cytomegalovirus Infection | Agyei, P.; Hasbun, R.; Gupta, R. | 2022 | Excluded | Irrelevant, did not discuss the subject matter |
| 1105 | Prevalence of cryptococcal meningitis among people living with human immuno-deficiency virus and predictors of mortality in adults on induction therapy in Africa: A systematic review and meta-analysis | Muzazu, S.G.Y.; Assefa, D.G.; Phiri, C.; Getinet, T.; Solomon, S.; Yismaw, G.; Manyazewal, T. | 2022 | Excluded | Irrelevant, did not discuss the subject matter |
| 1106 | Access to flucytosine for the treatment of HIV-associated cryptococcal meningitis in Africa | Temfack, E.; Lortholary, O. | 2022 | Excluded | Irrelevant, did not discuss the subject matter |
| 1107 | Advancing the prevention and treatment of HIV in children: priorities for research and development | Penazzato, M.; Townsend, C.L.; Hafiz, A.; Archary, M.; Bekker, A.; Capparelli, E.V.; Colbers, A.; Cressey, T.R.; Frigati, L.; Mukui, I.; Rakhmanina, N.; Ruel, T.D.; Sam-Agudu, N.A.; Sugandhi, N.; Abrams, E.J.; Rojo, P.; Vicari, M.; Watkins, M.; Abrams, E.; Capparelli, E.; Cressey, T.; Ruel, T.; Sam-Agudu, N.; Townsend, C. | 2022 | Excluded | Irrelevant, did not discuss the subject matter |
| 1108 | Molecular epidemiology of Cryptococcosis-over 14 years using Whole Genome Sequencing and Multi-Locus Se-quence Typing | Michael, J.S. | 2022 | Excluded | Irrelevant, did not discuss the subject matter |
| 1109 | Case series of Cryptococcal Meningitis-Experience in North Western India over 1 year (202122) | Naguthevar, S.; Garg, M.K.; Bohra, G.K.; Jain, V.; Kumar, D.; Midha, N.K.; Meena, D.S.; Sharma, S.; Akshatha, R.; Singh, K.; Kombade, S.P. | 2022 | Excluded | Irrelevant, did not discuss the subject matter |
| 1110 | An unusual recurrent case of Cryptococcal sacroilitis in an immunocompetent elderly female in Rajasthan, India | Choudhary, S.; Mishra, R.K.; Vyas, A.; Pathan, N.; Gupta, K. | 2022 | Excluded | Irrelevant, did not discuss the subject matter |
| 1111 | Fungal meningitis in an immunocompetent individual | Ningthoujam, P.; Khuraijam, R.; Konjengbam, O.; Bhattacharjee, S. | 2022 | Excluded | Irrelevant, did not discuss the subject matter |
| 1112 | Cryptococcus ne oformans-and Cryptococcus gattii-specific antibodies vary among children and adults with crypto-coccosis and healthy from Colombia | Becerra-Alvarez, P.; EscandÃ³n, P.; Lizarazo, J.; QuirÃ³s-GÃ³mez, Ã“.; Firacative, C. | 2022 | Excluded | Irrelevant, did not discuss the subject matter |
| 1113 | Disseminated Histoplasmosis in a Ghanaian HIV Patient: Role of Urine Histoplasma Antigen Testing in Rapid Diag-nosis | Agyei, M.; Ocansey, B.; Otoo, B.; Ofori, A.; Norman, B.; Erskine, I. | 2022 | Excluded | Irrelevant, did not discuss the subject matter |
| 1114 | Evaluation of new tools for the diagnosis of histoplasmosis | Leclere, A.S.; Garcia-Hermoso, D.; Alanio, A.; Donald, S.M.; Vreden, S.; Van Eer, M.; Moussiegt, A.; Nacher, M.; Lortholary, O.; Adenis, A.; Lanternier, F. | 2022 | Excluded | Irrelevant, did not discuss the subject matter |
| 1115 | Reliability of bedside point-of-care tests for Candida neoformans, M. tuberculosis and S. pneumoniae in adults living with HIV presenting with suspected central nervous system infection (CNS) in low-and middle-income settings: Preliminary results from the | Leclere, A.S.; Beaumont, E.; Cohen, J.F.; Kanyama, C.; Mfinanga, S.; Kouanfack, C.; Lesikari, S.; Nyirenda, S.; Phiri, S.; Boyer-Chammard, T.; Molloy, S.; Hosseinipour, M.; Bradley, J.; Jaffar, S.; Harrison, T.; Lortholary, O.; Loyse, A. | 2022 | Excluded | Irrelevant, did not discuss the subject matter |
| 1116 | Incidence of Histoplasmosis, Cryptococcosis, and TB among People Living with HIV in Paraguay-Preliminary Report | Samudio, T.; Aguilar, G.; Sued, O.; LÃ³pez, G.; Jordan, A.; CÃ¡ceres, D.H.; Ovelar, P.; Cardozo, M.; Vicenti, C.; Lezcano, V.; Pereira, J.; Rios-Gonzalez, C.; Munoz, S.; Figueredo, J.; Taboada, A.; Chiller, T.; BenÃ­tez, G. | 2022 | Excluded | Irrelevant, did not discuss the subject matter |
| 1117 | Burden of serious fungal infections in India | Ray, A.; Adarsh Aayilliath, K.; Banerjee, S.; Chakrabarti, A.; Denning, D.W. | 2022 | Excluded | Irrelevant, did not discuss the subject matter |
| 1118 | Hiv-positive, solid organ transplant (SOT), and non-Hiv-positive/non-transplant (NHNT) associated with cryptococ-cosis in Brazil: First national multicenter cohort study | Cavassin, F.B.; Vidal, J.E.; Godoy, C.S.M.; Soares, R.B.A.; Magri, M.M.C.; Falci, D.R.; De Oliveira, C.S.; Mendes, A.V.A.; BaÃº-Carneiro, J.L.; Queiroz-Telles, F. | 2022 | Excluded | Irrelevant, did not discuss the subject matter |
| 1119 | Trend of cryptococcal meningitis in patients attending a teachinghospital in north-east india -a single center study | Acharjee, A.; Ngangom, L.; Hari Presanambika, R.; Ningthoujam, P.; Khuraijam, R. | 2022 | Excluded | Irrelevant, did not discuss the subject matter |
| 1120 | Anti-granulocyte-macrophagecolony-stimulating factor (Anti-GM-CSF) autoantibodies-theunderrecognizedcause of Cryptococcosis in non-HIV individuals in Thailand: Case series from a single tertiary care hospital | Pongdumbun, T.; Treebupachatsakul, P.; Umrod, P.; Tantibhedhyangkul, W.; Chayakulkeeree, M. | 2022 | Excluded | Irrelevant, did not discuss the subject matter |
| 1121 | Biomarker detection for the diagnosis of disseminated histoplasmosis in people living with HIV/ AIDS in Southern Brazil: A year of implementation | Blan, B.; Basso, R.; Poester, V.; Benelli, J.; Sanchotene, K.; Xavier, M. | 2022 | Excluded | Irrelevant, did not discuss the subject matter |
| 1122 | Semi-quantitative cryptococcal antigen rapid test (CryptoPS, BiosynexÂ® ) for cryptococcal meningitis in patients living with HIV in Sub-Saharan Africa: Prospective multicenter diagnostic accuracy study (DREAMM) | Leclere, A.S.; Beaumont, E.; Kanyama, C.; Mfinanga, S.; Kouanfack, C.; Lesikari, S.; Nyirenda, S.; Phiri, S.; Boyer-Chammard, T.; Molloy, S.; Cohen, J.F.; Hosseinipour, M.; Bradley, J.; Jaffar, S.; Harrison, T.; Lortholary, O.; Loyse, A. | 2022 | Excluded | Irrelevant, did not discuss the subject matter |
| 1123 | Rise in HIVnegativeCryptococcal infection in liverdisease patient: Epidemiology, riskfactor, antifungalsusceptibility profile from tertiary care hepatobiliary center | Patel, D.; Kale, P.; Khillan, V.; Sarin, S.K. | 2022 | Excluded | Irrelevant, did not discuss the subject matter |
| 1124 | ISHAM 2022 Congress Abstracts | | 2022 | Excluded | Irrelevant, did not discuss the subject matter |
| 1125 | Disseminated cryptococcosis in HIV due to different species -dissimilar yet alike! | Madan, S.; Patel, A. | 2022 | Excluded | Irrelevant, did not discuss the subject matter |
| 1126 | Umbilicated papules in disseminated cryptococcosis | Mitaka, H.; Perlman, D.C. | 2022 | Excluded | Irrelevant, did not discuss the subject matter |
| 1127 | Evaluation of the effectiveness of a South African laboratory cryptococcal antigen screening programme using a retrospective cohort and a cluster-randomised trial design | Desanto, D.J.; Bangdiwala, A.S.; Van Schalkwyk, E.; Skipper, C.P.; Greene, G.; Paxton, J.; Huppler Hullsiek, K.; Mashau, R.; Rajasingham, R.; Boulware, D.R.; Govender, N.P. | 2022 | Excluded | Irrelevant, did not discuss the subject matter |
| 1128 | Diagnostic and Prognostic Value of Cerebrospinal Fluid Lactate and Glucose in HIV-Associated Tuberculosis Meningitis | Nuwagira, E.; Hullsiek, K.H.; Jjunju, S.; Rutakingirwa, M.; Kasibante, J.; Tadeo, K.K.; Kagimu, E.; Tugume, L.; Ssebambulidde, K.; Musubire, A.K.; Bangdiwala, A.; Muzoora, C.; Meya, D.B.; Boulware, D.R.; Bahr, N.C.; Creswell, F.V. | 2022 | Excluded | Irrelevant, did not discuss the subject matter |
| 1129 | Time to occurrence, predictors, and patterns of opportunistic infections incidence among HIV-positive patients attending Antiretroviral Therapy Clinic of Salale University Comprehensive Specialized Hospital: A retrospective cohort study | Girma, D.; Dejene, H.; Adugna Geleta, L.; Tesema, M.; Bati, F. | 2022 | Excluded | Irrelevant, did not discuss the subject matter |
| 1130 | Prior Pulmonary Tuberculosis Is a Risk Factor for Asymptomatic Cryptococcal Antigenemia in a Cohort of Adults with Advanced Human Immunodeficiency Virus Disease | Wake, R.M.; Ismail, N.A.; Omar, S.V.; Ismail, F.; Tiemessen, C.T.; Harrison, T.S.; Jarvis, J.N.; Govender, N.P. | 2022 | Excluded | Irrelevant, did not discuss the subject matter |
| 1131 | Study of clinical profile and outcomes in progressive multifocal leukoencephalopathy in acquired immunodeficiency syndrome patients in the highly active antiretroviral therapy era - An observational study | Arora, S.; Ahmad, F.M.H.; Deshwal, R.; Behal, P. | 2022 | Excluded | Irrelevant, did not discuss the subject matter |
| 1132 | Disseminated histoplasmosis as a presenting manifestation in an HIV patient - A case report from South India | Devi, G.A.K.; Rajamohanan, R.R.; Sakkaravarthi, V.; Toi, P.C.; Munisamy, M. | 2022 | Excluded | Irrelevant, did not discuss the subject matter |
| 1133 | Central nervous system cryptococcosis prevalence in a tertiary care facility | Lavanya, D.; Surapaneni, N. | 2022 | Excluded | Irrelevant, did not discuss the subject matter |
| 1134 | Paracoccidioidomycosis in people living with HIV/AIDS: A historical retrospective cohort study in a national reference center for infectious diseases, Rio de Janeiro, Brazil | FalcÃ£o, E.M.; de Macedo, P.M.; Freitas, D.F.S.; Freitas, A.D.; Grinsztejn, B.; Veloso, V.G.; Almeida-Paes, R.; Do Valle, A.C.F. | 2022 | Excluded | Irrelevant, did not discuss the subject matter |
| 1135 | ANTIGEN NEGATIVE CRYPTOCOCCAL MENINGITIS | Padrao, E.M.; Becerra, A.; Castro, M.D.; Bustos, B.; Dieckhaus, K. | 2022 | Excluded | Irrelevant, did not discuss the subject matter |
| 1136 | RUXOLITINIB-ASSOCIATED CRYPTOCOCCOSIS: A SYSTEMATIC REVIEW AND META-ANALYSIS | Gaman, M.-A. | 2022 | Excluded | Irrelevant, did not discuss the subject matter |
| 1137 | Whoâ€™s slipping through the cracks? A comprehensive individual, clinical and health system characterization of people with virological failure on first-line HIV treatment in Uganda and South Africa | Reynolds, Z.; McCluskey, S.M.; Moosa, M.Y.S.; Gilbert, R.F.; Pillay, S.; Aturinda, I.; Ard, K.L.; Muyindike, W.; Musinguzi, N.; Masette, G.; Moodley, P.; Brijkumar, J.; Rautenberg, T.; George, G.; Johnson, B.A.; Gandhi, R.T.; Sunpath, H.; Marconi, V.C.; Bwana, M.B.; Siedner, M.J. | 2022 | Excluded | Irrelevant, did not discuss the subject matter |
| 1138 | Giant lung cryptococcoma in immunocompetent patient: Case report | Mestre-Orozco, L.; VicuÃ±a-GonzÃ¡lez, R.M.; DomÃ­nguez-Sosa, F.R.; LÃ³pez-ValdÃ©s, J.C. | 2022 | Excluded | Irrelevant, did not discuss the subject matter |
| 1139 | Laboratory verification of new commercial lateral flow assays for Cryptococcal antigen (CrAg) detection against the predicate IMMY LFA in a reference laboratory in South Africa | Coetzee, L.-M.; Glencross, D.K. | 2022 | Excluded | Irrelevant, did not discuss the subject matter |
| 1140 | Clinical characteristics, common sites and drug resistance profile in culture-confirmed extrapulmonary TB/HIV co-infection patients, Southwest China | Wang, D.-M.; Li, Q.-F.; Zhu, M.; Xu, Y.-H.; Liao, Y. | 2022 | Excluded | Irrelevant, did not discuss the subject matter |
| 1141 | Effects of undernutrition on opportunistic infections among adults living with HIV on ART in Northwest Ethiopia: Using inverse-probability weighting | Alebel, A.; Demant, D.; Petrucka, P.; Sibbritt, D. | 2022 | Excluded | Irrelevant, did not discuss the subject matter |
| 1142 | THE DREAMM PROJECT: EPIDEMIOLOGICAL FINDINGS and CRYPTOCOCCAL MENINGITIS OUTCOMES | Kanyama, C.; Kouanfack, C.; Mfinanga, S.; Nyirenda, S.; Sturny-Leclere, A.; Beaumont, E.; Lesikari, S.; Boyer-Chammard, T.; Lortholary, O.; Jaffar, S.; Bradley, J.; Molloy, S.; Harrison, T.; Loyse, A. | 2022 | Excluded | Irrelevant, did not discuss the subject matter |
| 1143 | COST-EFFECTIVENESS of the AMBITION REGIMEN for HIV-ASSOCIATED CRYPTOCOCCAL MENINGITIS | Muthoga, C.; Lawrence, D.S.; Meya, D.; Mwandumba, H.; Kanyama, C.; Meintjes, G.; Muzoora, C.; Mosepele, M.; Ndhlovu, C.E.; Shiri, T.; Jaffar, S.; Harrison, T.; Jarvis, J.N.; Niessen, L.W. | 2022 | Excluded | Irrelevant, did not discuss the subject matter |
| 1144 | RAPID SEMI-QUANTITATIVE ANTIGEN TESTING and MORTALITY in CRYPTOCOCCAL MENINGITIS | Leeme, T.B.; Lechiile, K.; Kajanga, C.; Moyo, M.; Mwandumba, H.; Youssouf, N.; Mosepele, M.; Mbangiwa, T.; Alanio, A.; Sturny-Leclere, A.; Boyer-Chammard, T.; Lortholary, O.; Lawrence, D.S.; Harrison, T.; Jarvis, J.N. | 2022 | Excluded | Irrelevant, did not discuss the subject matter |
| 1145 | ASSOCIATION of PLASMA BIOMARKERS with EARLY MORTALITY in ADVANCED HIV INFECTION | Riitho, V.; Connon, R.; Gwela, A.; Namusanje, J.; Nhema, R.; Siika, A.; Hakim, J.; Musiime, V.; Berkley, J.A.; Szubert, A.; Klein, N.; Walker, A.S.; Gibb, D.; Prendergast, A.J. | 2022 | Excluded | Irrelevant, did not discuss the subject matter |
| 1146 | The Correlated Risk Factors for Severe Liver Damage Among HIV-Positive Inpatients With Abnormal Liver Tests | Liu, S.; Zhou, Y.; Wang, Y.; Li, C.B.; Wang, W.; Lu, X.; Liu, P.; Hu, Q.H.; Wen, Y. | 2022 | Excluded | Irrelevant, did not discuss the subject matter |
| 1147 | Clinical Profile of 24 AIDS Patients with Cryptococcal Meningitis in the HAART Era: A Report from an Infectious Diseases Tertiary Hospital in Western Romania | Marincu, I.; Citu, C.; Vidican, I.; Bratosin, F.; Mares, M.; Suciu, O.; Frent, S.; Bota, A.V.; Timircan, M.; Bratu, M.L.; Grigoras, M.L. | 2022 | Excluded | Irrelevant, did not discuss the subject matter |
| 1148 | Prevalence of Cryptococcaemia in HIV Infected Patients with CD4 Counts of â‰¤100 Cells/mm3-A Cross Sectional Study in a Tertiary Care Hospital | Kaur, P.; Kau, J. | 2022 | Excluded | Irrelevant, did not discuss the subject matter |
| 1149 | The epidemiological profile of meningitis among adults in a South African district hospital | Terwin, K.; Ferreira, M.; Minnie, C.; Marangellis, K.; Darby, R.; Berlyn, J.; Kleingeld, A.; Tiel, S.; Benedict, M.O.A.; van Rooyen, C.; Adefuye, A.O.; Sempa, J.B. | 2022 | Excluded | Irrelevant, did not discuss the subject matter |
| 1150 | THE CLINICAL FEATURES AND OUTCOMES OF PATIENTS WITH CRYPTOCOCCOSIS AFTER RENAL TRANSPLANTATION | Xu, S.-T.; Li, S.-J.; Chen, J.-S.; Wen, J.-Q.; Hu, W.-X.; Liu, Z.-H. | 2022 | Excluded | Irrelevant, did not discuss the subject matter |
| 1151 | Clinical review of the clinical necessity of lumbar punctures performed on adults at National District Hospital Emergency Department | Geldenhuys, S.; Boltman, C.; Steinberg, W.J.; Botes, J.; van Rooyen, C. | 2022 | Excluded | Irrelevant, did not discuss the subject matter |
| 1152 | Risk factors for ophthalmologic involvement and ocular findings in patients diagnosed with fungemia in a high-complexity hospital in the city of MedellÃ­n, Colombia | Restrepo Arango, M.; Cadavid Usuga, J.C.; Velazquez Ossa, L.F.; Donado GÃ³mez, J.H.; Higuita Duque, L.N.; Neira Gomez, J.P. | 2022 | Excluded | Irrelevant, did not discuss the subject matter |
| 1153 | Diagnosing disseminated histoplasmosis in advanced HIV/AIDS disease in Cameroon using a point of care lateral flow assay | Kuate, M.P.N.; Abessolo Abessolo, H.; Denning, D.W.; Stone, N.R.; Ndip, R.N. | 2022 | Excluded | Irrelevant, did not discuss the subject matter |
| 1154 | Isolated oral histoplasmosis | Delsinne, V.; Chua, V.; Arendt, V.; Staub, T. | 2022 | Excluded | Irrelevant, did not discuss the subject matter |
| 1155 | Characteristics and biomarkers of patients with central nervous system infection admitted to a referral hospital in Northern Vietnam | Ngo, C.C.; Katoh, S.; Hasebe, F.; Dhoubhadel, B.G.; Hiraoka, T.; Hamaguchi, S.; Le, A.T.K.; Nguyen, A.T.H.; Dang, A.D.; Smith, C.; Yoshida, L.-M.; Do, C.D.; Pham, T.T.T.; Ariyoshi, K. | 2021 | Excluded | Irrelevant, did not discuss the subject matter |
| 1156 | Closing gaps in histoplasmosis: clinical characteristics and factors associated with probable/histoplasmosis in HIV/AIDS hospitalized patients, a retrospective cross-sectional study in two tertiary centers in Pereira, Colombia | Hoyos Pulgarin, J.A.; Alzate Piedrahita, J.A.; Moreno GÃ³mez, G.A.; Sierra Palacio, J.F.; OrdoÃ±ez, K.M.; Arias Ramos, D. | 2021 | Excluded | Irrelevant, did not discuss the subject matter |
| 1157 | Genomic variation across a clinical Cryptococcus population linked to disease outcome | Sephton-Clark, P.; Tenor, J.L.; Toffaletti, D.L.; Meyers, N.; Giamberardino, C.; Molloy, S.F.; Palmucci, J.R.; Chan, A.; Chikaonda, T.; Heyderman, R.; Hosseinipour, M.; Kalata, N.; Kanyama, C.; Kukacha, C.; Lupiya, D.; Mwandumba, H.C.; Harrison, T.; Bicanic, T.; Perfect, J.R.; Cuomo, C.A. | 2021 | Excluded | Irrelevant, did not discuss the subject matter |
| 1158 | Environmental Risk Factors for Talaromycosis Hospitalizations of HIV-Infected Patients in Guangzhou, China: Case Crossover Study | Wang, Y.; Deng, K. | 2021 | Excluded | Irrelevant, did not discuss the subject matter |
| 1159 | A global call for talaromycosis to be recognised as a neglected tropical disease | Narayanasamy, S.; Dat, V.Q.; Thanh, N.T.; Ly, V.T.; Chan, J.F.-W.; Yuen, K.-Y.; Ning, C.; Liang, H.; Li, L.; Chowdhary, A.; Youngchim, S.; Supparatpinyo, K.; Aung, N.M.; Hanson, J.; Andrianopoulos, A.; Dougherty, J.; Govender, N.P.; Denning, D.W.; Chiller, T.; Thwaites, G.; van Doorn, H.R.; Perfect, J.; Le, T. | 2021 | Excluded | Irrelevant, did not discuss the subject matter |
| 1160 | Cerebrospinal Fluid Lactate as a Prognostic Marker of Disease Severity and Mortality in Cryptococcal Meningitis | Abassi, M.; Bangdiwala, A.S.; Nuwagira, E.; Kandole Tadeo, K.; Okirwoth, M.; Williams, D.A.; Mpoza, E.; Tugume, L.; Ssebambulidde, K.; Huppler Hullsiek, K.; Musubire, A.K.; Muzoora, C.; Rhein, J.; Meya, D.B.; Boulware, D.R. | 2021 | Excluded | Irrelevant, did not discuss the subject matter |
| 1161 | PULMONARY CRYPTOCOCCOSIS COMPLICATING POST-COVID-19 PULMONARY FIBROSIS | Isaac, S.; Afraz Pasha, M.; Lal, A.; Kyei-Nimako, E. | 2021 | Excluded | Irrelevant, did not discuss the subject matter |
| 1162 | Neuromeningeal cryptococcosis among people living with HIV in Lubumbashi, Democratic Republic of Congo | Kabongo, J.; Mukuku, O.; Wembonyama, S. | 2021 | Excluded | Irrelevant, did not discuss the subject matter |
| 1163 | Central nervous system cryptococcosis mimicking stroke | Thandampallayam, M.; Swafford, K. | 2021 | Excluded | Irrelevant, did not discuss the subject matter |
| 1164 | Gi meets id: two unusual findings on endoscopy leading to the life-saving diagnosis and treatments | Lenik, J.M.; Achuo-Egbe, Y.; Harley, J.M. | 2021 | Excluded | Irrelevant, did not discuss the subject matter |
| 1165 | Cerebrospinal fluid multidisciplinary management in meningoencephalitis. a cryptococcosis case report | BeterÃ© Cubillo, B.; Linares Rufo, M.; Pascual FernÃ¡ndez, A.; Ruiz Ãlvarez, M.J.; GÃ³mez Herruz, P.; PÃ©rez Tanoira, R. | 2021 | Excluded | Irrelevant, did not discuss the subject matter |
| 1166 | Diverse clinical and social circumstances: Developing patient-centred care for dr-tb patients in South Africa | Mitrani, L.; Dickson-Hall, L.; Le Roux, S.; Hill, J.; Loveday, M.; Grant, A.D.; Kielmann, K.; Mlisana, K.; Moshabela, M.; Nicol, M.P.; Black, J.; Cox, H. | 2021 | Excluded | Irrelevant, did not discuss the subject matter |
| 1167 | Cryptococcus early antigenemia in HIV positive asymptomatic patients in Barranquilla, Colombia | Noguera-Angarita, M.C.; EscandÃ³n-Escobar, P.; Silvera-Tapia, O.; Sierra-DÃ­az, M.B.; Torres-Alvarez, N.; de la Hoz, L.C. | 2021 | Excluded | Irrelevant, did not discuss the subject matter |
| 1168 | Integrase strand transfer inhibitor treatment does not increase the incidence of immune reconstitution inflammatory syndrome in HIV-infected Koreans | Kim, J.; Nam, H.-J.; Kang, S.-J.; Jung, S.-I.; Hwang, C.H.; Kim, Y.-S.; Chang, H.-H.; Kim, S.-W.; Park, K.-H. | 2021 | Excluded | Irrelevant, did not discuss the subject matter |
| 1169 | Determining the prevalence of tuberculosis in emergency departments in the Eastern Cape region of South Africa and the utility of the World Health Organization tuberculosis screening tool | Roberts, J.S.; Hahn, E.A.; Black, J.; Maharaj, R.; Farley, J.E.; Redd, A.D.; Reynolds, S.J.; Quinn, T.C.; Hansoti, B. | 2021 | Excluded | Irrelevant, did not discuss the subject matter |
| 1170 | Molecular characterization of Cryptococcus species using Whole genome based MLST: A pilot study | Rachel S, C.; Ninan, M.M.; Venkatesan, M.; Veeraraghavan, B. | 2021 | Excluded | Irrelevant, did not discuss the subject matter |
| 1171 | A 10 year analysis on the course and outcome of Cryptococcal infection | Bhavana, M.V.; Adhikary, R.; Anjana, A.; Beena, H.B. | 2021 | Excluded | Irrelevant, did not discuss the subject matter |
| 1172 | Risk Factors for Stroke in HIV-Positive and-Negative Patients in Pretoria, South Africa | Kroon, L.; van Zyl, D.G.; Schutte, C.M.; Smit, C.; Hiesgen, J. | 2021 | Excluded | Irrelevant, did not discuss the subject matter |
| 1173 | Pulmonary cryptococcus in a 62-year-old patient diagnosed by fine needle aspiration cytology: a case report | Radin, C.P.; De Luna, A. | 2021 | Excluded | Irrelevant, did not discuss the subject matter |
| 1174 | Impact of the COVID-19 pandemic on HIV care in Guatemala | Medina, N.; Alastruey-Izquierdo, A.; Bonilla, O.; OrtÃ­z, B.; Gamboa, O.; Salazar, L.R.; Mercado, D.; PÃ©rez, J.C.; Denning, D.W.; Arathoon, E.; Rodriguez-Tudela, J.L.; PÃ©rez, O.E.L.; Barrientos, B.O.; MuÃ±oz, V.A.R.; Aguilar, G.S.; Andrade, A.M.M.; Marina de LeÃ³n, L.R.S.; AlcÃ¡zar, A.L.G.; GonzÃ¡lez, E.C.; QuiÃ±Ã³nez M, G.A.; Sontay, G.O.C.; MarÃ­n, A.V.C.; de Lourdes Fong Araujo, M.; GuzmÃ¡n, B. | 2021 | Excluded | Irrelevant, did not discuss the subject matter |
| 1175 | Early Empirical Tuberculosis Treatment in HIV-Positive Patients Admitted to Hospital in South Africa: An Observational Cohort Study | Bresges, C.; Wilson, D.; Fielding, K.; Corbett, E.L.; Del-Greco, F.; Grint, D.; Peters, J.; Gupta-Wright, A. | 2021 | Excluded | Irrelevant, did not discuss the subject matter |
| 1176 | Single high-dose liposomal amphotericin based regimen for treatment of HIV-associated Cryptococcal Meningitis: results of the phase-3 Ambition-cm RandomisedTrial | Lawrence, D.S.; Meya, D.B.; Kagimu, E.; Kasibante, J.; Mpoza, E.; Rutakingirwa, M.; Ssebambulidde, K.; Tugume, L.; Rhein, J.; Boulware, D.R.; Mwandumba, H.; Alufandika, M.; Mzinganjira, H.; Kanyama, C.; Hosseinipour, M.C.; Chawinga, C.; Meintjes, G.; Schutz, C.; Comins, K.; Singh, A.; Muzoora, C.; Jjunju, S.; Nuwagira, E.; Mosepele, M.; Leeme, T.; Siamisang, K.; Ndhlovu, C.E.; Hlupeni, A.; Mutata, C.; Dan Widenfelt, E.; Hope, W.; Lortholary, O.; Lalloo, D.G.; Loyse, A.; Chen, T.; Molloy, S.F.; Boyer-Chammard, T.; Wang, D.; Youssouf, N.; Jaffar, S.; Harrison, T.S.; Jarvis, J.N. | 2021 | Excluded | Irrelevant, did not discuss the subject matter |
| 1177 | Tuberculosis in Myasthenia Gravis patients on immunosuppressive therapy in a high-risk area: Implications for preventative therapy | Steyn, E.C.; Naidoo, T.M.; Marais, S.; Heckmann, J.M. | 2021 | Excluded | Irrelevant, did not discuss the subject matter |
| 1178 | Causes and outcomes of hospitalizations among people living with HIV in Georgiaâ€™s referral institution, 2012â€“2017 | Rukhadze, N.; Kirk, O.; Chkhartishvili, N.; Bolokadze, N.; Sharvadze, L.; Gabunia, P.; Lundgren, J.; Tsertsvadze, T. | 2021 | Excluded | Irrelevant, did not discuss the subject matter |
| 1179 | Cryptococcal meningitis in a patient with idiopathic CD4+ T lymphopenia | Esquivel-Luna, J.; Zelada-RÃ­os, L.; Bejarano-Ferreyra, J. | 2021 | Excluded | Irrelevant, did not discuss the subject matter |
| 1180 | AIDS Malignancy Consortium 054: Safety and Immunogenicity of the Quadrivalent Vaccine in Indian Women Living with HIV | Palefsky, J.M.; Poongulali, S.; Lensing, S.; Lee, J.; Da Costa, M.; Chein, A.; Beulah, F.; Murugavel, K.G.; Kumarasamy, N. | 2021 | Excluded | Irrelevant, did not discuss the subject matter |
| 1181 | Predictors of postinfectious inflammatory response syndrome in HIV-negative immunocompetent cryptococcal meningitis | Liu, J.; Luo, C.; Li, M.; Wang, Y.-J.; Xu, X.; Yang, L.; Qin, B.-E.; Chen, Y.; Jiang, Y.; Peng, F. | 2021 | Excluded | Irrelevant, did not discuss the subject matter |
| 1182 | Spectrum of CNS infections in a Tertiary Health Care Centre in Cameroon | Massi, D.G.; Mintyene, M.; Magnerou, A.M.; Eko, S.M.; Doumbe, J.; Njankouo, Y.M. | 2021 | Excluded | Irrelevant, did not discuss the subject matter |
| 1183 | Evaluation of the BioFire FilmArray meningitis/encephalitis multiplex PCR panel in Uganda | Bridge, S.; Hullsiek, K.; Boulware, D.; Rhein, J. | 2021 | Excluded | Irrelevant, did not discuss the subject matter |
| 1184 | Cryptoccus in the immunocompetant host: A great mimicker of neoplasm | Johnson, K.; Dhamrah, U. | 2021 | Excluded | Irrelevant, did not discuss the subject matter |
| 1185 | Ocular manifestations of people living with HIV in Tunisia | Saadouli, D.; Ammari, L.; Mansour, K.B.; Yahyaoui, Y.; Aissa, S.; Ali, E.A.M.; Yahyaoui, S.; Tiouri, H. | 2021 | Excluded | Irrelevant, did not discuss the subject matter |
| 1186 | Cryptococcal Lymphadenitis Without Meningitis in an HIV-Positive Patient: A Rare Case Report | Atashabparvar, A.; Sajjadi, H. | 2021 | Excluded | Irrelevant, did not discuss the subject matter |
| 1187 | Pattern and outcome of opportunistic infections in hospitalized hiv-infected patients in specialist hospital waibargi, Myanmar | Aye, K.-P.; Phyu, S.; Mon, T.-S.; Soe, T.-Z. | 2021 | Excluded | Irrelevant, did not discuss the subject matter |
| 1188 | The dynamics of trust and structural coercion within a meningitis trial in sub-saharan africa | Lawrence, D.; Ssali, A.; Tsholo, K.; Jarvis, J.; Seeley, J. | 2021 | Excluded | Irrelevant, did not discuss the subject matter |
| 1189 | Validation of clinic-based point-of-care testing for cryptococcal antigen screening | Galagan, S.; Govere, S.; Krows, M.; Thulare, H.; Wallis, C.; Gosnell, B.; Moosa, M.-Y.; Bassett, I.V.; Celum, C.L.; Drain, P.K. | 2021 | Excluded | Irrelevant, did not discuss the subject matter |
| 1190 | The epidemiology of advanced hiv disease before and after universal art in Botswana | Lawrence, D.S.; Tenforde, M.W.; Milton, T.; Hurt, W.; Mitchell, H.; Lechiile, K.; Mulenga, F.; Muthoga, C.; Williams, C.G.; Owen, L.; Molefi, M.; Leeme, T.B.; Ngidi, J.; Mine, M.; Jarvis, J.N. | 2021 | Excluded | Irrelevant, did not discuss the subject matter |
| 1191 | Evaluation of Host Serum Protein Biomarkers of Tuberculosis in sub-Saharan Africa | Morris, T.C.; Hoggart, C.J.; Chegou, N.N.; Kidd, M.; Oni, T.; Goliath, R.; Wilkinson, K.A.; Dockrell, H.M.; Sichali, L.; Banda, L.; Crampin, A.C.; French, N.; Walzl, G.; Levin, M.; Wilkinson, R.J.; Hamilton, M.S. | 2021 | Excluded | Irrelevant, did not discuss the subject matter |
| 1192 | Incidence, clinical spectrum, risk factors and outcome of immune reconstitution inflammatory syndrome (IRIS) among HIV patients on highly active anti-retroviral therapy (HAART) in the south west region of Cameroon | Julius, M.; Peter, N.F.; Kebeya, M.C.; Forbinake, N.A.; Stephane, O.C. | 2021 | Excluded | Irrelevant, did not discuss the subject matter |
| 1193 | Effectiveness and usage of flucytosine in clinical practice in India | Bhagat, S.; Gupta, N.; Kareliya, H.; Patil, S.; Barkate, H. | 2021 | Excluded | Irrelevant, did not discuss the subject matter |
| 1194 | Evaluation of a cryptococcal antigen lateral flow assay test for rapid detection of cryptococcal infection in HIV-negative patients in Ibadan, Nigeria | Fayemiwo, S.A.; Makanjuola, O.B.; Nwaokenye, J.; Owolabi, M.O. | 2021 | Excluded | Irrelevant, did not discuss the subject matter |
| 1195 | Ending deaths from HIV-related cryptococcal meningitis by 2030 | Shroufi, A.; Chiller, T.; Jordan, A.; Denning, D.W.; Harrison, T.S.; Govender, N.P.; Loyse, A.; Baptiste, S.; Rajasingham, R.; Boulware, D.R.; Ribeiro, I.; Jarvis, J.N.; Van Cutsem, G. | 2021 | Excluded | Irrelevant, did not discuss the subject matter |
| 1196 | Tongue lesion due to Cryptococcus neoformans as the first finding in an HIV-positive patient | Santiso, G.M.; Messina, F.; Gallo, A.; MarÃ­n, E.; Depardo, R.; Arechavala, A.; Walker, L.; Negroni, R.; Romero, M.M. | 2021 | Excluded | Irrelevant, did not discuss the subject matter |
| 1197 | Profile of presentation of HIV-positive patients to an emergency department in Johannesburg, South Africa | Laher, A.E.; Venter, W.D.F.; Richards, G.A.; Paruk, F. | 2021 | Excluded | Irrelevant, did not discuss the subject matter |
| 1198 | Hematological parameters abnormalities and associated factors in HIV-positive adults before and after highly active antiretroviral treatment in Goba Referral Hospital, southeast Ethiopia: A cross-sectional study | Duguma, N.; Tesfaye Kiya, G.; Adissu Maleko, W.; Bimerew, L.G. | 2021 | Excluded | Irrelevant, did not discuss the subject matter |
| 1199 | Presentation, Clinical Characteristics, and Treatment Outcomes among Tuberculous Meningitis Patients with and Without HIV Infection at Vajira Hospital, Thailand: A Retrospective Cohort Study | Boonyagars, L.; Sangketchon, C.; Pholtawornkulchai, K. | 2021 | Excluded | Irrelevant, did not discuss the subject matter |
| 1200 | Prevalence of and factors associated with reoccurrence of opportunistic infections among adult hiv/aids patients attending the art clinic at public health facilities in arba minch town, southern Ethiopia | Dembelu, M.; Woseneleh, T. | 2021 | Excluded | Irrelevant, did not discuss the subject matter |
| 1201 | COVID-19, hiv-associated cryptococcal meningitis, disseminated tuberculosis and acute ischaemic stroke: A fatal foursome | Bongomin, F.; Sereke, S.G.; Okot, J.; Katsigazi, R.; Kandole, T.K.; Oriekot, A.; Olum, R.; Atukunda, A.; Baluku, J.B.; Nakwagala, F. | 2021 | Excluded | Irrelevant, did not discuss the subject matter |
| 1202 | Clinical manifestations in an immunocompetent patient with Cryptococcal meningoencephalitis: Case report and literature review | De La Luz Portillo, M.I.; Esparza Miranda, L.A.; PÃ©rez GonzÃ¡les, L.F. | 2021 | Excluded | Irrelevant, did not discuss the subject matter |
| 1203 | KAPOSI'S SARCOMA : A REVEALING FORM OF HIV INFECTION | Chaabouni, H.; Elleuch, E.; Smaoui, F.; Allouche, H.; Marrakchi, C.; Hammami, B.; Lahiani, D.; Benjemaa, M. | 2021 | Excluded | Irrelevant, did not discuss the subject matter |
| 1204 | A proposed algorithm for diagnosis of subacte and chronic meningoencephalitis cases in India: An evidence based work up | Pandey, S.; Reddy, H.; Gupta, P.; Jain, A. | 2020 | Excluded | Irrelevant, did not discuss the subject matter |
| 1205 | A retrospective study on the frequency of cryptococcosis in University Malaya Medical Centre from year 2013 to September 2019 | Guna Segaran, S.D.; Sockalingam, R.D.; Na, S.L.; Sri La Sri Ponnampalavanar, S.; Raja Azwa, R.I.S.; Velayuthan, R.D. | 2020 | Excluded | Irrelevant, did not discuss the subject matter |
| 1206 | Clinical and mycologycal characteristics of AIDS patients with meningeal cryptococcosis in Jakarta, Indonesia | Adawiyah, R.; Imran, D.; Rozaliyani, A.; Estiasari, R.; Maharani, K.; Natriana, T.; Syam, R.; Tugiran, M.; Boekhout, T.; Theelen, B.; Wahyuningsih, R. | 2020 | Excluded | Irrelevant, did not discuss the subject matter |
| 1207 | Epidemiology of Cryptococcal antigenemia among HIV infected patients in South-western Nigeria | Odegbemi, O.; Dada-Adegbola, H.; Adeoye, I.; Fayemiwo, S.A. | 2020 | Excluded | Irrelevant, did not discuss the subject matter |
| 1208 | gp120 enhances Cryptococcus neoformans-mediated blood-brain barrier disruption in an in vitro human brain microvascular endothelial cell model | Cao, H.D.; Xie, J.F.; Lin, Q.X.; Wan, Y.; Gong, Z.; Long, M.; Cao, H. | 2020 | Excluded | Irrelevant, did not discuss the subject matter |
| 1209 | Cryptococcal antigenemia and susceptibility of Cryptococcuss neoformans to antifungal agents in HIV infection | Atayese, A.O.; Ishola, R.O.; Banjo, T.A. | 2020 | Excluded | Irrelevant, did not discuss the subject matter |
| 1210 | Short- and medium-term prognosis of HIV-infected patients receiving intensive care: a Brazilian multicentre prospective cohort study | Andrade, H.B.; da Silva, I.R.F.; Ramos, G.V.; Medeiros, D.M.; Ho, Y.L.; de Carvalho, F.B.; Bozza, F.A.; JapiassÃº, A.M. | 2020 | Excluded | Irrelevant, did not discuss the subject matter |
| 1211 | Hospital-acquired SARS-CoV-2 pneumonia in a person living with HIV | Chiappe Gonzalez, A.J.; Montenegro-Idrogo, J.J.; Vargas Vadillo, A.R.; Slee Torres, M.; Vargas Matos, I.; ResurrecciÃ³n Delgado, C.P. | 2020 | Excluded | Irrelevant, did not discuss the subject matter |
| 1212 | Cryptococcosis and Tuberculosis Coinfection in a Regional Reference Service of HIV-AIDS from Southern Brazil | da Silva, L.B.; Klafke, G.B.; Poester, V.R.; Sanchotene, K.O.; Ramis, I.B.; da Silva, P.E.A.; Basso, R.P.; Silveira, J.M.; Xavier, M.O. | 2020 | Excluded | Irrelevant, did not discuss the subject matter |
| 1213 | The Enduring Burden of Advanced Human Immunodeficiency Virus Disease | Ford, N.; Goemaere, E.; Hildebrand, K.; Perez-Casas, C. | 2020 | Excluded | Irrelevant, did not discuss the subject matter |
| 1214 | Comparative analysis of clinical and laboratory parameters and assessment of antiretroviral therapy effectiveness in HIV-associated cryptococcal meningoencephalitis in the case of immune inflammation syndrome | Mustafaeva, D.A.; Tuychiev, L.N.; Musabaev, E.I.; Zaytseva, T.S.; Mukhamedov, K.S. | 2020 | Excluded | Irrelevant, did not discuss the subject matter |
| 1215 | Csf cryptococcus antigen test, trends and epidemiology | Edara, S.; Bathina, P. | 2020 | Excluded | Irrelevant, did not discuss the subject matter |
| 1216 | Epidemiology and outcomes for hiv patients with cryptococcal meningitis | Edara, S.; Bathina, P. | 2020 | Excluded | Irrelevant, did not discuss the subject matter |
| 1217 | The prevalence of HIV among hospitalized persons with acute febrile illness in rural Uganda, August 2019-June 2020 | Blair, P.W.; Kobba, K.; Robinson, M.; Candia, E.; Aniku, G.; Matovu, A.; Kakooza, F.; Walwema, R.; Lamorde, M.; Manabe, Y.C. | 2020 | Excluded | Irrelevant, did not discuss the subject matter |
| 1218 | Advanced HIV disease among adults in the African cohort study (AFRICOS) | Oboho, I.; Esber, A.L.; Dear, N.; Paulin, H.; Iroezindu, M.; Bahemana, E.; Kibuuka, H.; Owuoth, J.; Maswai, J.; Crowell, T.A.; Ake, J.A.; Polyak, C. | 2020 | Excluded | Irrelevant, did not discuss the subject matter |
| 1219 | Evaluation of a novel lateral flow assay for the diagnosis of Cryptococcus neoformans infection | Schub, T.; Forster, J.; Wagener, J.; Dichtl, K. | 2020 | Excluded | Irrelevant, did not discuss the subject matter |
| 1220 | Cytomegalovirus viremia associated with increased mortality in cryptococcal meningitis in sub-saharan Africa | Skipper, C.; Schleiss, M.R.; Bangdiwala, A.S.; Hernandez-Alvarado, N.; Taseera, K.; Nabeta, H.W.; Musubire, A.K.; Lofgren, S.M.; Wiesner, D.L.; Rhein, J.; Rajasingham, R.; Schutz, C.; Meintjes, G.; Muzoora, C.; Meya, D.B.; Boulware, D.R. | 2020 | Excluded | Irrelevant, did not discuss the subject matter |
| 1221 | Effect of AIDS-defining events at initiation of antiretroviral therapy on long-term mortality of HIV/AIDS patients in Southwestern China: A retrospective cohort study | Huang, Y.; Zhou, O.; Zheng, Z.; Xu, Y.; Shao, Y.; Qin, C.; Qin, F.; Lai, J.; Liu, H.; Chen, R.; Ye, L.; Liang, H.; Qin, X.; Jiang, J. | 2020 | Excluded | Irrelevant, did not discuss the subject matter |
| 1222 | Framework for the implementation of advanced HIV disease diagnostics in sub-Saharan Africa: programmatic perspectives | Ndlovu, Z.; Burton, R.; Stewart, R.; Bygrave, H.; Roberts, T.; Fajardo, E.; Mataka, A.; Szumilin, E.; Kerschberger, B.; Van Cutsem, G.; Ellman, T. | 2020 | Excluded | Irrelevant, did not discuss the subject matter |
| 1223 | What is AIDS in the Amazon and the Guianas in the 90-90-90 era? | Nacher, M.; Adenis, A.; Guarmit, B.; Lucarelli, A.; Blanchet, D.; Demar, M.; Djossou, F.; Abboud, P.; Epelboin, L.; CouppiÃ©, P. | 2020 | Excluded | Irrelevant, did not discuss the subject matter |
| 1224 | Concomitant cryptococcal meningitis and African tick bite fever in a returning traveler | Mupfumira, T.D.; Johnson, J.D.; Mupfumira, R.R.; Raney, B.L.; Hofinger, D.; Rukov, B. | 2020 | Excluded | Irrelevant, did not discuss the subject matter |
| 1225 | Toxoplasmicencephalitis (TE)-role of earlyempirical treatment in diagnosis | Shi, Y.; Mathew, A.; Byrd, K.; Cunha, C. | 2020 | Excluded | Irrelevant, did not discuss the subject matter |
| 1226 | Systematic or test-guided treatment for tuberculosis in HIV-infected adults | Blanc, F.-X.; Badje, A.D.; Bonnet, M.; Gabillard, D.; Messou, E.; Muzoora, C.; Samreth, S.; Nguyen, B.D.; Borand, L.; Domergue, A.; Rapoud, D.; Natukunda, N.; Thai, S.; Juchet, S.; EholiÃ©, S.P.; Lawn, S.D.; Domoua, S.K.; Anglaret, X.; Laureillard, D. | 2020 | Excluded | Irrelevant, did not discuss the subject matter |
| 1227 | Virtual croi 2020: Tuberculosis and coinfections in hiv infection | Kerkhoff, A.D.; Havlir, D.V. | 2020 | Excluded | Irrelevant, did not discuss the subject matter |
| 1228 | Clinicopathological features of isolated pulmonary cryptococcosis in HIV-negative patients | Wu, H.-H.; Chen, Y.-X.; Fang, S.-Y. | 2020 | Excluded | Irrelevant, did not discuss the subject matter |
| 1229 | Evaluation of Clinical Profile, Diagnostic Tests, And Prognosis of Cryptococcal Meningitis in HIV Infected Patients in Western India | Kumar, D.; Bishnoi, S.; Meena, D.S.; Bohra, G.K.; Midha, N.; Chhabra, V.; Bhambu, S.K. | 2020 | Excluded | Irrelevant, did not discuss the subject matter |
| 1230 | Neurological presentations of HIV infection: A retrospective observational study in Hong Kong | Cheung, C.H.; Sheng, B.; Chu, Y.P.; Wong, W.T.; Kwan, H.H.; Ng, L.P.; Lam, Y.L.T. | 2020 | Excluded | Irrelevant, did not discuss the subject matter |
| 1231 | Serum Cryptococcal Antigen Testing in Immunosuppressed HIV-positive Children and Adolescents | Zuma, P.; Ramsamy, Y.; Mlisana, K.; Archary, M. | 2020 | Excluded | Irrelevant, did not discuss the subject matter |
| 1232 | Steady-state pharmacokinetics and early safety data in HIV-infected african children weighing >25 kg after switching to 50 mg film-coated dolutegravir tablets in the odyssey trial | Bollen, P.D.J.; Turkova, A.; Kaudha, E.; Chidziva, E.; Lugemwa, A.; Kekitiinwa, A.; Parker, A.; Shakeshaft, C.; Montero, S.; Colbers, A.; Nanduudu, A.; Mujuru, H.; Makumbi, S.; Amuge, P.; Rojo, P.; Ford, D.; Burger, D.M.; Gibb, D.M. | 2020 | Excluded | Irrelevant, did not discuss the subject matter |
| 1233 | Emergomyces africanus: The mimicking fungus | Moodley, A.; Chateau, A.; Govender, N.; Mosam, A. | 2020 | Excluded | Irrelevant, did not discuss the subject matter |
| 1234 | The disney masquerade: Talaromyces marneffei | Moodley, A.; Moodley, N.; Mosam, A. | 2020 | Excluded | Irrelevant, did not discuss the subject matter |
| 1235 | Opportunistic penicilliosis infection causing intestinal obstruction in people living with HIV complicating antiretroviral therapy | Philip Sridhar, R.; Coelho, V.V.; Roopavathana, B.; Chase, S. | 2020 | Excluded | Irrelevant, did not discuss the subject matter |
| 1236 | Evaluation of a cryptococcal antigen lateral flow assay and the burden of cryptococcal disease in Atlanta, Georgia | Harrington, K.; Wang, Y.; Rebolledo, P.; Liu, Z.; Yang, Q.; Kempker, R. | 2020 | Excluded | Irrelevant, did not discuss the subject matter |
| 1237 | The prevalence of cryptococcal antigenemia in serum of human immunodeficiency viruses-infected patients of iran | Kamali, M.; Tabarsi, P.; Badihi, K.; Mortaz, E. | 2020 | Excluded | Irrelevant, did not discuss the subject matter |
| 1238 | Dichotomy in fatal outcomes in a large cohort of people living with htlv-1 in sÃ£o paulo, brazil | Marcusso, R.M.N.; Van Weyenbergh, J.; de Moura, J.V.L.; Dahy, F.E.; Brasil Matos, A.M.; Haziot, M.E.J.; Vidal, J.E.; Fonseca, L.A.M.; Smid, J.; Assone, T.; Casseb, J.; de Oliveira, A.C.P. | 2020 | Excluded | Irrelevant, did not discuss the subject matter |
| 1239 | Causes of Hospitalization and Death among Newly Diagnosed HIV-Infected Adults in Thailand | Chanto, S.; Kiertiburanakul, S. | 2020 | Excluded | Irrelevant, did not discuss the subject matter |
| 1240 | Magnitude of opportunistic infections and associated factors among HIV-positive adults on art at selected public hospitals in Sidama National Regional State, Southern Ethiopia | Wachamo, D.; Bonja, F. | 2020 | Excluded | Irrelevant, did not discuss the subject matter |
| 1241 | Prevalence of asymptomatic cryptococcal antigenemia and association with follow-up risk of cryptococcal meningitis and mortality among HIV infected patients in north west India: A prospective cohort study | Bhati, R.; Pramendra, S.; Sejoo, B.; Kumar, D.; Bohra, G.K.; Meena, D.S.; Verma, D.; Midha, N.K. | 2020 | Excluded | Irrelevant, did not discuss the subject matter |
| 1242 | Hospitalization and Predictors of Inpatient Mortality among HIV-Infected Patients in Jimma University Specialized Hospital, Jimma, Ethiopia: Prospective Observational Study | Mishore, K.M.; Hussein, N.; Huluka, S.A. | 2020 | Excluded | Irrelevant, did not discuss the subject matter |
| 1243 | Are Italy and Iran really suffering from COVID-19 epidemic? A controversial study | Al-Najjar, H.; Al-Rousan, N. | 2020 | Excluded | Irrelevant, did not discuss the subject matter |
| 1244 | Should cryptococcal antigen screening be considered as a routine procedure in antiretroviral therapy naÃ¯ve severely immunocompromised HIV-seropositives - A prevalence study from Eastern India to support recent 2018 WHO guidelines | Dutta, N.; De, R.G.; Bhowmik, A.; Bhandary, S.; Modak, D.; Guha, S.K. | 2020 | Excluded | Irrelevant, did not discuss the subject matter |
| 1245 | Diabetes mellitus as a risk factor for cryptococcal meningitis in immunocompetent | Acharya, R.; Khanal, K.; Upadhyaya, P.; Kafle, S.; Savaliya, V. | 2020 | Excluded | Irrelevant, did not discuss the subject matter |
| 1246 | Amphotericin B combined with flucytosine and fluconazole in the treatment of non-HIV, non-transplant cryptococcal meningitis: retrospective analysis of 45 cases | Xu, Z.; Deng, W.; Liu, Y. | 2020 | Excluded | Irrelevant, did not discuss the subject matter |
| 1247 | A potpourri of infection: When Fungi, Actinomycete and Bacteria coexist in the same host | Isabella Princess, B. | 2020 | Excluded | Irrelevant, did not discuss the subject matter |
| 1248 | A case of stubborn Cryptococcal meningitis in an immunocompetent patient | Anto, P.V.; Reddy, N.M.; Krishnan, V.; Kindo, A.J. | 2020 | Excluded | Irrelevant, did not discuss the subject matter |
| 1249 | Viral causes of meningitis detected by metagenomic next-generation sequencing in a ugandan tuberculous meningitis cohort | Quinn, C.; Ramachandran, P.; Rutakingirwa, M.; Bangdiwala, A.; Kagimu, E.; Kandole, K.T.; Cresswell, F.; Meya, D.; Boulware, D.; Wilson, M. | 2020 | Excluded | Irrelevant, did not discuss the subject matter |
| 1250 | Urine-based TB screening with TB-LAM and ultra in HIV+ Ugandans with meningitis | Ellis, J.; Kagimu, E.; Bangdiwala, A.; Okirwoth, M.; Mugumya, G.; Wadda, V.; Boulware, D.R.; Bahr, N.C.; Cresswell, F.V. | 2020 | Excluded | Irrelevant, did not discuss the subject matter |
| 1251 | Metagenomic next-generation sequencing for diagnosis of CNS infection in PLWH | Chen, J.; Zhang, R.; Liu, L.; Qi, T.; Wang, Z.; Song, W.; Tang, Y.; Sun, J.; Lu, H. | 2020 | Excluded | Irrelevant, did not discuss the subject matter |
| 1252 | HIV diversity in CSF and plasma of individuals with HIV and cryptococcal meningitis | Kelentse, N.; Moyo, S.; Mogwele, M.L.; Lechiile, K.; Seatla, K.; Moraka, N.O.; Molebatsi, K.; Leeme, T.B.; Lawrence, D.; Musonda, R.; Kasvosve, I.; Harrison, T.S.; Jarvis, J.N.; Gaseitsiwe, S. | 2020 | Excluded | Irrelevant, did not discuss the subject matter |
| 1253 | Diagnostic performance of a semiquantitative point-of-care assay for cryptococcosis | Kiiza, T.K.; Nimwesiga, A.; Skipper, C.P.; Kwizera, R.; Apeduno, L.; Okirwoth, M.; Kafufu, J.B.; Nalintya, E.; Williams, D.A.; Rhein, J.; Meya, D.B.; Boulware, D.R. | 2020 | Excluded | Irrelevant, did not discuss the subject matter |
| 1254 | Evaluating the IMMY semi-quantitative crag LFA in HIV-positive patients in Botswana | Lechiile, K.; Tenforde, M.W.; Milton, T.; Boose, A.; Leeme, T.B.; Tawe, L.; Muthoga, C.; Mulenga, F.; Rulaganyang, I.; Ngidi, J.; Mine, M.; Jarvis, J.N. | 2020 | Excluded | Irrelevant, did not discuss the subject matter |
| 1255 | High rates of meningitis or mortality among CRAG+ PLHIV with CD4 100-200 cells/mm3 | Wykowski, J.H.; Drain, P.K.; Galagan, S.; Govere, S.M.; Celum, C.L.; Moosa, M.-Y.; Wallis, C. | 2020 | Excluded | Irrelevant, did not discuss the subject matter |
| 1256 | The impact and cost-effectiveness of expanding cryptococcal antigen screening to include individuals with CD4 100 to 200 cells/Î¼L in Botswana | Muthoga, C.; Tenforde, M.W.; Ponatshego, P.; Ngidi, J.; Mine, M.; Larson, B.; Jarvis, J.N. | 2020 | Excluded | Irrelevant, did not discuss the subject matter |
| 1257 | The burden of advanced HIV disease in a concentrated HIV epidemic: A historical analysis of the Medecins Sans Frontieres HIV cohort in Myanmar | Homan, T.; Thit, P.; They Mar, H.; Thandar, M.P.; Sangma, M.; Thwe, T.T.; Spina, A.; Kremer, R.; Lenglet, A.; Mesic, A. | 2020 | Excluded | Irrelevant, did not discuss the subject matter |
| 1258 | Latent co-infections increase risk of neurocognitive impairment among older persons with HIV in Lima, Peru | Diaz, M.M.; ZacarÃ­as, M.G.; Bailey, M.A.; Franklin, D.; Cherner, M.; Lanata, S.; Ellis, R.J.; Garcia, P.J. | 2020 | Excluded | Irrelevant, did not discuss the subject matter |
| 1259 | Preliminary findings from the fiebre study in Mozambique: Clinical findings and results of point-of-care tests and microbiology studies | Valente, M.; Ajanovic, S.; Bramugy, J.; Vitorino, P.; Lal, S.; Mabey, D.; Bassat, Q.; Hopkins, H. | 2020 | Excluded | Irrelevant, did not discuss the subject matter |
| 1260 | Assessing gaps in care for HIV-infected people living with aids in two hospitals in Ethiopia | Zewde, A. | 2020 | Excluded | Irrelevant, did not discuss the subject matter |
| 1261 | AIDS-Related Mycoses in the Paediatric Population | Ekeng, B.E.; Olusoga, O.O.; Oladele, R.O. | 2019 | Excluded | Irrelevant, did not discuss the subject matter |
| 1262 | Clinical insights and epidemiology of central nervous system infection due to Cryptococcus neoformans/gattii species complexes: A prospective study from South India | Lahiri, S.; Manjunath, N.; Bhat, M.; Hagen, F.; Bahubali, V.H.; Palaniappan, M.; Maji, S.; Chandrashekar, N. | 2019 | Excluded | Irrelevant, did not discuss the subject matter |
| 1263 | Comparison of HIV-1 viral load and drug resistance mutations between cerebrospinal fluid and plasma in patients with HIV and Cryptococcal meningitis co-infection in Botswana | Kelentse, N.; Moyo, S.; Mogwele, M.; Lechiile, K.; Moraka, N.O.; Maruapula, D.; Esele, L.; Seatla, K.; Leeme, T.B.; Lawrence, D.; Musonda, R.; Kasvosve, I.; Jarvis, J.N.; Gaseitsiwe, S. | 2019 | Excluded | Irrelevant, did not discuss the subject matter |
| 1264 | Cryptococcal and tuberculosis coinfection: Case series identified through the implementation of an advanced HIV disease package of care linked to a TB active case finding strategy in rural Mozambique | Blanco-ArÃ©valo, A.; Salgado, J.; Murias, A.; Catorze, N.; Jordan, A.; Greene, G.; Santiago, I.; Nhampossa, T.; Letang, E. | 2019 | Excluded | Irrelevant, did not discuss the subject matter |
| 1265 | Cryptococcal meningitis in HIV and non-HIV patients - A comparative study of clinical and CSF parameters | Acharya, M.K.; Das, B.; Nahar, N.B. | 2019 | Excluded | Irrelevant, did not discuss the subject matter |
| 1266 | Cerebral neurocisticercosis and crytococcosis in a non-immunocomprommended patient. An unusual association. Literature review and case report. Ecuador | Bonilla, G.; Castrejon, J.; Mejia, I.B. | 2019 | Excluded | Irrelevant, did not discuss the subject matter |
| 1267 | Cerebral venous thrombosis associated with recurrent cryptococcal meningitis in an HIV infected patient | Mohamed, A.; Ali, S.; Kanyi, J.; Gardner, A. | 2019 | Excluded | Irrelevant, did not discuss the subject matter |
| 1268 | Secondary cutaneous cryptococcosis: A molluscum look-a-like and potential missed opportunity | Tourtellotte, L.; Kim, E. | 2019 | Excluded | Irrelevant, did not discuss the subject matter |
| 1269 | An unusual manifestation of an HIV patient with fungimea presenting with cryptococcal lymphadenitis | Mendoza, D.J.C.Z.; Librado, D.R.O. | 2019 | Excluded | Irrelevant, did not discuss the subject matter |
| 1270 | Infectious causes of acute meningitis among Thai adults: A university hospital setting | Aimbudlop, K.; Kiertiburanakul, S. | 2019 | Excluded | Irrelevant, did not discuss the subject matter |
| 1271 | Clinical characteristics and outcomes of cryptococcosis in a tertiary care center in Kentucky, 2005 to 2017 | Bhatt, M.; Ribes, J.A.; Arora, V.; Myint, T. | 2019 | Excluded | Irrelevant, did not discuss the subject matter |
| 1272 | Cryptococcal antigenemia in advanced HIV infection | Ahuja, J.; Soneja, M.; Wig, N.; Xess, I.; Biswas, A.; Singh, G.; Vibha, D.; Nischal, N. | 2019 | Excluded | Irrelevant, did not discuss the subject matter |
| 1273 | Evaluation of a cryptococcal antigen lateral flow assay and the burden of cryptococcal disease: A cohort study at grady memorial hospital in Atlanta, Georgia | Harrington, K.; Wang, Y.; Rebolledo, P.; Liu, Z.; Yang, Q.; Kempker, R.R. | 2019 | Excluded | Irrelevant, did not discuss the subject matter |
| 1274 | Tuberculosis and HIV co-infection at a tertiary care hospital in Thailand | Nimitvilai, S.; Jintanapramote, K. | 2019 | Excluded | Irrelevant, did not discuss the subject matter |
| 1275 | Care Continuum and Postdischarge Outcomes among HIV-Infected Adults Admitted to the Hospital in Zambia | Haachambwa, L.; Kandiwo, N.; Zulu, P.M.; Rutagwera, D.; Geng, E.; Holmes, C.B.; Sinkala, E.; Claassen, C.W.; Mugavero, M.J.; Wa Mwanza, M.; Turan, J.M.; Vinikoor, M.J. | 2019 | Excluded | Irrelevant, did not discuss the subject matter |
| 1276 | Fiebre (febrile illness evaluation in a broad range of endemicities): A multi-site prospective observational study of causes of fever in sub-Saharan Africa and Southeast Asia | Hopkins, H.; Bassat, Q.; Chandler, C.I.R.; Crump, J.A.; Feasey, N.A.; Ferrand, R.A.; Lalloo, D.G.; Mayxay, M.; Newton, P.N.; Mabey, D.C.W. | 2019 | Excluded | Irrelevant, did not discuss the subject matter |
| 1277 | Idiopathic CD4+ T cell lymphocytopenia presenting with atypical meningitis | Sharma, S.; Panda, S.; Jain, S.; Tiwari, S. | 2019 | Excluded | Irrelevant, did not discuss the subject matter |
| 1278 | Advanced HIV: diagnosis, treatment, and prevention | Prabhu, S.; Harwell, J.I.; Kumarasamy, N. | 2019 | Excluded | Irrelevant, did not discuss the subject matter |
| 1279 | The contributions of lay workers in providing home-based treatment adherence support to patients with aids in urban settings: Lessons from the field in Tanzania and Zambia | Kimaro, G.D.; Bottomley, C.; Kahwa, A.; Guinness, L.; Kivuyo, S.; Simms, V.; Ngowi, B.; Chanda, D.; Jaffar, S.; Mfinanga, G.S. | 2019 | Excluded | Irrelevant, did not discuss the subject matter |
| 1280 | MON-280 TENOFOVIR INDUCED FANCONI'S SYNDROME - A CASE STUDY | Dahwa, R.; Taderera, C.; Lowe, S. | 2019 | Excluded | Irrelevant, did not discuss the subject matter |
| 1281 | Prevalence, characteristics and outcomes of patients with Cryptococcal meningitis in Maputo, Mozambique | Deiss, R.; Loreti, C.; Gutierrez, A.; Tatia, M.; Vivaldo, H.; Molfino, L.; Tamayo Antabak, N.; Issufo, S.; Ciglienicki, I. | 2019 | Excluded | Irrelevant, did not discuss the subject matter |
| 1282 | High yield of active tuberculosis case finding among HIV-infected patients using xpert MTB/RIF testing | Kempker, R.R.; Chkhartishvili, N.; Kinkladze, I.; Schechter, M.C.; Harrington, K.; Rukhadze, N.; Dzigua, L.; Tserstvadze, T.; Del Rio, C.; Blumberg, H.M.; Tukvadze, N. | 2019 | Excluded | Irrelevant, did not discuss the subject matter |
| 1283 | Cryptococcal antigenemia among HIV infected patients at a referral hospital, Northwest Ethiopia | Habteyohannes, A.D.; Ayalew, W.; Mekonnen, D.; Alemu, M.; Mulugeta, Y. | 2019 | Excluded | Irrelevant, did not discuss the subject matter |
| 1284 | Prevalence of cryptococcal antigenemia and associated factors among HIV/AIDS patients on second-line antiretroviral therapy at two hospitals in Western Oromia, Ethiopia | Geda, N.; Dabsu, R.; Beyene, T.; Mengist, H.M. | 2019 | Excluded | Irrelevant, did not discuss the subject matter |
| 1285 | Prevalence of cryptococcaemia in HIV seropositive patients in an Indian setting | Vijay, S.; Ingole, N.; Wanjare, S.; Mehta, P. | 2019 | Excluded | Irrelevant, did not discuss the subject matter |
| 1286 | Age-related comorbidities and mortality in people living with HIV in rural Tanzania | Albrecht, S.; Franzeck, F.C.; Mapesi, H.; Hatz, C.; Kalinjuma, A.V.; Glass, T.R.; Mnzava, D.; Letang, E.; Paris, D.H.; Battegay, M.; Weisser, M. | 2019 | Excluded | Irrelevant, did not discuss the subject matter |
| 1287 | False negative serum cryptococcal antigen lateral flow assay test in a patient with disseminated cryptococcal disease | Soni, P.; Sharma, S.; Ghatak, A.; Patti, R.S.; Seneviratne, C.; Yoon, T.; Kupfer, Y. | 2019 | Excluded | Irrelevant, did not discuss the subject matter |
| 1288 | Necrotizing cavitary lung lesion: A case of primary mucinous adenocarcinoma | Hines, A.; Iftikhar, A.; Patel, A.A.; Thakkar, V.R.; Adial, A.; Sanso, L. | 2019 | Excluded | Irrelevant, did not discuss the subject matter |
| 1289 | Cryptococcosis-Associated Immune Reconstitution Inflammatory Syndrome Is Associated with Dysregulation of IL-7/IL-7 Receptor Signaling Pathway in T Cells and Monocyte Activation | Akilimali, N.A.; Muema, D.M.; Specht, C.; Chang, C.C.; Moosa, M.-Y.S.; Levitz, S.M.; Lewin, S.R.; French, M.A.; Ndung'U, T. | 2019 | Excluded | Irrelevant, did not discuss the subject matter |
| 1290 | Cryptococcus meningitis by cryptococcus gattii in a patient with systemic lupus erythematosus | Donadio, P.R.; Grizzo, F.M.F.; Marchiotti, L.B.M.; De Paula Monarin, S.; Ramos, G.B.; De AraÃºjo, M.C.P.; Menegon, R.G.; Abreu, V.C.; De Andrade, P.H.G. | 2019 | Excluded | Irrelevant, did not discuss the subject matter |
| 1291 | Emergomyces africanus: The Mimicking Fungus | Moodley, A.; Mosam, A.; Govender, N.P.; Mahabeer, Y.; Chateau, A.V. | 2019 | Excluded | Irrelevant, did not discuss the subject matter |
| 1292 | Point-of-care C-reactive protein and risk of early mortality among adults initiating antiretroviral therapy | Chaisson, L.H.; Semitala, F.C.; Asege, L.; Mwebe, S.; Katende, J.; Nakaye, M.; Andama, A.O.; Marquez, C.; Atuhumuza, E.; Kamya, M.; Cattamanchi, A.; Yoon, C. | 2019 | Excluded | Irrelevant, did not discuss the subject matter |
| 1293 | ROBUST CLINICAL TRIALS ARE NOT ENOUGH: OVERCOMING OPERATIONAL CHALLENGES FOR IMPLEMENTING REMSTART INTERVENTION PACKAGE (TRIP STUDY) INTO ROUTINE PRACTICE | Kivuyo, S.; Erick, F.; Loyse, A.; Jaffar, S.; Mfinanga, G.S. | 2019 | Excluded | Irrelevant, did not discuss the subject matter |
| 1294 | INTRODUCING A NEW AFRICA MENINGITIS NETWORK - A NORTH-SOUTH COLLABORATION | Mosepele, M.; Kanyama, C.; Meya, D.; Cresswell, F.; Chammard, T.; Mwandumba, H.; Meintjes, G.; Mfinanga, G.S.; Ndhlovu, C.; Jarvis, J. | 2019 | Excluded | Irrelevant, did not discuss the subject matter |
| 1295 | Incidence and risk factors of cardiovascular diseases among HIV patients in Thailand | Sitticharoenchai, P.; Putcharoen, O.; Buddhari, W. | 2019 | Excluded | Irrelevant, did not discuss the subject matter |
| 1296 | Cryptococcal meningitis leading to fatal outcomes in immunocompetent patients: A case study and review of literature | Ramirez-Ramos, C.F.; Salinas-Cortes, D.; Rivera-Marin, J.D.; Peralta-Agudelo, M.; Escobar-Montealegre, F. | 2019 | Excluded | Irrelevant, did not discuss the subject matter |
| 1297 | Barriers to diagnosis and management of CNS infections in Indonesia | Imran, D.; Satiti, S.; Sugianto, P.; Estiasari, R.; Maharani, K.; Pangeran, D.; Andini, P.W.; Munir, B.; Mawuntu, A.H.P.; Susilawathi, N.M.; Ritarwan, K.; Hartanto, O.S.; Frida, M.; Ganiem, A.R.; Gunawan, D.; Dian, S.; Sudewi, A.A.R.; Van Crevel, R. | 2019 | Excluded | Irrelevant, did not discuss the subject matter |
| 1298 | Estimated burden of serious fungal infections in Ethiopia | Denning, D.; Beyene, T. | 2019 | Excluded | Irrelevant, did not discuss the subject matter |
| 1299 | The estimated burden of fungal diseases in South Africa | Schwartz, I.; Denning, D. | 2019 | Excluded | Irrelevant, did not discuss the subject matter |
| 1300 | Prognosis of fungal infection of central nervous system in HIV-infected patients: A retrospective study of 77 patients in Ukraine | Lytvyn, K.Y.; Shostakovych-Koretskaya, L.R.; Doroshenko, A.A.; Andreeva, T.I. | 2019 | Excluded | Irrelevant, did not discuss the subject matter |
| 1301 | Evolutionary prognosis of neuromeningeal cryptococcosis at the university hospital Sylvanus Olympio (Togo) | LÃ©hleng, A.; Guinhouya Kokou, M.; Vincent, K.; Anayo Komla, N.; Mofou, B.; Balogou Agnon, A.K. | 2019 | Excluded | Irrelevant, did not discuss the subject matter |
| 1302 | Prevalence and predictors of opportunistic infections among HIV positive adults on antiretroviral therapy (On-art) versus pre-art in addis ababa, Ethiopia: A comparative cross-sectional study | Dereje, N.; Moges, K.; Nigatu, Y.; Holland, R. | 2019 | Excluded | Irrelevant, did not discuss the subject matter |
| 1303 | Clinico-immunological correlation among hiv-infected persons in North-Western India | Thomas, R.; Loomba, V.; Mary, J. | 2019 | Excluded | Irrelevant, did not discuss the subject matter |
| 1304 | The diagnostic utility of bone marrow examination in an infectious disease ward | Bharuthram, N.; Feldman, C. | 2019 | Excluded | Irrelevant, did not discuss the subject matter |
| 1305 | The estimated burden of fungal disease in South Africa | Schwartz, I.S.; Boyles, T.H.; Kenyon, C.R.; Hoving, J.C.; Brown, G.D.; Denning, D.W. | 2019 | Excluded | Irrelevant, did not discuss the subject matter |
| 1306 | Clinical and immunological outcomes after initiation of second line anti-retroviral therapy in people living with HIV | Achappa, B.; Madi, D.; Yousuff, M.; Rao, S.; Ramapuram, J. | 2019 | Excluded | Irrelevant, did not discuss the subject matter |
| 1307 | Mortality-related risks in treatment-naive hospitalized AIDS patients with opportunistic infections in Southwest China | Du, L.; Lin, Y.; Wang, M.; Yan, L.; Bai, L.; Feng, P.; Tang, H. | 2019 | Excluded | Irrelevant, did not discuss the subject matter |
| 1308 | A review of the management and outcome of patients admitted with cryptococcal meningitis at a regional hospital in KwaZulu-Natal province | Ross, A.J.; Ndayishimiye, E. | 2019 | Excluded | Irrelevant, did not discuss the subject matter |
| 1309 | Cryptococcal antigenemia in HIV patients with virologic failure in Uganda | Mpoza, E.; Meya, D.; Nabaggala, M.S.; Tugume, L.; Ssebambulidde, K.; Rhein, J.; Boulware, D.R.; Rajasingham, R. | 2019 | Excluded | Irrelevant, did not discuss the subject matter |
| 1310 | Asymptomatic talaromyces marneffei antigenemia and mortality in advanced HIV disease | Thu, N.T.; Dat, V.Q.; Chan, J.F.; Ha, H.T.; Nguyen, D.T.; Ho, A.T.; Woo, P.C.; Yuen, K.-Y.; Lyss, S.; Bateganya, M.; Nguyen, K.V.; Le, T. | 2019 | Excluded | Irrelevant, did not discuss the subject matter |
| 1311 | Utility of CD4 cell count monitoring in Botswana: Analysis of routine laboratory data | Leeme, T.B.; Mine, M.; Lechiile, K.; Mosepele, M.; Mphoyakgosi, T.; Muthoga, C.; Ngidi, J.; Nkomo, B.; Ramaabya, D.; Tau, M.; Tenforde, M.W.; Hayes, R.; Jarvis, J.N. | 2019 | Excluded | Irrelevant, did not discuss the subject matter |
| 1312 | 12 month outcomes on dolutegravir-based regimens in Botswana: The beat cohort study | Avalos, A.; Gaolathe, T.; Brown, D.; Vannappaggari, V.; Phillips, H.; Melamu, P.; Ramaabya, D.; Nkomo, B.; Matlho, K.; Seatla, K.; Jarvis, J.N.; Moyo, S.; Matshaba, M.; Gaseitsiwe, S. | 2019 | Excluded | Irrelevant, did not discuss the subject matter |
| 1313 | Screening for talaromyces and cryptococcal antigenemia in aids patients in guangdong | Li, L.; He, Y.; Wang, H.; He, K.; Tang, X.; Le, T.; Cai, W. | 2019 | Excluded | Irrelevant, did not discuss the subject matter |
| 1314 | The cost-effectiveness of ambisome for asymptomatic cryptococcal infection | Rajasingham, R.; Meya, D.; Nalintya, E.; Larson, B.; Boulware, D.R. | 2019 | Excluded | Irrelevant, did not discuss the subject matter |
| 1315 |  | LeÃ£o, A.C.; Santos, M.S.B.; Coelho, A.D.C.; Bertolini, D.V.; Teixeira, D.O.; Yoshioka, L.; Pimentel, S.R.; Antonorsi, S.L.; Vidal, J.E. | 2018 | Excluded | Irrelevant, did not discuss the subject matter |
| 1316 | HIV and maternal deaths: 2014-2016 | Ramogale-Zungu, M.R.; Burton, R. | 2018 | Excluded | Irrelevant, did not discuss the subject matter |
| 1317 | Post malaria neurological syndrome: A rare complication of malaria | Yadava, S.; Fazili, T.; Laleker, A.; Poudel, P. | 2018 | Excluded | Irrelevant, did not discuss the subject matter |
| 1318 | A Case of Massive Cryptococcal Pleurisy and Spontaneous Cryptococcal Peritonitis with Hepatitis B Virus-Related Liver Cirrhosis Cured by Antifungal Therapy | Jung, G.M.; Cho, Y.K.; Yun, S.H.; Song, H.Y. | 2018 | Excluded | Irrelevant, did not discuss the subject matter |
| 1319 | Detection of Mycobacterium tuberculosis in urine by Xpert MTB/RIF Ultra: A useful adjunctive diagnostic tool in HIV-associated tuberculosis | Atherton, R.R.; Cresswell, F.V.; Ellis, J.; Skipper, C.; Tadeo, K.K.; Mugumya, G.; Wadda, V.; Meya, D.B.; Boulware, D.R. | 2018 | Excluded | Irrelevant, did not discuss the subject matter |
| 1320 | Cryptococcal meningitis: an unusual presentation of primary HIV infection | Barletta, J.; Falak, A.; PÃ©rez, H. | 2018 | Excluded | Irrelevant, did not discuss the subject matter |
| 1321 | Performance of novel diagnostics for tuberculous meningitis in Zambia | Siddiqi, O.K.; Love, S.; Mubanga, E.; Atadzhanov, M.; Kosloff, B.; Ayles, H.; Birbeck, G.L.; Koralnik, I.J. | 2018 | Excluded | Irrelevant, did not discuss the subject matter |
| 1322 | Cryptococcal craniospinal meningoradiculitis in an apparently immunocompetent patient | Diop, M.; Gaye, N.-M.; Ka, D.; Dieye, A.; Massaly, A.; Fall, N.-M.; Cisse-Diallo, V.-M.P.; Diallo-Mbaye, K.; Lakhe, N.-A.; Fortes-Deguenonvo, L.; Ndour, C.-T.; Soumare, M.; Seydi, M. | 2018 | Excluded | Irrelevant, did not discuss the subject matter |
| 1323 | Atypical manifestation of disseminated sporotrichosis in an AIDS patient | Ferreira, T.A.; Trope, B.M.; Barreiros, G.; Quintela, D.C.; Ramos-E-Silva, M. | 2018 | Excluded | Irrelevant, did not discuss the subject matter |
| 1324 | Talaromycosis and mucormycosis: Challenges and opportunities | Woo, P. | 2019 | Excluded | Irrelevant, did not discuss the subject matter |
| 1325 | Outcomes of HIV-associated pneumocystis pneumonia at a South African referral hospital | Chiliza, N.; Toit, M.D.; Wasserman, S. | 2018 | Excluded | Irrelevant, did not discuss the subject matter |
| 1326 | Cryptococcus neoformans meningitis with renal involvement in an HIV-positive patient: A case report | SarÄ±gÃ¼l, F.; Ãœser, Ãœ.; Ã–ztoprak, N. | 2018 | Excluded | Irrelevant, did not discuss the subject matter |
| 1327 | Cryptococcal Meningitis and Tuberculous Meningitis Co-infection in HIV-Infected Ugandan Adults | Ellis, J.; Cresswell, F.V.; Rhein, J.; Ssebambulidde, K.; Boulware, D.R. | 2018 | Excluded | Irrelevant, did not discuss the subject matter |
| 1328 | Early versus delayed antiretroviral treatment in HIV-positive people with cryptococcal meningitis | Eshun-Wilson, I.; Okwen, M.P.; Richardson, M.; Bicanic, T. | 2018 | Excluded | Irrelevant, did not discuss the subject matter |
| 1329 | Prediction of unfavorable outcomes in cryptococcal meningitis: results of the multicenter Infectious Diseases International Research Initiative (ID-IRI) cryptococcal meningitis study | Hakyemez, I.N.; Erdem, H.; Beraud, G.; Lurdes, M.; Silva-Pinto, A.; Alexandru, C.; Bishop, B.; Mangani, F.; Argemi, X.; Poinot, M.; Hasbun, R.; Sunbul, M.; Akcaer, M.; Alp, S.; Demirdal, T.; Angamuthu, K.; Amer, F.; Ragab, E.; Shehata, G.A.; Ozturk-Engin, D.; Ozgunes, N.; Larsen, L.; Zimmerli, S.; Sipahi, O.R.; Tukenmez Tigen, E.; Celebi, G.; Oztoprak, N.; Yardimci, A.C.; Cag, Y. | 2018 | Excluded | Irrelevant, did not discuss the subject matter |
| 1330 | Multiple facial ulcers: Do you smell a rat? | Kuan, L.Y.; Pan, J.Y. | 2018 | Excluded | Irrelevant, did not discuss the subject matter |
| 1331 | Same-day ART initiation in HIV/STI testing center in Bangkok, Thailand: Initial results from an implementation research | Seekaew, P.; Teeratakulpisarn, N.; Surapuchong, P.; Teeratakulpisarn, S.; Amatavete, S.; Jomja, P.; Prabjunteuk, C.; Hanaree, C.; Chaison, W.; Plodgratoke, P.; Singhaseni, K.; Lingjongrat, D.; Janyam, S.; Na Nakorn, P.; Charoenying, S.; Mills, S.; Vannakit, R.; Phanuphak, P.; Phanuphak, N. | 2018 | Excluded | Irrelevant, did not discuss the subject matter |
| 1332 | Mortality trends among HIV infected patients at Newlands Clinic in Harare, Zimbabwe | Shamu, T.; Chimbetete, C.; Bote, S.; Mudzviti, T.; Luethy, R. | 2018 | Excluded | Irrelevant, did not discuss the subject matter |
| 1333 | Xpert MTB/Rif Ultra for earlier diagnosis of TB meningitis in HIV-positive adults | Cresswell, F.; Bahr, N.; Bangdiwala, A.; Akampuria, A.; Ssemambulidde, K.; Rhein, J.; Williams, D.; Kwizera, R.; Nuwagira, E.; Orikiriza, P.; Muzoora, C.; Meya, D.; Boulware, D.; Elliott, A. | 2018 | Excluded | Irrelevant, did not discuss the subject matter |
| 1334 | Evaluation of a national cryptococcal antigen screening program for HIV-infected patients in Uganda: A costeffectiveness modeling analysis | Rajasingham, R.; Meya, D.; Greene, G.; Jordan, A.; Chiller, T.; Boulware, D.; Larson, B. | 2018 | Excluded | Irrelevant, did not discuss the subject matter |
| 1335 | HIV-associated central nervous system infections in Indonesia: A cohort study examining etiology, presentation and outcome | Imran, D.; Estiasari, R.; Maharani, K.; Sucipto, S.; Lestari, D.C.; Eddy, R.; Yunihastuti, E.; Harjono, T.; Oei, D.; Timan, I.S.; Wulandari, D.; Wahyuningsih, R.; Bahri, A.; Kurniawan, A.; Mulyadi, R.; Karuniawati, A.; Jaya, U.A.; Safari, D.; Van Laarhoven, A.; Alisjahbana, B.; Dian, S.; Chaidir, L.; Ganiem, A.R.; Lastri, D.N.; Myint, K.S.A.; Van Crevel, R. | 2018 | Excluded | Irrelevant, did not discuss the subject matter |
| 1336 | Clinico-microbiological profile of Cryptococcal infections in non-HIV infected patients | Borde, K.R.; Marak, R.S.K.; Kaul, A.; Dixit, A.K.; Dhole, T.N. | 2018 | Excluded | Irrelevant, did not discuss the subject matter |
| 1337 | Detection of cryptococcal antigen in HIV positive patients using the crag lateral flow assay in Jos, Plateau State, Nigeria | Nnadi, I.; Vendagor, N.; Enweani, I.; Ayanbimpe, G. | 2018 | Excluded | Irrelevant, did not discuss the subject matter |
| 1338 | Epidemiology and outcome of IFD in pediatric patients: The Southern hemisphere | Carlesse, F. | 2018 | Excluded | Irrelevant, did not discuss the subject matter |
| 1339 | Molecular epidemiology of Cryptococcus in South Africa | Naicker, S.D. | 2018 | Excluded | Irrelevant, did not discuss the subject matter |
| 1340 | Molecular epidemiology of Cryptococcus neoformans and Cryptococcus gattii in Latin America | Firacative, C. | 2018 | Excluded | Irrelevant, did not discuss the subject matter |
| 1341 | Burden of serious fungal diseases in republic of Moldova | Burduniuc, O.; Mares, M.; Denning, D.W. | 2018 | Excluded | Irrelevant, did not discuss the subject matter |
| 1342 | Cryptococcosis in a regional HIV/AIDS reference service in southern Brazil | Poester, V.R.; Bock, D.; Sanchotene, K.O.; Klafke, G.B.; Xavier, M.O. | 2018 | Excluded | Irrelevant, did not discuss the subject matter |
| 1343 | Neglected diseases in Nigerians, diagnostics challenges: Invasive Fungal Infections | Oladele, R.O.; Richardson, M.; Ogunsola, F.T.; Denning, D.W. | 2018 | Excluded | Irrelevant, did not discuss the subject matter |
| 1344 | Involuntary movements associated with encefalitis autoimmune antinmda-R: Clinical case report | Mendoza, M.H.; Huertas, M. | 2018 | Excluded | Irrelevant, did not discuss the subject matter |
| 1345 | Cryptococcal meningoencephalitis in sarcoidosis patient associated with positive CSF anti-NMDA receptor antibodies-a therapeutic challenge | Calejo, M.; Samoes, R.; Valdoleiros, S.; Dias, D.C.; Lopes, J.M.C.F.; Marinho, A.; GonÃ§alves, M.J.; Vasconcelos, O.; Silva, A.M.D. | 2018 | Excluded | Irrelevant, did not discuss the subject matter |
| 1346 | Abstracts from the 2018 Conference on Retroviruses and Opportunistic Infections | | 2018 | Excluded | Irrelevant, did not discuss the subject matter |
| 1347 | Adjunctive sertraline in HIV-associated cryptococcal meningitis | Rhein, J.; Hullsiek, K.H.; Tugume, L.; Nuwagira, E.; Mpoza, E.; Kiggundu, R.; Ssebambulidde, K.; Williams, D.A.; Bangdiwala, A.; Abassi, M.; Musubire, A.; Muzoora, C.; Meya, D.; Boulware, D.R. | 2018 | Excluded | Irrelevant, did not discuss the subject matter |
| 1348 | Diagnostic utility of multiplex PCR in an HIV-infected population with meningitis | Bridge, S.; Hullsiek, K.H.; Kwizera, R.; Nuwagira, E.; Stadelman, A.; Kiiza, T.K.; Ndyatunga, L.; Ssebambulidde, K.; Mwesigye, J.; Mpoza, E.; Abassi, M.; Meya, D.; Muzoora, C.; Boulware, D.R.; Rhein, J. | 2018 | Excluded | Irrelevant, did not discuss the subject matter |
| 1349 | CrAg status and effect on benefits from enhanced prophylaxis in the reality trial | Pett, S.; Haddow, L.; Nhema, R.; Spyer, M.J.; Benjamin, L.; Najjuka, G.; Daud, I.; Berkley, J.; Kitabalwa, J.; Hakim, J.G.; Heyderman, R.; Walker, S.; Gibb, D. | 2018 | Excluded | Irrelevant, did not discuss the subject matter |
| 1350 | Evaluation of a cryptococcal screening and treatment program in HIV clinics in Uganda | Nalintya, E.; Meya, D.; Lofgren, S.; Huppler Hullsiek, K.; Boulware, D.R.; Rajasingham, R. | 2018 | Excluded | Irrelevant, did not discuss the subject matter |
| 1351 | Clinic-based lateral flow cryptococcal antigen testing at HIV diagnosis, South Africa | Drain, P.K.; Hong, T.; Thulare, H.; Moosa, M.-Y.; Celum, C.L. | 2018 | Excluded | Irrelevant, did not discuss the subject matter |
| 1352 | Symptomatic cryptococcal antigenemia presenting as early cryptococcal meningitis | Ssebambulidde, K.; Abassi, M.; Rhein, J.; Bangdiwala, A.; Williams, D.A.; Mpoza, E.; Tugume, L.; Kwizera, R.; Musubire, A.; Cresswell, F.; Lofgren, S.; Hullsiek, K.H.; Boulware, D.R.; Meya, D. | 2018 | Excluded | Irrelevant, did not discuss the subject matter |
| 1353 | Declining incidence of HIV-associated cryptococcosis in South Africa, 2005-2015 | Govender, N.P.; Cohen, C.; Matlapeng, P.; Crowther-Gibson, P.; Meiring, S.; Karstaedt, A.; Dawood, H.; McCarthy, K.; Meintjes, G.; Quan, V. | 2018 | Excluded | Irrelevant, did not discuss the subject matter |
| 1354 | Etiologies of symptomatic recurrence of HIV-associated cryptococcal meningitis | Evans, E.; Hullsiek, K.H.; Nuwagira, E.; Mpoza, E.; Ssebambulidde, K.; Tugume, L.; Morawski, B.M.; Lofgren, S.; Abassi, M.; Williams, D.A.; Muzoora, C.; Meya, D.; Boulware, D.R.; Rhein, J. | 2018 | Excluded | Irrelevant, did not discuss the subject matter |
| 1355 | High mortality associated with unmasking cryptococcal meningitis | Rhein, J.; Hullsiek, K.H.; Bahr, N.C.; Tugume, L.; Nuwagira, E.; Ssebambulidde, K.; Kiggundu, R.; Evans, E.; Mpoza, E.; Williams, D.A.; Abassi, M.; Musubire, A.; Muzoora, C.; Meya, D.; Boulware, D.R. | 2018 | Excluded | Irrelevant, did not discuss the subject matter |
| 1356 | Customized amphotericin duration for cryptococcal meningitis based on fungal burden | Williams, D.A.; Hullsiek, K.H.; Akampurira, A.; Luggya, T.; Nuwagira, E.; Tugume, L.; Mpoza, E.; Kiggundu, R.; Ssebambulidde, K.; Abassi, M.; Muzoora, C.; Meya, D.; Boulware, D.R.; Rhein, J. | 2018 | Excluded | Irrelevant, did not discuss the subject matter |
| 1357 | Predictors of mortality in adults on treatment for human immunodeficiency virus-associated tuberculosis in Botswana: A retrospective cohort study | Muyaya, L.M.; Young, T.; Loveday, M. | 2018 | Excluded | Irrelevant, did not discuss the subject matter |
| 1358 | The Continuing Burden of Advanced HIV Disease over 10 Years of Increasing Antiretroviral Therapy Coverage in South Africa | Osler, M.; Hilderbrand, K.; Goemaere, E.; Ford, N.; Smith, M.; Meintjes, G.; Kruger, J.; Govender, N.P.; Boulle, A. | 2018 | Excluded | Irrelevant, did not discuss the subject matter |
[truncated: 340,684 more chars]
