# Supplementary material for: Systematic review on the compliance of WHO guidelines in the management of patients with advanced HIV disease in Africa: The case of cryptococcal antigen screening
Source: PLoS One. 2025 Jan 24;20(1):e0313453. doi: 10.1371/journal.pone.0313453 (PMC11761098; doi:10.1371/journal.pone.0313453)
Supplement: S6 Table — (DOCX) [file pone.0313453.s006.docx]

| **S6 Table: Overall quality of systematic review using GRADE system framework** | | | | | |
| --- | --- | --- | --- | --- | --- |
| Outcome | Risk of bias | Consistency | Directness | Precision | Risk of publication bias |
| Uptake of CrAg screening | Low | Low | High | High | Low |
| CrAg antigenemia | Low | Low | High | High | Low |
| Preemptive Antifungal initiations | Low | Low | High | High | Low |
| Lumbar puncture | Low | Low | High | High | Low |
| Abbreviations: CrAg indicates Cryptococcal antigen | | | | | |
