# Supplementary material for: Systematic review on the compliance of WHO guidelines in the management of patients with advanced HIV disease in Africa: The case of cryptococcal antigen screening
Source: PLoS One. 2025 Jan 24;20(1):e0313453. doi: 10.1371/journal.pone.0313453 (PMC11761098; doi:10.1371/journal.pone.0313453)
Supplement: Attachment — Submitted filename: PONE-D-24-28898_reviewer_ggreene.pdf [file pone.0313453.s007.pdf]

PLOS ONE

Systematic review on the compliance of WHO guidelines in the management of patients with advanced HIV disease in low- and middle-income countries: The case of cryptococcal antigen screening.

--Manuscript Draft--

Manuscript Number:	PONE-D-24-28898
Article Type:	Research Article
Full Title:	Systematic review on the compliance of WHO guidelines in the management of patients with advanced HIV disease in low- and middle-income countries: The case of cryptococcal antigen screening.
Short Title:	Uptake of cryptococcal antigen screening
Corresponding Author:	Zuhura Mbwana Ally<br>District Hospital Tanga<br>Tanga, TANZANIA, UNITED REPUBLIC OF
Keywords:	Cryptococcal antigen screening, Advanced HIV disease, People living HIV, low- and mid-income countries.
Abstract:	Background.<br>The World Health Organization (WHO) recommended cryptococcal antigen (CrAG) screening for people presenting with advanced HIV disease (AHD) and for those with positive CrAG to initiate preemptive antifungal medication. Data on WHO recommendations about CrAG screening implementation is limited. We estimated pooled prevalence of CrAG screening uptake, cryptococcal antigenemia, and initiation of preemptive antifungal medication in low- and mid-income countries.<br>Methods.<br>In this systematic-review PubMed, Cochrane Library and Embase were searched for articles published between January 2005 and December 2023. CrAG uptake was defined as percentage of eligible people (CD4<200cells/mm3 or WHO stage III/IV) who received cryptococcal antigen testing. Stratified analysis to compare uptake and cryptococcal antigenemia between ART-naïve and ART-experienced individuals and those with CD4<100 vs 100-199cells/mm3 was performed. Using random effects models, we computed the pooled prevalence of CrAG screening uptake, cryptococcal antigenemia, and preemptive antifungals treatment and 95% confidence intervals (CIs).<br>Results.<br>Sixteen studies with 26,132 individuals with AHD were analyzed. Overall, the pooled prevalence of CrAG screening uptake was 62.9% (95%CI:46.2-77.0). CrAG screening uptake was significantly higher among ART-naïve compared to ART-experienced individuals, (64.0% vs 42.1%; p<0.001) and higher among those with CD4 <100cells/mm3 compared to those with CD4 100-199 (58.1% vs 44.2%; p<0.001). Overall, the pooled prevalence of cryptococcal antigenemia was 8.1% (95%CI:7.1-11.6). Cryptococcal antigenemia was significantly higher among ART-experienced compared to ART-naïve individuals, (12.6% vs 8.5%; p<0.001) and higher among those with CD4 <100cells/mm3 compared to those with CD4 100-199cells/mm3 (8.1% vs 2.6%; p<0.001). The overall prevalence of preemptive antifungal treatment was 83.5% (95%CI:65.9-96.0).<br>Conclusions.<br>Not screening for CrAG among people with AHD and failure to initiate antifungal medications among those with cryptococcal antigenemia presents a significant missed opportunity. Emphasis on improving CrAG screening is critical given its proven cost-effective benefits.
Order of Authors:	Zuhura Mbwana Ally
	Jackline Vicent Mbishi
	Mariam Salim Mbwana
	Hafidha Mhando Bakari

Powered by Editorial Manager® and ProduXion Manager® from Aries Systems Corporation

	Swalehe Mustafa Salim
	Zarin Nudar Rodoshi
	Muhidin Ibrahim Hundisa
	Rebecca Mesfin Sileshi
	Biruk D. Ayalew
	Rahma Musoke
	Lynn Moshi
	Yousef Elias Fakhoury
	Habib Omari Ramadhani
**Additional Information:**	
**Question**	**Response**
**Financial Disclosure**<br><br>Enter a financial disclosure statement that describes the sources of funding for the work included in this submission. Review the submission guidelines for detailed requirements. View published research articles from *PLOS ONE* for specific examples.<br><br>This statement is required for submission and **will appear in the published article** if the submission is accepted. Please make sure it is accurate.<br><br>**Funded studies**<br>Enter a statement with the following details:<br>• Initials of the authors who received each award<br>• Grant numbers awarded to each author<br>• The full name of each funder<br>• URL of each funder website<br>• Did the sponsors or funders play any role in the study design, data collection and analysis, decision to publish, or preparation of the manuscript?<br><br>Did you receive funding for this work?	The author(s) received no specific funding for this work.
**Competing Interests**<br><br>Use the instructions below to enter a competing interest statement for this submission. On behalf of all authors, disclose any competing interests that	The authors have declared that no competing interests exist.

could be perceived to bias this work—acknowledging all financial support and any other relevant financial or non-financial competing interests.<br><br>This statement is required for submission and **will appear in the published article** if the submission is accepted. Please make sure it is accurate and that any funding sources listed in your Funding Information later in the submission form are also declared in your Financial Disclosure statement.<br><br>View published research articles from *PLOS ONE* for specific examples.<br><br>**NO authors have competing interests**<br><br>Enter: *The authors have declared that no competing interests exist.*<br><br>**Authors with competing interests**<br><br>Enter competing interest details beginning with this statement:<br><br>*I have read the journal's policy and the authors of this manuscript have the following competing interests: [insert competing interests here]*<br><br>* typeset	
**Ethics Statement**<br><br>Enter an ethics statement for this submission. This statement is required if the study involved:<br><br>• Human participants<br>• Human specimens or tissue<br>• Vertebrate animals or cephalopods<br>• Vertebrate embryos or tissues<br>• Field research<br><br>Write "N/A" if the submission does not require an ethics statement.	This was a systematic review of the published manuscripts and therefore ethics approval was not required

General guidance is provided below. Consult the submission guidelines for detailed instructions. **Make sure that all information entered here is included in the Methods section of the manuscript.**<br><br>**Format for specific study types**<br><br>**Human Subject Research (involving human participants and/or tissue)**<br>• Give the name of the institutional review board or ethics committee that approved the study<br>• Include the approval number and/or a statement indicating approval of this research<br>• Indicate the form of consent obtained (written/oral) or the reason that consent was not obtained (e.g. the data were analyzed anonymously)<br><br>**Animal Research (involving vertebrate animals, embryos or tissues)**<br>• Provide the name of the Institutional Animal Care and Use Committee (IACUC) or other relevant ethics board that reviewed the study protocol, and indicate whether they approved this research or granted a formal waiver of ethical approval<br>• Include an approval number if one was obtained<br>• If the study involved *non-human primates*, add *additional details* about animal welfare and steps taken to ameliorate suffering<br>• If anesthesia, euthanasia, or any kind of animal sacrifice is part of the study, include briefly which substances and/or methods were applied<br><br>**Field Research**<br><br>Include the following details if this study involves the collection of plant, animal, or other materials from a natural setting:<br>• Field permit number<br>• Name of the institution or relevant body that granted permission	
**Data Availability**<br><br>Authors are required to make all data	Yes - all data are fully available without restriction

Powered by Editorial Manager® and ProduXion Manager® from Aries Systems Corporation

underlying the findings described fully available, without restriction, and from the time of publication. PLOS allows rare exceptions to address legal and ethical concerns. See the PLOS Data Policy and FAQ for detailed information.<br><br>A Data Availability Statement describing where the data can be found is required at submission. Your answers to this question constitute the Data Availability Statement and **will be published in the article**, if accepted.<br><br>**Important:** Stating 'data available on request from the author' is not sufficient. If your data are only available upon request, select 'No' for the first question and explain your exceptional situation in the text box.<br><br>Do the authors confirm that all data underlying the findings described in their manuscript are fully available without restriction?	
**Describe where the data may be found in full sentences. If you are copying our sample text, replace any instances of XXX with the appropriate details.**<br><br>• If the data are **held or will be held in a public repository**, include URLs, accession numbers or DOIs. If this information will only be available after acceptance, indicate this by ticking the box below. For example: *All XXX files are available from the XXX database (accession number(s) XXX, XXX.*).<br>• If the data are all contained **within the manuscript and/or Supporting Information files**, enter the following: *All relevant data are within the manuscript and its Supporting Information files.*<br>• If neither of these applies but you are able to provide **details of access elsewhere**, with or without limitations, please do so. For example:<br><br>*Data cannot be shared publicly because of [XXX]. Data are available from the*	All relevant data are within the manuscript and its Supporting Information files.

Powered by Editorial Manager® and ProduXion Manager® from Aries Systems Corporation

XXX Institutional Data Access / Ethics Committee (contact via XXX) for researchers who meet the criteria for access to confidential data.<br><br>*The data underlying the results presented in the study are available from (include the name of the third party and contact information or URL).*<br>• This text is appropriate if the data are owned by a third party and authors do not have permission to share the data.<br><br>* typeset	
Additional data availability information:	

Powered by Editorial Manager® and ProduXion Manager® from Aries Systems Corporation

Manuscript

Click here to access/download;Manuscript;Manuscript_12JUL2024.docx

Systematic review on the compliance of WHO guidelines in the management of patients with advanced HIV disease in low- and middle-income countries: The case of cryptococcal antigen screening.

Zuhura Mbwana Ally[1], Jackline Vicent Mbishi[2], Mariam Salim Mbwana[3], Hafidha Mhando Bakari[4], Swalehe Mustafa Salim[5], Zarin Nudar Rodoshi[6], Muhidin Ibrahim Hundisa[7],Rebecca Mesfin Sileshi[8], Biruk D. Ayalew[8], Rahma Musoke[9], Lynn Moshi[10], Yousef Elias Fakhoury[11] Habib Omari Ramadhani[12]

Affiliations.

1. District Hospital, Tanga, Tanzania.
2. Muhimbili University of Health and Allied Sciences, Dar es salaam, Tanzania.
3. Primary Health Care Institute, Iringa, Tanzania.
4. University of Dar es salaam, Dar es salaam, Tanzania.
5. Canada Youth Group, Dar es salaam, Tanzania.
6. Mymensingh Medical College & Hospital, Mymensingh, Bangladesh.
7. Ministry of Health, Addis Ababa, Ethiopia.
8. St. Paul's Hospital Millennium Medical College, Addis Ababa, Ethiopia.
9. Water mission, Dar es salaam, Tanzania.
10. Aga Khan Hospital, Dar es salaam, Tanzania.
11. Al Hussain New Salt Hospital, Jordan.
12. Kilimanjaro Christian Medical Center, Tanzania, Kilimanjaro, Tanzania.

Corresponding author: Zuhura Mbwana Ally- District Hospital Tanga, Tanzania, zuhuraally86@gmail.com

Short title: Uptake of cryptococcal antigen screening

Abstract.

Background.

The World Health Organization (WHO) recommended cryptococcal antigen (CrAG) screening for people presenting with advanced HIV disease (AHD) and for those with positive CrAG to initiate preemptive antifungal medication. Data on WHO recommendations about CrAG screening implementation is limited. We estimated pooled prevalence of CrAG screening uptake, cryptococcal antigenemia, and initiation of preemptive antifungal medication in low- and mid-income countries.

Methods.

In this systematic-review PubMed, Cochrane Library and Embase were searched for articles published between January 2005 and December 2023. CrAG uptake was defined as percentage of eligible people (CD4<200cells/mm^3 or WHO stage III/IV) who received cryptococcal antigen testing. Stratified analysis to compare uptake and cryptococcal antigenemia between ART-naïve and ART-experienced individuals and those with CD4<100 vs 100-199cells/mm^3 was performed. Using random effects models, we computed the pooled prevalence of CrAG screening uptake, cryptococcal antigenemia, and preemptive antifungals treatment and 95% confidence intervals (CIs).

Results.

Sixteen studies with 26,132 individuals with AHD were analyzed. Overall, the pooled prevalence of CrAG screening uptake was 62.9% (95%CI:46.2-77.0). CrAG screening uptake was significantly higher among ART-naïve compared to ART-experienced individuals, (64.0% vs 42.1%; p<0.001) and higher among those with CD4 <100cells/mm^3 compared to those with CD4

100-199 (58.1% vs 44.2%; p<0.001). Overall, the pooled prevalence of cryptococcal antigenemia was 8.1% (95%CI:7.1-11.6). Cryptococcal antigenemia was significantly higher among ART-experienced compared to ART-naïve individuals, (12.6% vs 8.5%; p<0.001) and higher among those with CD4 <100cells/mm^3 compared to those with CD4 100-199cells/mm^3 (8.1% vs 2.6%; p<0.001). The overall prevalence of preemptive antifungal treatment was 83.5% (95%CI:65.9-96.0).

Conclusions.

Not screening for CrAG among people with AHD and failure to initiate antifungal medications among those with cryptococcal antigenemia presents a significant missed opportunity. Emphasis on improving CrAG screening is critical given its proven cost-effective benefits.

Keywords: Cryptococcal antigen screening, Advanced HIV disease, People living HIV, low- and mid-income countries.

Introduction

Cryptococcal meningitis is the second most common cause of HIV related deaths after tuberculosis[1]. Nearly 15% of HIV related deaths are due to cryptococcal meningitis globally. A systematic review conducted in Africa showed that cryptococcal meningitis accounted for 44% of mortality among people living with HIV (PLWH) [2]. Annually, 223,100 cases of cryptococcal meningitis are estimated to occur among PLWH with CD4 count <100cells/mm^3 globally [3]. Low- and mid-income countries (LMICs) are the most hit by cryptococcal meningitis even during this period of an increased access to antiretroviral therapy (ART) . Of these estimated annual incident cases of cryptococcal meningitis, 73% occurred in Africa and 19% in Asia and Pacific [3]. ART naïve people living with HIV who present with baseline advance HIV disease (AHD) defined by the World Health Organization (WHO) (CD4 count < 200cells/mm^3 or WHO stage III/IV) are at higher risk of developing cryptococcal meningitis[4, 5].

Prior data indicated that screening for cryptococcal meningitis is cost effective as it averts up to 43% of deaths related to this infection among PLWH presenting with AHD[6, 7]. Provision of antifungal medications such as fluconazole among PLWH screened positive for cryptococcal antigen (CrAG) prevents the development of cryptococcal meningitis and deaths[8]. One meta-analysis showed that, the incidence of cryptococcal meningitis among CrAG positive patients who received antifungals was 3% compared to 5% among those who did not receive antifungals, equivalent to 40% reduction in the development of cryptococcal meningitis[9]. Due to these notable benefits, the WHO recommended regular screening for cryptococcal disease among PLWH presenting with AHD and for those with detectable CrAG to receive preemptive antifungal medications[10].

Several studies have evaluated the prevalence of positive CrAG among ART naïve PLWH presenting with AHD[11-13] and a few also explored CrAG positivity among ART experienced PLHIV[14, 15]. While understanding the magnitude of CrAG positivity is critical, there is limited data on the uptake of CrAG implementation screening following WHO recommendations. Reported CrAG screening uptake is as low as 19% in Uganda to as high as 89% in Malawi [7, 16]. Supplies stock out, dysfunctional CD4 count instruments, lack of training and unawareness of the WHO guidelines are some of the identified reasons for the low uptake of CrAG screening as reported by prior studies[17-19]. Among patients who tested positive for CrAG, 54% to 100% are documented to have initiated preemptive antifungal medications[20, 21]. In this systematic review and meta-analysis, we estimated pooled prevalence of CrAG screening uptake, cryptococcal antigenemia and initiation of preemptive antifungal medication in LMICs.

Methods.

Study protocol and registration.

This systematic review has been registered in the International Prospective Registry of Systematic Review with registration number CRD42023494535. This systematic review and meta-analysis was reported according to the Preferred Reporting Items for Systematic Reviews and Meta-Analyses (PRISMA) guidelines[22].

Ethical approval.

Because this was a systematic review of published manuscripts, ethical approval was not sought.

Search strategy.

PubMed, Cochrane CENTRAL, Embase and clinicaltrials.gov were searched by a medical librarian (EL) for the articles published between January 2005 and December 2023. Search terms were used to capture information on uptake of cryptococcal meningitis screening among PLWH who presented with advanced HIV disease in LMICs. A full search strategy can be found in supplemental table 1. The search was restricted to papers written in English. Search results were uploaded to Covidence Systematic Review Software (Melbourne, Australia) for deduplication and screening.

Eligibility criteria.

The eligibility criteria were structured using the PICOS (ie, population, intervention, comparison, outcome, and study type) framework[23] as follows: People with advanced HIV disease as the population; ART naïve or CD4 < 100 cells/mm^3 as the intervention/exposed groups; ART experienced or CD4 100-199 cells/mm^3 as comparison groups; CrAG screening uptake, cryptococcal antigenemia, and preemptive antifungals initiations as outcome; and observational studies as study design

Inclusion and exclusion criteria.

Observational studies conducted in LMICs, reported proportions of PLWH eligible for cryptococcal meningitis screening who received CrAG screening test and/or proportions of eligible persons who received preemptive antifungal medications and written in English were eligible for inclusion. We excluded studies that did not report the uptake CrAG screening.

Study selection and data extraction.

The manuscripts searched from outlined databases were managed by Covidence software from which the final list of manuscripts was deduplicated. Two pairs of review authors (ZMA/HRO and ZNR/BDA) independently completed the study selection for inclusion in the appraisal process. Disagreement between two independent pairs of reviewers for the inclusion of the manuscripts was handled by the third pair of reviewers (SMS/MIH). Using a pre specified Excel spread sheet template, the two pairs of review authors (ZMA/HRO and ZNR/BDA) independently extracted the following data elements from the included studies: authors, year of publication, the country in which the study was conducted, study design, study period, study population (ART naïve vs ART experienced), CrAG eligibility criteria (CD4 < 100 vs CD4 < 200), number of people eligible for CrAG screening, number of people who screened for CrAG, number of people who were positive for CrAG, number of people who received preemptive antifungal medications, and study sample size.

Risk bias assessment.

The National Institute for Health (NIH) tool was used to assess the quality studies.[24] Reliability and validity of the measurement tools, participation rate, source of study participant recruitment, justification of sample size or power calculations and appropriateness of statistical analysis were evaluated for all studies that met inclusion criteria. Finally, percentage scores reflecting the quality of the studies were computed based on the stated parameters.

Definition of variables.

Exposure and outcome.

The main outcome of interest is the uptake of CrAG screening defined as percentage of eligible PLWH (CD4 < 100cells/mm^3 or <200 cells/mm^3) who received CrAG screening test. Other secondary outcomes included prevalence of positive CrAG , and preemptive antifungal medications initiations. Uptake of CrAG screening, CrAG positivity and proportions of people who received preemptive antifungal medications were further stratified by the type of study population (ART naïve vs ART experienced) and the level of CD4 count (<100 cells/mm^3 vs 100-199 cells/mm^3).

Data synthesis and analysis.

Meta regression for proportions using standard random effects models were used to compute pooled prevalence of CrAG uptake, CrAG positivity and receipt of preemptive antifungal medications. The variance of proportions were stabilized using Freeman-Tukey double arcsine transformation before pooling[25, 26]. Subgroup analysis on the pooled prevalences of these outcomes were performed based on the type of study population (ART naïve vs ART experienced) and the level of CD4 count (<100 cells/mm3 vs 100-199 cells/mm3). Chi square tests were used to compare pooled prevalences of the stratified analysis. We evaluated heterogeneity across studies using the I^2 statistic, which represents the percentage of total variability due to between-study heterogeneity. The I^2 statistic was categorized at values of 25%, 50% and 75% to represent low, moderate, and high extent of heterogeneity respectively as previously described[27].The publication bias was assessed using the Egger regression asymmetry test. For both heterogeneity and publication test, a p value < 0.05 indicated the presence of heterogeneity and publication bias respectively. To explore the source of heterogeneity, an influential analysis using the leave-one-out method was performed. Studies with missing information, such as those that reported proportions of outcomes without actual

numerators and/or denominators, were excluded from the analysis. All statistical tests were performed using STATA version 17 (Stata Corporation, College Station, Texas, USA) and R programming version 4.4.0.

Results.

A total of 2,688 publications were retrieved through searches and 844 were duplicates and removed, leaving 1,844 publications to have their titles and abstracts screened and 1,826 were excluded as they did not address the intended study matter. The remaining 18 received a full review and 16 were eligible for final analysis (Fig 1).

Fig 1. PRISMA flow diagram of the included studies for meta-analysis of on the compliance of WHO guidelines in the management of patients with advanced HIV disease in low- and middle-income countries.

Study selection.

Characteristics of studies included.

Study articles included ART naïve participants (n = 9)[11-14, 17, 19-21] , ART experienced (n = 2)[14, 15] and both ART naïve and ART experienced (n = 5)[16, 28-31].Sample size for studies ranged from 47 to 7,210. A total of 26,132 PLWH who had AHD were included in this analysis (Table 1). We included studies from Africa (n = 15), and Asia (n = 1).

Table 1: Summary of characteristics of the included studies

Author	Study period	Country	CrAG criteria	Type of population	Eligible for CrAG screening	CrAG screening uptake	Prevalence of positive CrAG	Preemptive antifungal initiations	Quality scores %
Baluku et al, 2021	2015–2018	Uganda	CD4 < 100	Naïve	937	95.1	13.8	NR	86
Baluku et al, 2021	2015–2018	Uganda	CD4 < 100	Experienced	7,210	11.5	10.5	NR	86
Enock et al, 2022	2015–2017	Uganda	CD4 < 100	Naïve	359	71.0	22.0	83.9	92
Huang et al, 2021	2009–2018	Taiwan	CD4 < 200	Naïve	1,858	59.4	6.3	NR	86
Mbewe et al, 2022	2018–2019	Zambia	CD4 < 200	Experienced	47	80.9	18.4	100.0	86
Tiam et al, 2023	2018–2019	Lesotho	CD4 < 100	Naïve	109	35.8	12.8	NR	71
Blankley et al, 2019	2015–2016	Zimbabwe	CD4 < 100	Naïve	377	83.0	8.0	NR	85
Vallabhaneni et al, 2016	2012–2013	South Africa	CD4 < 100	Naïve	4,395	26.6	2.1	54.2	71
Drain et al, 2021	2013–2019	South Africa	CD4 < 200	Naïve	908	33.1	9.3	100.0	71
Heller et al, 2022	2017	Malawi	CD4 < 100	Naïve	230	80.4	10.8	NR	92
Meyer et al, 2013	2009	Kenya	CD4 < 100	Naïve	782	65.7	11.5	86.4	85
Heller et al, 2022	2017–2020	Malawi	CD4 < 200	Both	475	89.1	13.9	NR	92
Sing'oei et al, 2017	2013-2017	Multi country*	CD4 < 200	Both	494	80.4	3.8	NR	86
Kanyama et al,2022	2016-2017	Malawi	CD4 < 200	Both	221	81.0	10.6	NR	89
Longley et al, 2016	2012-2013	South Africa	CD4 < 100	Both	4,395	26.6	2.1	54.2	86
Hurt et al, 2021	2014-2016	Botswana	CD4 < 100	Both	3,335	49.3	6.3	NR	85

Abbreviations: CrAG indicates cryptococcal antigen; NR indicates not reported; * indicates Uganda, Kenya, Tanzania, and Nigeria.

Risk of bias assessment.

Quality assessment showed that, all studies included were of high quality with median scores of 86% [interquartile range (85–88)]. Failure to provide justification of the sample size and/or power calculations was the most common methodological weakness.

Prevalence of CrAG screening uptake.

The overall, pooled prevalence of CrAG screening uptake was 62.9% (95% CI 46.2-77.0) (Fig 2). Table 2 describes results of stratified analysis on the prevalence of CrAG screening uptake and cryptococcal antigenemia. Pooled prevalence of CrAG screening uptake was significantly higher among ART naïve 64.0%(95% CI 42.3-81.1) compared to 42.1%% (95% CI 0.0-99.5) among ART experienced, and higher among people who had CD4 count < 100 cells/mm^3 58.1%(95% CI 34.6-78.4) compared to 44.2%(95% CI 40.5-48.0) among those who had CD4 of 100-199 cells/mm^3.

Fig 2. Overall prevalence of CrAG screening uptake among people living with HIV in low- and mid-income countries.

Assessment of heterogeneity, publication bias, and influential analysis on the prevalence of retesting uptake.

The I^2 statistic test of heterogeneity showed a value of 100% (p=0.0) on the overall prevalence of CrAG screening uptake, indicating high heterogeneity.(Table 2). Egger's test showed no statistically significant small-study effect (z-test = 1.13, p=0.258), indicating absence of publication bias. Estimates from the leave-one-out forest plot showed a non-significant influence of all studies on the prevalence of CrAG retesting uptake (Fig 3).

Table 2: Prevalence of cryptococcal meningitis screening uptake, cryptococcal antigenemia and preemptive antifungal medication initiations among people living with HIV in low- and mid-income countries.

Characteristic	# of studies	n	Prevalence (95%CI)	I^2%	p-value	Egger's test (z)	p-value
CrAG screening uptake							
Overall	17	26,132	62.9 (46.2 – 77.0)	100	0.0	1.13	0.258
ART naïve	10	9,995	64.0 (42.3 – 81.1)	99	0.0	9;16	<0.001
ART experienced	2	7,257	42.1 (0.0 – 99.5)	99	0.0	1.89	0.058
Both ART naïve and experienced	5	8,920	68.5 (31.0 – 91.3)	100	0.0	8.03	<0.001
CD4 < 100	11	23,279	58.1 (34.6 – 78.4)	100	0.0	5.53	<0.001
CD4 < 100-199	1	708	44.2 (40.5 – 48.0)	-	-	23.67	<0.001
CD4 < 200	5	2,145	75.5 (44.7 – 92.2)	99	0.0	5.79	<0.001
CrAG antigenemia							
Overall	17	9,454	8.1 (5.6 – 11.7)	94	0.0	3.35	0.001
ART naïve	10	4,772	8.5 (5.0 – 14.1)	94	0.0	5.38	<0.001
ART experienced	2	868	12.6 (0.3 – 87.3)	56	0.1	3.82	<0.001
Both ART naïve and experienced	5	3,814	6.1 (2.3 – 15.2)	95	0.0	4.09	<0.001
CD4 < 100	11	7,803	8.1 (4.9 – 13.2)	95	0.0	6.61	<0.001
CD4 < 100-199	1	313	2.6 (1.1 – 5.0)	-	-	2.87	0.004
CD4 < 200	5	1,338	9.9 (4.7 – 19.5)	84	0.0	4.07	<0.001
Preemptive antifungal initiation							
Overall	6	159	83.5 (65.9 – 96.0)	85.6	0.0	0.56	0.574

Abbreviations: n, indicates number; CI, indicates confidence intervals; CrAG indicates cryptococcal antigen

Fig 3. Influential analysis on the overall prevalence of CrAG screening uptake among people living with HIV in low- and mid-income countries.

Prevalence of CrAG antigenemia.

Seven hundred and twenty-five people with AHD had positive CrAG, translating to an overall pooled prevalence of 8.1% (95% CI 5.6-11.6) (Fig 4). The prevalence of positive CrAG was 8.5% (95% CI 5.0-14.1) among ART naïve and 12.6% (95% CI 0.3-87.3) among ART experienced. Compared to people who had CD4 < 100 cells/mm^3, those who had CD4 of 100-199 cells/mm^3 had significantly lower prevalence of positive CrAG [2.6% (95% CI 1.1-50.0) vs 8.1% (95% CI 4.9-13.2)]. (Table 2)

Fig 4. Overall prevalence of cryptococcal antigenemia among people living with HIV in low- and mid-income countries.

Assessment of heterogeneity, publication bias, and influential analysis on the prevalence of cryptococcal antigen.

There was high heterogeneity with I^2 statistic of 94% (p =0.0) on the overall prevalence of positive CrAG (Table 2). Egger's test showed statistically significant small-study effect (z-test = 3.23, p=0.001), indicating presence of publication bias. Estimates from the leave-one-out forest plot showed a non-significant influence of all studies on the prevalence of CrAG (Fig 5).

Fig 5. Influential analysis on the overall prevalence of cryptococcal antigenemia among people living with HIV in low- and mid-income countries.

Prevalence of preemptive antifungal medications initiations.

Only six studies reported preemptive antifungal medications initiations and an overall pooled prevalence was 83.5% (95% CI 65.9-96.0) (Table 2).

Discussion.

We conducted a systematic review and meta-analysis among PLWH in LMICs to evaluate pooled estimates of uptake of CrAG screening, cryptococcal antigenemia and preemptive initiation of antifungal medications. The overall pooled estimates of uptake of CrAG screening, cryptococcal antigenemia and preemptive initiation of antifungal medications in these settings were 60%, 9% and 83%, respectively. Although the uptake of CrAG screening was higher among ART naïve individuals compared to ART experienced, CrAG positivity yield was significantly higher among the latter compared to the former (12.0% vs 10.4%).

Despite of its potential benefits in reducing the development of fulminant cryptococcal meningitis and mortality associated with cryptococcal infection, compliance of the WHO guidelines in the implementation of CrAG screening among PLWH presenting with AHD is suboptimal. Previous study showed that compared to high income countries, the trajectory of cryptococcal meningitis in LMICs has not decreased despite the expansion of ART[32]. This observation may be supported by the low uptake CrAG screening among eligible individuals leading to missed opportunity to identify people at risk of developing cryptococcal meningitis. Effective screening followed by initiation of preemptive antifungal medications for those with cryptococcal antigenemia is known to reduce the burden of cryptococcal meningitis. In this analysis, although nearly 83% of those with cryptococcal antigenemia initiated antifungal medications, nearly four in ten PLWH eligible for CrAG screening did not receive screening test. Historically, CrAG screening has been low and in this era of test and treat irrespective of CD4 count levels, CD4 count testing has declined. For example, Several African countries including Cameroon, Kenya, Malawi, Namibia, Swaziland and Uganda do not recommend routine CD4 testing if viral load testing is available[33]. The declining in CD4 count testing makes even

harder in identifying PLWH eligible for CrAG screening among ART naïve as WHO staging remained to be the only criteria in identifying these individuals. Some of the reasons reported to be associated with the low uptake of CrAG includes unawareness of WHO guidelines, reagents stock out and dysfunctional CD4 count instruments[17-19]. Periodic training of healthcare workers and continuous medical education would increase awareness of WHO recommendations guidelines. Moreover, domestic investments in restocking and restoration of CD4 count machines need to be prioritized given the benefits of early identification and management of patients with cryptococcal antigenemia.

Although CrAG screening is predominantly performed among ART naïve, we have demonstrated a higher proportion of cryptococcal antigenemia among ART experienced compared to ART naïve. One study in Uganda evaluated 212 PLWH who had cryptococcal meningitis showed that, 91% were ART experienced and overall, 43% of the patients died within the hospital [34].Other studies showed nearly 50% of PLWH with cryptococcal antigenemia were ART experienced [32, 35] and many patients on ART had cryptococcal meningitis [36, 37]. These results further underscore the need for the increased CrAG screening to ART experienced meeting screening criteria. Although viral load is recommended as screening criteria among ART experienced patients, it is often routinely not available[38].

We acknowledge the presence of limitations in this meta-analysis. Majority of the studies included ART naïve patients and only two studies reported data on ART experienced, making smaller sample size among ART experienced persons. Furthermore, we restricted our search to publications written in English, it is likely that other relevant publications from non-English journals were missed. The main strength of this research is the inclusion of sixteen studies leadings to an overall large sample size to compute pooled estimates of CrAG screening uptake,

CrAG antigenemia and preemptive antifungal medication initiations. This meta-analysis remains relevant as it provides estimates of the uptake of CrAG screening to understand the magnitude of compliance of WHO recommendations in the management of PLWH presenting with AHD in LMICs.

Conclusions.

In LMICs, we identified significant gaps in CrAG screening among people with AHD and initiations of antifungal medications among people with positive CrAG. Our observation indicates compliance of WHO guidelines in the management of people with AHD is suboptimal. To reduce the risk of developing full blown cryptococcal meningitis and its associated mortality, efforts to improve screening for cryptococcal antigen and initiations of preemptive antifungal medications to those screened positive should be increased.

Author contributions

Conceptualization: Zuhura Mbwana Ally, Habib Ramadhani Omari.

Data curation: Biruk D. Ayalew, Swaleh Mustafa Salim, Muhidin Ibrahim Hundisa, Rebecca Mesfin Sileshi, Zarin Nudar Rodoshi.

Formal analysis: Jackline Vicent Mbishi, Mariam Salim Mbwana.

Investigation: Lynn Moshi, Hafidha Mhando Bakari.

Methodology: Habib Omari Ramadhani, Jackline Vicent Mbishi.

Software: Habib Omari Ramadhani, Rahma Musoke.

Validation: Mariam Salim Mbwana, Swalehe Mustafa Salim, Hafidha Mhando Bakari, Yousef Elias Fakhoury

Visualization: Rahma Musoke, Rebecca Mesfin Sileshi, Zarin Nudar Rodoshi

Writing – original draft: Zuhura Mbwana Ally.

Writing – review & editing: Habib Omari Ramadhani, Lynn Moshi**,** Yousef Elias Fakhoury.

Acknowledgements.

We acknowledge Emilie Ludeman and Taylor Lascko for database searches and linguistic review and the authors of included studies and all the clients that contributed to their research.

Funding.

There was no source of funding for this study

Competing interests

Authors declared no competing interests

Data availability

All data have been presented in the manuscript. Additional information such as analytic codes can be requested from the corresponding author.

References

1. Castelnuovo B, Manabe YC, Kiragga A, Kamya M, Easterbrook P, Kambugu A. Cause-specific mortality and the contribution of immune reconstitution inflammatory syndrome in the first 3 years after antiretroviral therapy initiation in an urban African cohort. Clin Infect Dis **2009**; 49(6): 965-72.
2. Tenforde MW, Gertz AM, Lawrence DS, et al. Mortality from HIV-associated meningitis in sub-Saharan Africa: a systematic review and meta-analysis. J Int AIDS Soc **2020**; 23(1): e25416.
3. Rajasingham R, Smith RM, Park BJ, et al. Global burden of disease of HIV-associated cryptococcal meningitis: an updated analysis. Lancet Infect Dis **2017**; 17(8): 873-81.
4. Wykowski J, Galagan SR, Govere S, et al. Cryptococcal antigenemia is associated with meningitis or death in HIV-infected adults with CD4 100-200 cells/mm(3). BMC Infect Dis **2020**; 20(1): 61.
5. Ford N, Shubber Z, Jarvis JN, et al. CD4 Cell Count Threshold for Cryptococcal Antigen Screening of HIV-Infected Individuals: A Systematic Review and Meta-analysis. Clin Infect Dis **2018**; 66(suppl_2): S152-s9.
6. Larson BA, Rockers PC, Bonawitz R, et al. Screening HIV-Infected Patients with Low CD4 Counts for Cryptococcal Antigenemia prior to Initiation of Antiretroviral Therapy: Cost Effectiveness of Alternative Screening Strategies in South Africa. PLoS One **2016**; 11(7): e0158986.
7. Rajasingham R, Meya DB, Greene GS, et al. Evaluation of a national cryptococcal antigen screening program for HIV-infected patients in Uganda: A cost-effectiveness modeling analysis. PLoS One **2019**; 14(1): e0210105.
8. Rajasingham R, Nalintya E, Israelski DM, Meya DB, Larson BA, Boulware DR. Cost-effectiveness of single-dose AmBisome pre-emptive treatment for the prevention of cryptococcal meningitis in African low and middle-income countries. Med Mycol **2022**; 60(2).
9. Li Y, Huang X, Chen H, et al. The prevalence of cryptococcal antigen (CrAg) and benefits of pre-emptive antifungal treatment among HIV-infected persons with CD4+ T-cell counts < 200 cells/µL: evidence based on a meta-analysis. BMC Infect Dis **2020**; 20(1): 410.
10. World Health Organization. Guidelines for the Diagnosis, Prevention, and Management of Cryptococcal Disease in HIV-Infected Adults, Adolescents and Children, March 2018. Geneva: World Health Organization. **2018**.
11. Huang SH, Lee CY, Tsai CS, et al. Screening for Cryptococcal Antigenemia and Burden of Cryptococcosis at the Time of HIV Diagnosis: A Retrospective Multicenter Study. Infect Dis Ther **2021**; 10(3): 1363-77.
12. Blankley S, Gashu T, Ahmad B, et al. Lessons learned: Retrospective assessment of outcomes and management of patients with advanced HIV disease in a semi-urban polyclinic in Epworth, Zimbabwe. PLoS One **2019**; 14(4): e0214739.
13. Meyer AC, Kendi CK, Penner JA, et al. The impact of routine cryptococcal antigen screening on survival among HIV-infected individuals with advanced immunosuppression in Kenya. Trop Med Int Health **2013**; 18(4): 495-503.
14. Baluku JB, Mugabe P, Mwebaza S, et al. Cryptococcal Antigen Screening Among Antiretroviral Therapy-Experienced People With HIV With Viral Load Nonsuppression in Rural Uganda. Open Forum Infect Dis **2021**; 8(2): ofab010.

15. Mbewe N, Vinikoor MJ, Fwoloshi S, et al. Advanced HIV disease management practices within inpatient medicine units at a referral hospital in Zambia: a retrospective chart review. AIDS Res Ther **2022**; 19(1): 10.
16. Heller T, Damba D, Kumwenda T, et al. Implementing Advanced HIV Disease Care for Inpatients in a Referral Hospital in Malawi - Demand, Results and Cost Implications. Ann Glob Health **2022**; 88(1): 16.
17. Enock K, Julius K, Griffith BC, et al. Evaluation of the initial 12 months of a routine cryptococcal antigen screening program in reduction of HIV-associated cryptococcal meningitis in Uganda. BMC Health Serv Res **2022**; 22(1): 301.
18. Srishyla D, Saemisch G, Turya F, et al. Determinants of cryptococcal antigen (CrAg) screening uptake in Kampala, Uganda: An assessment of health center characteristics. Med Mycol **2022**; 60(4).
19. Tiam A, Paulin H, Machekano R, et al. Rapid antiretroviral therapy initiation in patients with advanced HIV disease: 6-month outcomes of an observational cohort evaluation in Lesotho. PLoS One **2023**; 18(10): e0292660.
20. Vallabhaneni S, Longley N, Smith M, et al. Implementation and Operational Research: Evaluation of a Public-Sector, Provider-Initiated Cryptococcal Antigen Screening and Treatment Program, Western Cape, South Africa. J Acquir Immune Defic Syndr **2016**; 72(2): e37-e42.
21. Drain PK, Galagan SR, Govere S, et al. Laboratory Reflex and Clinic-Based Point-of-Care Cryptococcal Antigen Screening for Preventing Meningitis and Mortality Among People Living With HIV. J Acquir Immune Defic Syndr **2021**; 87(5): 1205-13.
22. Page MJ, McKenzie JE, Bossuyt PM, et al. The PRISMA 2020 statement: an updated guideline for reporting systematic reviews. Bmj **2021**; 372: n71.
23. Amir-Behghadami M, Janati A. Population, Intervention, Comparison, Outcomes and Study (PICOS) design as a framework to formulate eligibility criteria in systematic reviews. Emerg Med J **2020**; 37(6): 387.
24. National Institute for Health. Study Quality Assessment Tools. Available at: https://www.nhlbi.nih.gov/health-topics/study-quality-assessment-tools.
25. R Core Team R: a language and environment for statistical computing. Vienna, Austria: R Foundation for Statistical Computing. Available at: https://www.r-project.org/. Accessed 10 May.
26. Campbell I. Chi-squared and Fisher-Irwin tests of two-by-two tables with small sample recommendations. Stat Med **2007**; 26(19): 3661-75.
27. Higgins JP, Thompson SG, Deeks JJ, Altman DG. Measuring inconsistency in meta-analyses. Bmj **2003**; 327(7414): 557-60.
28. Sing'oei VO, J.; Ganesan, K.; Andagalu, B.; Amusu, S.; Bahemana, E.; Kiweewa, F.; Maswai, J.; Ake, J.; Esber, A.; Crowell, T.A.; Polyak, C. Cryptococcal antigenemia in HIV-infected adults in the African cohort study. American Journal of Tropical Medicine and Hygiene **2017**; 97(5): 2.
29. Kanyama C, Chagomerana MB, Chawinga C, et al. Implementation of tuberculosis and cryptococcal meningitis rapid diagnostic tests amongst patients with advanced HIV at Kamuzu Central Hospital, Malawi, 2016-2017. BMC Infect Dis **2022**; 22(1): 224.
30. Longley NV, S.; Smith, M.; Smith, R.; Osler, M.; Kelly, N.; Cross, A.; Boulle, A.; Meintjes, G.; Govender, N. Evaluation of provider-initiated cryptococcal antigen screening, South Africa. Topics in Antiviral Medicine **2016**; 24(E-1): 2.
31. Hurt WJ, Tenforde MW, Molefi M, et al. Prevalence and Sequelae of Cryptococcal Antigenemia in Antiretroviral Therapy-Experienced Populations: An Evaluation of Reflex Cryptococcal Antigen Screening in Botswana. Clin Infect Dis **2021**; 72(10): 1745-54.
32. Jarvis JN, Boulle A, Loyse A, et al. High ongoing burden of cryptococcal disease in Africa despite antiretroviral roll out. Aids **2009**; 23(9): 1182-3.

33. United States Agency Intenational Development. The need for routine viral load testing: questions and answers. Available at: https://www.unaids.org/sites/default/files/media_asset/JC2845_en.pdf.
34. Okwir M, Link A, Rhein J, et al. High Burden of Cryptococcal Meningitis Among Antiretroviral Therapy-Experienced Human Immunodeficiency Virus-Infected Patients in Northern Uganda in the Era of "Test and Treat": Implications for Cryptococcal Screening Programs. Open Forum Infect Dis **2022**; 9(2): ofac004.
35. Beyene T, Woldeamanuel Y, Asrat D, Ayana G, Boulware DR. Comparison of cryptococcal antigenemia between antiretroviral naïve and antiretroviral experienced HIV positive patients at two hospitals in Ethiopia. PLoS One **2013**; 8(10): e75585.
36. Kambugu A, Meya DB, Rhein J, et al. Outcomes of cryptococcal meningitis in Uganda before and after the availability of highly active antiretroviral therapy. Clin Infect Dis **2008**; 46(11): 1694-701.
37. Butler EK, Boulware DR, Bohjanen PR, Meya DB. Long term 5-year survival of persons with cryptococcal meningitis or asymptomatic subclinical antigenemia in Uganda. PLoS One **2012**; 7(12): e51291.
38. Roberts T, Cohn J, Bonner K, Hargreaves S. Scale-up of Routine Viral Load Testing in Resource-Poor Settings: Current and Future Implementation Challenges. Clin Infect Dis **2016**; 62(8): 1043-8.

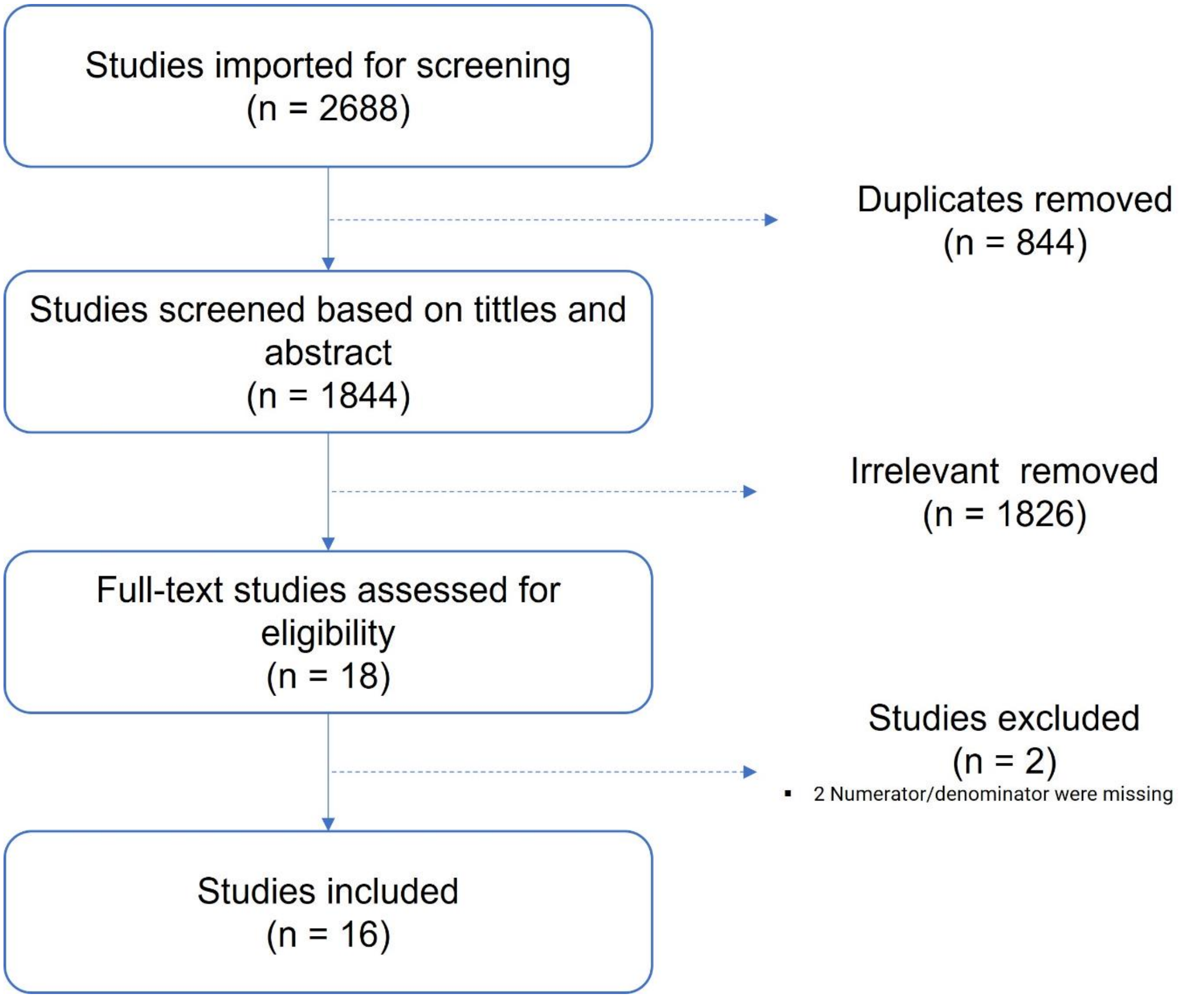

Fig 1. PRISMA flow diagram of the included studies for meta-analysis of on the compliance of WHO guidelines in the management of patients with advanced HIV disease in low- and middle-income countries.

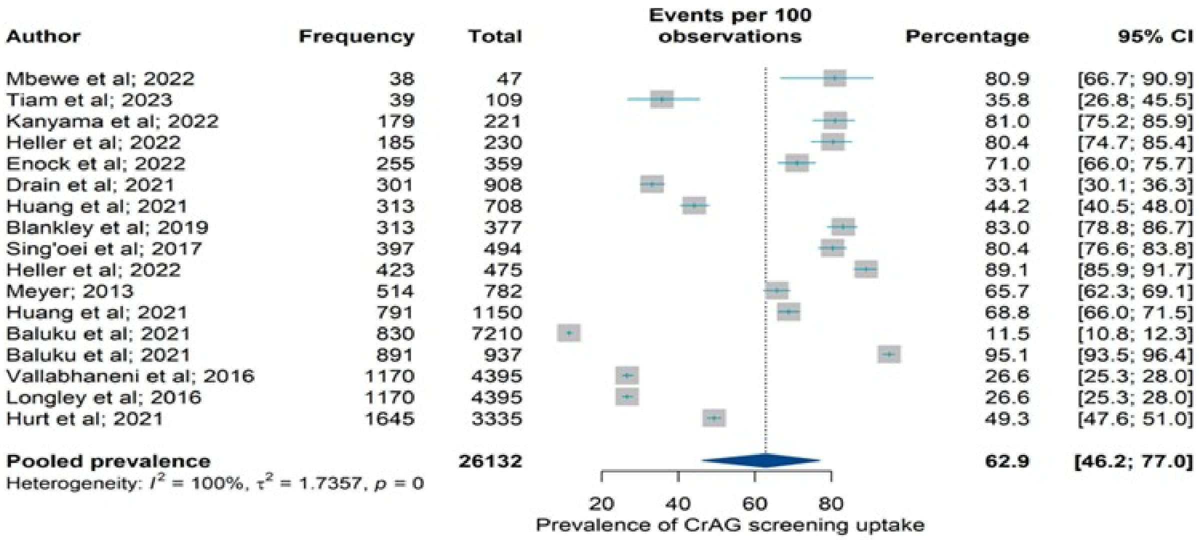

Fig 2. Overall prevalence of CrAG screening uptake among people living with HIV in low- and mid-income countries.

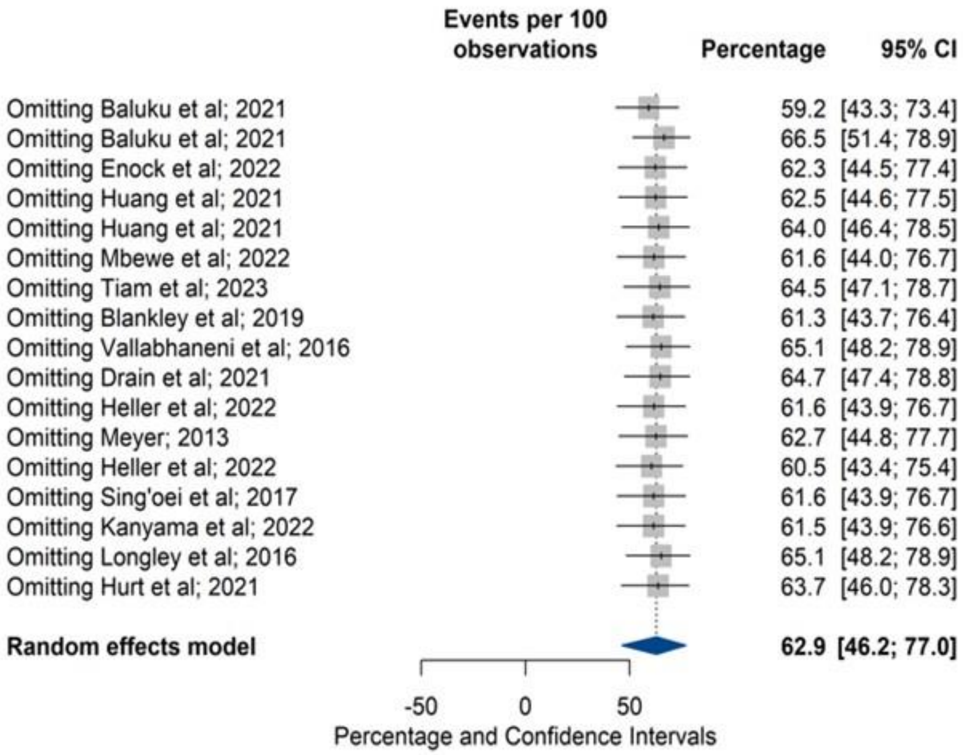

Fig 3. Influential analysis on the overall prevalence of CrAG screening uptake among people living with HIV in low- and mid-income countries.

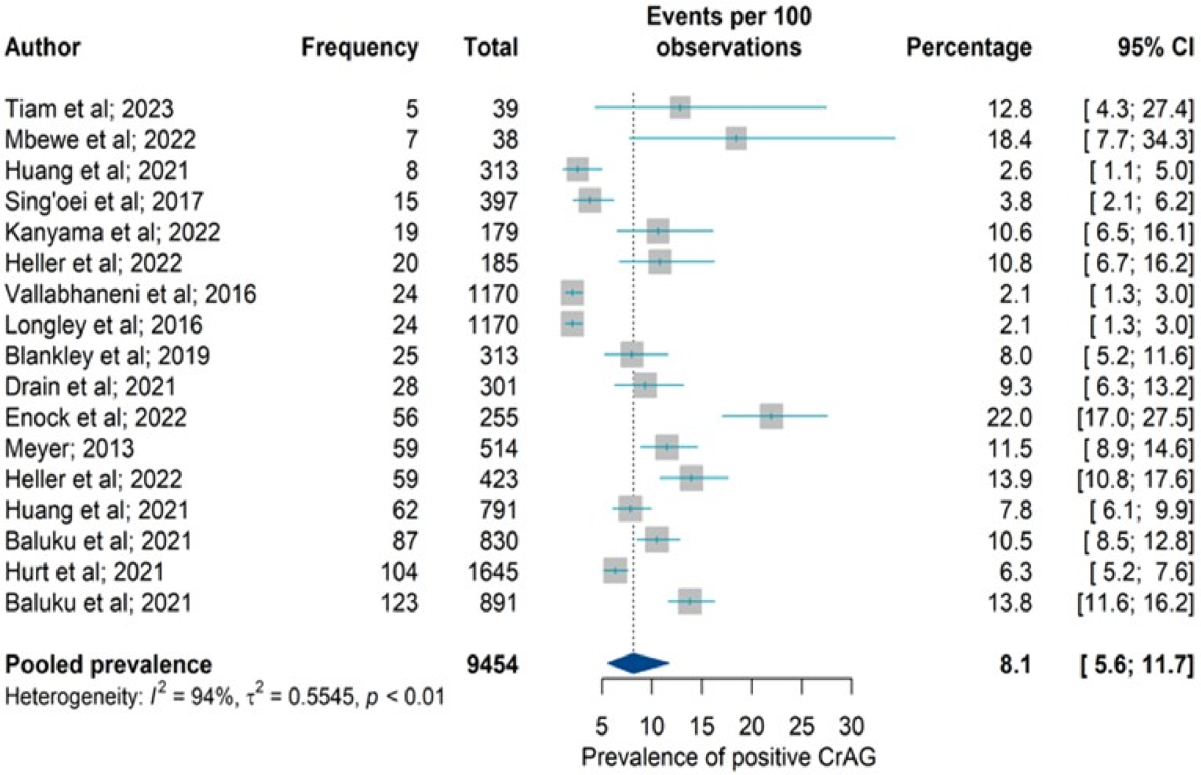

Fig 4. Overall prevalence of cryptococcal antigenemia among people living with HIV in low- and mid-income countries.

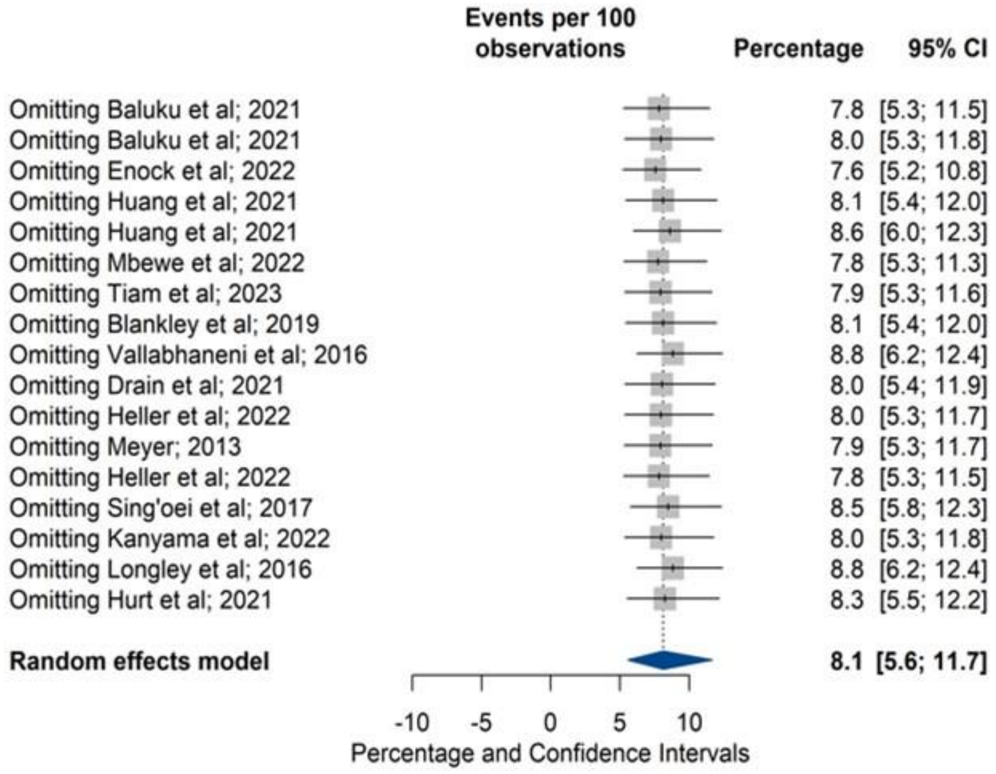

Fig 5. Influential analysis on the overall prevalence of cryptococcal antigenemia among people living with HIV in low- and mid-income countries.

Click here to access/download

Supporting Information - Compressed/ZIP File Archive

Support information.zip
