# Supplementary material for: Systematic review on the compliance of WHO guidelines in the management of patients with advanced HIV disease in Africa: The case of cryptococcal antigen screening
Source: PLoS One. 2025 Jan 24;20(1):e0313453. doi: 10.1371/journal.pone.0313453 (PMC11761098; doi:10.1371/journal.pone.0313453)
Supplement: Attachment — Submitted filename: Response to reviewers.docx [file pone.0313453.s009.docx]

Dear editors PLOS ONE journal,

This letter serves as response to reviewer’s comments for the manuscript “**Systematic review on the compliance of WHO guidelines in the management of patients with advanced HIV disease in Africa: The case of** **cryptococcal antigen screening.**” We greatly appreciate editors and reviewers and believe their comments have significantly improved our manuscript.

Below are point by point responses

Reviewer #1: This is a revised manuscript I reviewed before. From the reviewer's response to my comments and other comments, the authors addressed the reviewer's comments point-by-point and now it is suitable for publication.

**We are grateful we managed to respond to all your earlier comments.**

Reviewer #3: Comments:

Line 28-29: this statement on CrAg negative seems to be a bit less relevant to the focus of the paper.

**We thank you for your comments. The statement has been revised.**

line 31: Please make it clear that the estimates you derived are from available selected published studies. Stating that the stimates are for "Africa" gives the impression that the estimates in this analysis are much more comprehensive than they are.

**We thank you for your comments. The statement has been revised.**

Line 44-45: Suggest making it clear that the prevalence of antigenemia is what was found to be lower in the multi-site studies versus single sites

**We thank you for your comments. Both CrAg uptake and the prevalence of antigenemia were lower in studies that involved multiple sites vs single sites. Line 44-45 refers to CrAg uptake comparisons (multi vs single site) while line 46-48 refers to prevalence of antigenemia comparisons (multi vs single site).No changes were made.**

Lines 90-91: Suggest making it clear that this line is referring to a different study (ref #11) and maybe also commenting briefly on the differences between the ref #10 and ref #11in terms of included studies and locations.

**We thank you for your comments. We have provided some additional details about these two references.**

lines 99-100: Please avoid making statements that imply a national estimate when referencing studies/analyses which had only a few sites (or only one).

**We thank you for your comments. We made changes where these statements were made.**

line 100-101: It appears that this statement on improved coverage in ART naive patients in Uganda may be referencing a study that was analysing ART-experienced patients with viral unsuppression; please review and edit as needed

**We thank you for your comments. We have edited this section.**

Lines 307-310: Somewhere in the limitations section, I suggest commenting even more on the reasons why the estimates of CrAg screening coverage, pre-emptive treatment, and coverage of LP might have been higher in this analysis versus what is likely occurring under routine HIV program settings (i.e. in a setting where study is being conducted things like LPs, fluconazole, CrAg kits, etc are more likely to be avialable) and why the true coverage may be lower in reality. Could use this to mention the need for more programmatic HIV program data on these interventions.

I also suggest adding more mention of the combination of inpatient and outpatient settings included in the reviewed papers (prevalence of CrAg typically going to be higher in HIV+ inpatients versus outpatients).

**We thank you for your comments. We have incorporated these suggestions in the limitation section.**

Line 354-355: Lumbar puncture itself is not a test, it’s the procedure needed to derive the CSF specimen which would then be tested for cryptococcus.

**We thank you for your comments. We have edited this section**

Reviewer #5: The authors have responded adequately to the comments. Therefore, this revised manuscript is suitable for publication.

**We are grateful we managed to respond to all your earlier comments.**
